# Design of Hybrid Bioactive Peptides Derived From Cecropin and Cathepsin for Therapeutic Application

**DOI:** 10.1111/cbdd.70193

**Published:** 2025-11-07

**Authors:** Gabriele Santos Cepinho, Bruna Vitória Scavassa, Gabrielle L. de Cena, Vitor Martins de Andrade, Luís Roberto F. Lima, André Zelanis, Montserrat Heras, Miguel A. R. B. Castanho, Katia Conceição

**Affiliations:** ^1^ Laboratory of Peptide Biochemistry Federal University of São Paulo – UNIFESP São José dos Campos Brazil; ^2^ Functional Proteomics Laboratory Federal University of São Paulo – UNIFESP São José dos Campos Brazil; ^3^ Institute of Food Agricultural Technology (INTEA), Campus Montilivi, University of Girona Girona Spain; ^4^ GIMM ‐ Gulbenkian Institute for Molecular Medicine Lisboa Portugal

**Keywords:** ADMET, antimicrobial peptides, bioactive peptides, cecropin, in silico prediction

## Abstract

The escalating global health crisis of antimicrobial resistance demands the urgent development of novel therapeutic agents with new mechanisms of action. Bioactive peptides (BAPs), and specifically antimicrobial peptides (AMPs), represent a highly promising class of candidates due to their broad‐spectrum activity and superior biocompatibility compared to conventional antibiotics. This manuscript presents a novel approach to drug discovery by designing multifunctional hybrid peptides through the strategic fusion of conserved domains from cecropin and cathepsin‐derived sequences. We established an integrated in silico pipeline, utilizing machine learning for activity prediction and comprehensive ADMET profiling to rationally select three lead candidates with optimal physicochemical properties. Experimental validation confirmed their potent efficacy in vitro, demonstrating significant inhibition of both planktonic cultures and resilient biofilms. Critically, these peptides displayed a high safety profile, with no toxicity in erythrocyte or *Galleria mellonella* larvae models. To elucidate their mode of action, target fishing and molecular docking studies were conducted, revealing high‐affinity interactions with essential *E. coli* enzymes, DNA gyrase, and thymidylate synthase. By combining computational design with robust biological validation, this work establishes a streamlined framework for accelerating anti‐infective discovery and positioning these engineered hybrid BAPs as a promising class of antimicrobial agents.

## Graphical abstract

This study designs hybrid bioactive peptides from cecropins and cathepsins, showcasing their antimicrobial potential. Notably, we identify critical interactions with microbial targets, DNA gyrase and thymidylate synthase in *E. coli* , revealing insights into their mechanisms of action as promising novel antimicrobial agents.

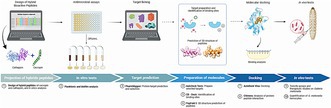

## 1 Introduction

The exploration of bioactive compounds is pivotal in biochemistry and pharmacology, providing essential insights that drive innovation in drug development. Bioactive peptides (BAPs), have gained significant attention due to their ability to modulate biological processes through specific interactions, making them valuable candidates for therapeutic applications. Their small size and inherent specificity enable them to inhibit protein–protein interactions effectively, a mechanism that supports their potential as drug candidates (Akbarian et al. 2022).

Historically, the exploration of peptide hormones such as insulin, oxytocin, and gonadotropin‐releasing hormones has catalyzed advancements in pharmacology. Pioneering researchers, including Banting, Macleod, and Sanger, contributed fundamental knowledge that shaped modern drug discovery (Henninot et al. 2018). The successful synthesis of these early peptide drugs represented a significant milestone, leading to an expansion in the peptide therapeutics market, which now boasts over 80 approved peptide drugs globally. Notable examples include glucagon‐like peptide‐1 (GLP‐1) analogs for diabetes management and calcitonin for osteoporosis treatment, illustrating the diverse therapeutic applications of peptides (D'Hondt et al. 2014).

Despite these advancements, the pharmaceutical landscape faces a growing threat from antimicrobial resistance (AMR). This issue poses one of the primary global threats to public health, with an estimated 1.27 million deaths directly attributable to resistant bacterial infections in 2019, contributing to a total of 4.95 million deaths worldwide. Projections suggest that, without immediate interventions, drug‐resistant infections could lead to up to 10 million deaths annually by 2050 (Mwangi et al. 2019; World Health Organization 2021). This alarming statistic highlights the urgent need for alternative therapeutic strategies.

In this context, antimicrobial peptides (AMPs) have emerged as promising alternatives to traditional antibiotics. AMPs exhibit broad‐spectrum activity and unique mechanisms of action that can circumvent resistance, including disruption of bacterial membranes and modulation of immune responses, making them effective against a wide range of pathogens, including gram‐positive and gram‐negative bacteria, fungi, and viruses (Mura et al. 2016; Yeung et al. 2011). However, the clinical application of AMPs is hindered by several limitations, including low oral bioavailability, rapid proteolytic degradation, and high production costs (Muttenthaler et al. 2021). These challenges restrict the commercial viability of many peptide candidates, leading to stagnation in the development of peptide‐based therapies.

To address these limitations, the rational design of peptides through in silico methods represents a modern strategy. Computational models can simulate biological interactions, enabling researchers to design novel peptides and predict their biological activities efficiently. This methodology not only accelerates the drug discovery process but also reduces reliance on animal testing, offering ethical and economic advantages (Ekins et al. 2007). Current in silico tools utilize advanced machine learning algorithms, such as Random Forest (RF) and Support Vector Machines (SVM), to refine peptide design and optimize therapeutic properties (Jenssen 2011; Jenssen et al. 2007; Lata et al. 2007). For example, recent studies have successfully employed SVM modeling to design defensins, demonstrating the potential of computational methods to generate peptides with specific biological functions (Kaur et al. 2021).

This study aims to advance the field by designing in silico hybrid peptides that combine the conserved sequences of cecropins and cathepsins. Cecropins are well‐characterized AMPs known for their broad‐spectrum antimicrobial activity, while cathepsins, a family of cysteine proteases, play critical roles in various physiological processes, including immune response modulation and apoptosis (Turk et al. 2012; Yadati et al. 2020). By exploiting the strengths of these two classes of peptides, we seek to identify prototype compounds that exhibit both antimicrobial and antitumor activities.

Despite their therapeutic promise, both cecropins and cathepsins face significant challenges that hinder their effective application. Cecropins are vulnerable to proteolytic degradation, which can diminish their efficacy in physiological environments. Strategies to enhance their stability through structural modifications or delivery systems are essential (Ekengren 1999; Gadwala et al. 2021). Additionally, concerns regarding the cytotoxic effects of cecropins necessitate the development of approaches that enhance selectivity for microbial targets (Brady et al. 2019). On the other hand, cathepsins present challenges related to their dual biological roles, complicating therapeutic targeting and the design of selective inhibitors (Kasperkiewicz et al. 2017; Vizovišek et al. 2015). Effective delivery systems are crucial for cathepsin‐targeting drugs to ensure they reach their intended sites while minimizing degradation and adverse immune responses (Anes et al. 2022; Almalki et al. 2023; Pogorzelska et al. 2018).

Our “hybrid approach” aims to exploit the synergistic effects of cecropins and cathepsins, enhancing therapeutic outcomes while minimizing collateral damage to healthy cells. By focusing on the effects of amphiphilicity and charge on antimicrobial activity, we anticipate reducing the cytotoxicity associated with cecropins. In silico techniques have facilitated the development of engineered peptides with desirable properties, such as antimicrobial and anticancer effects (Quigua‐Orozco et al. 2024; Zhang et al. 2024). As peptides gain prominence over small molecule drugs (Rossino et al. 2023), challenges remain regarding stability, pharmacokinetics, and cytotoxicity (Sharma et al. 2023; Wang et al. 2022).

In summary, this study will evaluate the physicochemical and ADMET profiles of newly predicted hybrid peptides derived from cathepsins and cecropins. We will also explore the antimicrobial and cytotoxic properties of three selected peptides identified as promising candidates. By integrating computational predictions with experimental validation, we aspire to streamline the drug discovery process and contribute to the development of effective treatments for drug‐resistant infections and other pressing health challenges. This integrated approach aims to address the critical need for novel therapeutic strategies in the face of increasing antimicrobial resistance.

## 2 Materials and Methods

### 2.1 Study Design

**FIGURE 1:**
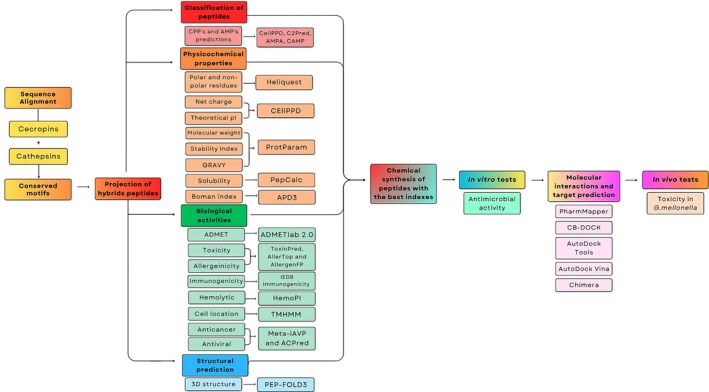
Flowchart of the study.

The development of this study relied on the use of bioinformatics tools to assist in the prospection and selection of potential BAPs for therapeutic application, which were then tested experimentally. The workflow is presented in Figure 1.

### 2.2 Design of Hybrid Peptides and In Silico Analysis

The design of hybrid peptides started with the alignment of the sequences of the BAPs cecropin and cathepsin found in the Swiss‐Prot database on the free UniProt server (https://www.uniprot.org/). This process aimed to identify the conserved amino acid sequences of these well‐characterized biomolecules.

Cecropins exhibit variations in amino acid residues, leading to different designations such as Cecropin, Cecropin A, B, C, and D (Ponnuvel et al. 2010). Consequently, a sequence alignment was conducted for each molecular variant of cecropin. Similarly, cathepsins also display variations in amino acid residues, resulting in classifications such as cathepsins E, F, S, K, and B. However, the available data on these cathepsins is insufficient for separate analyses, prompting an alignment that encompasses all cathepsins. Additionally, an alignment of pro‐cathepsins was performed to enhance the dataset.

With the alignment, it was possible to select 22 amino acid sequences for the projection of the hybrid peptides. The conserved sequences obtained from the alignments were concatenated and two variations of the hybrid peptides were constructed. First, in the N‐terminal of the projected peptides the cecropin sequences were placed and, in the C‐terminal the cathepsin sequences. Subsequently, the N‐terminal of the engineered peptides was formed by the cathepsin sequences and the C‐terminal by the cecropins. From all these variations, it was possible to design 192 hybrid peptide sequences for further bioinformatics analyses. To facilitate such analyses, the hybrids were placed in the fasta text format.

#### 2.2.1 CPP's and AMP's Prediction

AMPs and CPPs have several overlapping features, especially regarding their structural properties and mechanisms of action. Both types of peptides are generally cationic and amphiphilic, which enhances their interaction with membranes. This amphiphilicity is essential for AMPs to disrupt bacterial membranes, while it enables CPPs to penetrate cellular membranes with minimal disruption (Henriques et al. 2006; Oh et al. 2014). The cellular penetration capacity of the conserved sequences and the projected hybrids was predicted with the use of the web servers CellPPD (https://webs.iiitd.edu.in/raghava/cellppd/index.html) and C2Pred (http://lin‐group.cn/server/C2Pred), which make use of the SVM score classifier. The antimicrobial potential of the same conserved sequences and the projected hybrids was predicted with the aid of the bioinformatics tools AMPA (http://tcoffee.crg.cat/apps/ampa/do), CAMP algorithm (http://www.camp.bicnirrh.res.in) and AmpGram (http://biongram.biotech.uni.wroc.pl/AmpGram).

#### 2.2.2 Physicochemical Profile Prediction

The characterization of the physicochemical properties of the engineered hybrids was conducted using various bioinformatics tools. The ProtParam server, part of ExPASy and hosted by the Swiss Institute of Bioinformatics, was utilized to estimate molecular weight (MW), instability index, and GRAVY. Additionally, CellPPD provided theoretical values for the isoelectric point and net charge of the peptides. The PepCalc server was employed to assess the potential for water solubility. The values of hydrophobic moment (μH), hydrophobicity (H), and amino acid charge were estimated using Heliquest and the Antimicrobial Peptide Database (APD3).

#### 2.2.3 Evaluation of Membrane Binding Capacity

The APD3 software (https://aps.unmc.edu/prediction/predict) was employed to calculate the Boman Index, which represents the sum of the solubility values of all amino acids in a peptide sequence. This allows for an inference of the peptide's ability to bind to cell membranes or other proteins. Further to this analysis, the TMHMM server (http://www.cbs.dtu.dk/services/TMHMM) was utilized to determine the cellular localization of the engineered peptide sequences and their potential for binding to negatively charged bacterial cell membranes.

#### 2.2.4 Evaluation of Immunogenicity, Toxicity, Allergenicity and Anticancer and Antiviral Properties

To ensure the therapeutic capacity of the engineered peptides, their sequences were submitted to in silico evaluations of immune response. The IEDB Immunogenicity Predictor (http://tools.iedb.org/immunogenicity) server was used to evaluate the immunogenicity of the designed sequences. Toxicity and allergenicity were analyzed using ToxinPred (https://webs.iiitd.edu.in/raghava/toxinpred/algo.php), AllerTop (https://www.ddg‐pharmfac.net/AllerTOP), and AllergenFP (http://ddg‐pharmfac.net/AllergenFP). Antiviral and anticancer peptides were predicted using Meta‐iAVP (http://codes.bio/meta‐iavp) and ACPred (http://codes.bio/acpred), respectively.

#### 2.2.5 Hemolytic Activity and Half‐Life

ProtParam (https://web.expasy.org/protparam) also provided the half‐life of hybrid peptides designed into *E. coli* , yeast, and mammals. Hemolytic activity was predicted in the HemoPI server (https://webs.iiitd.edu.in/raghava/hemopi/design.php).

#### 2.2.6 Prediction of ADMET

Based on the in silico data obtained and the parameters provided by the computational tools used, comparative analysis was carried out with the aim of selecting the hybrid peptide sequences with better potential for cell penetration, antimicrobial action and physicochemical profile. The MarvinSketch program (//marvinjs‐demo.chemaxon.com/latest/demo.html) was used to obtain the SMILES code (Simplified Molecular Input Line Entry Specification) of the designed hybrid peptides. This text format carries molecular information of the sequences that are loaded into various other in silico tools to obtain pharmacokinetic and pharmacodynamic properties. Thus, the selected peptides, in the Simplified Molecular Input Line Entry System (SMILES) format, were submitted to the ADMETlab 2.0 platform (https://admetmesh.scbdd.com) for prediction of pharmacokinetic parameters, including human intestinal absorption (HIA), mutagenicity, carcinogenicity, central nervous system penetration, drug‐induced liver injury (DILI), cytochrome P450 enzyme inhibition, carcinogenicity, mutagenicity, clearance, half‐life, and skin sensitization. Still on the ADMETlab 2.0 platform, pan‐assay interference compounds (PAINS) and undesirable reactive compounds were predicted using PAINS and Pfizer rules.

#### 2.2.7 Structure Prediction

The PEP‐FOLD3 software (https://bioserv.rpbs.univ‐paris‐diderot.fr/services/PEP‐FOLD3/) was applied to project peptide structures from their primary amino acid sequences. Thus, along with the peptide helical wheel diagram provided by Schiffer, the Edmundson wheel modeling using Heliquest evaluated the quality of the best models, and the Ramachandran diagram was predicted for each of them using the SAVES v6.0 server; the structural analysis was carried out.

### 2.3 Peptides Chemical Synthesis

Selected hybrid peptides C‐terminal amide were synthesized manually by the solid‐phase method following a 9‐fluorenylmethoxycarbonyl (Fmoc)/*tert‐butyl* (*t*‐Bu) protocol (Amblard et al. 2006), in polypropylene syringes fitted with a polyethylene porous disk. Solvents and soluble reagents were removed in vacuum. Commercially available reagents were used throughout without purification. A Fmoc‐Rink‐MBHA resin (0.71 mmol/g) was used for the synthesis of sequences containing up to 15 residues and for longer sequences an aminomethyl ChemMatrix resin (0.62 mmol/g) was employed as a solid support. Coupling of Fmoc‐Rink (4 equiv) onto the aminomethyl ChemMatrix resin was mediated by DIC (4 equiv) and Oxyma (4 equiv) in DMF at room temperature overnight. Fmoc group removal was achieved with piperidine‐DMF (3:7, 1 × 2 min + 2 × 10 min). Coupling of commercial Fmoc‐amino acids (4 or 3 equiv) was performed using DIC (4 or 3 equiv) and Oxyma (4 or 3 equiv) in DMF under stirring at room temperature for 4 or 8 h, and monitoring by the Kaiser test (Kaiser et al. 1970). For each coupling and deprotection step, the resin was washed with DMF (6 × 1 min) and CH_2_Cl_2_ (3 × 1 min) and aired‐dried. After coupling of the ninth amino acid residue, NMP was used instead of DMF. Peptide elongation was performed by repeated cycles of Fmoc removal, coupling and washings. Once the synthesis was completed, peptidyl resins were subjected to the N‐terminal Fmoc removal. Then, the peptides were cleaved by treatment with TFA‐H_2_O‐Phenol‐TIS (88:5:5:2) for 2 h. Following TFA evaporation, peptides were precipitated by adding cold diethyl ether and collected by centrifugation. Finally, the crude peptides were purified by reverse‐phase column chromatography, lyophilized, analyzed by HPLC, and characterized by high‐resolution mass spectrometry (HRMS) and proton nuclear magnetic resonance (^1^H‐NMR) (Appendix S1).

### 2.4 In Vitro Analysis

#### 2.4.1 Antimicrobial Assays (Planktonic and Biofilm Analysis)

The antimicrobial activity of selected peptides was evaluated through colony‐forming unit (CFU/mL) assays, following the analytical parameters of the NCCLS method (NCCLS, 2002). To perform the assays, different concentrations of each of the peptides were tested against the bacterial strains *Escherichia coli* (ATCC 25922) and *Staphylococcus aureus* (ATCC 6538), as well as the fungal strain *Candida albicans* (ATCC 10231). The action of the hybrid peptides was analyzed against these strains using a sterile 96‐well plate with inocula standardized to a concentration of 10^3^ cells/mL. The bacterial pre‐inocula were prepared in Mueller Hinton Broth (MHB—Kasvi), and brain‐heart infusion (BHI—Kasvi) was used for the fungal strains, incubated for approximately 24 h at 37°C. Plating was performed in duplicate, and absorbance readings were taken corresponding to the standard absorbance for *E. coli* (600 nm), *S. aureus* (630 nm), and *C. albicans* , using a microplate reader (Synergy H1, BIOTEK, USA). Positive controls included ampicillin (1 mg/mL) for gram‐negative bacteria, tetracycline (1 mg/mL) for gram‐positive bacteria, and amphotericin (32 μg/mL) for fungi. The inoculum of each microorganism was used as the negative control. The samples were evaluated by the colony‐forming unit (CFU) drip method assay, following the procedure described by Herigstad et al. (2001), which involves taking 20 μL aliquots from the wells, followed by serial dilution in 180 μL of 0.9% saline solution and subsequent plating on agar plates. The plates were then incubated for 24 h, after which the number of colonies was counted and used to calculate the cell density per mL (CFU/mL) in the original culture.

Biofilm inhibition assays were performed as previously described (Martins de Andrade et al. 2020). Microorganisms were cultivated in appropriate broth medium and adjusted to a density of 10^5^ cells/mL. To induce biofilm formation, 200 μL of the cell suspension was inoculated into 96‐well polystyrene microplates and incubated for 24 h at 37°C, with the microplates kept at rest. After this period, the broth was removed, and the wells were washed three times with 0.9% NaCl solution to ensure the preservation of the biofilm adhered to the bottom of the wells. Then, 200 μL of the peptide solution at concentrations of 5 and 10 times the MIC (of each peptide) were added to evaluate biofilm inhibition. As a negative control, the inoculum was prepared only with the broth, without the addition of peptides, to evaluate the natural formation of the biofilm. After 24 h of treatment, adherent cells were detached by sonication using a probe sonicator (Vibra‐cell, VCX 130 SONICS ultrasonic processor, USA), applying an amplitude of 50% for 3 min in each well. Biofilm formation quantification was carried out by counting Colony Forming Units (CFU/mL), as previously described. All experiments were performed in triplicate to ensure the reproducibility of the results.

#### 2.4.2 Cytotoxicity and Hemolytic Assay

The cell viability of human umbilical vein endothelial cells (HUVEC CS) (Gifford et al. 2004) and human melanoma cell line (A375 CRL 1619, ATCC, USA) was evaluated in the presence of the studied peptides through a cytotoxicity assay (Riss et al. 2004). HUVEC and A375 cells were pre‐prepared in 100 μL of DMEM culture medium containing 10% Fetal Bovine Serum (FBS) for the assay, at a concentration of 10,000 cells per well and incubated for 24 h at 37°C with 5% CO_2_. After 24 h, the wells were washed twice with PBS (without divalent cations), followed by the addition of 95 μL of culture medium and 5 μL of the peptide (dissolved in cultured medium), using three biological replicates. After 24 h of exposure to the peptides, 100 μL of the medium from each well was removed and replaced with 100 μL of MTT (3‐[4,5‐dimethylthiazol‐2‐yl]‐2,5‐diphenyltetrazolium bromide) at a concentration of 0.05 mg/mL in DMEM. The plate was then incubated for 3 h under the same conditions. Following the MTT incubation period, 100 μL of DMSO was added to each well, and the plate was shaken overnight. The multi‐well plate was quantified by measuring absorbance at 570 nm using a spectrophotometer (Synergy H1, BIOTEK, USA). Cell viability was normalized against a control in which no stimuli were added (i.e., only DMEM culture medium +10% FBS).

To perform the hemolytic test, human red blood cells were obtained from a volunteer donor. After centrifugation at 1000 × g for 5 min, the red blood cell pellets were resuspended in 5% (vol/vol) sterile saline at different concentrations of hybrid peptides and subsequently incubated at 37°C for 1 h. The supernatants were transferred to a 96‐well plate, and the absorbance at 570 nm (A570) was measured. 0.12% DMSO and 1% TritonX‐100 were used as negative and positive controls, respectively (Conceicao et al. 2006). The hemolysis rate was calculated as follows:

$$\text{Hemolysis}\%=\left[A_{\left(\text{sample}\right)}-A_{\left(\text{DMSO}\right)}\right]/\left[A_{\left(\text{Triton}\right)}-A_{\left(\text{DMSO}\right)}\right]\times 100$$

This study was approved by Plataforma Brasil through the number 71282023.5.0000.5505.

### 2.5 In Vivo Studies

#### 2.5.1 Toxicity Assays and Therapeutic Studies on *Galleria mellonella*

For this study, the methodologies described by Mylonakis et al. 2005 and Jorjão et al. 2018 with brief modifications were employed. *G. mellonella* larvae in their final larval stage were used for the experiment. Ten randomly selected *G. mellonella* larvae of similar weight and size (250–350 mg) were used per group in all assays. Syringes (Hamilton Inc., USA) used for injections were sterilized with peracetic acid (Henkel—Ecolab GmbH, Düsseldorf, Germany) according to the manufacturer's instructions prior to a concentration of 1500 μg/mL, 800 μg/mL, 200 μg/mL, and 100 μg/mL into the last left proleg. A control group was injected with PBS to assess overall viability. The number of deceased *G. mellonella* was recorded every 24 h after peptide injection, with monitoring continuing for 7 days across three independent experiments. Larvae were considered dead if they showed no movement upon touch. The experiment concluded either when all larvae in the experimental group had died or transitioned into the pupal form (dos Santos et al. 2017).

To evaluate the action of the peptides, the lethal concentration of selected microorganisms' inoculum was determined by injecting serial dilutions of the cell suspension into the larvae. Cells were centrifuged and washed with 0.9% NaCl, and cell density was standardized to 10^8^ cells/mL using spectrophotometry. The concentrations of 10^5^, 10^6^, 10^7^, and 10^8^ cells/mL were tested in the assay. Then, 10 μL of the standardized suspension were injected into the last pair of prolegs of each larva, with the aim of identifying the most lethal cell density. The larvae were incubated in Petri dishes at 37°C, and survival was monitored daily for a period of 7 days.

In order to observe therapeutic activities, the selected peptides were injected into the larvae at their respective MIC, using the last right pseudopod as the administration site. Sequentially, a suspension in Mueller‐Hinton broth containing microorganisms in 10^8^ bacterial cells/mL (10 μL) was injected into the hemocoel of the larvae through the last left pseudopod. The larvae were incubated at 37°C, and survival was monitored daily for a period of 7 days. Larvae were considered dead when they did not respond to tactile stimuli, and mortality or transition to pupae was assessed at the end of the experiment.

#### 2.5.2 Quantification of *G. mellonella* Hemocytes

The larvae were infected with selected microorganisms (10^8^ cells per larvae) by injecting the microorganisms into the last left proleg and, subsequently, subjected to treatment with the injection of 10 μL of peptides at MIC, applied to the last right proleg. The larvae were incubated at 37°C for 6 h. At 3‐h intervals, the larvae were cut in the cephalocaudal direction, using a scalpel blade, and the hemolymph was collected and transferred to a microtube. The microtubes contained a cold, sterile insect physiological saline (IPS) solution (150 mM sodium chloride; 5 mM potassium chloride; 100 mM Tris‐hydrochloride, pH 6.9 with 10 mM EDTA and 30 mM sodium citrate). Then, 10 μL of suspended hemocytes were mixed with 10 μL of 0.4% trypan blue dye, and 10 μL of this mixture was loaded under the coverslip of a hemocytometer previously cleaned with 70% ethanol. Viable cells remain colorless, as they do not absorb the trypan blue dye. Cell counting was performed in the four outer quadrants, allowing the calculation of hemocyte density per milliliter. The trypan blue staining method has been previously demonstrated by Senior and Titball (2021) to evaluate cell viability in hemocytes.

### 2.6 Circular Dichroism Studies

Circular dichroism spectra of 50 μM of the peptides in 10 mM HEPES buffer, 50 mM NaF, pH 7.4, in the absence or presence of 30 mM of Sodium Dodecyl Sulphate (SDS), were acquired at 25°C in the 190–260 nm wavelength range using 0.1 cm quartz cells in a JASCO model J‐815 spectropolarimeter (Tokyo, Japan). Each final spectrum corresponded to an average of five scans, which were subsequently corrected for buffer or SDS baseline.

### 2.7 Target Prediction

To identify the potential protein targets of each peptide, the PharmMapper web server (www.lilab‐ecust.cn) (Wang et al. 2017) was used, which employs the pharmacophoric mapping approach. The adopted parameter set included the use of generating conformers, maximum conformations generated (300), complete pharmacophoric mapping selecting all targets (v2010, 7302), and the number of reserved corresponding targets (300). The obtained results were ranked based on the normalized fit score and subsequently filtered, focusing on proteins isolated in the gram‐negative microorganism, *E. coli* .

### 2.8 Ligand Binding Site Prediction and Molecular Docking

The binding sites of the target receptors were predicted using the CB‐Dock software (CB‐Dock2: An accurate protein‐ligand blind docking tool [labshare.cn]) (Liu and Cao 2024), a docking tool that focuses on improving docking accuracy. CB‐Dock predicts binding regions of a given protein and calculates the centers and sizes of these regions using a curvature‐based cavity detection approach. The PDB file of each protein was used for the localization of the sites. The results consisted of the coordinates of five different sites for each protein, which were subsequently tested to obtain the best molecular docking.

Molecular docking was performed between the previously identified and selected protein targets and the peptides of interest. The structures of the target proteins were downloaded from the Research Collaboratory for Structural Bioinformatics Protein Data Bank (RCSB‐PDB; https://www.rcsb.org), and then prepared using the AutoDockTools v1.5.7 (AutoDockTools – AutoDock Suite (scripps.edu)) and Discovery Studio (BIOVIA Discovery Studio|Dassault Systèmes (3ds.com)) software to configure the proteins by eliminating solvents and associated ligands, adding hydrogen atoms, and assigning Gasteiger charges. The prepared proteins and peptides were then saved in PDBQT format. Molecular docking between peptides and target proteins was performed using AutoDock Vina software (AutoDock Vina (scripps.edu)). Considering the conformational stability of both the ligand and the protein, binding energy was adopted as a screening criterion, with a threshold set at ≤ −23.01 kJ/mol.

### 2.9 Analysis of Ligands and Receptors Interaction

The interaction between peptides and protein receptors was analyzed using the UCSF Chimera software (https://www.cgl.ucsf.edu/chimera/) (Pettersen et al. 2004). The FindHBond tool was used with a relaxed bonding restriction (2Å and 20°) to examine the interactions. In this study, only hydrogen bonds with a distance of ≤ 4 Å between heavy atoms were considered. Additionally, a comparison was made between the receptor binding sites and the sites that showed the best docking energy results. Information about residual electron donors and acceptors was also collected. Using the PDBsum server (PDBsum home page (ebi.ac.uk)), the PDB code of the enzymes was used to compare with the binding sites obtained from docking.

### 2.10 Statistical Analysis

To calculate the MIC values (planktonic cells), the data were converted into percentage of inhibition, the results of the colony forming units (CFU) in Log10 of three means of the triplicates. The analyses were carried out in the GraphPad Prism program using the variance test (ANOVA) and Tukey's multiple comparison test. For the survival experiments in *G. mellonella*, the survival curve was performed using the Kaplan–Meier method and the significance level was calculated using the Log‐rank test (Mantel‐Cox). The GraphPad Prism program was used in all tests, with a significance level of 5%. All tests were performed three times on different days.

## 3 Results and Discussion

### 3.1 Design of Hybrid Peptides

#### 3.1.1 Identification of Conserved Sequences Between Cecropins and Cathepsins

We analyzed the alignment of cecropins annotated in UniProt, identifying the most conserved amino acid sequences that form the peptides: KIGKKIERVGQH (Cecropin A; Figure S1B), KLGKKIE (Cecropin B; Figure S1C), and KLGKRIERIGQ (Cecropin C; Figure S1D). These sequences were found in 5 cecropin A sequences, 6 cecropin B sequences, and 9 cecropin C sequences, respectively. Notably, the sequence KLGKKIE, conserved in cecropin B (Figure S1C), shows significant similarity to the sequences of cecropins A (Figure S1B) and C (Figure S1D). The structure of cecropins typically consists of 34–39 amino acids with an N‐terminal amphipathic α‐helix, an AGP (Alanine, Glycine, Proline) hinge, and a hydrophobic C‐terminal α‐helix (Wu et al. 2022). The selected conserved sequences are localized on the N‐terminal amphipathic α‐helix, right before the AGP ringe.

Among the 7 amino acids, only the 2nd and 5th differ, yet they exhibit similar functional structures. The 2nd amino acid residue varies between Leucine (L) and Isoleucine (I), both of which are nonpolar. Similarly, the 5th amino acid can be either Lysine (K) or Arginine (R), both classified as basic amino acids.

The analysis of cathepsins focused on the following two sequences: HAVLVVGYG (Figure S2A) and HGVLVVGYG (Figure S2A). These sequences differ only at the 2nd amino acid, which alternates between A (alanine) and G (glycine); both are nonpolar amino acids with similar properties. Pro‐cathepsins were also analyzed, resulting in four additional sequences. The first pair of conserved sequences identified are PVKNQGACGSCWTFS (Figure S2B) and PVKNQGQCGSCWAFS (Figure S2B). In this pair, when the 7th amino acid is nonpolar, the 13th is polar, and vice versa. The last pair of conserved sequences selected consists of YWIVKNSWG (Figure S2B) and YWLVKNSWG (Figure S2B), where the only difference lies in the 3rd amino acid, which can be either I (isoleucine) or L (leucine). Since both are apolar, they exhibit similar functional structures. As results of the alignments, 16 sequences derived from cecropins sequences, and 6 sequences derived from cathepsins were obtained (Table S1).

Cecropins are a class of peptides recognized for their antimicrobial and antifungal properties, playing a vital role in the innate immune response of certain insects (Ekengren 1999). However, their cationic nature and high toxicity can also cause the lysis of healthy cells. Research by Brady et al. (2019) suggests that combining cecropins with other BAPs, such as cathepsins, holds promise. These proteases possess active catalytic activity that can reduce toxicity and enhance cellular penetration, which are crucial attributes for the therapeutic potential of these molecules.

After obtaining the conserved amino acid sequences between cecropins and cathepsins, we proceeded to project hybrid peptides by interchanging the sequences of cecropins and cathepsins between the N‐terminal and C‐terminal regions. This concatenation of 16 cecropin sequences and 6 cathepsin sequences resulted in 192 hybrid sequences, detailed in the table below (Table S2). The nomenclature of these peptides is based on the position (N‐terminal or C‐terminal) of the conserved sequence within the new peptide. For instance, the hybrid CEC1_CATK is composed of the first conserved sequence from Cecropin at the N‐terminal and the conserved sequence from Cathepsin K at the C‐terminal region.

### 3.2 In Silico Analysis of the Designed Hybrids

#### 3.2.1 Evaluation of CPP's and AMP's Profiles

AMPs and CPPs have several overlapping features, especially regarding their structural properties and mechanisms of action. Both types of peptides are generally cationic and amphiphilic, which enhances their interaction with membranes. This amphiphilicity is essential for AMPs to disrupt bacterial membranes, while it enables CPPs to penetrate cellular membranes with minimal disruption (Henriques et al. 2006; Oh et al. 2014). Recent research has investigated the potential of hybrid peptides that integrate characteristics of both AMPs and CPPs. These hybrid peptides can boost antimicrobial efficacy while maintaining the ability to penetrate cells, presenting a promising strategy for developing new therapeutic agents (Kravchenko et al. 2022, 2023). For instance, fusing CPP sequences with antimicrobial sequences has been shown to enhance effectiveness against both intracellular and extracellular pathogens (Cruz et al. 2022; del Rio et al. 2022). The antimicrobial potential and cell penetration capability of the 192 designed peptides were predicted using the AMPA, CAMP, C2Pred, and CellPPD web servers, and the results are presented in Table S3.

The prediction of CPPs by the CellPPD tool did not identify any CPP sequences; however, the C2Pred tool indicated 4 CPP sequences. CAMP predicted 83 sequences with antimicrobial potential, with scores above 0.9. On the other hand, the four sequences with cell penetration potential varied between 0.73 and 0.84 in AMP scoring. Additionally, the AMPA server showed that 11 sequences had a 0% chance of appearing in sequences with non‐antimicrobial potential (Non‐AMP). This step was necessary to verify which of the designed hybrid peptides retained the cell penetration and antimicrobial potential characteristics of the original cecropin and cathepsin molecules. In addition, one more peptide was designed based on a recommendation from the AMPA software, which identified a new peptide with a high probability of antimicrobial activity from the sequence “CATL1.2_CECB1” (YWLVKNSWGKLGKKIE). This peptide was later designated “CATL1.2_CECB1_2” (WLVKNSWGKLGKKI).

#### 3.2.2 Physicochemical Analysis

**FIGURE 2:**
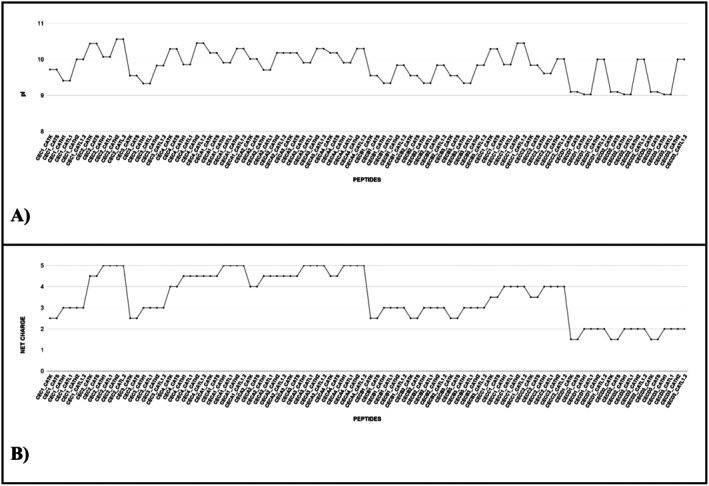
(A) Graph of the isoelectric point. (B) Graph of the net charge of the peptides at pH 7.0.

An analysis of physicochemical characteristics—including isoelectric point (pI), net charge, molecular weight, amphipathicity, solubility in water, and hydrophobicity—was conducted, as these factors influence modes of action (Pirtskhalava et al. 2021). The isoelectric points of the peptides are illustrated in Figure 2A, while the net charges are presented in Figure 2B. All designed peptides are cationic, as confirmed by their positive net charges. This positive charge enhances electrostatic interactions with the plasma membranes of certain pathogens (Tan et al. 2017).

Studies have shown that cathepsins vary in their isoelectric points (pI), which directly influence the variability in the net charge of these proteases (Kozlowski 2022). In contrast, cecropins are cationic peptides that are positively charged, α‐helical, and amphipathic; these physicochemical properties are essential for their antimicrobial activities (Zdybicka‐Barabas et al. 2019). Therefore, it is functionally important that the designed hybrid peptides, which incorporate sequences from both cecropins and cathepsins, retain a positive net charge. This characteristic is likely inherited from the cecropin sequences included in the hybrids.

Factors such as solubility and stability (Table S4) are crucial for facilitating favorable interactions between bioactive compounds and ensuring effective applicability in experimental assays (De Cena et al. 2022). The length of peptides, the proportion of amino acids with hydrophobic properties, and other physicochemical characteristics (Table S4) play significant roles in determining the solubility and functional activity of peptides (Al Musaimi et al. 2023). Peptides exhibiting a higher helical hydrophobic moment and greater hydrophobicity typically demonstrate enhanced potential for cellular internalization (Su et al. 2024; Wang et al. 2015).

Cecropins, for instance, are amphipathic peptides characterized by distinct hydrophilic and hydrophobic regions, possessing a significant hydrophobic moment that enhances their interaction with and insertion into bacterial cell membranes, which is critical for their antimicrobial activity (Wang et al. 2015). Alternatively, cathepsins are globular proteases featuring an internal hydrophobic core, optimized to function in aqueous environments such as lysosomes. In this context, their hydrophobicity contributes more to structural stability than to membrane interaction (Turk et al. 2012).

By designing hybrids of cecropins and cathepsins, the amphipathic and hydrophobic properties of cecropins were integrated with the structural stability of cathepsins, leading to the prediction of molecules with enhanced solubility and increased potential for cellular internalization.

#### 3.2.3 Evaluation of Membrane Binding Capacity

To assess membrane binding capacity, we analyzed the Boman index, the protein's binding potential, (Table S4), which is calculated by dividing the LogP coefficient of the respective amino acid side chains by the total number of amino acid residues in the peptide (Boman 2003). Among the analyzed sequences, only 16 peptides exhibit a high index (> 2.48), indicating significant membrane binding potential. Conversely, peptides with indices below 1 are associated with lower toxicity and a reduced likelihood of side effects; of the 192 hybrid peptides examined, 68 possess this characteristic.

#### 3.2.4 Evaluation of Immunogenicity, Toxicity, Allergenicity and Anticancer and Antiviral Properties

Given the complexity of the immune system, predicting immunogenicity, allergenicity, and toxicity (Table S5) is essential in drug development for the success of subsequent experimental studies. The Immune Epitope Database (IEDB) provided scores for hybrid peptides, indicating that higher scores correlate with a greater likelihood of eliciting an immune response. The highest score recorded was 0.70712 for the sequence “GQRIRDAIISAAPAVHAVLVVGYG” from the CECD1_CATK hybrid. Consequently, the engineered hybrids displayed immunogenicity scores ≤ 0.7, suggesting they are unlikely to provoke an immune response (De Cena et al. 2022).

The AllergenFP web server predicted that among the 192 designed peptide sequences, 141 are nonallergenic, while the AllerTOP server identified 105 as nonallergenic. According to guidelines from the Food and Agriculture Organization (FAO) and the World Health Organization (WHO), a protein is considered a potential allergen if it shares 6–8 consecutive amino acid residues or more than 35% overall sequence similarity within an 80 amino acid window compared to known allergens (FAO/WHO 2001). The ToxinPred server, utilizing a dataset of experimentally validated peptides and proteins along with an SVM test model (Dimitrov et al. 2014; Gupta et al. 2013), indicated that all designed sequences were classified as nontoxic.

As BAPs peptides are increasingly recognized as potential sources for therapeutic applications, the antiviral and anticancer capacities (Table S5) of the designed hybrid sequences were predicted. The ACPred and Meta‐iAVP web servers were utilized for anticancer and antiviral predictions, respectively, employing a classification scale from 0 to 1, where scores closer to 1 indicate a higher likelihood of therapeutic efficacy. Among the 192 sequences analyzed, 137 achieved scores greater than 0.9 in anticancer prediction (ACP), while 140 did so in antiviral prediction (AVP). Notably, 51 sequences demonstrated both anticancer and antiviral activity (Schaduangrat et al. 2019).

A literature review of cecropins and cathepsins reveals distinct and significant characteristics pertinent to the development of therapeutic peptides (Brady et al. 2019; Liu et al. 2024; Zhao et al. 2023). Generally, cecropins exhibit robust antimicrobial activity and low immunogenicity, but they can display selective toxicity in certain cell types, which poses challenges for systemic application (Suttmann et al. 2008). Furthermore, cecropins have shown promising antitumor effects against bladder cancer cells, inhibiting cell viability and proliferation while inducing direct cell lysis (Suttmann et al. 2008). In contrast, cathepsins are lysosomal proteases that play critical roles in protein degradation and antigen processing. While they can be immunogenic and toxic when dysregulated, inhibiting cathepsins has been explored as a therapeutic strategy to mitigate tumor progression, demonstrating encouraging antitumor effects (Yadati et al. 2020).

Thus, it can be concluded that the in silico predictions based on the hybridization of cecropin and cathepsin sequences were successful in designing new peptides reducing the toxicity associated with cecropins and the immunogenicity of cathepsins.

#### 3.2.5 Hemolytic Activity and Half‐Life

The data on the predicted hemolytic activity of the peptides (Table S5) show values ranging from 0.30 to 0.51. A score close to 0 indicates a lower likelihood of hemolytic activity, whereas values closer to 1 suggest a higher probability. Overall, the analyzed peptides exhibit low hemolytic activity, comparable to that of isolated cecropins and cathepsins. Although cecropins generally demonstrate low hemolytic activity, they may induce hemolysis at high concentrations (Sitaram and Nagaraj 1999). In contrast, cathepsins do not exhibit direct hemolytic activity; however, their uncontrolled release can cause tissue damage through proteolytic degradation (Erickson et al. 2013; Vidak et al. 2019).

AMPs have inherent instability due to their susceptibility to degradation by proteolytic enzymes, such as trypsin, which can significantly reduce their half‐life (Svenson et al. 2022). Although their half‐life is considerably shorter compared to conventional drugs, this characteristic presents a significant therapeutic advantage. The rapid degradation of AMPs by endogenous proteases limits the microorganisms' exposure time to the compounds, thereby reducing the likelihood of resistance development. This property makes AMPs promising agents in the fight against resistant infections, especially in a scenario where antimicrobial resistance emerges as one of the greatest challenges to global public health.

#### 3.2.6 Prediction of Pharmacokinetics Parameters

The SMILES codes for the sequences were generated to predict absorption, distribution, metabolism, excretion, and toxicity (ADMET) profile. Of the 192 hybrid peptide sequences studied, 3 sequences (CATL1.2_CECB2, CECB1_CATL1.2, and CATL1.2_CECB1_2) were selected based on key criteria. The selection focused on peptides with 16 amino acids or fewer, as shorter peptides typically offer better bioavailability and easier synthesis. We prioritized candidates with a C2 Pred score nearing 0.5 to ensure effective cellular uptake. Additionally, all selected peptides were classified as nonallergenic to guarantee safety for clinical use. The decision‐making process involved initial screening, CPP assessment, allergenicity check, and evaluation of physicochemical properties like hydrophobicity using the GRAVY score. This structured approach ensured that the final candidates were well‐suited for therapeutic applications and enhanced the transparency of our selection process. Table S6 shows the selected peptides for subsequent pharmacokinetic and structural analyses along with their evaluated properties.

ADMET (Absorption, Distribution, Metabolism, Excretion, and Toxicity) properties were predicted using the ADMETlab version 2.0 web server (Table S7). The parameters analyzed included blood–brain barrier penetration (BBB), Caco‐2 permeability, volume of distribution (VD), plasma protein binding (PPB), human intestinal absorption (HIA), clearance (CL), half‐life (T1/2), skin sensitization, AMES toxicity, and carcinogenicity. All compounds exhibited positive HIA, indicating a strong ability to cross the intestinal barrier. Higher BBB penetration is typically linked to greater lipophilicity and increased uptake; however, the calculated BBB values suggested a high likelihood of being negative.

PPB is a critical parameter in drug safety assessments, as compounds with high PPB (> 90%) often have a narrow therapeutic index, while those with low PPB are generally safer. All analyzed peptides demonstrated low PPB, indicating a favorable therapeutic index. Caco‐2 cells, derived from human colon adenocarcinoma, possess permeability characteristics similar to those of intestinal enterocytes and are used to predict in vivo drug intestinal absorption. All compounds achieved optimal scores (greater than −5.15) in the Caco‐2 permeability assays.

Regarding carcinogenicity, none of the analyzed peptides indicated a potential to cause cancer. The AMES test results confirmed that none of the peptides were genotoxic. Additionally, assessments of other toxicity parameters, such as hERG inhibition, hepatotoxicity, and skin sensitization, indicated that all peptides were harmless.

Peptide‐based molecules, defined by the US Food and Drug Administration (FDA) as polymers consisting of ≤ 40 amino acids with a molecular weight (MW) of 500–5000 Da, are gaining popularity in the development of new therapies (Jois 2022). While peptide‐derived drugs do not conform to the classic Lipinski's Rule of Five for designing orally bioavailable medications (which includes criteria such as a molecular weight under 500 Da, fewer than five hydrogen bond donors, fewer than 10 hydrogen bond acceptors, and a LogP value of less than 5, indicating lipophilicity) (Lipinski 2016), they present a unique opportunity for creating therapies that are more specific than small‐molecule drugs and more membrane‐permeable than biologics (Philippe et al. 2021). Key parameters such as MW, topological polar surface area (tPSA), LogP, Fsp3, number of rotatable bonds (NRB), hydrogen bond acceptors (HBAs), hydrogen bond donors (HBDs), and number of aromatic rings (NARs) were evaluated. FDA‐approved peptides had MWs as high as 1200 Da and LogP values ranging from 5 to 8, which contributed to improved oral bioavailability (Santos et al. 2016). However, these peptides also exhibited more H‐bond donors and acceptors than what is considered acceptable under Ro5 for small molecules. Additionally, elevated values of MW, tPSA, and NRB can restrict cellular permeability. The analyzed peptides displayed a range of values for NRB, Fsp3, LogP, and NAR, indicating variability in lipophilicity and molecular complexity.

Specific ADMET studies on cecropins and cathepsins reveal that, despite some inherent limitations of peptides, chemical modifications can substantially enhance their pharmacokinetic properties. For example, pegylation and peptide cyclization have been shown to improve stability and half‐life, decrease rates of enzymatic degradation, and enhance oral bioavailability (Kremsmayr et al. 2022; Kumar et al. 2020). Medicinal chemistry research on cecropins indicates that optimizing peptide sequences can minimize toxicity and improve antimicrobial selectivity. The quantity and positioning of cationic residues within the peptide sequence significantly influence bactericidal activity, with specific combinations, such as proline and arginine or tryptophan, arginine, and lysine, enhancing antimicrobial properties (Jiang et al. 2008). Furthermore, the design and optimization of antimicrobial peptides (AMPs) can produce peptides with improved selectivity and reduced cytotoxicity, achieved through structural modifications and amino acid substitutions (Fjell et al. 2009).

#### 3.2.7 Structure Prediction

In the analysis of the structural models, a remarkable conservation of alpha‐helix formations was observed, a typical characteristic of cecropins. This structural feature is essential for the biological function of cecropins, as the formation of amphipathic alpha‐helices enables their binding to and penetration of bacterial membranes, resulting in cell lysis (Hoskin and Ramamoorthy 2008; Park et al. 1998).

While cathepsins can exhibit variations between alpha and beta conformations (Turk et al. 2012), their structural flexibility including the capacity to switch between these conformations plays a crucial role in their function of degrading a diverse array of protein substrates within the lysosome (Bunk et al. 2021).

The analysis of the modeled peptide structures indicated that most of the predicted BAPs retained the alpha‐helix configuration, consistent with the characteristics of cecropins (Guo et al. 2023). This structural conservation suggests that the BAPs could possess similar antimicrobial potential, benefiting from the stability and membrane‐insertion capabilities typical of amphipathic alpha‐helices. The incorporation of cathepsin features may enhance the functional diversity of BAPs, allowing them to leverage structural robustness and stability across various environments. Consequently, combining the structural traits of cecropins and cathepsins could lead to peptides with optimized characteristics for specific biological functions. The 3D homology structure models predicted by PEP FOLD, Ramachandran validation plots, and helical wheel projections of the 3 peptides selected for this phase are available in Figures S3–S5.

### 3.3 Selection of Hybrid Peptides and Chemical Synthesis

Three peptides were carefully selected and synthesized (Table 1) through a comprehensive analysis that integrated previous predictive models (Tables S6 and S7). The selection criteria focused on several crucial parameters: the peptides were required to have between 14 and 16 amino acid residues, a molecular weight (MW) below 2040 Da, an ideal cell‐penetrating peptide index of approximately 0.5, and demonstrated antimicrobial activity values exceeding 0.8, as indicated by predictive tools. In silico analyses of these three peptides revealed their promising potential for cell penetration, along with significant antimicrobial, anticancer, and antiviral activities. Furthermore, these hybrid peptides were characterized by their cationic nature, solubility, stability, and nontoxicity, with hemolytic activity maintained below 5%. The synthesis of the selected hybrid peptides CATL1.2_CECB2 (YWLVKNSWGKIFKKIE), CATL1.2_CECB1_2 (WLVKNSWGKLGKKI) and CECB1_CATL1.2 (KIGKKIEYWLVKNSWG) was performed in solid‐phase following a (Fmoc)/(*t*‐Bu) procedure, to obtain C‐terminal amide peptides. After purification, peptides were obtained at > 99% HPLC purity and were characterized by High Resolution Mass Spectrometry (HRMS) (Table 1) and proton nuclear magnetic resonance (^1^H‐NMR) (Appendix S1).

**TABLE 1:** Sequences, retention times, HPLC purities, and mass spectrometry data of the synthesized peptides.

Peptide	Sequence	*t*_R_ (min)^a^	Purity (%)^b^	HRMS
CATL1.2 _CECB2	YWLVKNSWG KIFKKIE	5.57	> 99	1020.0836 [M+2H]^2+^
CATL1.2 _CECB1_2	WLVKNSWG KLGKKI	4.83	> 99	828.5069 [M+2H]^2+^
CECB1_ CATL1.2	KIGKKIE YWLVKNSWG	5.14	> 99	975.0611 [M+2H]^2+^

*Note:* Red for Cathepsin derived sequences and Blue for cecropin derived sequences.

^a^ HPLC retention time.

^b^ Percentage determined by HPLC at 220 nm after purification by column chromatography.

### 3.4 Circular Dichroism Analysis

**FIGURE 3:**
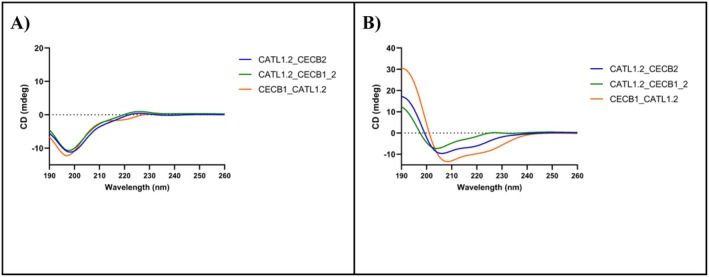
Circular dichroism spectra of peptides in the presence of 30 mM SDS (A) and HEPES Buffer (B).

The far‐UV CD spectra of the peptides is presented in Figure 3 where it reveals a random conformation in water (Figure 3A), indicated by a negative band at 200 nm. Upon the addition of 1 mM SDS micelles (serving as a model for anionic lipids) (Figure 3B), a conformational transition occurred, leading to the formation of an alpha‐helical structure. This transition was characterized by a pronounced maximum at 194 nm and two minima at 208 and 222 nm. These findings support the in silico predictions of the peptides' 2D structures and are consistent with previous observations, such as those by Turk et al. (2012), which demonstrated regions with random coil and alpha‐helix conformations in cathepsin structures. Furthermore, studies by Lee and colleagues (Lee et al. 2013) also demonstrated that peptides such as cecropin and its derivatives exhibit random conformation in water and adopt an alpha‐helical structure in the presence of SDS, as observed in CD spectra.

### 3.5 In Vitro Analysis

#### 3.5.1 Antimicrobial Assays

The antimicrobial activity of the three synthesized peptides (CATL1.2_CECB2, CECB1_CATL1.2, and CATL1.2_CEC1_2) was evaluated using the colony‐forming unit (CFU/mL) counting method. The results showed greater efficacy against the Gram‐negative bacterium *Escherichia coli* , with minimum inhibitory concentration (MIC) values ranging from 150.8 to 67.3 μM (Table 2). Among the tested peptides, CECB1_CATL1.2 is the most potent, exhibiting the lowest MIC and, consequently, the highest bacterial inhibition capacity.

**TABLE 2:** Minimum inhibitory concentration (MIC) of hybrid peptides against selected microorganisms.

MIC (μM)			
Microorganism	CATL1.2_CECB2	CATL1.2_CECB1_2	CECB1_CATL1.2
*E. coli*	71.39	150.81	67.31
*S. aureus*	287.09	> 307.72	269.32
*C. albicans*	142.78	> 307.72	> 269.32

When comparing these results with available literature data, it is observed that the MIC values obtained for the synthesized peptides are higher than those reported for well‐studied antimicrobial peptides, such as those derived from human cathepsin G. For example, the study by Bangalore et al. (1990) demonstrated that cathepsin G‐derived peptides exhibited MIC values against *E. coli* ranging from 0.25 to 0.5 μM. These peptides showed high efficacy due to their cationicity and hydrophobicity, which are essential for interaction with bacterial membranes. Similarly, the work of Shafer et al. (2002) highlighted structural modifications that enhanced the antimicrobial activity of cathepsin G‐derived peptides, achieving an expanded action spectrum and efficacy at very low micromolar concentrations (MICs below 1 μM for *E. coli* ).

Although the peptides synthesized in this study demonstrated significant antimicrobial activity against *E. coli* , the MIC values obtained (150.8 to 67.3 μM) suggest there is place for new structural optimizations that could improve their efficacy. Another relevant point is the specificity observed against gram‐negative bacteria, which indicates that the composition of the outer membrane of gram‐negative bacteria makes them more susceptible to the action of cationic peptides (Shafer et al. 2002). The ability of CECB1_CATL1.2 to stand out among the synthesized peptides reflects the impact of specific structural variations on antimicrobial efficacy. Comparisons with Cecropin peptides isolated from several species presented MIC values from 0.5 to 78 μg/mL (Gholizadeh and Moradi 2021).

Regarding the gram‐positive bacterium *S. aureus* , the peptides also demonstrated growth inhibition, although at higher concentrations when compared with antimicrobial studies on Cathepsins and cecropins (Bangalore et al. 1990; Gholizadeh and Moradi 2021). The MIC values for *S. aureus* were 287.09 μM for CATL1.2_CECB2, 269.32 μM for CECB1_CATL1.2, and 307.72 μM for CATL1.2_CEC1_2. The higher MIC values for gram‐positive bacteria are expected due to their more complex cell wall, which requires higher concentrations for effective inhibition (Rohde 2019). Despite these elevated values, the observed antimicrobial activity suggests potential for further optimization of these peptides.

Additionally, the peptide CATL1.2_CECB2 was the only one to show inhibition against the fungus *C. albicans* , with an MIC of 142.78 μM. In contrast, CECB1_CATL1.2 and CATL1.2_CEC1_2 exhibited significantly higher MIC values of 269.32 μM and 307.72 μM, respectively. Antimicrobial peptides, particularly those derived from cecropins, often show reduced activity against fungi, requiring higher concentrations for antifungal effects (Wang et al. 2015). The efficacy of CATL1.2_CECB2 against *C. albicans* suggests the affinity of this peptide for fungal membranes, which could be explored through structural modifications.

Among the three peptides, CATL1.2_CECB2 stands out for its broad‐spectrum activity, effectively inhibiting *E. coli* , *S. aureus* , and *C. albicans* . All peptides share similar amino acid compositions, with subtle differences that significantly impact their antimicrobial activities. For example, CATL1.2_CEC1_2, which differs from CATL1.2_CECB2 only in the first and last amino acids, exhibited better antimicrobial performance against *E. coli* , emphasizing the importance of amino acid positioning.

Research by Abraham et al. (2014) emphasizes the correlation between the molecular structure of peptides and their antimicrobial efficacy, demonstrating that specific changes in amino acid sequences can directly influence antimicrobial activity. Additionally, studies by Oñate‐Garzón et al. (2017) suggest that increasing positively charged residues in cecropins can improve their interaction with bacterial membranes, potentially enhancing their efficacy against various pathogens. These findings indicate that, although the peptides analyzed show significant antimicrobial activity, future structural modifications could further improve their efficacy and therapeutic potential.

**FIGURE 4:**
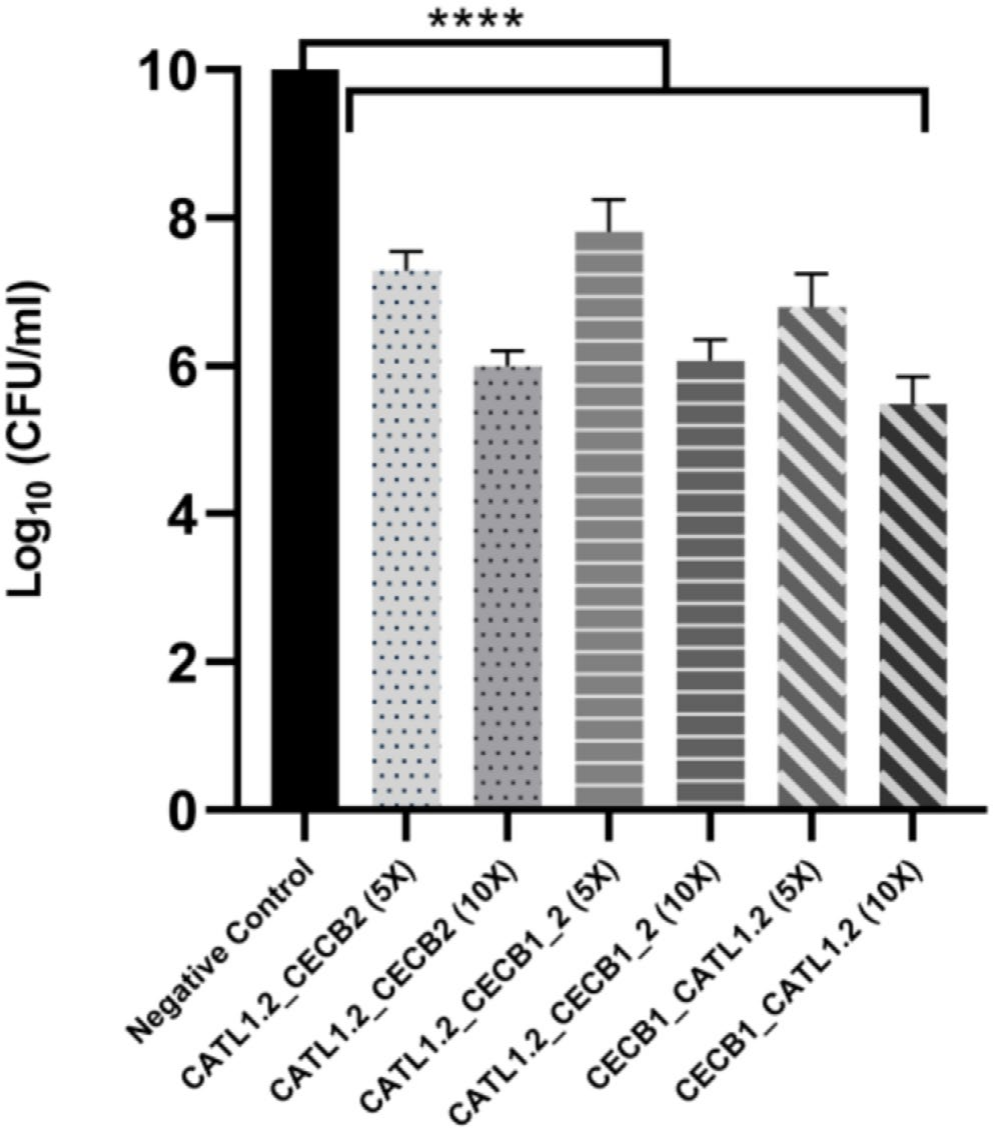
*E. coli* biofilm inhibition. Inhibition was measured by the mean and standard deviation of the cell count (CFU/mL, log10) of the microorganisms at 5× MIC and 10× MIC. The negative control group represents untreated bacteria. Statistical significance was calculated by one‐way analysis of variance (ANOVA), followed by Tukey's multiple comparisons test (****p < 0.0001).

Biofilm inhibition assays were performed with the peptides that exhibited significant efficacy and lowest MIC against planktonic microorganisms. Biofilm inhibition assays performed on previously established cultures of *E. coli* demonstrated significant activity of the tested antimicrobial peptides at both concentrations, showing marked reductions in biofilm dimension compared to the untreated control (Figure 4).

Kavanaugh et al. (2021) emphasized the efficacy of cathepsins in antibiofilm activity, attributed to their ability to degrade crucial structural proteins in the biofilm's extracellular matrix. Cecropins are also known for their antibiofilm properties (Lee et al. 2023), although they typically exhibit high MIC and undergo proteolytic degradation before effectively targeting the biofilm (Kalsy et al. 2020).

All designed hybrid peptides evaluated displayed inhibitory effects against *E. coli* biofilm, indicating that the advantageous properties of both cathepsins and cecropins were preserved in these hybrids. Although additional studies are required to clarify the detailed mechanisms of action, initial findings suggest that hybrid peptides can successfully integrate the beneficial characteristics of their parent peptides, offering a promising approach for inhibiting biofilm formation.

#### 3.5.2 Cytotoxicity and Hemolytic Assay

**FIGURE 5:**
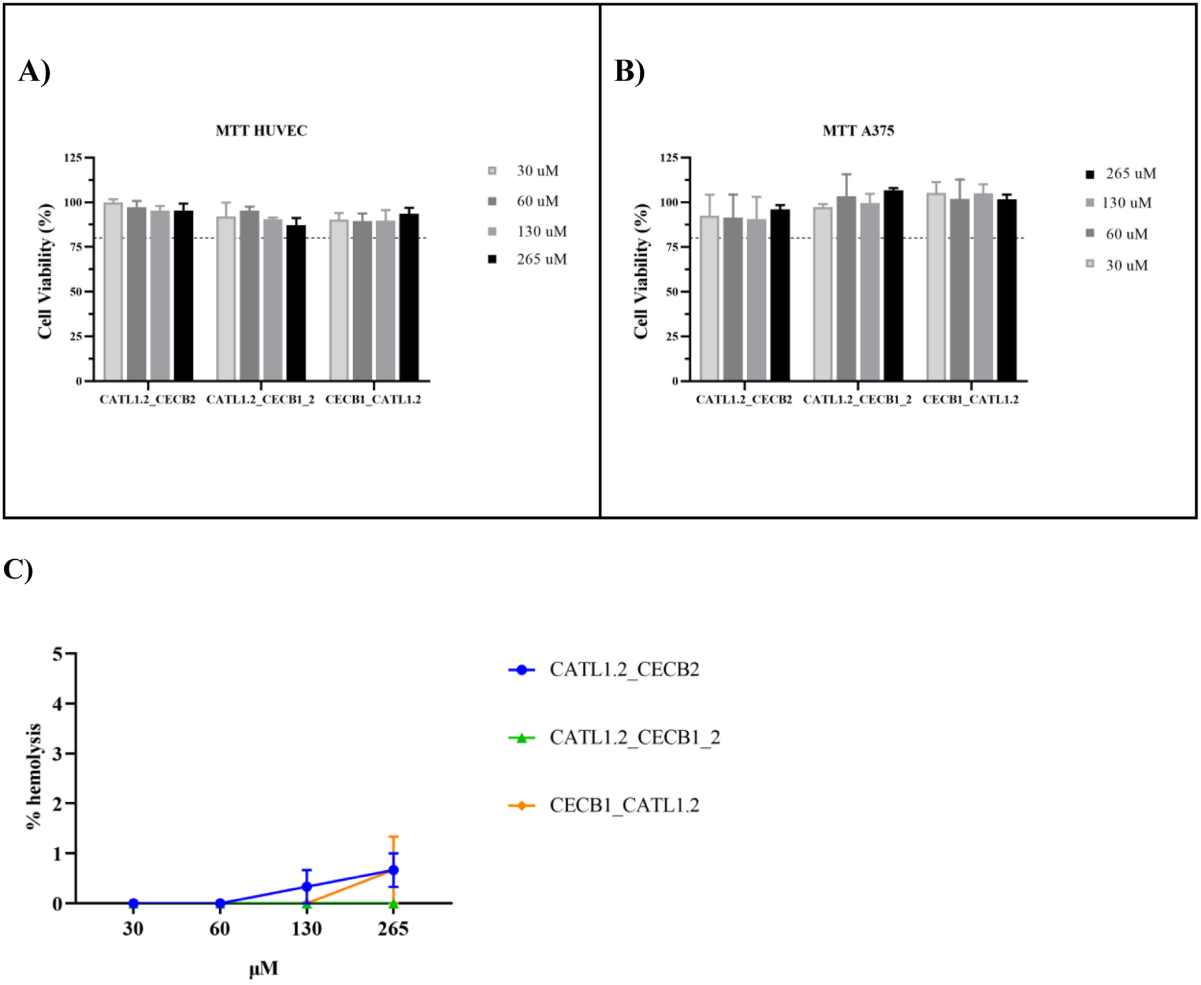
Cytotoxicity of hybrid peptides on cells. (A) Cell viability of human umbilical cord vein endothelial cells (HUVEC); (B) Cell viability of human melanoma cell line (A375) Cell viability was normalized against a control in which no stimuli were added (i.e., only DMEM culture medium +10% FBS). (C) Hemolytic test. The experiments were performed in triplicate and the data were expressed as the mean ± SD. The statistical analysis was performed by one‐way ANOVA and Tukey's test at *p* < 0.05 level.

Figure 5 presents the results of the cytotoxicity assays. Using the cell viability test by evaluation of metabolic activity (Vieira‐da‐Silva and Castanho 2023), the designed peptides showed low toxicity to HUVEC and A375 cells (Figure 5a,b). At all tested concentrations (up to 250 μM), cell viability remained above 85%. These data are consistent with the findings of this study, which, after in silico analyses, suggest that the hybridization of cecropin and cathepsin sequences effectively reduced the toxicity associated with cecropin (Tamang and Saier Jr. 2006).

The results are particularly encouraging, especially for HUVEC, as they suggest that the developed peptides have a high safety margin, which is essential for future studies. The low toxicity observed can be attributed to the careful design of the peptides, which considered critical parameters such as charge, length, and hydrophobic moments—key factors in minimizing undesirable side effects (Khabbaz et al. 2021). Furthermore, the sustained high levels of cell viability support the therapeutic potential of the peptides, especially in the context of broad‐spectrum antimicrobial therapies (Hancock and Sahl 2006; Xiao et al. 2025). However, considering the in silico results, the data for A375 did not meet expectations for tumor cell lines. This may be related to specific characteristics of this cell line, such as intrinsic resistance mechanisms, differences in receptor or key protein expression, and particularities of the tumor microenvironment. Nonetheless, subsequent studies should be conducted on other tumor lines with distinct molecular profiles to enhance the understanding of the action mechanism of the analyzed peptides and identify potential targets or more favorable contexts for their therapeutic efficacy.

**FIGURE 6:**
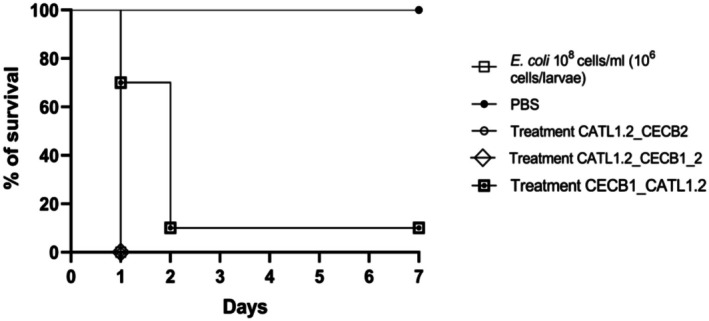
Survival curve in *G. mellonella*. Treatment of *E. coli* with the peptides CATL1.2_CECB2 (71.4 μM), CATL1.2_CECB1_2 (150.8 μM) and CECB1_CATL1.2 (67.3 μM) *p < 0.0001. Statistical significance was calculated using the Gehan‐Breslow‐Wilcoxon test *p* < 0.0001.

To assess hemolytic activity, the peptides were incubated with erythrocytes for 1 h at 37°C. The peptides did not show significant hemolytic activity when compared to the control (Figure 6). For antimicrobial peptides to be viable for systemic applications, it is crucial that they exhibit low toxicity to erythrocytes (Greco et al. 2020). The absence of hemolytic activity in the tested peptides is advantageous, as many AMPs are limited in their use due to their significant hemolytic properties (Fathi et al. 2024). For instance, Brady and colleagues (Brady et al. 2019) described hemolytic activity of several Cecropins and Cecropins like, and particularly AeaeCec 5 peptide from *Aedes aegypti* presented hemolytic activity at 12.5 μM.

### 3.6 In Vivo Analysis

#### 3.6.1 Toxicity Assays and Therapeutic Studies on *Galleria mellonella*

The toxicity of the peptides was also evaluated using the alternative *G. mellonella* model. At all tested concentrations, 100% larval survival was observed, indicating that the peptides did not exhibit significant toxicity in this model. This system is widely used for the preliminary evaluation of new compounds due to its correlation with mammalian models and its experimental simplicity (Desbois and Coote 2012).

However, it is important to note that while *G. mellonella* provides a useful preliminary assessment, extrapolating these results to mammals should be done with caution. Previous studies, including the work of Wu and Hancock (1999), emphasized the potential systemic toxicity of cationic peptides like cecropins at high concentrations, particularly concerning hemolysis and damage to mammalian cells. Therefore, although the results obtained in *G. mellonella* are promising and consistent with the cell viability data observed in assays with HUVEC and A375 cells, further validation steps are required. Future studies should include toxicity evaluations in mammalian models to confirm the safety of hybrid peptides for systemic applications. Additionally, structural modifications of the peptides, particularly those hybridized with cathepsins, could be explored to reduce toxicity without compromising their antimicrobial efficacy.

The in vivo assay using *G. mellonella* larvae involving *E. coli* infection and treatment was performed to evaluate the therapeutic potential of CATL1.2_CECB2, CATL1.2_CECB1_2 and CECB1_CATL1.2 peptides (Figure 6). The CECB1_CATL1.2 peptide was the only one that showed therapeutic potential, demonstrating a 70% increase in the survival rate of infected larvae 24h post‐treatment, along with a 10% increase in the survival rate from the second day, maintaining this level until the end of the experimental period.

**FIGURE 7:**
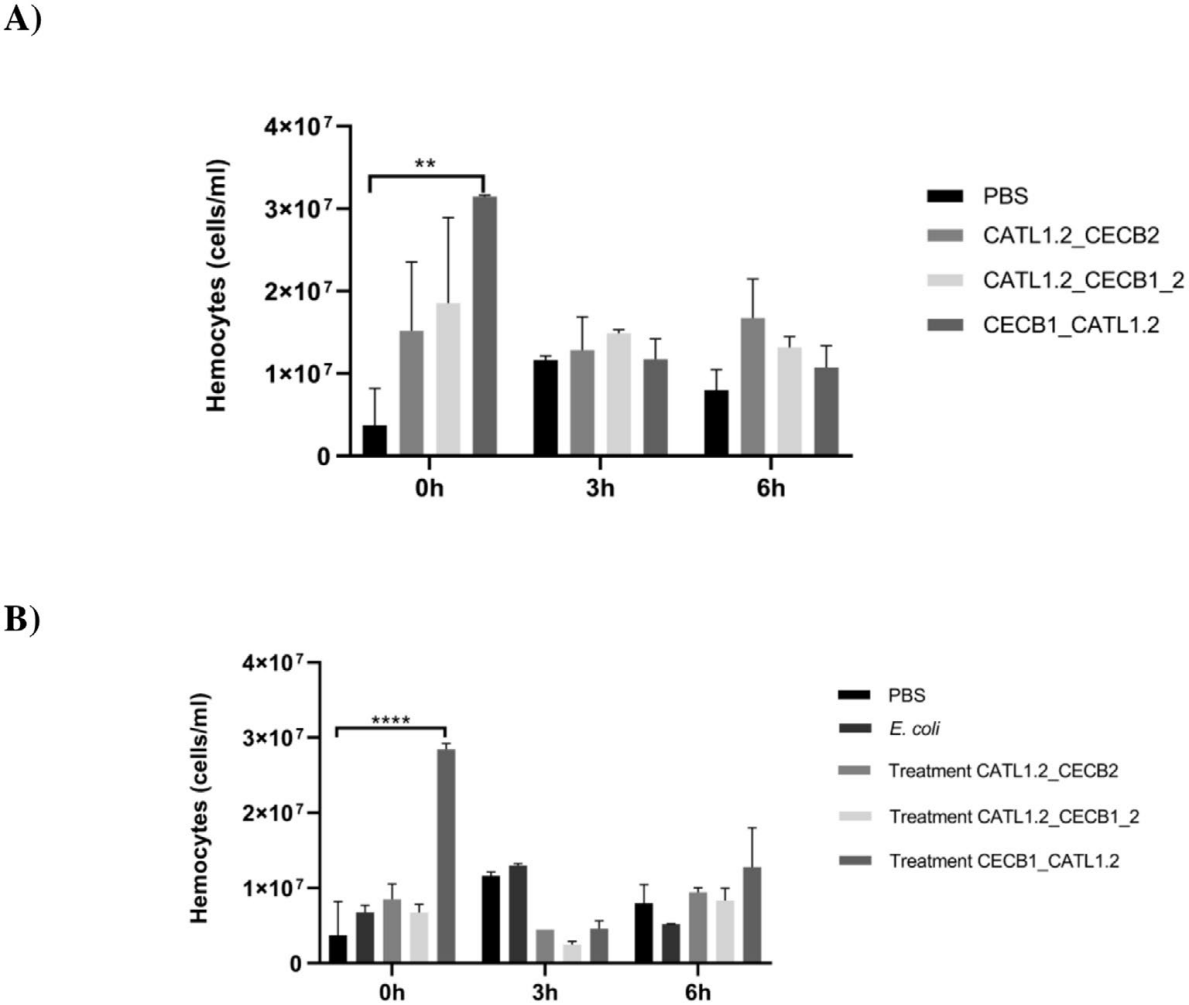
(A) Comparison of hemocyte density between control groups (PBS, CATL1.2_CECB2, CATL1.2_CECB1_2, and CECB1_CATL1.2); (B) infection and treatment with CATL1.2_CECB2 (71.4 μM), CATL1.2_CECB1_2 (150.8 μM), and CECB1_CATL1.2 (67.3 μM) at different times. Statistical significance was calculated by applying one‐way analysis of variance (ANOVA) followed by Tukey's multiple comparison test, (**p = 0.0022), (****p < 0.0001).

Analyzing the regulation of hemocyte counts in *G. mellonella* larvae under different time conditions, it was observed that the CECB1_CATL1.2 peptide significantly increased the number of hemocytes in larvae at 0 h, an effect that did not persist in subsequent evaluations (3 h and 6 h). This finding indicates that CECB1_CATL1.2 can induce an initial immune response in larvae, with temporary activation of hemocytes, compared to the control group (PBS), as observed in Figure 7A. In assays with *E. coli* , the infected group showed an increase in hemocyte counts at 3 h compared to the initial count (0 h), followed by a decrease at 6 h, possibly due to the progress of the infection and depletion of hemocytes. In the groups treated with the peptides, the response to CECB1_CATL1.2 treatment was more effective, with a peak in hemocytes at 0 h, and a decrease in the 3 h and 6 h assessments (Figure 7B). These data indicate that CECB1_CATL1.2 can potentially contribute to an initial immune response and help control the infection in its early stages.

Studies conducted by Vergis et al. (2021) demonstrated that the CAMA peptide, a hybrid of cecropin and melittin, has significant immunomodulatory effects on *G. mellonella* larvae. The analysis revealed that this peptide, in addition to immunomodulation, reduced bacterial counts, presented low toxicity and was not toxic to larval cells, confirming its therapeutic potential. These findings, along with the results obtained with the CECB1_CATL1.2 peptide, which combines cecropin and cathepsin, highlight the usefulness of the *G. mellonella* model for the study of cecropin‐based and hybrid immunomodulation and underscore the potential of these combinations in improving the immune response.

### 3.7 Molecular Interactions and Target Prediction

#### 3.7.1 Target Prospection and Docking Results

The PharmMapper target search yielded 300 overall targets for each peptide, but only the 50 highest normalized scores for each were analyzed. The collected data were categorized by function, indication, protein classification, and source/origin, and only potential receptors whose microorganism of origin was *E. coli* were selected. The best potential receptors for the peptides are listed in Table S8.

The most promising receptors identified by this method were Thymidylate synthase (PDB_ID_: 1AOB) for the peptide CATL1.2_CECB1_2, Xanthosine phosphorylase (PDB_ID_: 1YR3) for the peptide CECB1_CATL1.2, and DNA gyrase B for both peptides. The prediction of the receptor binding site to the ligands was performed for all selected proteins, resulting in the identification of five possible binding sites for each protein. The result of each site presents the coordinates of the centers of the grid box in x, y, and z in which the subsequent docking was performed. The data obtained on the coordinates are represented in Table 3.

**TABLE 3:** Representation of the grid box centers in x, y, and z coordinates, referring to the five prospected binding sites for each of the target proteins. PDB ID (Identification code in the Protein Database): 1AOB (Thymidylate synthase), 1EI1 (DNA gyrase B), 1YR3 (Xanthosine phosphorylase), 1USQ (Dr hemagglutinin), 1WEI (A/G‐specific DNA glycosylase), and 1A99 (Periplasmic putrescine‐binding protein).

	Targets PBD ID					
	1AOB	1EI1	1YR3	1USQ	1WEI	1A99
Binding sites	−30, −6, 18	23, 33, 17	35, −1, 44	−6, 20, −3	20, 4, 19	168, 28, 64
	−23, −6, 9	39, 21, 30	40, 42, 35	91, 15, 32	17, 17, 10	177, 74, 78
	−24, 5, 5	31, 23, 6	36, 30, 3	121, 21, 36	8, −17, 20	179, 7, 78
	−25, 13, 33	11, 18, 11	47, 43, 16	98, 62, 37	22, −8, 18	148, 68, 34
	−40, 8, 8	19, 24, 34	47, −11, 30	20, 61, −1	13, −2, 2	170, 51, 38

The docking results show binding affinity values between −23 and −33 kJ/mol. The receptor classification results presented by AutoDock Vina were different from PharmMapper for all peptides. Although PharmMapper did not identified *E. coli* targets for the peptide CATL1.2_CECB2, docking analyses included the interaction of this peptide with the DNA gyrase B protein (referred as ## in Table 4), as it was identified as a potential target for the other peptides. Among the proteins studied, those that presented the highest binding affinity energy were DNA gyrase subunit B (PDB_ID_: 1EI1), for all peptides, Thymidylate synthase, (PDB_ID_: 1AOB), for peptide CATL1.2_CECB1_2 and Adenine A/G‐specific glycosylase, (PDB_ID_: 1WEI), for peptide CECB1_CATL1.2. All identified binding sites were tested, however, in this work only the sites with the best binding affinities are represented in Table 4.

**TABLE 4:** Docking results by AutoDock Vina. Peptides were used as ligands, and targets obtained by PharmMapper were used as receptors. PM Rank (Overall rank by PharmMapper), PDB ID (Protein Data Bank identification code).

PM Rank	PDB ID	Receptor name	Binding site	Affinity energy (kJ/mol)
*CATL1.2_CECB2*				
##	1EI1	DNA gyrase B	23, 33, 17	−33.5
*CATL1.2_CECB1_2*				
1	1AOB	Thymidylate synthase	−23, −6, 9	−32.6
13	1EI1	DNA gyrase B	23, 33, 17	−33.4
*CECB1_CATL1.2*				
1	1YR3	Xanthosine phosphorylase	36, 30, 3	−23.4
16	1USQ	Dr hemagglutinin	98, 62, 37	−26.3
21	1EI1	DNA gyrase B	19, 24, 34	−27.6
23	1WEI	A/G‐specific DNA glycosylase	17, 17, 10	−27.2
48	1A99	Periplasmic putrescine‐binding protein	170, 51, 38	−26.8

**FIGURE 8:**
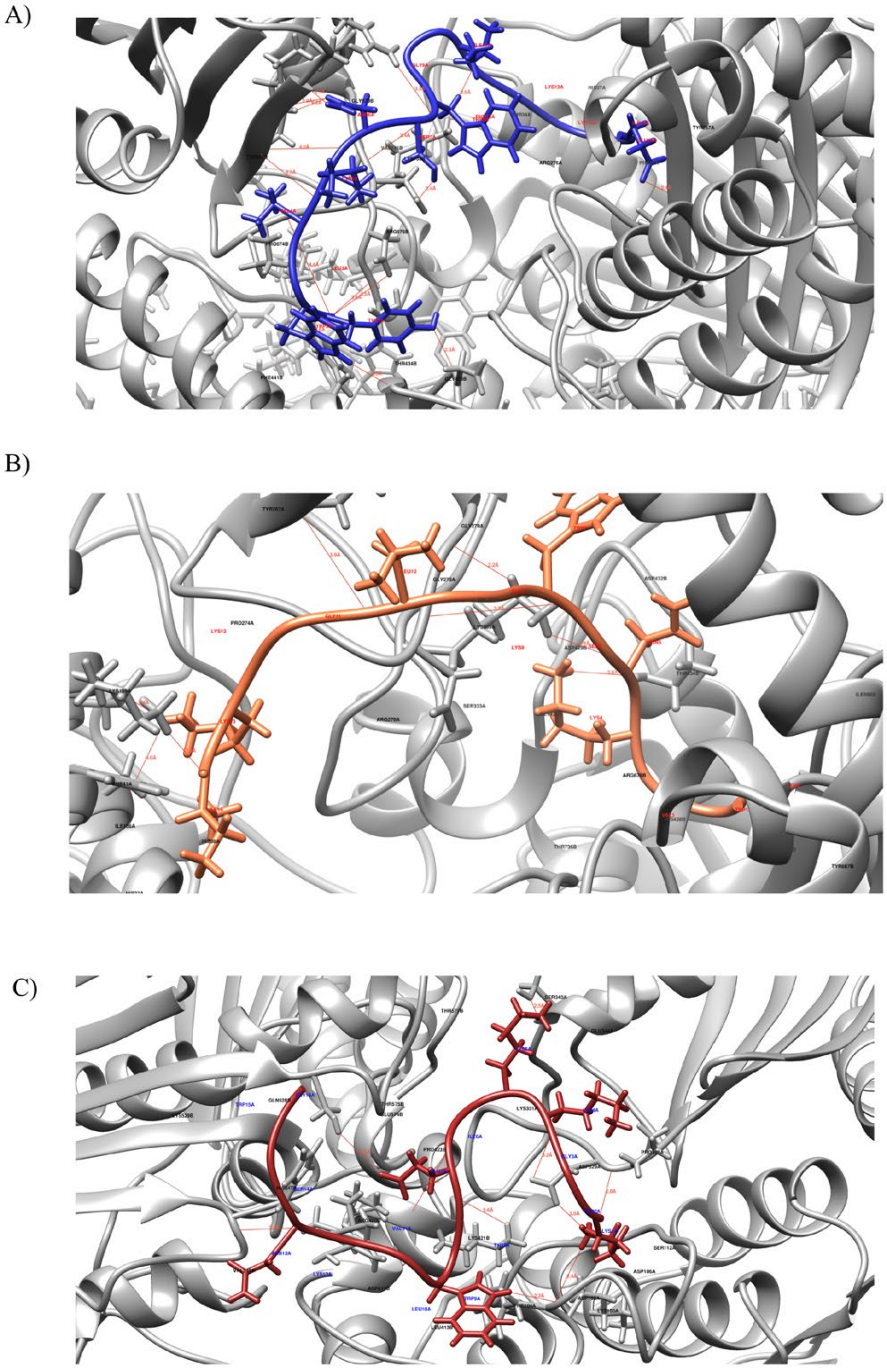
Molecular docking between the peptides and DNA gyrase. (A) Interaction between the CATL1.2_CECB2 peptide (blue) and DNA gyrase (gray), with thirteen hydrogen bonds identified between the peptide and the receptor. (B) Interaction between the CATL1.2_CECB1_2 peptide (orange) and DNA gyrase (gray), with eight hydrogen bonds identified between the peptide and the receptor. (C) Interaction between the CECB1_CATL1.2 peptide (red) and DNA gyrase (gray), with nine hydrogen bonds identified between the peptide and the receptor.

The molecular docking performed with AutoDock Vina demonstrated relevant interactions between the peptides and target proteins. For a better understanding of the docking analysis, only the strongest hydrogen bonds (≤ 4Å) between the peptides and receptors were considered. All the peptides showed better interactions with DNA gyrase compared to the other proteins studied. The CATL1.2_CECB2 peptide formed 13 hydrogen bonds with DNA gyrase (Figure 8A), while CATL1.2_CECB1_2 formed 8 hydrogen bonds (Figure 8B), and CECB1_CATL1.2 formed 9 hydrogen bonds (Figure 8C). The hydrogen bonds with the highest docking scores are summarized in Table 5, highlighting the role of the peptide as both electron acceptor and donor in these interactions.

**TABLE 5:** Donor (electron donating residue), Acceptor (electron acceptor residue), D‐A distance (distance between heavy atoms of acceptor and electron donor), GLY 1.L N (residue name/number/chain ID/shared electron atom), L (Ligand ID), and Rx (Receptor Subunit Identification).

PM Rank	PDB ID	Receptor name	Donor	Acceptor	D‐A distance (Å)
*CATL1.2_CECB2*					
##	1EI1	DNA gyrase B	HIS 438.RB ND1	TYR 1.L O	2.616
			ASP 429.RB N	TYR 1.L OH	2.316
			ARG 686.RB NH2	SER 7.L O	3.252
			CYS 668.RB N	LYS 5.L O	4.018
			LYS 739.RB NZ	ILE 11.L O	2.601
			ILE 666.RB N	ASN 6.L OD1	2.783
			CYS 268.RA N	GLU 16.L OE1	2.427
			TYR 1.L H1	THR 736.RB O	3.574
			TYR 1.L H3	ASP 738.RB OD2	3.516
			TRP 2.L HE1	GLY 433.RB O	3.762
			ASN 6.A HD22	GLU 664.RB O	2.682
			SER 7.L HG	ASP 667.RB OD2	2.405
			TRP 8.L H	ASP 667.RB O	3.364
*CATL1.2_CECB1_2*					
13	1EI1	DNA gyrase B	LYS 189.RA NZ	ILE 14.L O	3.296
			CYS 268.RA N	LEU 10.L O	3.908
			GLY 279.RA N	TRP 7.L O	2.186
			LYS 339.RA NZ	ASN 5.L O	2.381
			LYS 4.L HZ3	THR 434.RB OG1	3.838
			TRP 7.L H	ASP 227.RA O	3.220
			LYS 13.L HZ1	PHE 41.RA O	4.028
			LYS 13.L HZ1	ASP 45.RA O	3.397
*CECB1_CATL1.2*					
21	1EI1	DNA gyrase B	LYS 421.RB NZ	GLU 7.L O	3.587
			SER 572.RB OG	GLU 7.L OE1	4.025
			LYS 1.L H2	PRO 328.RA O	2.771
			LYS 1.L HZ3	LYS 103.RA O	2.378
			LYS 1.L HZ2	ASP 329.RA OD2	3.046
			LYS 4.L H	ASP 329.RA OD1	3.174
			LYS 5.L HZ2	SER 343.RA OG	2.493
			TRP 9.L HE1	PHE 104.RA O	2.225
			ASN 13.L H	HIS 547.RB O	3.444

Comparison between the binding sites predicted from PDBsum and those observed using Chimera 1.6 software (Pettersen et al. 2004) revealed that the interacting peptides can be colocalized or close to known active sites of the target proteins.

DNA gyrase (PDB ID: 1EI1), a type II topoisomerase, plays a crucial role in maintaining DNA topology by introducing transient double‐strand breaks that allow the passage of a second segment of DNA before sealing it again (Spencer and Panda 2023). DNA gyrase B (PDB ID: 1EI1) is composed of a dimer of GyrB, has a TOPRIM domain that is involved in the linking and cleavage of DNA, and a domain which exhibits ATPase activity (Stracy et al. 2019). Antibiotic novobiocin is known to exert its mechanism of action on GyrB by competing with the ATP binding site (Gly_519_, His_516_, Lys_737_, Val_520_, Asn_446_, Asp_473_, Gly_502_, Lys_503_, Gly_517_, Val_518_, and Leu_515_) in the ATPase (Chauhan et al. 2024). Fluoroquinolones, in turn, interact with GyrA, stabilizing the enzyme‐DNA cleavage complex, and inhibiting DNA replication (Alfonso et al. 2022). However, the efficacy of both antibiotics is compromised by microbial resistance, especially due to activation of the AcrAB‐TolC efflux pump (Kuo et al. 2024). In this context, all tested peptides showed interaction with DNA gyrase, suggesting their potential as alternative inhibitors to combat resistant strains of *E. coli* . Specifically, the CATL1.2_CECB2 peptide, although it does not compete directly for the novobiocin site, is located in the same binding pocket, interacting with critical residues of GyrB, such as Thr_736B_, Lys_739B_, and Asp_738B_, close to the ATP binding site. This interaction can interfere with the enzyme's conformation or ATP binding dynamics, contributing to the inhibition of catalytic activity. Furthermore, the CATL1.2_CECB1_2 and CECB1_CATL1.2 peptides interacted with the GyrA subunit, indicating a potential to inhibit DNA replication and transcription in a manner similar to the effect of fluoroquinolones. In particular, the CECB1_CATL1.2 peptide demonstrated significant interaction with the Lys_103A_ residue, a key residue located close to the cleavage site, as evidenced by the PDBsum database. This interaction may be associated with the stabilization of the peptide‐enzyme complex, possibly interfering with DNA binding or the formation of the cleavage complex, which reinforces the potential of these peptides as innovative models for antimicrobial agents.

The interaction between hybrid peptides of cecropin and cathepsin with the targeted enzymes suggests a new therapeutic potential in combating resistant strains of *E. coli* . Previous studies indicate that the synergy between antimicrobial peptides and antibiotics can be explored to enhance the effectiveness of antibiotics, promoting effects such as increased membrane permeability, rupture of biofilms, direct increase in antimicrobial activity, and inhibition of resistance mechanisms (Taheri‐Araghi 2024). By targeting multiple sites within bacterial cells, this approach creates a more complex adaptive barrier, making the development of resistance more challenging. Therefore, the combination of antimicrobial peptides with antibiotics represents a promising strategy to optimize or prolong the efficacy of existing drugs in the treatment of resistant microorganisms (Morroni et al. 2021).

## 4 Conclusion

This study successfully designed hybrid bioactive peptides by combining sequences from cecropins and cathepsins, using their distinct properties for therapeutic applications. Utilizing bioinformatics tools, we investigated the physicochemical characteristics and bioactivities of these peptides, identifying promising candidates with potential antimicrobial, anticancer, and antiviral activities. The hybrid peptides demonstrated significant antimicrobial efficacy, particularly against gram‐negative bacteria, while exhibiting low cytotoxicity to human cells. Importantly, the integration of cecropin's antimicrobial properties with cathepsin's structural stability resulted in peptides that are not only effective against various pathogens but also have reduced toxicity. In vitro and in vivo analyses indicated that the selected hybrid peptides maintain their functionality, with promising results in enhancing immune responses and inhibiting biofilm formation. Molecular docking studies further revealed their interactions with critical bacterial targets, suggesting potential as innovative antimicrobial agents. Overall, this research highlights the potential of hybrid peptides as multifunctional therapeutics, opening roads for novel strategies to combat antibiotic resistance and emerging infectious diseases while minimizing adverse effects. Future studies should focus further on optimizing these peptides for improved efficacy and exploring their mechanisms of action in greater detail.

## Conflicts of Interest

The authors declare no conflicts of interest.

## Supporting information


**Appendix S1:** cbdd70193‐sup‐0001‐AppendixS1.docx.

## Data Availability

The data that support the findings of this study are available from the corresponding author upon reasonable request.
