# Supplementary material for: Design of Hybrid Bioactive Peptides Derived From Cecropin and Cathepsin for Therapeutic Application
Source: Chem Biol Drug Des. 2025 Nov 7;106(5):e70193. doi: 10.1111/cbdd.70193 (PMC12594624; doi:10.1111/cbdd.70193)
Supplement: Supplementary file 1 — Appendix S1: cbdd70193‐sup‐0001‐AppendixS1.docx. [file CBDD-106-e70193-s001.docx]

***Supplementary information***

**Design of Hybrid Bioactive Peptides Derived From Cecropin And Cathepsin For Therapeutic Application**

**Gabriele Santos Cepinho¹^$^, Bruna Vitória Scavassa¹^$^, Gabrielle L. de Cena¹, Vitor Martins de Andrade¹, Luís Roberto F. Lima^2^, André Zelanis^2^, Montserrat Heras^3^,  Miguel ARB Castanho^4^, Katia Conceição¹***

¹Laboratory of Peptide Biochemistry and ^2^Functional Proteomics - Federal University of São Paulo – UNIFESP. Rua Talim, 330, São José dos Campos, Brazil; ^3^Institute of Food Agricultural Technology (INTEA), Campus Montilivi, 17003 University of Girona, Spain; ^4^Instituto de Medicina Molecular, Faculdade de Medicina da Universidade de Lisboa, Av. Professor Egas Moniz, 1649-028 Lisboa, Portugal.

**^$^ Authors contributed equally to this work**

*Corresponding author: Professor Katia Conceição, Universidade Federal de São Paulo – UNIFESP, Rua Talim, 330, 12231-280. São José dos Campos, Brasil. E-mail: katia.conceicao@unifesp.br. Telephone: (+55) 11 3385.4135 (R. 9724).

Figure S1

**Identification of conserved sequences between cecropins and cathepsins**

| **(A)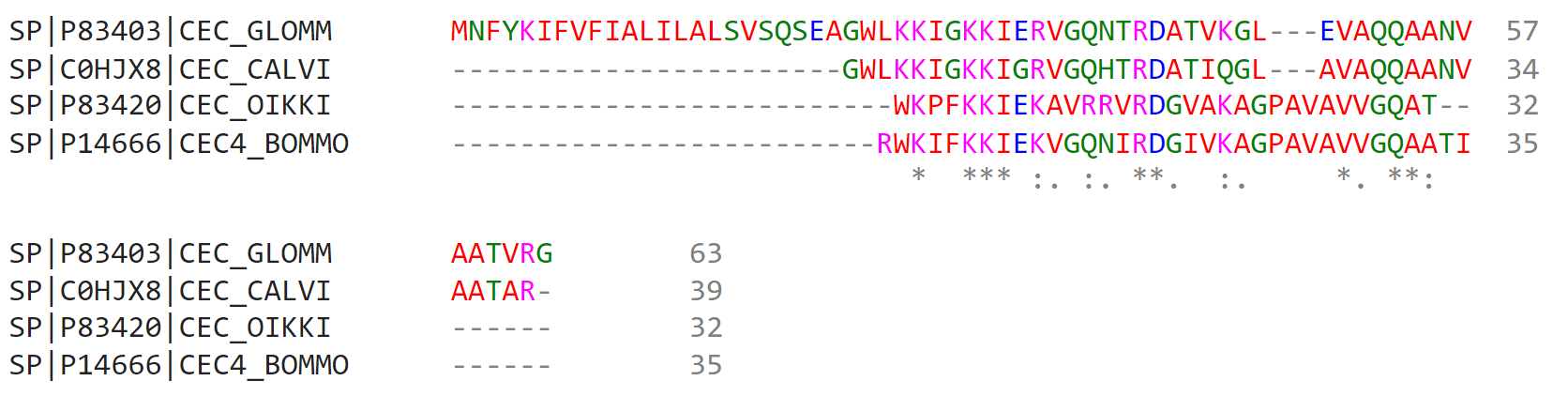** |
| --- |
| **(B)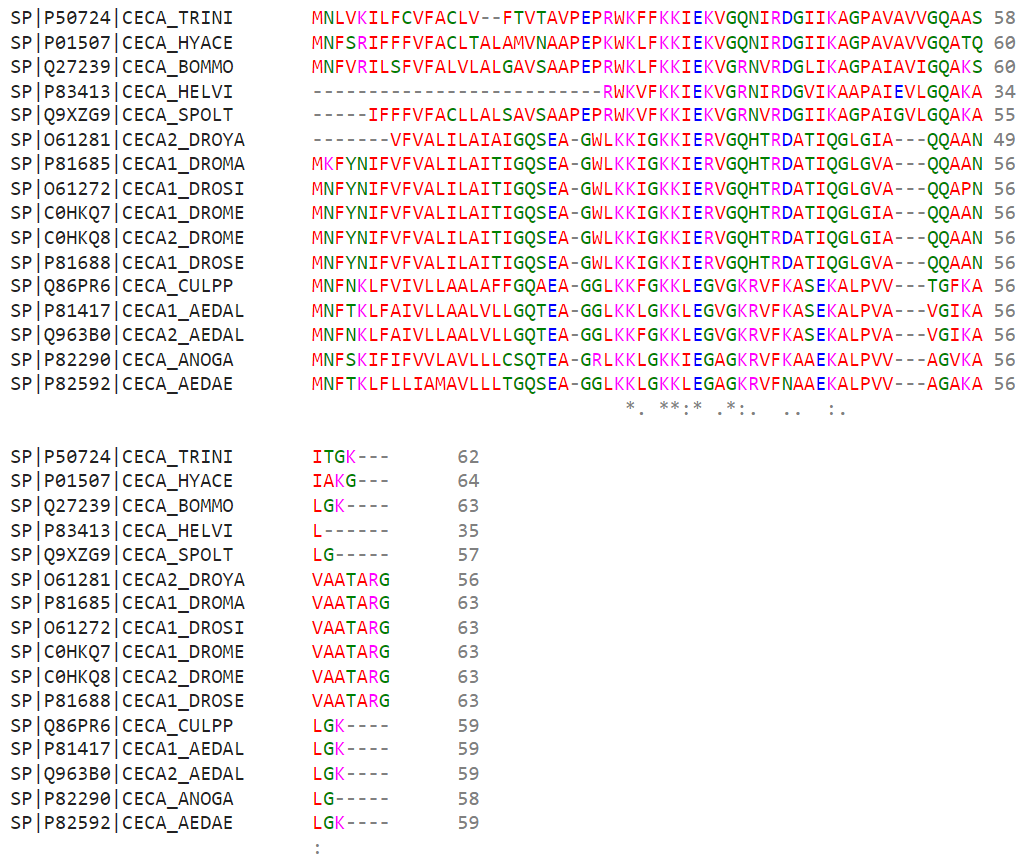** |
| **(C)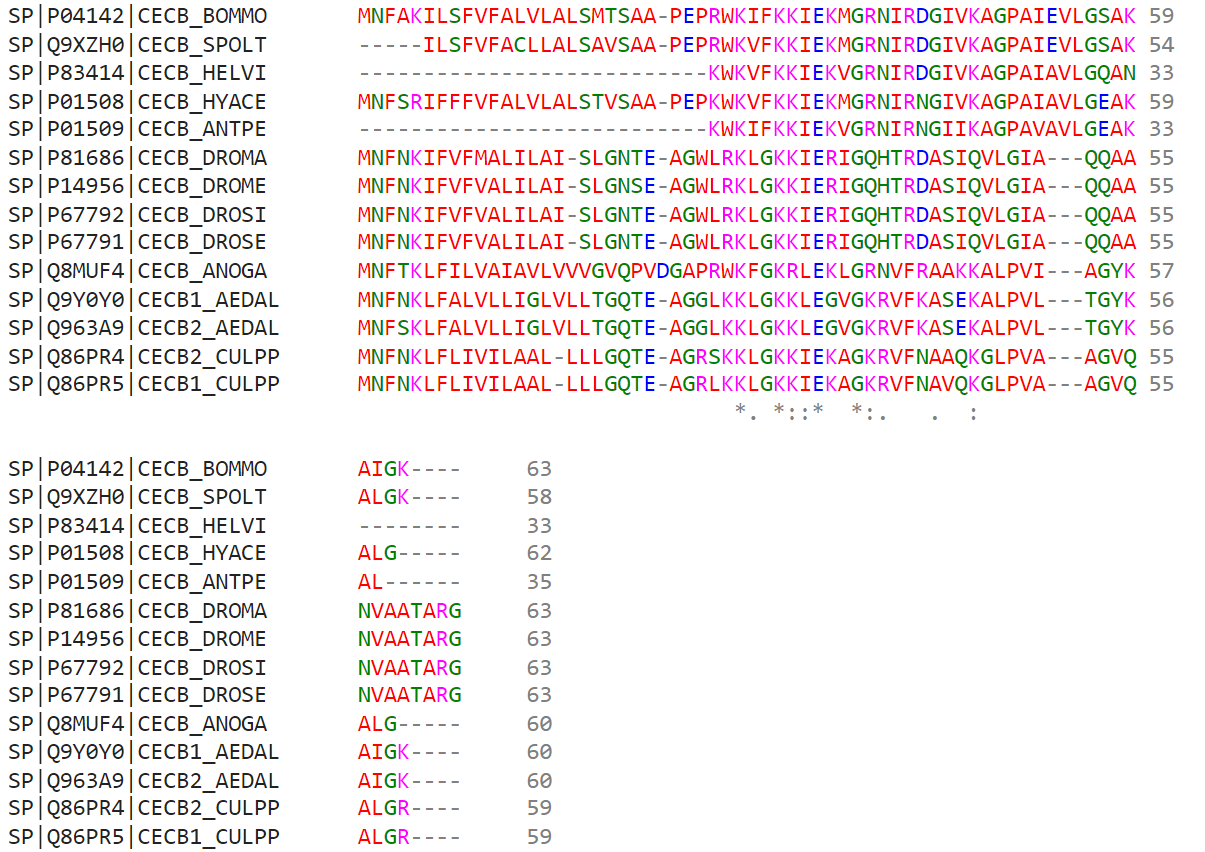** |
| **(D)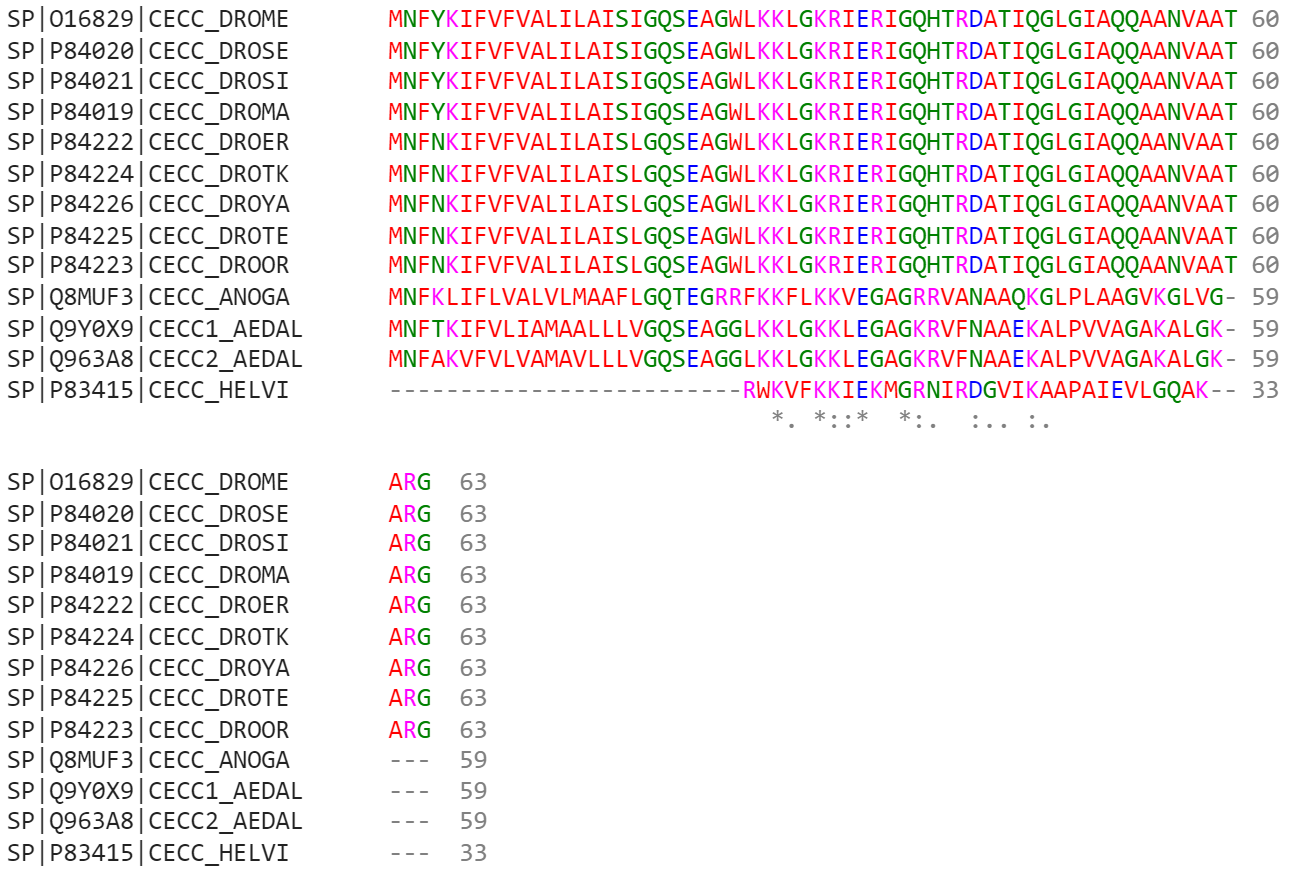** |
| **(E)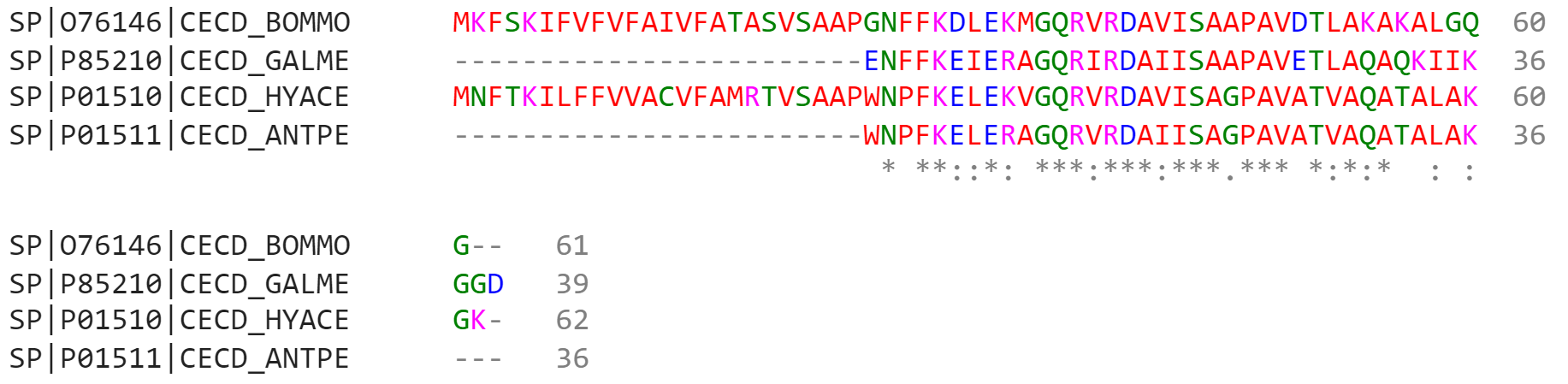** |

**Figure S1.** Multiple alignment of Cecropins by Clustal Omega (Clustal W). **(A**) CEC_GLOMM (Glossina  [morsitans morsitans), CEC_CALVI(](https://www.uniprot.org/taxonomy/7373)Calliphora [vicina) , CEC_OIKKI (Oike](https://www.uniprot.org/taxonomy/201386)ticus [kirby) and CEC4_BOMMO (Bombyx mori), (](https://www.uniprot.org/taxonomy/7091)**B)** CECA_TRINI ([Trichoplusia ni), CECA_HYACE (Hyalophora](https://www.uniprot.org/taxonomy/7111) cecropia[), CECA_BOMMO (](https://www.uniprot.org/taxonomy/7123)Bombyx [mori), CECA_HELVI (](https://www.uniprot.org/taxonomy/7091)Heliothis [virescens), CECA_SPOLT (Spodoptera](https://www.uniprot.org/taxonomy/7102) litura), [CECA2_DROYA (Drosophila yakuba), CECA1_DROMA (Drosophila](https://www.uniprot.org/taxonomy/69820)  mauritiana), CECA1_DROSI (Drosophila  [simulans), CECA1_DROME (Drosophila melanogaster), CECA2_DROME](https://www.uniprot.org/taxonomy/7245)(Drosophila [melanogaster), CECA1_DROSE (](https://www.uniprot.org/taxonomy/7227)Drosophila [sechellia), CECA_CULPP (](https://www.uniprot.org/taxonomy/7238)[Culex](https://www.uniprot.org/taxonomy/38569)  [pipiens](https://www.uniprot.org/taxonomy/7238) [pipiens](https://www.uniprot.org/taxonomy/38569)), CECA1_AEDAL (Aedes albopictus), CECA2_AEDAL ([Aedes albopictus](https://www.uniprot.org/taxonomy/7160)), CECA_ANOGA ([Anopheles gambiae](https://www.uniprot.org/taxonomy/7165)) and CECA_AEDAE ([Aedes aegypti](https://www.uniprot.org/taxonomy/7159) ), (**C)** CECB_ BOMMO ([Bombyx mori), CECB_SPOLT (](https://www.uniprot.org/taxonomy/7091)Spodoptera [litura), CECB_HELVI (Heliothis](https://www.uniprot.org/taxonomy/69820) virescens), CECB_HYACE ([Hyalophora cecropia), CECB_ANTPE (](https://www.uniprot.org/taxonomy/7102)Antheraea [pernyi), CECB_DROMA (Drosophila](https://www.uniprot.org/taxonomy/7123)  mauritiana), [CECB_DROME (Drosophila](https://www.uniprot.org/taxonomy/7119) melanogaster[),](https://www.uniprot.org/taxonomy/7227) CECB_DROSI ([Drosophila simulans), CECB_DROSE (](https://www.uniprot.org/taxonomy/7240)Drosophila [sechellia), CECB_ANOGA (](https://www.uniprot.org/taxonomy/7238)[Anopheles gambiae](https://www.uniprot.org/taxonomy/7165)), CECB1_AEDAL (Aedes albopictus), CECB2_AEDAL ([Aedes albopictus](https://www.uniprot.org/taxonomy/7160) ), CECB2_CULPP ([Culex](https://www.uniprot.org/taxonomy/38569)  pipiens [pipiens](https://www.uniprot.org/taxonomy/38569)) and CECB1_CULPP ([Culex](https://www.uniprot.org/taxonomy/38569)  pipiens [pipiens](https://www.uniprot.org/taxonomy/38569)), (**D)** CECC_DROME (Drosophila  [melanogaster), CECC_DROSE (Drosophila sechellia), CECC_DROSI (Drosophila](https://www.uniprot.org/taxonomy/7227)  simulans), CECC_DROMA (Drosophila  [mauritiana), CECC_DROER](https://www.uniprot.org/taxonomy/7238) [(](https://www.uniprot.org/taxonomy/7240)Drosophila [erecta), CECC_DROTK (Drosophila](https://www.uniprot.org/taxonomy/7220)  takahashii), [CECC_DROYA (Drosophila yakuba), CECC_DROTE (Drosophila](https://www.uniprot.org/taxonomy/29030)  teissieri), CECC_DROOR ([Drosophila orena](https://www.uniprot.org/taxonomy/7245)), CECC_ANOGA ([Anopheles gambiae](https://www.uniprot.org/taxonomy/7165)[), CECC1_AEDAL (](https://www.uniprot.org/taxonomy/7243)[Aedes albopictus](https://www.uniprot.org/taxonomy/7160)), CECC2_AEDAL ( [Aedes albopictus](https://www.uniprot.org/taxonomy/7160)) and CECC_HELVI (Heliothis [virescens) and (E) CECD_BOMMO (Bombyx](https://www.uniprot.org/taxonomy/7102) mori), CECD_GALME ([Galleria mellonella](https://www.uniprot.org/taxonomy/7137)[), CECD_HYACE (](https://www.uniprot.org/taxonomy/7091)Hyalophora cecropia [) and CECD_ANTPE (](https://www.uniprot.org/taxonomy/7123)Antheraea [pernyi](https://www.uniprot.org/taxonomy/7119)); The meaning of colors is describedin (Sievers et al. (2011), Mol Sys Bio 7:539). (A) Cecropines, (B) Cecropines A, (C) Cecropines B, (D) Cecropines C and (E) Cecropines D.

| **(A)**  **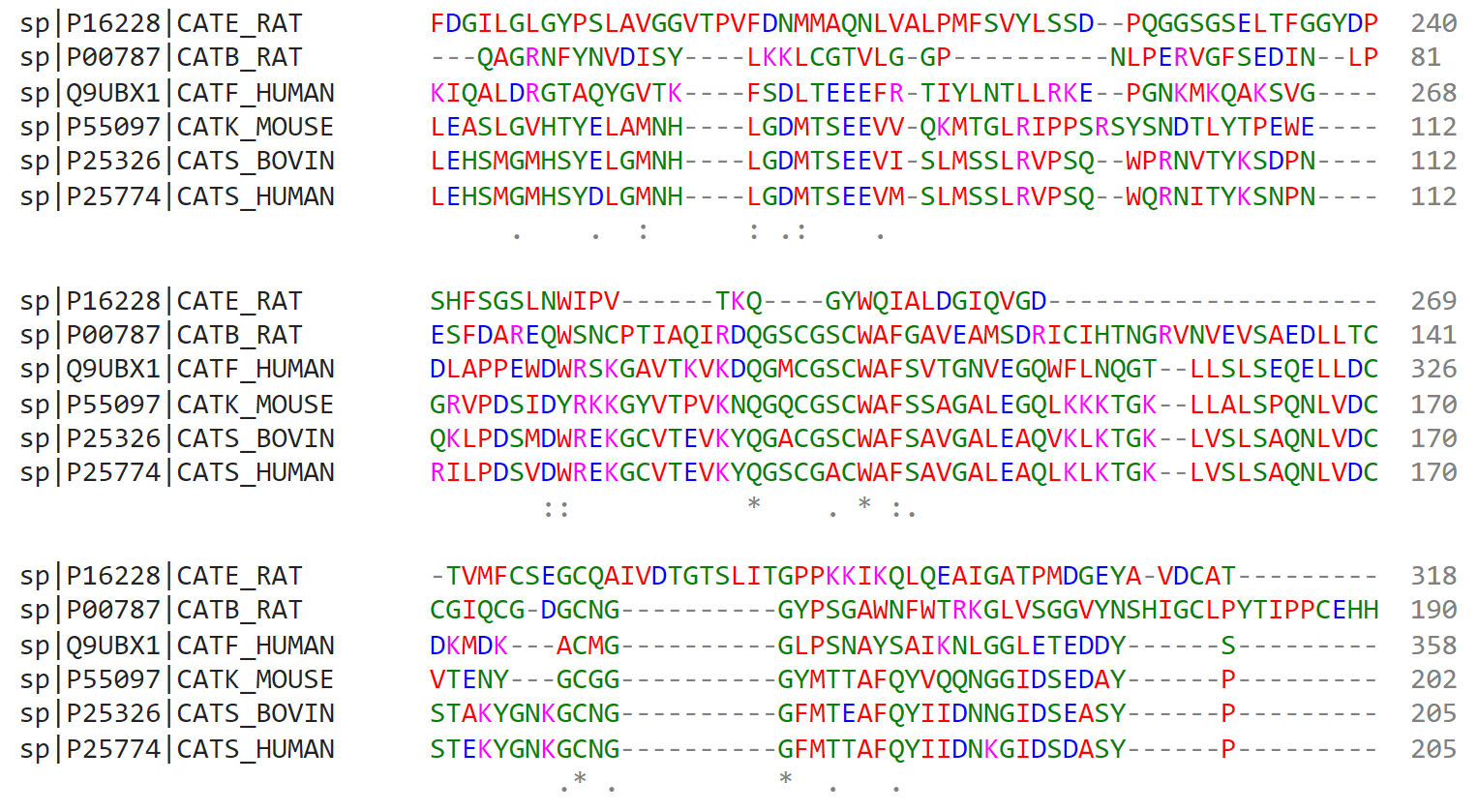**  **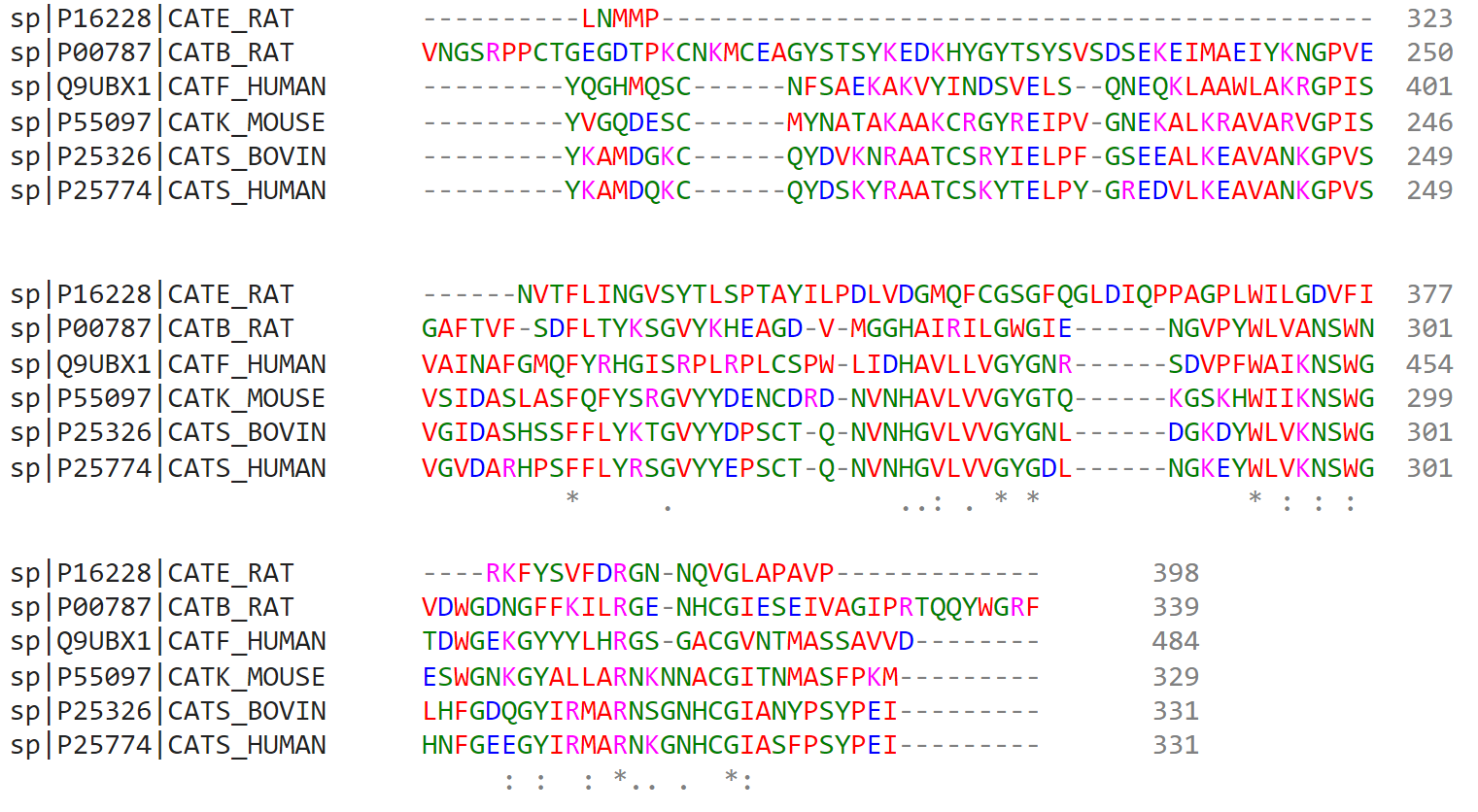** |
| --- |
| **(B)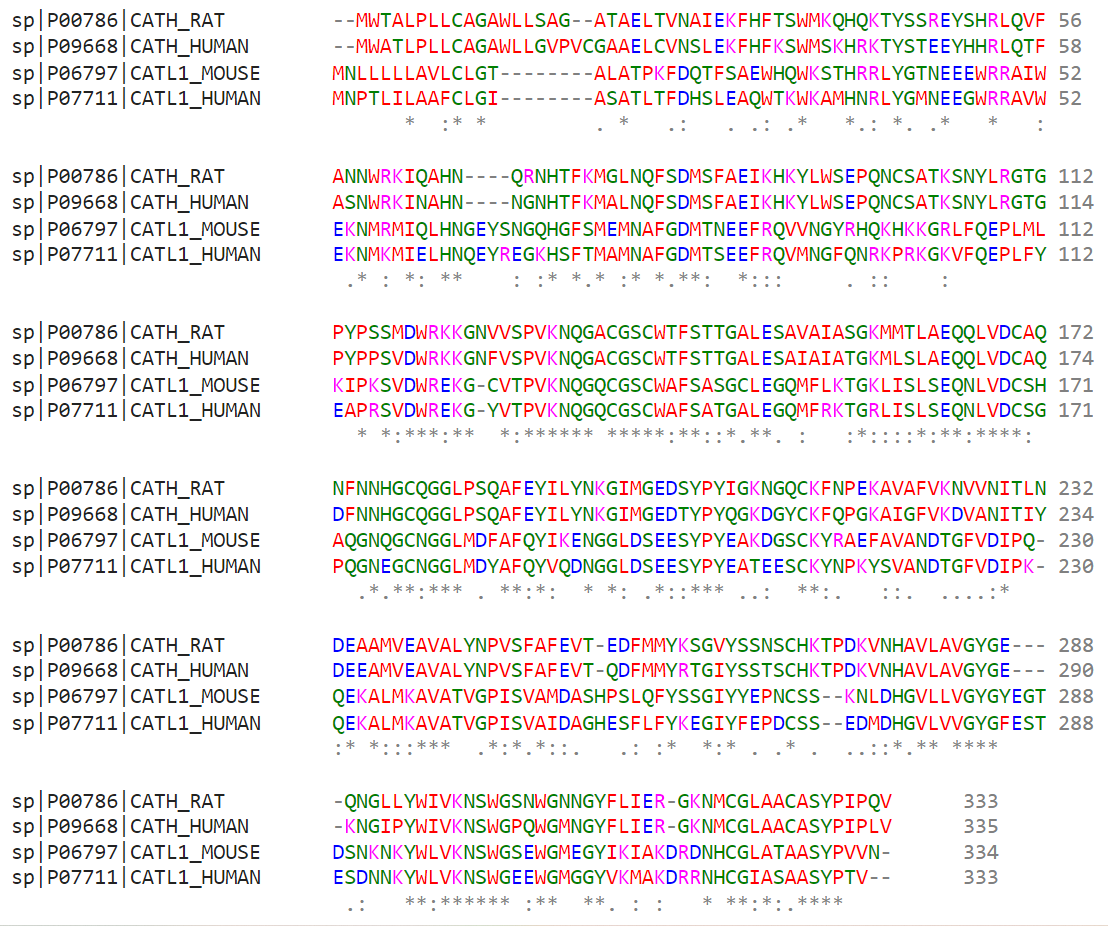** |

**Figure S2.** Multiple alignment of Cathepsins by Clustal Omega (Clustal W). **(A**) CATE_RAT (Rattus norvegicus), CATB_RAT (Rattus norvegicus), CATF_HUMAN (Homo sapiens), CATK_MOUSE ([Mus musculus](https://www.uniprot.org/taxonomy/10090)[), CATS_BOVIN (](https://www.uniprot.org/taxonomy/10116)[Bos taurus](https://www.uniprot.org/taxonomy/9913)) and [CATS_HUMAN (Homo](https://www.uniprot.org/taxonomy/10116) [sapiens](https://www.uniprot.org/taxonomy/9606)[) and (](https://www.uniprot.org/taxonomy/10116)**B)** CATH_RAT (Rattus norvegicus ), CATH_HUMAN [(](https://www.uniprot.org/taxonomy/10116)[Homo sapiens](https://www.uniprot.org/taxonomy/9606)), CATL1_MOUSE ([Mus musculus](https://www.uniprot.org/taxonomy/10090)) and CATL1_HUMAN ([Homo sapiens](https://www.uniprot.org/taxonomy/9606)); The meaning of colors is described in (Sievers et al. (2011), Mol Sys Bio 7:539). (A) Cathepsins and (B) Pro-Cathepsins


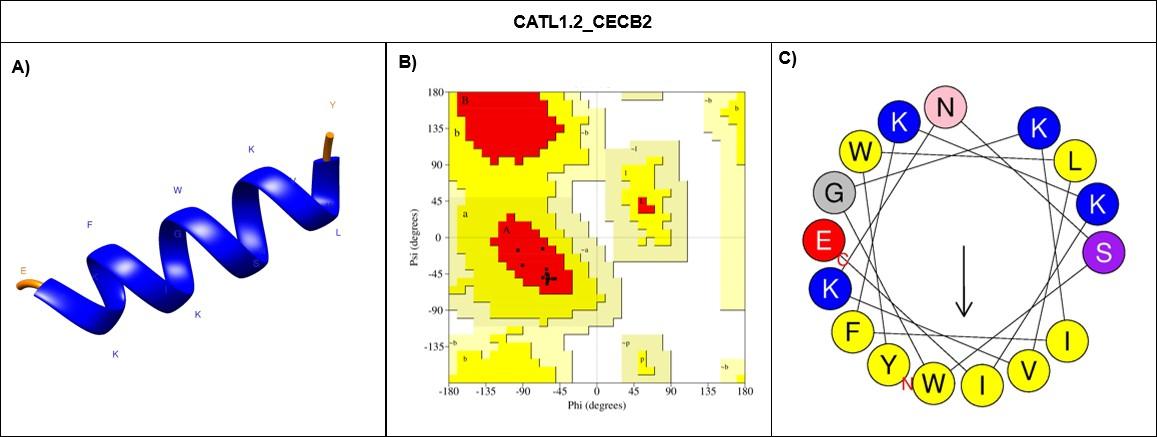


**Figure S3**. (A) 3D structure, (B) Ramachandran plot, (C) Helical wheel projection. Arrows indicate the direction of the hydrophobic moment (μH)


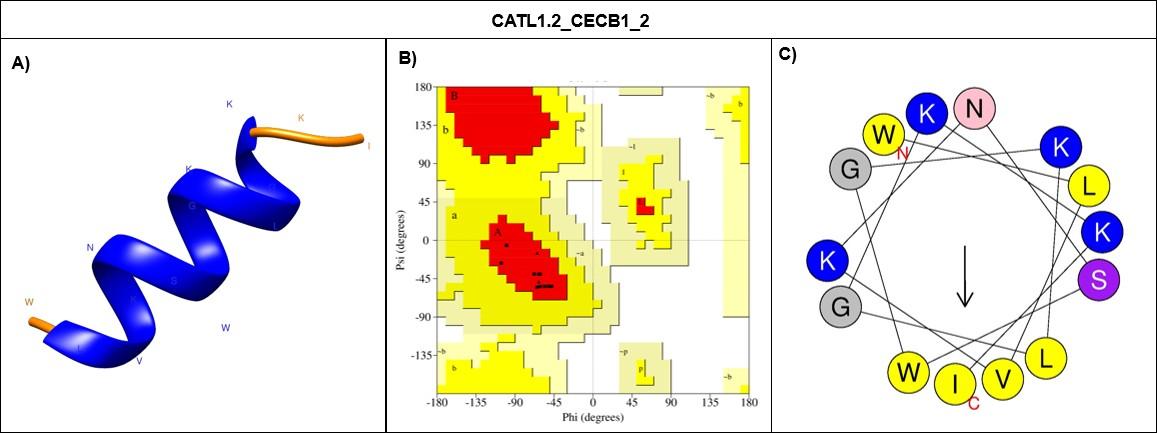


**Figure S4.** (A) 3D structure, (B) Ramachandran plot, (C) Helical wheel projection. Arrows indicate the direction of the hydrophobic moment (μH)


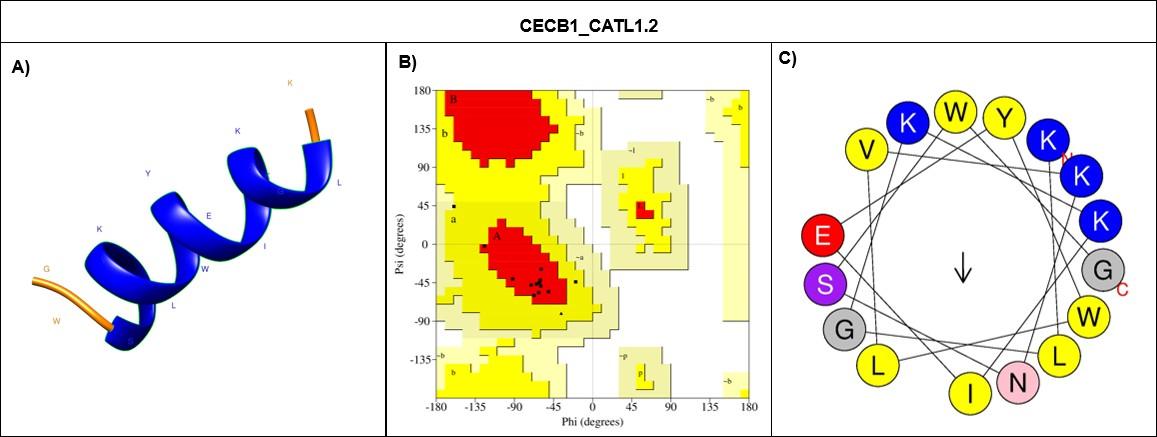


**Figure S5.** (A) 3D structure, (B) Ramachandran plot, (C) Helical wheel projection. Arrows indicate the direction of the hydrophobic moment (μH)

**Material and Methods**

**M1. Synthesis of hybrid peptides derived from Cecropin and Cathepsin**

**M1.2. General remarks**

All commercially available chemicals were used as purchased without further purification. Reverse-phase column chromatography purification of hybrid peptides was performed on a CombiFlash Rf200 automated flash chromatography system using a RediSep Rf Gold reversed-phase column packed with high-performance C18 derivatized silica (Teledyne ISCO, Lincoln, NE, USA). Hybrid peptides were analyzed under standard analytical HPLC conditions with a 1260 Infinity II liquid chromatography instrument (Agilent, Santa Clara, CA, USA), using a Kromasil 100 C_18_ (4.6 mm × 40 mm, 3 µm) column with a 2-100% B linear gradient over 17 min at a flow rate of 1 mL min^-1^. Solvent A was 0.1% aqueous TFA, and solvent B was 0.1% TFA in CH_3_CN. Detection was carried out at 220 nm. ESI-HRMS analyses of hybrid peptides were performed with a compact mass spectrometer from Bruker Daltonics (Serveis Tècnics de Recerca, University of Girona, STR-UdG). The instrument is equipped with an electrospray ionization (ESI) source with a hybrid quadrupole-time-of-flight (QTOF) analyser. The instrument was operated in the positive ESI(+) ion mode, in a range of 50 to 3000 *m/z*. Samples were dissolved and they were introduced (1-5 μL) to the spectrometer through an Agilent Technologies 1200 Series HPLC automatic injector at a flow rate of 0.1 mL/min. Nitrogen was employed as drying and nebulising gas. Results were analysed with Bruker Compass DataAnalysis 6.1 software. NMR experiments were performed in STR-UdG with an Ultrashield ASCEND Nanobay 400 instrument (9.4 T) from Bruker (^1^H-NMR, 400 MHz). Structural assignments were made with additional information from COSY experiments. NMR spectra were processed and analyzed using TopSpin 3.6.4. Chemical shifts were reported as δ (parts per million, ppm) directly calibrated with the solvent signal. All ^1^H-NMR spectra were recorded in CD_3_OD and referenced to residual CH_3_OH at 3.31 ppm. Coupling constants (*J*) are given in Herst (Hz). The following abbreviations were used for spin multiplicity: d = doblet, t = triplet, m = multiplet, dd = doublet of doublets.

**M1.3. Solid Phase Synthesis of hybrid peptides. General Procedure**

Hybrid peptides CATL1.2_CECB2 (YWLVKNSWGKIFKKIE), CATL1.2_CECB1_2 (WLVKNSWGKLGKKI) and CECB1_CATL1.2 (KIGKKIEYWLVKNSWG) were synthesized manually by the solid-phase method following a 9-fluorenylmethoxycarbonyl (Fmoc)/*tert-butyl* (*t*-Bu) protocol, in polypropylene syringes fitted with a polyethylene porous disk. Solvents and soluble reagents were removed in vacuum. Commercially available reagents were used throughout without purification. A Fmoc-Rink-MBHA resin (0.71 mmol/g) was used for the synthesis of CATL1.2_CECB1_2; and an aminomethyl ChemMatrix resin (0.62 mmol/g) for the synthesis of CATL1.2_CECB2 and CECB1_CATL1.2 to obtain C-terminal peptides amides. Coupling of Fmoc-Rink (4 equiv) onto the aminomethyl ChemMatrix resin was mediated by DIC (4 equiv) and Oxyma (4 equiv) in DMF at room temperatura overnight. Fmoc group removal was achieved with piperidine-DMF (3:7, 1 x 2 min + 2 x 10 min). Coupling of commercial Fmoc-amino acids (4 or 3 equiv) were performed using DIC (4 or 3 equiv) and Oxyma (4 or 3 equiv) in DMF under stirring at room temperature for 4 or 8 h, and monitoring by the Kaiser test. For each coupling and deprotection step, the resin was washed with DMF (6 x 1 min) and CH_2_Cl_2_ (3 x 1 min) and aired-dried. After coupling of ninth amino acid residue, NMP was used instead DMF. Peptide elongation was performed by repeated cycles of Fmoc removal, coupling and washings. Once the synthesis was completed, peptidyl resins were subjected to the N-terminal Fmoc removal. Then, the hybrid peptides were cleaved by treatment with TFA-H_2_O-Pheol-TIS (88:5:5:2) for 2 h. Following TFA evaporation, peptides were precipitated by adding cold diethyl ether and collected by centrifugation. Finally, the crude peptides were purified by reverse-phase column chromatography, lyophilized, analyzed by HPLC, and characterized by high resolution mass spectrometry (HRMS) and proton nuclear magnetic resonance (^1^H-NMR)

**M1.4. Kaiser test procedure**

The Kaiser or nynhidrin test is a colorimetric and qualitative test that allows the detection of free primary amines. It is used to monitor the coupling of an amino acid to a peptidyl resin. To perform the test, a portion of resin is placed in an Eppendorf and 2-3 drops of the solutions A (2.5 g ninhydrin / 50 mL ethanol), B (0.2 g phenol/ 50 mL ethanol) and C (NaCN 1 mM in pyridine), are added consecutively. The suspension is heated for 3 min at 100 °C and the color of the resin is checked. If the coupling is not complete and free amino groups remain, the solution acquires a blue color (positive test); otherwise, the color is yellow (negative test).

**M1.5. Synthesis of CATL1.2_CECB2**

Following the general procedure described above, CATL1.2_CECB2 (YWLVKNSWGKIFKKIE) was obtained in >99% purity HPLC (λ=220 nm): *t*_R_ 5.57. ESI-HRMS (*m/z*) calculated for C_101_H_152_N_24_O_21_, M+nH; [M+H]^+^ 2039.1642 found 2039.1555, [M+2H]^2+^ 1020.0869 found 1020.0836, [M+3H]^3+^ 680.3937 found 680.3935. ^1^H-NMR (400 MHz, CD_3_OD) δ (ppm) 7.59 (d, *J*=7.9 Hz, 1H, *H*_indol_-Trp), 7.51 (d, *J*=7.9 Hz, 1H, *H*_indol_-Trp), 7.32 (t, *J*=8.1 Hz, 2H, 2x*H*_indol_-Trp), 7.23-7.06 (m, 11H, 4x *H*_indol_-Trp, 5x *H*_arom_-Phe, 2xC*H_meta_*-(OH)Tyr), 7.03-6.99 (m, 2H, 2x *H*_indol_-Trp), 6.75 (d, *J*=8.5 Hz, 2H, 2x C*H_orto_*-(OH)Tyr), 4.91-4.84 (m, 2H, C*H_α_*-Trp, C*H_α_*-Asn),^^[[1]](#footnote-1)^^ 4.61 (t, *J*=7.7 Hz, C*H_α_*-Lys), 4.54-4.44 (m, 3H, C*H_α_*-Phe, 2x C*H_α_*), 4.41-4.37 (m, 2H, C*H_α_*-Trp, C*H_α_*), 4.36-4.21 (m, 4H, C*H_α_*, C*H_α_*-Ser, C*H_α_*-Glu, C*H_α_*-Val), 4.15-4.11 (m, 2H, 2x C*H_α_*-Ile), 4.05 (dd, *J*=8.0 Hz, *J’*=4.9 Hz, 1H, C*H_α_*-Trp), 4.00 (d, *J*=16.5 Hz, 1H, one proton C*H_2α_*-Gly), 3.82-3.75 (m, 2H, C*H_2β_*-Ser), 3.72 (d, *J*=16.5 Hz, 1H, one proton C*H_2α_*-Gly), 3.46-3.42 (m, 1H, one proton C*H_2β_*-Trp), 3.34-3.25 (m, 2H, one proton C*H_2β_*-Trp, one proton C*H_2β_*-Trp),^^[[2]](#footnote-2)^^ 3.17-3.12 (m, 2H, one proton C*H_2β_*-Trp, one proton C*H_2β_*-Tyr), 3.08 (dd, *J*=8.0 Hz, *J’*=4.9 Hz, 1H, one proton C*H_2β_*-Phe), 2.97-2.82 (m, 10H, 4x C*H_2ε_*-Lys, one proton C*H_2β_*-Phe, one proton C*H_2β_*-Tyr), 2.78 (t, 2H, *J*=7.3 Hz, C*H_2β_*-Asn), 2.36-2.31 (m, 2H, C*H_2γ_*-Glu), 2.20-1.29 (m, 34H, 4x C*H_2β_*-Lys, 4x C*H_2γ_*-Lys, 4x C*H_2δ_*-Lys, C*H_2β_*-Leu, C*H_γ_*-Leu, C*H_2β_*-Glu, C*H_β_*-Val, 2x C*H_β_*-Ile, 2x one proton C*H_2γ_*-Ile), 1.13-1.06 (m, 2H, 2x one proton C*H_2γ_*-Ile), 0.98-0.76 (m, 24H, 2x C*H_3δ_*-Leu, 2x C*H_3γ_*-Val, 2x C*H_3γ_*-Ile, 2x C*H_3δ_*-Ile).

**M1.6. Synthesis of CATL1.2_CECB1_2**

Following the general procedure described above, CATL1.2_CECB1_2 (WLVKNSWGKLGKKI) was obtained in >99% purity HPLC (λ=220 nm): *t*_R_ 4.83. ESI-HRMS (*m/z*) calculated for C_80_H_130_N_22_O_16_, M+nNa; [M+Na] ^+^ 1677.9927 found 1677.9901; calculated for C_80_H_130_N_22_O_16_, M+nH; [M+H] ^+^ 1656.0108 found 1656.0120 [M+2H]^2+^ 828.5090 found 828.5069, [M+3H]^3+^ 552.6751 found 552.6745. ^1^H-NMR (400 MHz, CD_3_OD) δ (ppm) 7.70 (d, *J*=7.9 Hz, 1H, *H*_indol_-Trp), 7.55 (d, *J*=7.9 Hz, 1H, *H*_indol_-Trp), 7.39 (d, *J*=8.1 Hz, 1H, *H*_indol_-Trp), 7.34 (d, *J*=8.1 Hz, 1H, *H*_indol_-Trp), 7.22 (s, 1H, *H*_indol_-Trp), 7.17 (s, 1H, *H*_indol_-Trp), 7.15-7.08 (m, 3H, 3x *H*_indol_-Trp), 7.08 (t, *J*=7.2 Hz, 1H, *H*_indol_-Trp), 4.73 (t, *J*=6.3 Hz, 1H, C*H_α_*-Asn), 4.61 (dd, *J*=9.4 Hz, *J’*=5.7 Hz, 1H, C*H_α_*-Lys or Leu), 4.52 (dd, *J*=8.9 Hz, *J’*=5.5 Hz, 1H, C*H_α_*-Trp), 4.41-4.35 (m, 4H, 4x C*H_α_*-Lys or Leu), 4.30 (dd, *J*=9.2 Hz, *J’*=5.2 Hz, 1H, C*H_α_*-Lys or Leu), 4.27-4.22 (m, 3H, C*H_α_*-Ser, C*H_α_*-Ile, C*H_α_*-Trp), 4.19 (d, *J*=7.5 Hz, 1H, C*H_α_*-Val), 3.92-3.87 (m, 2H, C*H_2α_*-Gly), 3.77-3.73, (m, 2H, C*H_2β_*-Ser), 3.47 (dd, *J*=15.0 Hz, *J’*=4.6 Hz, one proton C*H_2β_*-Trp), 3.37 (dd, *J*=14.9 Hz, *J’*=5.5 Hz, one proton C*H_2β_*-Trp), 3.25 (dd, *J*=15.0 Hz, *J’*=9.2 Hz, one proton C*H_2β_*-Trp), 3.17 (dd, *J*=15.1 Hz, *J’*=9.4 Hz, one proton C*H_2β_*-Trp), 2.96-2.88 (m, 8H, 4x C*H_2ε_*-Lys), 2.85-2.77 (m, 2H, C*H_2β_*-Asn), 2.14-2.03 (m, 1H, C*H_β_*-Val), 1.93-1.26 (m, 32H, , 4x C*H_2β_*-Lys, 4x C*H_2γ_*-Lys, 4x C*H_2δ_*-Lys, 2x C*H_2β_*-Leu, 2x C*H_γ_*-Leu, C*H_β_*-Ile, 2x one proton C*H_2γ_*-Ile), 1.23-1.11 (m, 2H, 2x one proton C*H_2γ_*-Ile), 0.99-0.87 (m, 24H, 4x C*H_3δ_*-Leu, 2x C*H_3γ_*-Val, C*H_3γ_*-Ile, C*H_3δ_*-Ile).

**M1.7. Synthesis of CECB1_CATL1.2**

Following the general procedure described above, CECB1_CATL1.2 (KIGKKIEYWLVKNSWG) was obtained in >99% purity HPLC (λ=220 nm): *t*_R_ 5.14. ESI-HRMS (*m/z*) calculated for C_94_H_146_N_24_O_21_, M+nNa; [M+Na] ^+^ 1971.1016 found 1971.0934; calculated for C_94_H_146_N_24_O_21_, M+nH; [M+H]^+^ 19479.1196 found 1949.1112 [M+2H]^2+^ 975.0635 found 975.0611, [M+3H]^3+^ 650.3781 found 650.3765. ^1^H-NMR (400 MHz, CD_3_OD) δ (ppm) 8.35-7.90 (residual N*H*, no CD_3_OD-interchangeable), 7.59-7.55 (m, 2H, *H*_indol_-Trp), 7.40 (d, *J*=8.1 Hz, 1H, *H*_indol_-Trp), 7.31 (d, *J*=8.1 Hz, 1H, *H*_indol_-Trp), 7.26 (s, 1H, *H*_indol_-Trp), 7.23 (s, 1H, *H*_indol_-Trp), 7.16 (t, *J*=7.3 Hz, 1H, *H*_indol_-Trp), 7.07 (t, *J*=7.6 Hz, 2H, *H*_indol_-Trp), 6.99 (t, *J*=7.2 Hz, 1H, *H*_indol_-Trp), 6.76 (d, *J*=8.3 Hz, 2H, 2x C*H_meta_*-(OH)Tyr), 6.48 (d, *J*=8.3 Hz, 2H, 2x C*H_orto_*-(OH)Tyr), 4.60 (dd, *J*=9.7 Hz, *J’*=4.1 Hz, 1H, C*H_α_*-Asn), 4.55 (dd, *J*=8.7 Hz, *J’*=4.7 Hz, 1H, C*H_α_*-Trp), 4.38 (dd, *J*=9.1 Hz, *J’*=5.7 Hz, 1H, C*H_α_*-Lys), 4.31 (dd, *J*=6.4 Hz, *J’*=3.8 Hz, 1H, C*H_α_*-Ser), 4.24-4.17 (m, 2H, C*H_α_*-Trp, C*H_α_*-Lys), 4.14 (t, *J*=8.3 Hz, 1H, C*H_α_*-Lys), 4.08-4.04 (m, 3H, C*H_α_*-Tyr, C*H_α_*-Glu, C*H_α_*-Leu), 4.01-3.84 (m, 6H, 2x C*H_α_*-Ile, C*H_2α_*-Gly, C*H_α_*-Lys, one proton C*H_2β_*-Ser), 3.81-3.72 (m, 3H, C*H_2α_*-Gly, one proton C*H_2β_*-Ser), 3.66 (dd, *J*=9.6 Hz, *J’*=5.7 Hz, 1H, C*H_α_*-Val), 3.49 (dd, *J*=14.2 Hz, *J’*=7.0 Hz, 1H, one proton C*H_2β_*-Trp), 3.39 (dd, *J*=14.2 Hz, *J’*=4.9 Hz, 1H, one proton C*H_2β_*-Trp), 3.34-3.34 (m, 1H, one proton C*H_2β_*-Trp),^^[[3]](#footnote-3)^^ 3.22 (dd, *J*=14.9 Hz, *J’*=9.0 Hz, 1H, one proton C*H_2β_*-Trp), 3.12-3.03 (m, 2H, C*H_2β_*-Tyr), 2.97-2.91 (m, 6H, 3x C*H_2ε_*-Lys), 2.88-2.74 (m, 2H, C*H_2ε_*-Lys), 2.65 (dd, *J*=15.3 Hz, *J’*=4.1 Hz, 1H, one proton C*H_2β_*-Asn), 2.54 (dd, *J*=15.3 Hz, *J’*=9.7 Hz, 1H, one proton C*H_2β_*-Asn), 2.52-2.36 (m, 2H, C*H_2γ_*-Glu), 2.23-2.07 (m, 3H, C*H_2β_*-Glu, C*H_β_*-Val), 2.06-1.41 (m, 31H, 4x C*H_2β_*-Lys, 4x C*H_2γ_*-Lys, 4x C*H_2δ_*-Lys, C*H_2β_*-Leu, C*H_γ_*-Leu, 2x C*H_β_*-Ile, 2x one proton C*H_2γ_*-Ile), 1.33-1.20 (m, 2H, 2x one proton C*H_2γ_*-Ile), 1.07 (d, *J*=6.4 Hz, 3H, C*H_3γ_*-Val), 1.02 (d, *J*=6.7 Hz, 3H, C*H_3γ_*-Ile), 1.00-0.90 (m, 15H, C*H_3γ_*-Ile, 2x C*H_3δ_*-Ile, 2x C*H_3δ_*-Leu), 0.88 (d, *J*=6.7 Hz, 3H, C*H_3γ_*-Val)

**HPLC of CATL1.2_CECB2 (YWLVKNSWGKIFKKIE)**

**
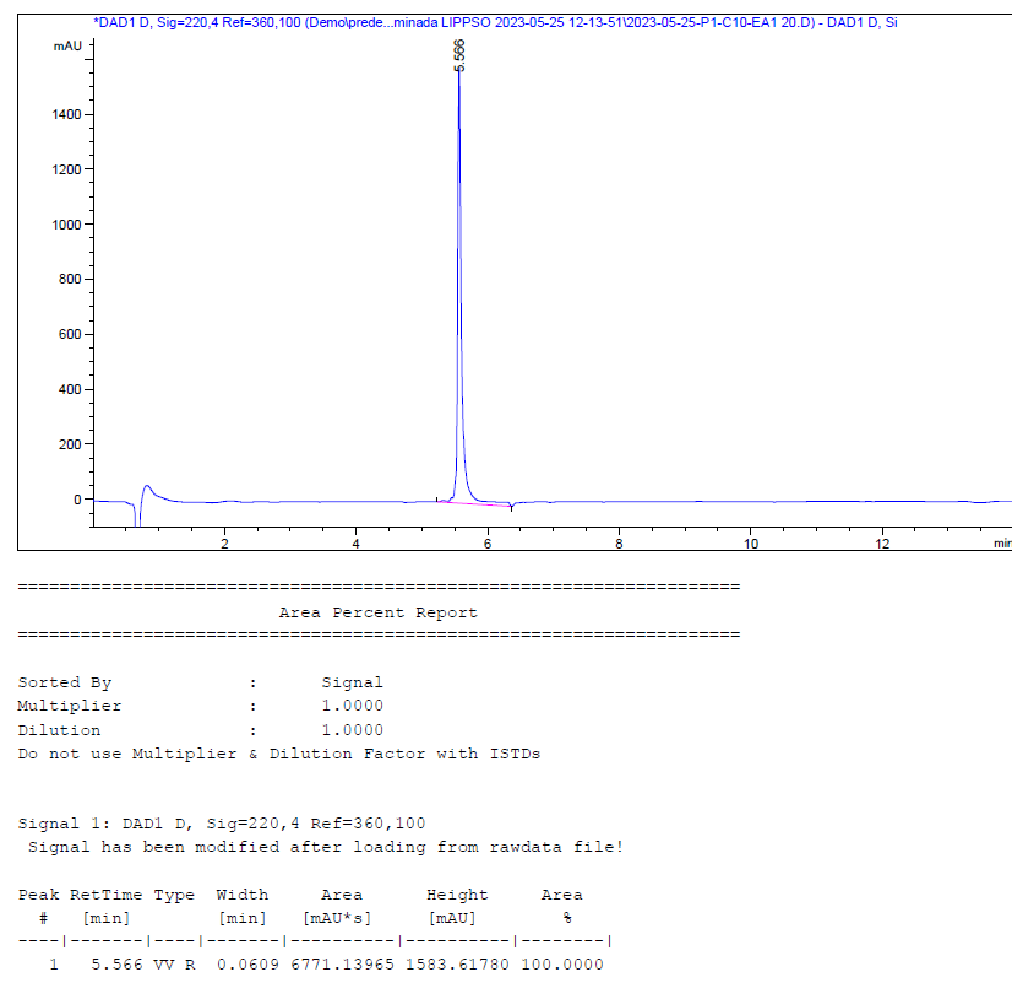
Figure S6.** Representative Chromatogram of synthesis purification process of **CATL1.2_CECB2 (YWLVKNSWGKIFKKIE)** hybrid peptide

**ESI-HRMS of CATL1.2_CECB2 (YWLVKNSWGKIFKKIE)**


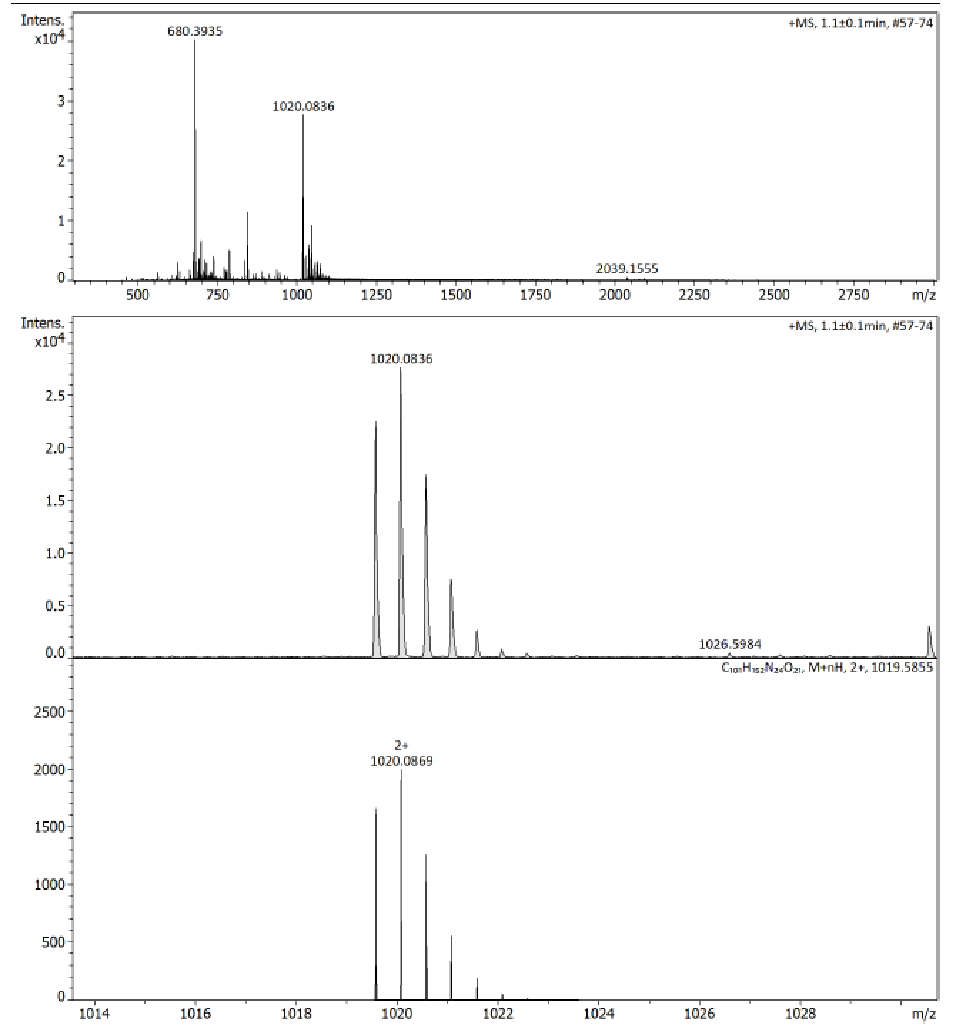


[M+H]^+^

[M+2H]^2+^

[M+3H]^3+^

[M+2H]^2+^

**Figure S7.** Representative ESI-HRMS purification process of **CATL1.2_CECB2 (YWLVKNSWGKIFKKIE)** hybrid peptide.

**^1^H-NMR of CATL1.2_CECB2 (YWLVKNSWGKIFKKIE)**

**A)**


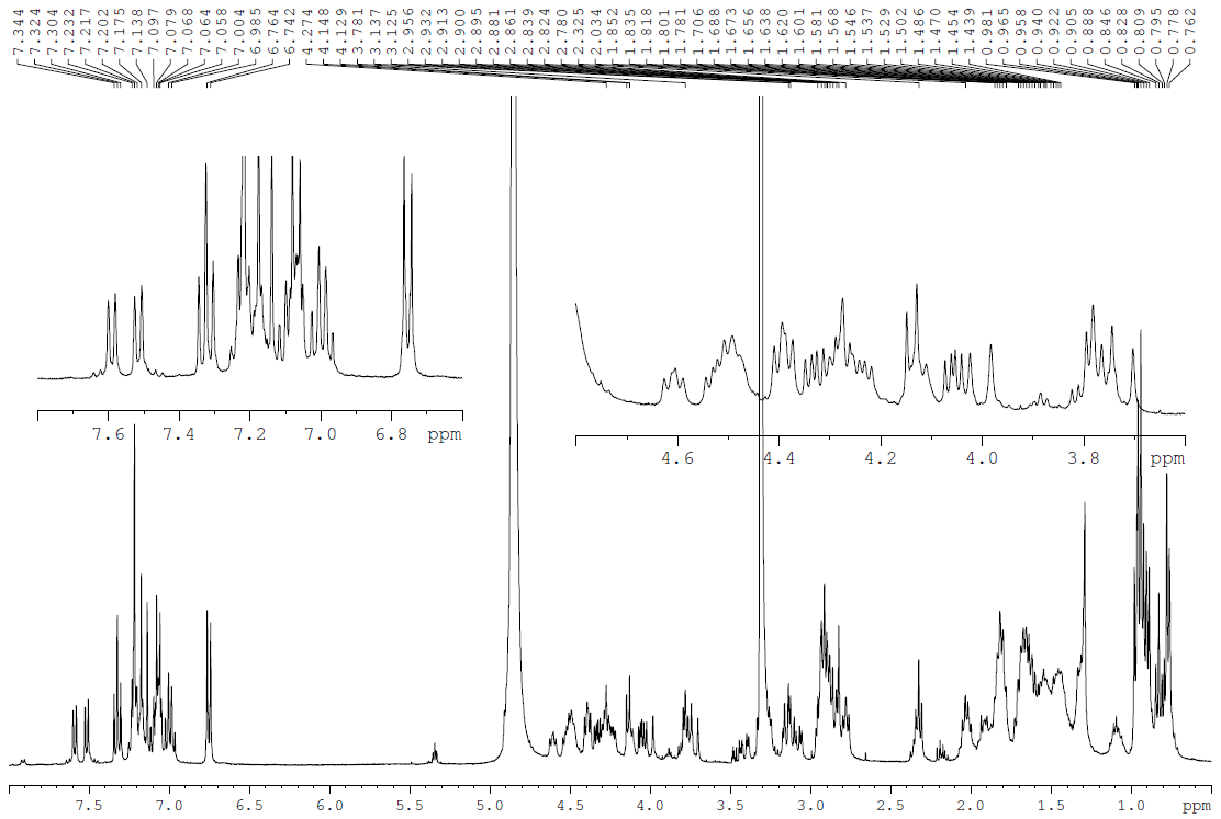


**B**

**
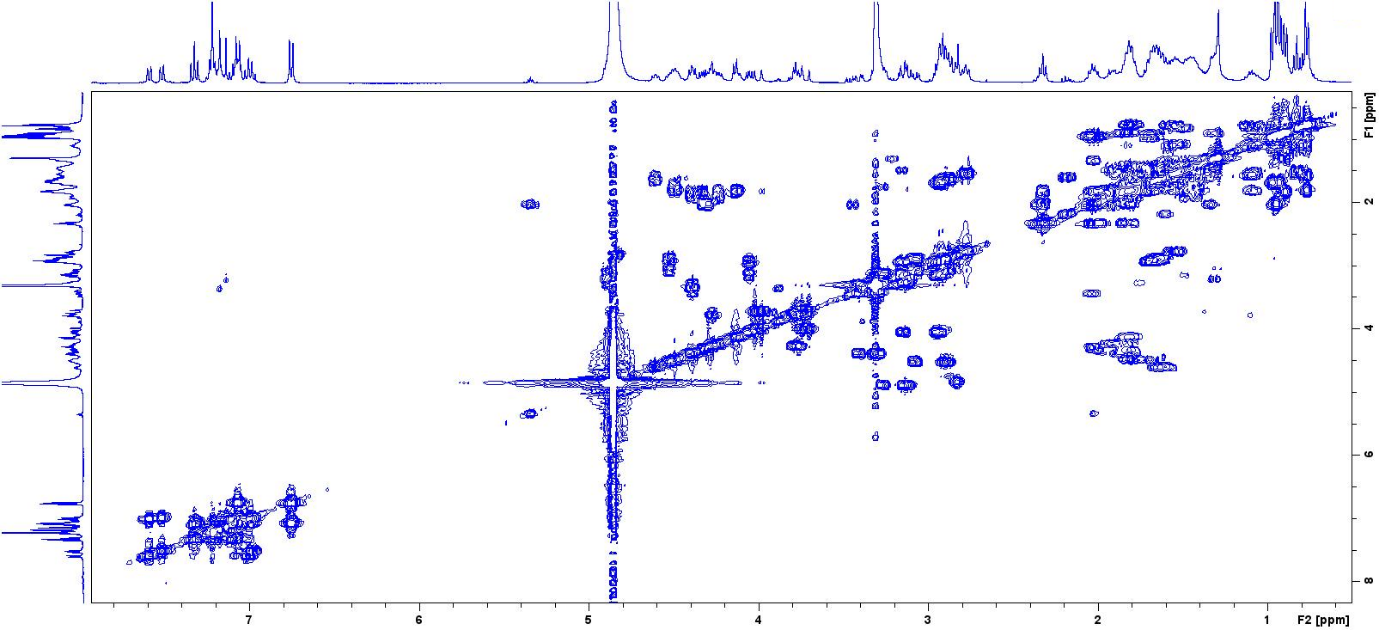
**

**
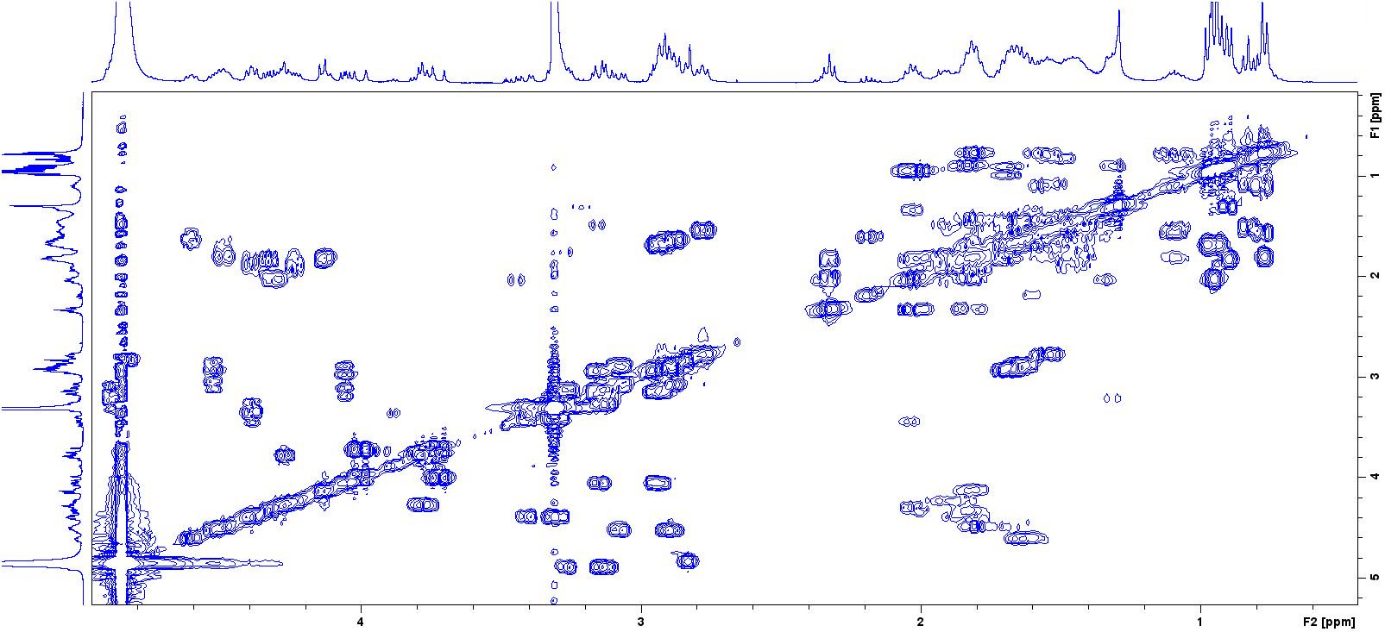
**

**Figure S8.** Representative (A) ^1^H-NMR and (B) COSY NMR purification process of **CATL1.2_CECB2 (YWLVKNSWGKIFKKIE)** hybrid peptide.

**HPLC of CATL1.2_CECB1_2 (WLVKNSWGKLGKKI)**

**
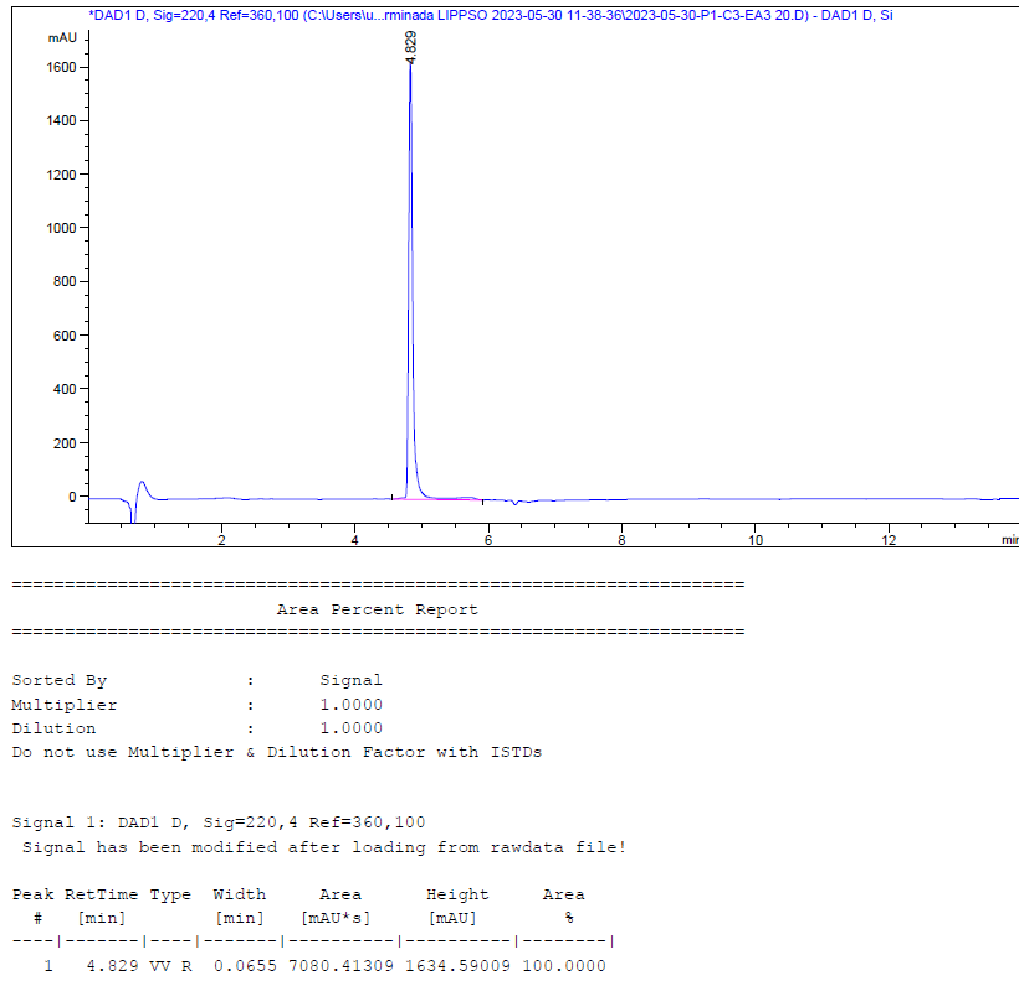
**

**Figure S9.** Representative Chromatogram of synthesis purification process of **CATL1.2_CECB1_2 (WLVKNSWGKLGKKI)** hybrid peptide.

**ESI-HRMS of CATL1.2_CECB1_2 (WLVKNSWGKLGKKI)**

**
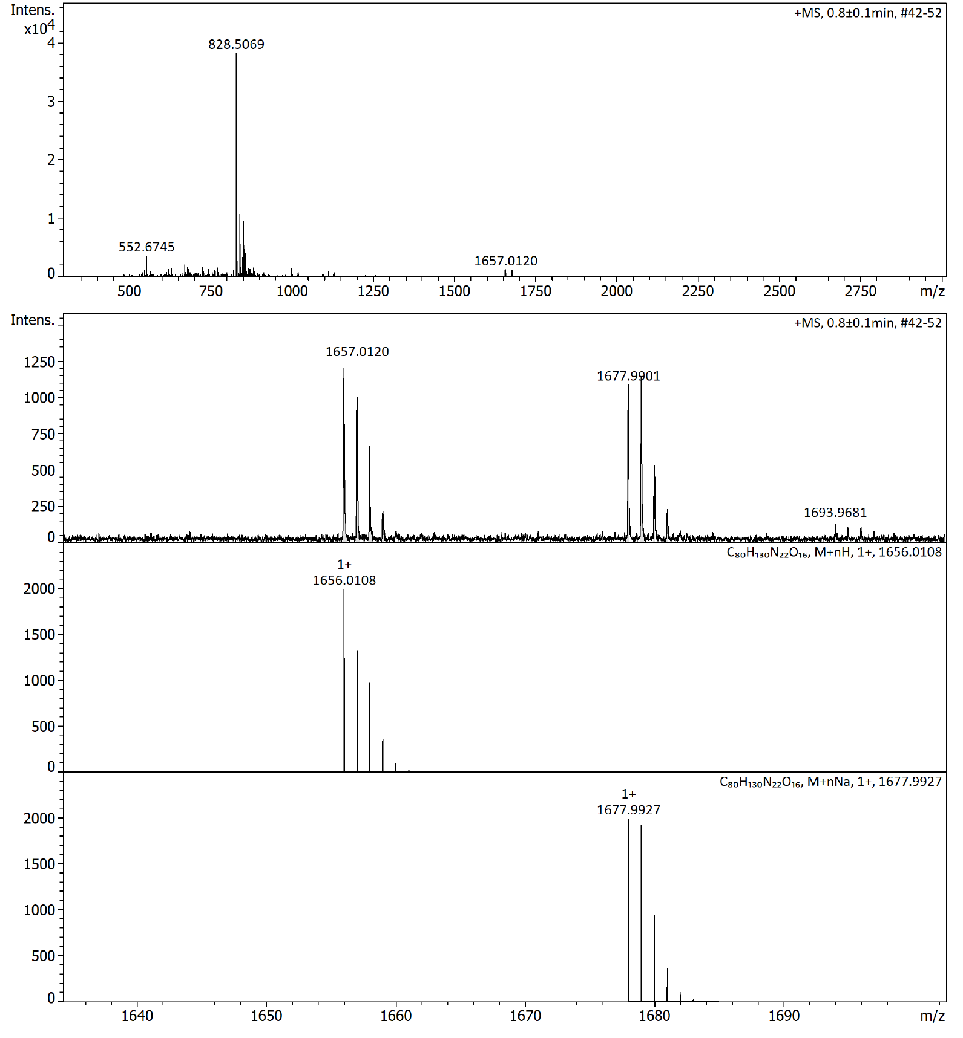
**

[M+H]^+^

[M+H]^+^

[M+Na]^+^

[M+2H]^2+^

[M+3H]^3^+^+^

**Figure S10.** Representative ESI-HRMS purification process of **CATL1.2_CECB1_2 (WLVKNSWGKLGKKI)** hybrid peptide.

**^1^H-NMR of CATL1.2_CECB1_2 (WLVKNSWGKLGKKI)**

**A)**


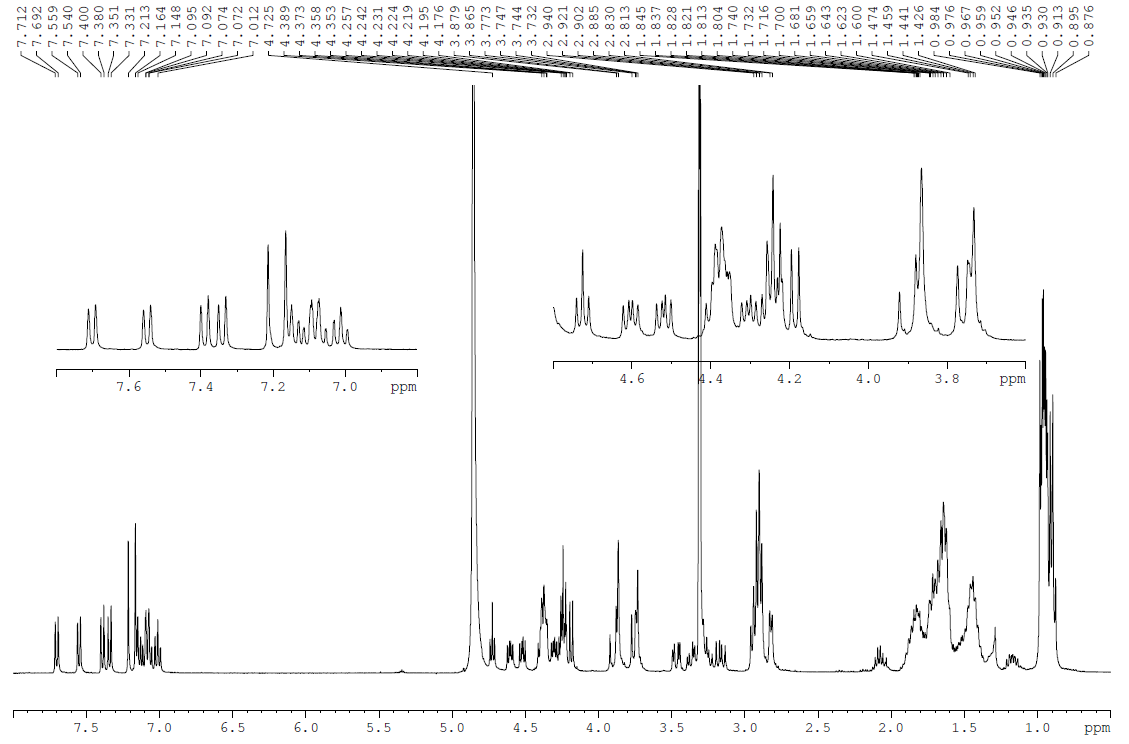


**COSY of CATL1.2_CECB1_2 (WLVKNSWGKLGKKI)**

**B)**

**
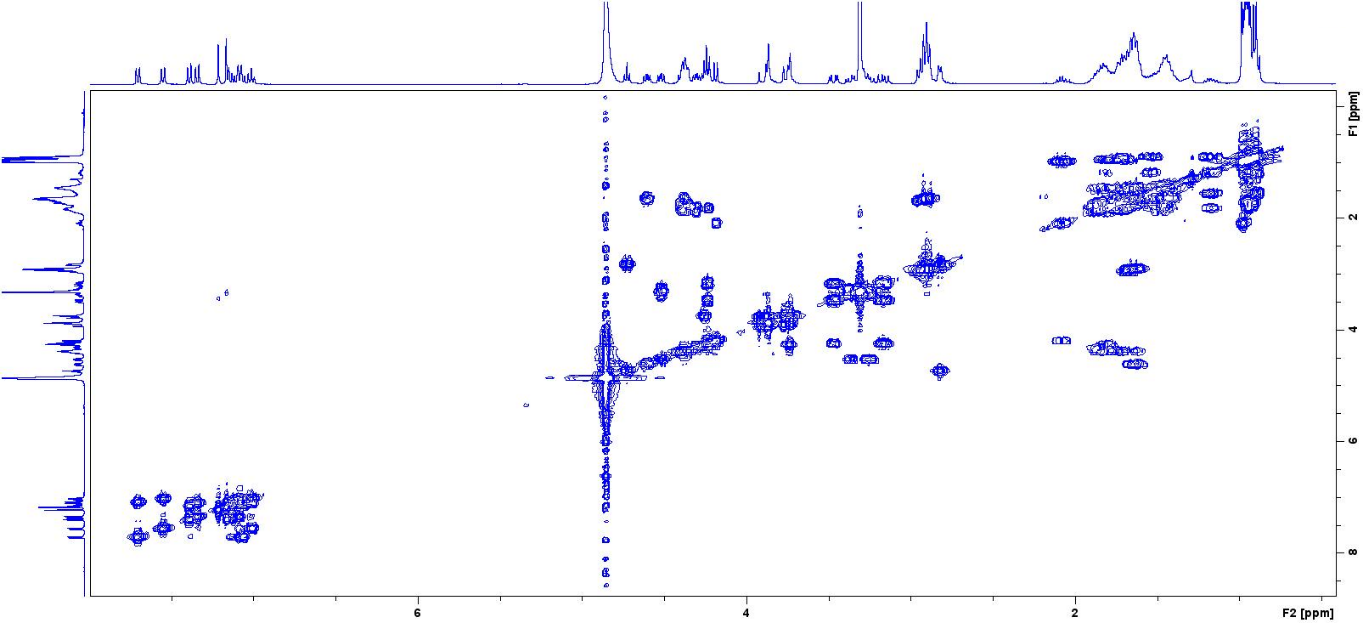
**

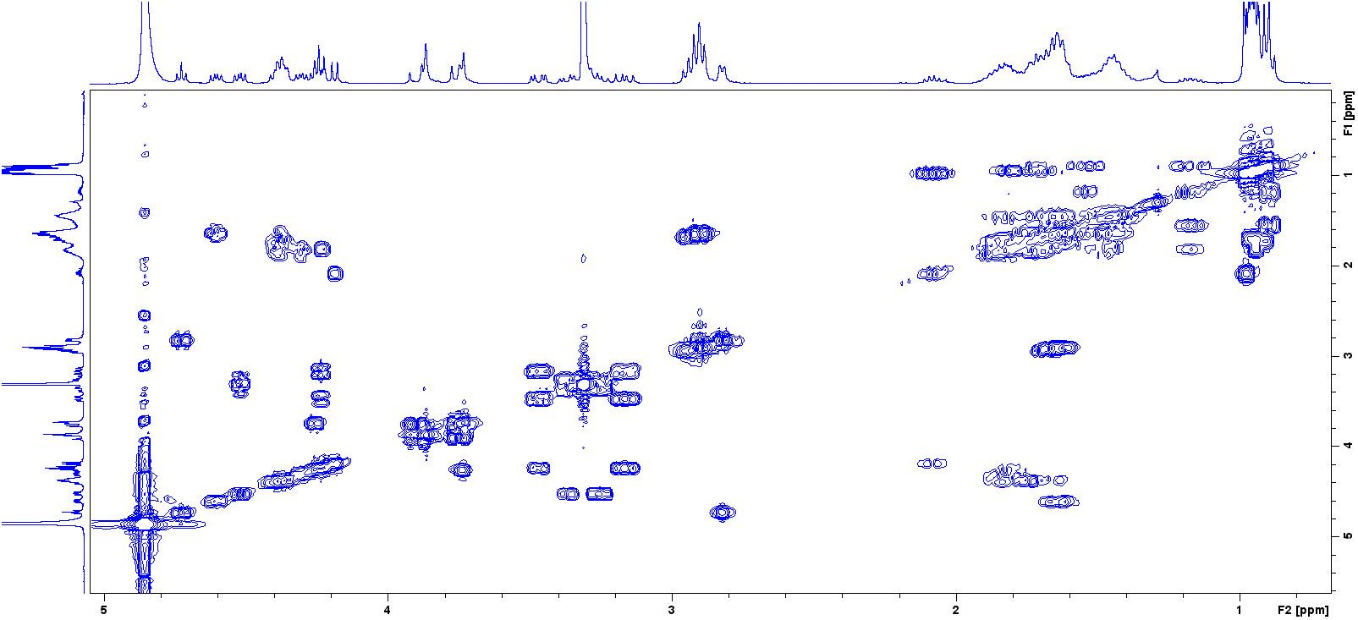


**Figure S11.** Representative (A) ^1^H-NMR and (B) COSY NMR purification process of **CATL1.2_CECB1_2 (WLVKNSWGKLGKKI)** hybrid peptide.

**HPLC of CECB1_CATL1.2 (KIGKKIEYWLVKNSWG)**

**
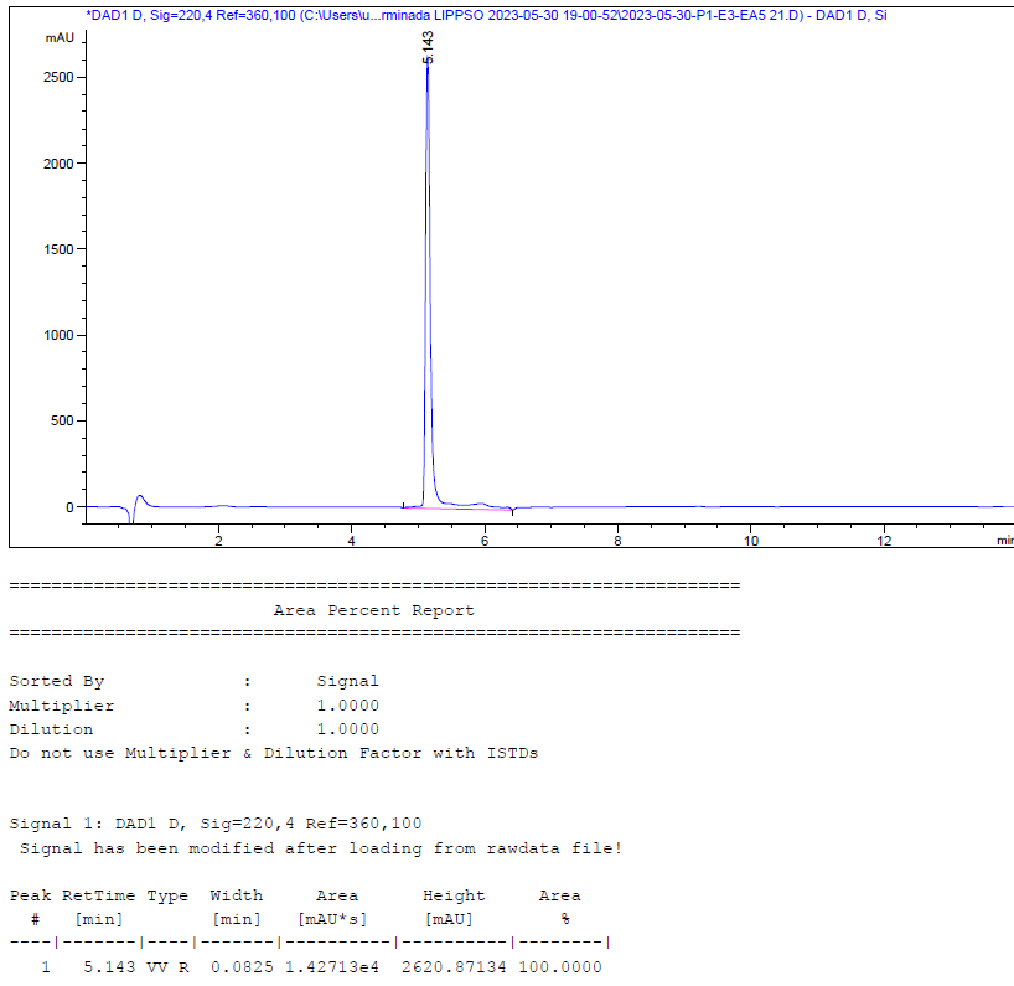
**

**Figure S12.** Representative Chromatogram of synthesis purification process of **CECB1_CATL1.2 (KIGKKIEYWLVKNSWG)** hybrid peptide

**ESI-HRMS of CECB1_CATL1.2 (KIGKKIEYWLVKNSWG)**

**
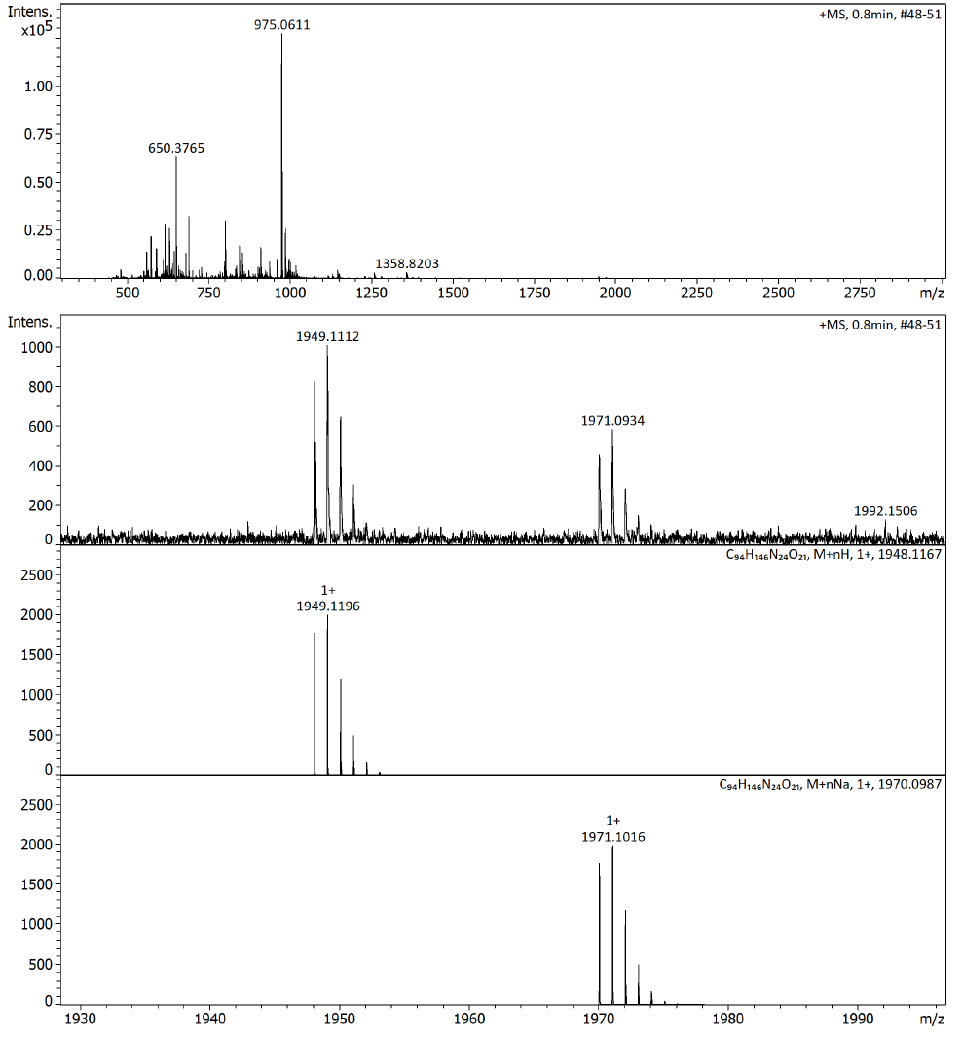
**

[M+H]^+^

[M+Na]^+^

[M+2H]^2+^

[M+3H]^3+^

**Figure S13.** Representative ESI-HRMS purification process **CECB1_CATL1.2 (KIGKKIEYWLVKNSWG)** hybrid peptide.

**^1^H-NMR of CECB1_CATL1.2 (KIGKKIEYWLVKNSWG)**

A)


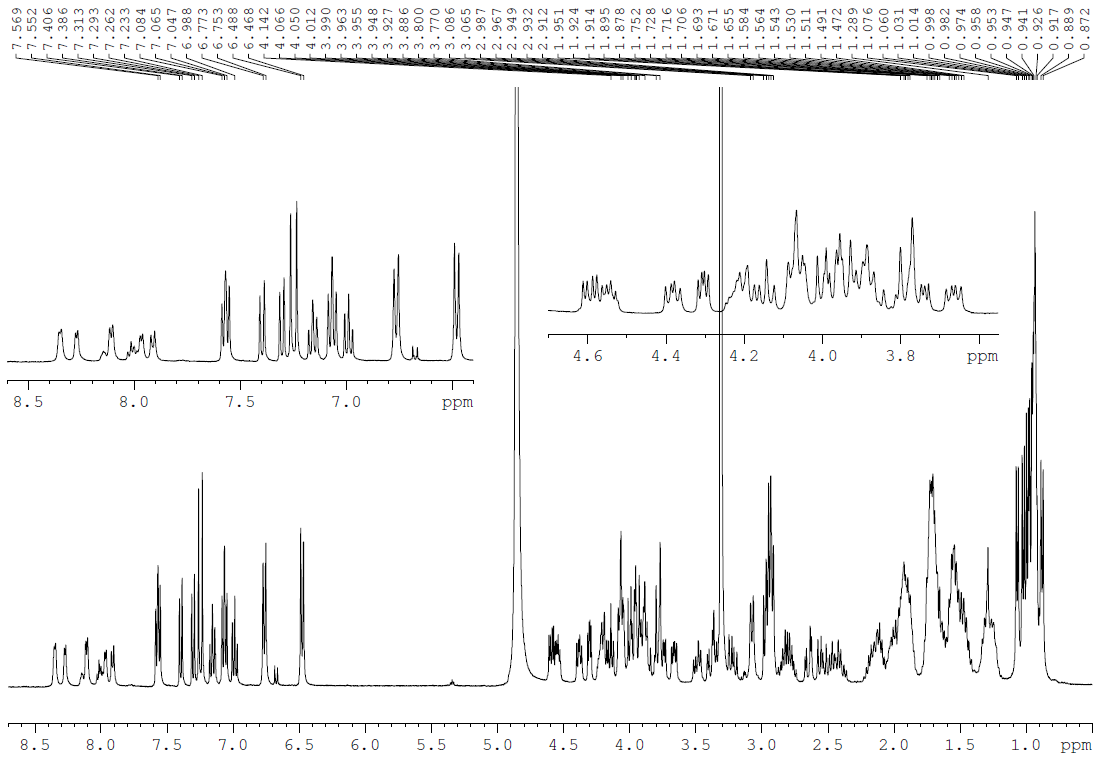


**COSY of CECB1_CATL1.2 (KIGKKIEYWLVKNSWG)**

B)


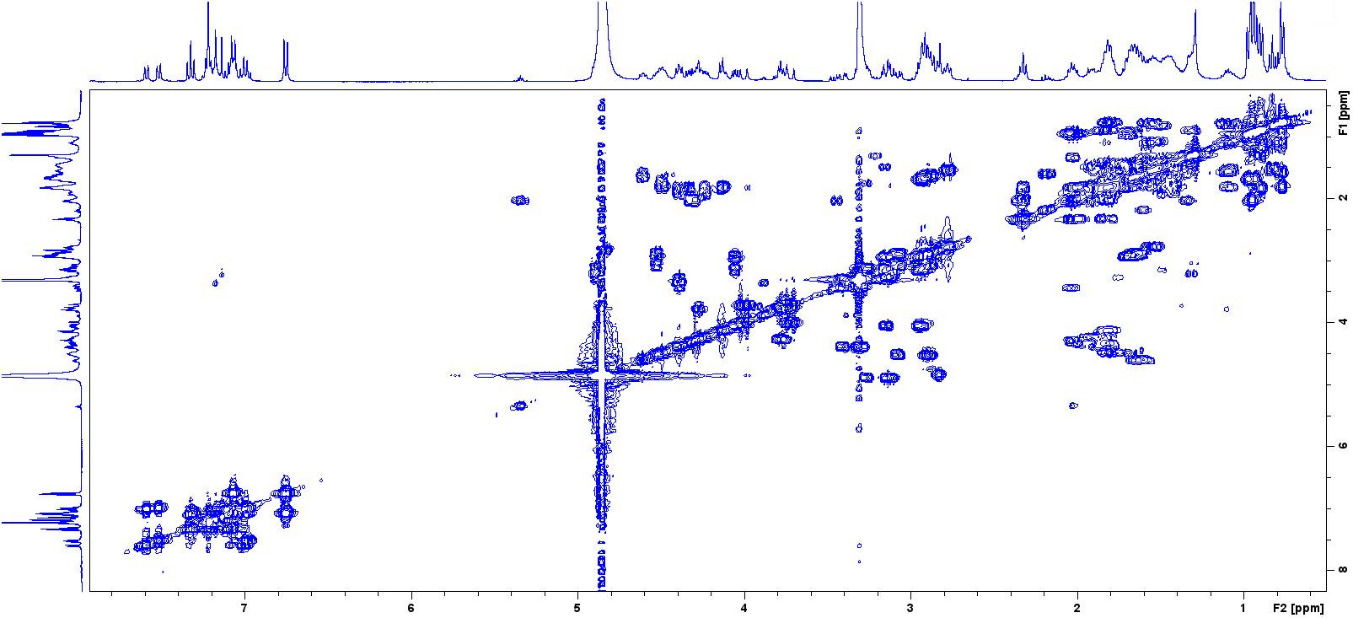


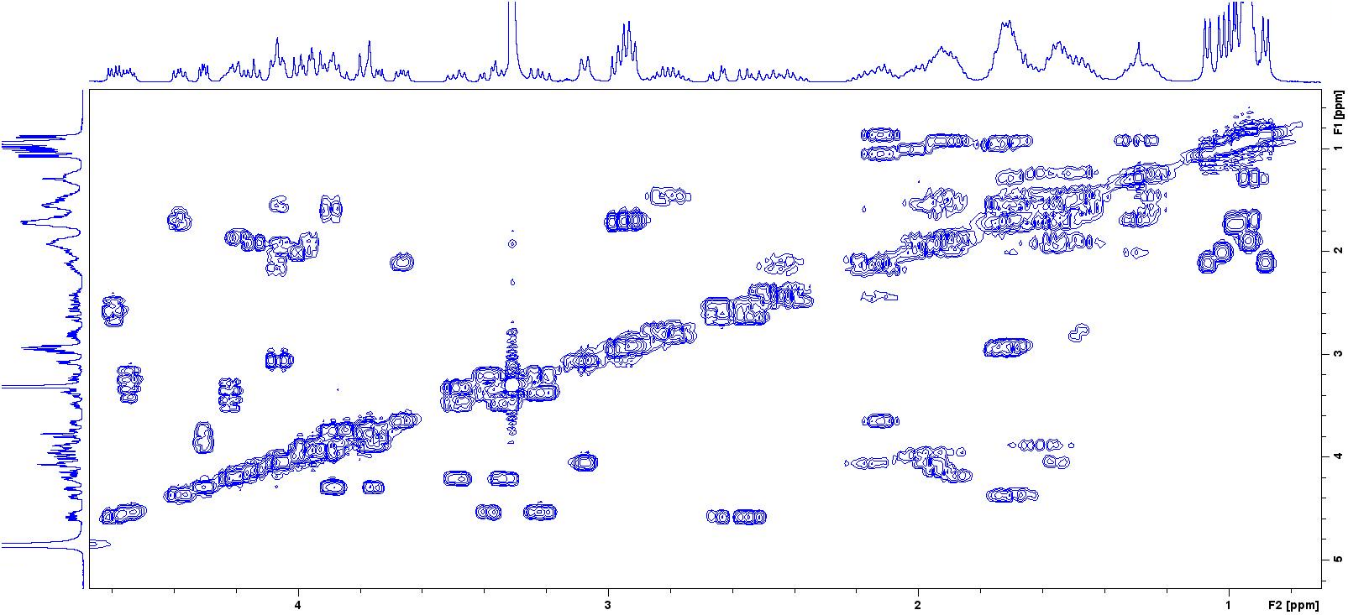


**Figure S14.** Representative (A) ^1^H-NMR and (B) COSY NMR purification process of **CECB1_CATL1.2 (KIGKKIEYWLVKNSWG)** hybrid peptide.

**Table S1 – Cecropin and Cathepsins conserved sequences**

| **ALIGNMENT CECROPIN AND CATHEPSINS** | | | |
| --- | --- | --- | --- |
| **PROTEIN/PEPTIDE** | **IDENTIFIERS** | **NAME** | **CONSERVED SEQUENCE** |
| CECROPIN | P83403 | CEC_GLOMM | KKIERVGQNTRDA |
| CECROPIN | P83420 | CEC_OIKKI | KKIEKAVRRVRDG |
| CECROPIN | P14666 | CEC4_BOMMO | KKIEKVGQNIRDG |
| CECROPIN | C0HJX8 | CEC_CALVI | KKIGRVGQHTRDA |
| CECROPIN A | P82592 | CECA_AEDAE | KLGKKLEGAGKR |
| CECROPIN A | C0HKQ7 | CECA1_DROME | KIGKKIERVGQH |
|  | C0HKQ8 | CECA2_DROME |  |
|  | O61272 | CECA1_DROSI |  |
|  | P81685 | CECA1_DROMA |  |
|  | P81688 | CECA1_DROSE |  |
| CECROPIN A | P83413 | CECA_HELVI | KVFKKIEKVGRN |
|  | Q9XZG9 | CECA_SPOLT |  |
| CECROPIN A | Q86PR6 | CECA_CULPP | KFGKKLEGVGKR |
|  | Q963B0 | CECA2_AEDAL |  |
| CECROPIN B | P14956 | CECB_DROME | KLGKKIE |
|  | P67792 | CECB_DROSI |  |
|  | P67791 | CECB_DROSE |  |
|  | P81686 | CECB_DROMA |  |
|  | Q86PR4 | CECB2_CULPP |  |
|  | Q86PR5 | CECB1_CULPP |  |
| CECROPIN B | P04142 | CECB_BOMMO | KIFKKIE |
|  | P01509 | CECB_ANTPE |  |
| CECROPIN B | P83414 | CECB_HELVI | KVFKKIE |
|  | P01508 | CECB_HYACE |  |
|  | Q9XZH0 | CECB_SPOLT |  |
| CECROPIN C | O16829 | CECC_DROME | KLGKRIERIGQ |
|  | P84222 | CECC_DROER |  |
|  | P84020 | CECC_DROSE |  |
|  | P84021 | CECC_DROSI |  |
|  | P84019 | CECC_DROMA |  |
|  | P84224 | CECC_DROTK |  |
|  | P84226 | CECC_DROYA |  |
|  | P84225 | CECC_DROTE |  |
|  | P84223 | CECC_DROOR |  |
| CECROPIN C | Q9Y0X9 | CECC1_AEDAL | KLGKKLEGAGK |
|  | Q963A8 | CECC2_AEDAL |  |
| CECROPIN D | P85210 | CECD_GALME | GQRIRDAIISAAPAV |
| CECROPIN D | P01510 | CECD_HYACE | GQRVRDAVISAGPAV |
| CECROPIN D | P01511 | CECD_ANTPE | GQRVRDAIISAGPAV |
| CATHEPSINS | P55097 | CATK_MOUSE | HAVLVVGYG |
| CATHEPSINS | P25326 | CATS_BOVIN | HGVLVVGYG |
|  | P25774 | CATS_HUMAN |  |
| PRO-CATHEPSINS | P09668 | CATH_HUMAN | PVKNQGACGSCWTFS |
|  | P00786 | CATH_RAT |  |
| PRO-CATHEPSINS | P06797 | CATL1_MOUSE | PVKNQGQCGSCWAFS |
|  | P07711 | CATL1_HUMAN |  |
| PRO-CATHEPSINS | P09668 | CATH_HUMAN | YWIVKNSWG |
|  | P00786 | CATH_RAT |  |
| PRO-CATHEPSINS | P06797 | CATL1_MOUSE | YWLVKNSWG |
|  | P07711 | CATL1_HUMAN |  |

**Table S2 - Hybrid peptide formation**

| **HYBRID - CECROPIN N-TERMINAL** | | | | | |  | **HYBRID - CATHEPSINS N-TERMINAL** | | | | | |
| --- | --- | --- | --- | --- | --- | --- | --- | --- | --- | --- | --- | --- |
| **NAME CONSERVED CECROPIN** | **SEQUENCE CECROPIN** | **NAME CONSERVED CATHEPSINS** | **SEQUENCE CATHEPSINS** | **NAME HYBRID** | **HYBRID PEPTIDE** |  | **NAME CONSERVED CATHEPSINS** | **SEQUENCE CATHEPSINS** | **NAME CONSERVED CECROPIN** | **SEQUENCE CECROPIN** | **NAME** | **HYBRID PEPTIDE** |
|  |  |  |  |  |  |  |  |  |  |  |  |  |
| **CEC1** | **KKIERVGQNTRDA** | **CATK** | **HAVLVVGYG** | **CEC1_CATK** | **KKIERVGQNTRDAHAVLVVGYG** |  | **CATK** | **HAVLVVGYG** | **CEC1** | **KKIERVGQNTRDA** | **CATK_CEC1** | **HAVLVVGYGKKIERVGQNTRDA** |
|  | **KKIERVGQNTRDA** | **CATS** | **HGVLVVGYG** | **CEC1_CATS** | **KKIERVGQNTRDAHGVLVVGYG** |  | **CATS** | **HGVLVVGYG** |  | **KKIERVGQNTRDA** | **CATS_CEC1** | **HGVLVVGYGKKIERVGQNTRDA** |
|  | **KKIERVGQNTRDA** | **CATH1** | **PVKNQGACGSCWTFS** | **CEC1_CATH1** | **KKIERVGQNTRDAPVKNQGACGSCWTFS** |  | **CATH1** | **PVKNQGACGSCWTFS** |  | **KKIERVGQNTRDA** | **CATH1_CEC1** | **PVKNQGACGSCWTFSKKIERVGQNTRDA** |
|  | **KKIERVGQNTRDA** | **CATL1** | **PVKNQGQCGSCWAFS** | **CEC1_CATL1** | **KKIERVGQNTRDAPVKNQGQCGSCWAFS** |  | **CATL1** | **PVKNQGQCGSCWAFS** |  | **KKIERVGQNTRDA** | **CATL1_CEC1** | **PVKNQGQCGSCWAFSKKIERVGQNTRDA** |
|  | **KKIERVGQNTRDA** | **CATH2** | **YWIVKNSWG** | **CEC1_CATH2** | **KKIERVGQNTRDAYWIVKNSWG** |  | **CATH2** | **YWIVKNSWG** |  | **KKIERVGQNTRDA** | **CATH2_CEC1** | **YWIVKNSWGKKIERVGQNTRDA** |
|  | **KKIERVGQNTRDA** | **CATL1.2** | **YWLVKNSWG** | **CEC1_CATL1.2** | **KKIERVGQNTRDAYWLVKNSWG** |  | **CATL1.2** | **YWLVKNSWG** |  | **KKIERVGQNTRDA** | **CATL1.2_CEC1** | **YWLVKNSWGKKIERVGQNTRDA** |
| **CEC2** | **KKIEKAVRRVRDG** | **CATK** | **HAVLVVGYG** | **CEC2_CATK** | **KKIEKAVRRVRDGHAVLVVGYG** |  | **CATK** | **HAVLVVGYG** | **CEC2** | **KKIEKAVRRVRDG** | **CATK_CEC2** | **HAVLVVGYGKKIEKAVRRVRDG** |
|  | **KKIEKAVRRVRDG** | **CATS** | **HGVLVVGYG** | **CEC2_CATS** | **KKIEKAVRRVRDGHGVLVVGYG** |  | **CATS** | **HGVLVVGYG** |  | **KKIEKAVRRVRDG** | **CATS_CEC2** | **HGVLVVGYGKKIEKAVRRVRDG** |
|  | **KKIEKAVRRVRDG** | **CATH1** | **PVKNQGACGSCWTFS** | **CEC2_CATH1** | **KKIEKAVRRVRDGPVKNQGACGSCWTFS** |  | **CATH1** | **PVKNQGACGSCWTFS** |  | **KKIEKAVRRVRDG** | **CATH1_CEC2** | **PVKNQGACGSCWTFSKKIEKAVRRVRDG** |
|  | **KKIEKAVRRVRDG** | **CATL1** | **PVKNQGQCGSCWAFS** | **CEC2_CATL1** | **KKIEKAVRRVRDGPVKNQGQCGSCWAFS** |  | **CATL1** | **PVKNQGQCGSCWAFS** |  | **KKIEKAVRRVRDG** | **CATL1_CEC2** | **PVKNQGQCGSCWAFSKKIEKAVRRVRDG** |
|  | **KKIEKAVRRVRDG** | **CATH2** | **YWIVKNSWG** | **CEC2_CATH2** | **KKIEKAVRRVRDGYWIVKNSWG** |  | **CATH2** | **YWIVKNSWG** |  | **KKIEKAVRRVRDG** | **CATH2_CEC2** | **YWIVKNSWGKKIEKAVRRVRDG** |
|  | **KKIEKAVRRVRDG** | **CATL1.2** | **YWLVKNSWG** | **CEC2_CATL.2** | **KKIEKAVRRVRDGYWLVKNSWG** |  | **CATL1.2** | **YWLVKNSWG** |  | **KKIEKAVRRVRDG** | **CATL1.2_CEC2** | **YWLVKNSWGKKIEKAVRRVRDG** |
| **CEC3** | **KKIEKVGQNIRDG** | **CATK** | **HAVLVVGYG** | **CEC3_CATK** | **KKIEKVGQNIRDGHAVLVVGYG** |  | **CATK** | **HAVLVVGYG** | **CEC3** | **KKIEKVGQNIRDG** | **CATK_CEC3** | **HAVLVVGYGKKIEKVGQNIRDG** |
|  | **KKIEKVGQNIRDG** | **CATS** | **HGVLVVGYG** | **CEC3_CATS** | **KKIEKVGQNIRDGHGVLVVGYG** |  | **CATS** | **HGVLVVGYG** |  | **KKIEKVGQNIRDG** | **CATS_CEC3** | **HGVLVVGYGKKIEKVGQNIRDG** |
|  | **KKIEKVGQNIRDG** | **CATH1** | **PVKNQGACGSCWTFS** | **CEC3_CATH1** | **KKIEKVGQNIRDGPVKNQGACGSCWTFS** |  | **CATH1** | **PVKNQGACGSCWTFS** |  | **KKIEKVGQNIRDG** | **CATH1_CEC3** | **PVKNQGACGSCWTFSKKIEKVGQNIRDG** |
|  | **KKIEKVGQNIRDG** | **CATL1** | **PVKNQGQCGSCWAFS** | **CEC3_CATL1** | **KKIEKVGQNIRDGPVKNQGQCGSCWAFS** |  | **CATL1** | **PVKNQGQCGSCWAFS** |  | **KKIEKVGQNIRDG** | **CATL1_CEC3** | **PVKNQGQCGSCWAFSKKIEKVGQNIRDG** |
|  | **KKIEKVGQNIRDG** | **CATH2** | **YWIVKNSWG** | **CEC3_CATH2** | **KKIEKVGQNIRDGYWIVKNSWG** |  | **CATH2** | **YWIVKNSWG** |  | **KKIEKVGQNIRDG** | **CATH2_CEC3** | **YWIVKNSWGKKIEKVGQNIRDG** |
|  | **KKIEKVGQNIRDG** | **CATL1.2** | **YWLVKNSWG** | **CEC3_CATL1.2** | **KKIEKVGQNIRDGYWLVKNSWG** |  | **CATL1.2** | **YWLVKNSWG** |  | **KKIEKVGQNIRDG** | **CATL1.2_CEC3** | **YWLVKNSWGKKIEKVGQNIRDG** |
| **CEC4** | **KKIGRVGQHTRDA** | **CATK** | **HAVLVVGYG** | **CEC4_CATK** | **KKIGRVGQHTRDAHAVLVVGYG** |  | **CATK** | **HAVLVVGYG** | **CEC4** | **KKIGRVGQHTRDA** | **CATK_CEC4** | **HAVLVVGYGKKIGRVGQHTRDA** |
|  | **KKIGRVGQHTRDA** | **CATS** | **HGVLVVGYG** | **CEC4_CATS** | **KKIGRVGQHTRDAHGVLVVGYG** |  | **CATS** | **HGVLVVGYG** |  | **KKIGRVGQHTRDA** | **CATS_CEC4** | **HGVLVVGYGKKIGRVGQHTRDA** |
|  | **KKIGRVGQHTRDA** | **CATH1** | **PVKNQGACGSCWTFS** | **CEC4_CATH1** | **KKIGRVGQHTRDAPVKNQGACGSCWTFS** |  | **CATH1** | **PVKNQGACGSCWTFS** |  | **KKIGRVGQHTRDA** | **CATH1_CEC4** | **PVKNQGACGSCWTFSKKIGRVGQHTRDA** |
|  | **KKIGRVGQHTRDA** | **CATL1** | **PVKNQGQCGSCWAFS** | **CEC4_CATL1** | **KKIGRVGQHTRDAPVKNQGQCGSCWAFS** |  | **CATL1** | **PVKNQGQCGSCWAFS** |  | **KKIGRVGQHTRDA** | **CATL1_CEC4** | **PVKNQGQCGSCWAFSKKIGRVGQHTRDA** |
|  | **KKIGRVGQHTRDA** | **CATH2** | **YWIVKNSWG** | **CEC4_CATH2** | **KKIGRVGQHTRDAYWIVKNSWG** |  | **CATH2** | **YWIVKNSWG** |  | **KKIGRVGQHTRDA** | **CATH2_CEC4** | **YWIVKNSWGKKIGRVGQHTRDA** |
|  | **KKIGRVGQHTRDA** | **CATL1.2** | **YWLVKNSWG** | **CEC4_CATL1.2** | **KKIGRVGQHTRDAYWLVKNSWG** |  | **CATL1.2** | **YWLVKNSWG** |  | **KKIGRVGQHTRDA** | **CATL1.2_CEC4** | **YWLVKNSWGKKIGRVGQHTRDA** |
| **CECA1** | **KLGKKLEGAGKR** | **CATK** | **HAVLVVGYG** | **CECA1_CATK** | **KLGKKLEGAGKRHAVLVVGYG** |  | **CATK** | **HAVLVVGYG** | **CECA1** | **KLGKKLEGAGKR** | **CATK_CECA1** | **HAVLVVGYGKLGKKLEGAGKR** |
|  | **KLGKKLEGAGKR** | **CATS** | **HGVLVVGYG** | **CECA1_CATS** | **KLGKKLEGAGKRHGVLVVGYG** |  | **CATS** | **HGVLVVGYG** |  | **KLGKKLEGAGKR** | **CATS_CECA1** | **HGVLVVGYGKLGKKLEGAGKR** |
|  | **KLGKKLEGAGKR** | **CATH1** | **PVKNQGACGSCWTFS** | **CECA1_CATH1** | **KLGKKLEGAGKRPVKNQGACGSCWTFS** |  | **CATH1** | **PVKNQGACGSCWTFS** |  | **KLGKKLEGAGKR** | **CATH1_CECA1** | **PVKNQGACGSCWTFSKLGKKLEGAGKR** |
|  | **KLGKKLEGAGKR** | **CATL1** | **PVKNQGQCGSCWAFS** | **CECA1_CATL1** | **KLGKKLEGAGKRPVKNQGQCGSCWAFS** |  | **CATL1** | **PVKNQGQCGSCWAFS** |  | **KLGKKLEGAGKR** | **CATL1_CECA1** | **PVKNQGQCGSCWAFSKLGKKLEGAGKR** |
|  | **KLGKKLEGAGKR** | **CATH2** | **YWIVKNSWG** | **CECA1_CATH2** | **KLGKKLEGAGKRYWIVKNSWG** |  | **CATH2** | **YWIVKNSWG** |  | **KLGKKLEGAGKR** | **CATH2_CECA1** | **YWIVKNSWGKLGKKLEGAGKR** |
|  | **KLGKKLEGAGKR** | **CATL1.2** | **YWLVKNSWG** | **CECA1_CATL1.2** | **KLGKKLEGAGKRYWLVKNSWG** |  | **CATL1.2** | **YWLVKNSWG** |  | **KLGKKLEGAGKR** | **CATL1.2_CECA1** | **YWLVKNSWGKLGKKLEGAGKR** |
| **CECA2** | **KIGKKIERVGQH** | **CATK** | **HAVLVVGYG** | **CECA2_CATK** | **KIGKKIERVGQHHAVLVVGYG** |  | **CATK** | **HAVLVVGYG** | **CECA2** | **KIGKKIERVGQH** | **CATK_CECA2** | **HAVLVVGYGKIGKKIERVGQH** |
|  | **KIGKKIERVGQH** | **CATS** | **HGVLVVGYG** | **CECA2_CATS** | **KIGKKIERVGQHHGVLVVGYG** |  | **CATS** | **HGVLVVGYG** |  | **KIGKKIERVGQH** | **CATS_CECA2** | **HGVLVVGYGKIGKKIERVGQH** |
|  | **KIGKKIERVGQH** | **CATH1** | **PVKNQGACGSCWTFS** | **CECA2_CATH1** | **KIGKKIERVGQHPVKNQGACGSCWTFS** |  | **CATH1** | **PVKNQGACGSCWTFS** |  | **KIGKKIERVGQH** | **CATH1_CECA2** | **PVKNQGACGSCWTFSKIGKKIERVGQH** |
|  | **KIGKKIERVGQH** | **CATL1** | **PVKNQGQCGSCWAFS** | **CECA2_CATL1** | **KIGKKIERVGQHPVKNQGQCGSCWAFS** |  | **CATL1** | **PVKNQGQCGSCWAFS** |  | **KIGKKIERVGQH** | **CATL1_CECA2** | **PVKNQGQCGSCWAFSKIGKKIERVGQH** |
|  | **KIGKKIERVGQH** | **CATH2** | **YWIVKNSWG** | **CECA2_CATH2** | **KIGKKIERVGQHYWIVKNSWG** |  | **CATH2** | **YWIVKNSWG** |  | **KIGKKIERVGQH** | **CATH2_CECA2** | **YWIVKNSWGKIGKKIERVGQH** |
|  | **KIGKKIERVGQH** | **CATL1.2** | **YWLVKNSWG** | **CECA2_CATL1.2** | **KIGKKIERVGQHYWLVKNSWG** |  | **CATL1.2** | **YWLVKNSWG** |  | **KIGKKIERVGQH** | **CATL1.2_CECA2** | **YWLVKNSWGKIGKKIERVGQH** |
| **CECA3** | **KVFKKIEKVGRN** | **CATK** | **HAVLVVGYG** | **CECA3_CATK** | **KVFKKIEKVGRNHAVLVVGYG** |  | **CATK** | **HAVLVVGYG** | **CECA3** | **KVFKKIEKVGRN** | **CATK_CECA3** | **HAVLVVGYGKVFKKIEKVGRN** |
|  | **KVFKKIEKVGRN** | **CATS** | **HGVLVVGYG** | **CECA3_CATS** | **KVFKKIEKVGRNHGVLVVGYG** |  | **CATS** | **HGVLVVGYG** |  | **KVFKKIEKVGRN** | **CATS_CECA3** | **HGVLVVGYGKVFKKIEKVGRN** |
|  | **KVFKKIEKVGRN** | **CATH1** | **PVKNQGACGSCWTFS** | **CECA3_CATH1** | **KVFKKIEKVGRNPVKNQGACGSCWTFS** |  | **CATH1** | **PVKNQGACGSCWTFS** |  | **KVFKKIEKVGRN** | **CATH1_CECA3** | **PVKNQGACGSCWTFSKVFKKIEKVGRN** |
|  | **KVFKKIEKVGRN** | **CATL1** | **PVKNQGQCGSCWAFS** | **CECA3_CATL1** | **KVFKKIEKVGRNPVKNQGQCGSCWAFS** |  | **CATL1** | **PVKNQGQCGSCWAFS** |  | **KVFKKIEKVGRN** | **CATL1_CECA3** | **PVKNQGQCGSCWAFSKVFKKIEKVGRN** |
|  | **KVFKKIEKVGRN** | **CATH2** | **YWIVKNSWG** | **CECA3_CATH2** | **KVFKKIEKVGRNYWIVKNSWG** |  | **CATH2** | **YWIVKNSWG** |  | **KVFKKIEKVGRN** | **CATH2_CECA3** | **YWIVKNSWGKVFKKIEKVGRN** |
|  | **KVFKKIEKVGRN** | **CATL1.2** | **YWLVKNSWG** | **CECA3_CATL1.2** | **KVFKKIEKVGRNYWLVKNSWG** |  | **CATL1.2** | **YWLVKNSWG** |  | **KVFKKIEKVGRN** | **CATL1.2_CECA3** | **YWLVKNSWGKVFKKIEKVGRN** |
| **CECA4** | **KFGKKLEGVGKR** | **CATK** | **HAVLVVGYG** | **CECA4_CATK** | **KFGKKLEGVGKRHAVLVVGYG** |  | **CATK** | **HAVLVVGYG** | **CECA4** | **KFGKKLEGVGKR** | **CATK_CECA4** | **HAVLVVGYGKFGKKLEGVGKR** |
|  | **KFGKKLEGVGKR** | **CATS** | **HGVLVVGYG** | **CECA4_CATS** | **KFGKKLEGVGKRHGVLVVGYG** |  | **CATS** | **HGVLVVGYG** |  | **KFGKKLEGVGKR** | **CATS_CECA4** | **HGVLVVGYGKFGKKLEGVGKR** |
|  | **KFGKKLEGVGKR** | **CATH1** | **PVKNQGACGSCWTFS** | **CECA4_CATH1** | **KFGKKLEGVGKRPVKNQGACGSCWTFS** |  | **CATH1** | **PVKNQGACGSCWTFS** |  | **KFGKKLEGVGKR** | **CATH1_CECA4** | **PVKNQGACGSCWTFSKFGKKLEGVGKR** |
|  | **KFGKKLEGVGKR** | **CATL1** | **PVKNQGQCGSCWAFS** | **CECA4_CATL1** | **KFGKKLEGVGKRPVKNQGQCGSCWAFS** |  | **CATL1** | **PVKNQGQCGSCWAFS** |  | **KFGKKLEGVGKR** | **CATL1_CECA4** | **PVKNQGQCGSCWAFSKFGKKLEGVGKR** |
|  | **KFGKKLEGVGKR** | **CATH2** | **YWIVKNSWG** | **CECA4_CATH2** | **KFGKKLEGVGKRYWIVKNSWG** |  | **CATH2** | **YWIVKNSWG** |  | **KFGKKLEGVGKR** | **CATH2_CECA4** | **YWIVKNSWGKFGKKLEGVGKR** |
|  | **KFGKKLEGVGKR** | **CATL1.2** | **YWLVKNSWG** | **CECA4_CATL1.2** | **KFGKKLEGVGKRYWLVKNSWG** |  | **CATL1.2** | **YWLVKNSWG** |  | **KFGKKLEGVGKR** | **CATL1.2_CECA4** | **YWLVKNSWGKFGKKLEGVGKR** |
| **CECB1** | **KLGKKIE** | **CATK** | **HAVLVVGYG** | **CECB1_CATK** | **KLGKKIEHAVLVVGYG** |  | **CATK** | **HAVLVVGYG** | **CECB1** | **KLGKKIE** | **CATK_CECB1** | **HAVLVVGYGKLGKKIE** |
|  | **KLGKKIE** | **CATS** | **HGVLVVGYG** | **CECB1_CATS** | **KLGKKIEHGVLVVGYG** |  | **CATS** | **HGVLVVGYG** |  | **KLGKKIE** | **CATS_CECB1** | **HGVLVVGYGKLGKKIE** |
|  | **KLGKKIE** | **CATH1** | **PVKNQGACGSCWTFS** | **CECB1_CATH1** | **KLGKKIEPVKNQGACGSCWTFS** |  | **CATH1** | **PVKNQGACGSCWTFS** |  | **KLGKKIE** | **CATH1_CECB1** | **PVKNQGACGSCWTFSKLGKKIE** |
|  | **KLGKKIE** | **CATL1** | **PVKNQGQCGSCWAFS** | **CECB1_CATL1** | **KLGKKIEPVKNQGQCGSCWAFS** |  | **CATL1** | **PVKNQGQCGSCWAFS** |  | **KLGKKIE** | **CATL1_CECB1** | **PVKNQGQCGSCWAFSKLGKKIE** |
|  | **KLGKKIE** | **CATH2** | **YWIVKNSWG** | **CECB1_CATH2** | **KLGKKIEYWIVKNSWG** |  | **CATH2** | **YWIVKNSWG** |  | **KLGKKIE** | **CATH2_CECB1** | **YWIVKNSWGKLGKKIE** |
|  | **KLGKKIE** | **CATL1.2** | **YWLVKNSWG** | **CECB1_CATL1.2** | **KLGKKIEYWLVKNSWG** |  | **CATL1.2** | **YWLVKNSWG** |  | **KLGKKIE** | **CATL1.2_CECB1** | **YWLVKNSWGKLGKKIE** |
| **CECB2** | **KIFKKIE** | **CATK** | **HAVLVVGYG** | **CECB2_CATK** | **KIFKKIEHAVLVVGYG** |  | **CATK** | **HAVLVVGYG** | **CECB2** | **KIFKKIE** | **CATK_CECB2** | **HAVLVVGYGKIFKKIE** |
|  | **KIFKKIE** | **CATS** | **HGVLVVGYG** | **CECB2_CATS** | **KIFKKIEHGVLVVGYG** |  | **CATS** | **HGVLVVGYG** |  | **KIFKKIE** | **CATS_CECB2** | **HGVLVVGYGKIFKKIE** |
|  | **KIFKKIE** | **CATH1** | **PVKNQGACGSCWTFS** | **CECB2_CATH1** | **KIFKKIEPVKNQGACGSCWTFS** |  | **CATH1** | **PVKNQGACGSCWTFS** |  | **KIFKKIE** | **CATH1_CECB2** | **PVKNQGACGSCWTFSKIFKKIE** |
|  | **KIFKKIE** | **CATL1** | **PVKNQGQCGSCWAFS** | **CECB2_CATL1** | **KIFKKIEPVKNQGQCGSCWAFS** |  | **CATL1** | **PVKNQGQCGSCWAFS** |  | **KIFKKIE** | **CATL1_CECB2** | **PVKNQGQCGSCWAFSKIFKKIE** |
|  | **KIFKKIE** | **CATH2** | **YWIVKNSWG** | **CECB2_CATH2** | **KIFKKIEYWIVKNSWG** |  | **CATH2** | **YWIVKNSWG** |  | **KIFKKIE** | **CATH2_CECB2** | **YWIVKNSWGKIFKKIE** |
|  | **KIFKKIE** | **CATL1.2** | **YWLVKNSWG** | **CECB2_CATL1.2** | **KIFKKIEYWLVKNSWG** |  | **CATL1.2** | **YWLVKNSWG** |  | **KIFKKIE** | **CATL1.2_CECB2** | **YWLVKNSWGKIFKKIE** |
| **CECB3** | **KVFKKIE** | **CATK** | **HAVLVVGYG** | **CECB3_CATK** | **KVFKKIEHAVLVVGYG** |  | **CATK** | **HAVLVVGYG** | **CECB3** | **KVFKKIE** | **CATK_CECB3** | **HAVLVVGYGKVFKKIE** |
|  | **KVFKKIE** | **CATS** | **HGVLVVGYG** | **CECB3_CATS** | **KVFKKIEHGVLVVGYG** |  | **CATS** | **HGVLVVGYG** |  | **KVFKKIE** | **CATS_CECB3** | **HGVLVVGYGKVFKKIE** |
|  | **KVFKKIE** | **CATH1** | **PVKNQGACGSCWTFS** | **CECB3_CATH1** | **KVFKKIEPVKNQGACGSCWTFS** |  | **CATH1** | **PVKNQGACGSCWTFS** |  | **KVFKKIE** | **CATH1_CECB3** | **PVKNQGACGSCWTFSKVFKKIE** |
|  | **KVFKKIE** | **CATL1** | **PVKNQGQCGSCWAFS** | **CECB3_CATL1** | **KVFKKIEPVKNQGQCGSCWAFS** |  | **CATL1** | **PVKNQGQCGSCWAFS** |  | **KVFKKIE** | **CATL1_CECB3** | **PVKNQGQCGSCWAFSKVFKKIE** |
|  | **KVFKKIE** | **CATH2** | **YWIVKNSWG** | **CECB3_CATH2** | **KVFKKIEYWIVKNSWG** |  | **CATH2** | **YWIVKNSWG** |  | **KVFKKIE** | **CATH2_CECB3** | **YWIVKNSWGKVFKKIE** |
|  | **KVFKKIE** | **CATL1.2** | **YWLVKNSWG** | **CECB3_CATL1.2** | **KVFKKIEYWLVKNSWG** |  | **CATL1.2** | **YWLVKNSWG** |  | **KVFKKIE** | **CATL1.2_CECB3** | **YWLVKNSWGKVFKKIE** |
| **CECC1** | **KLGKRIERIGQ** | **CATK** | **HAVLVVGYG** | **CECC1_CATK** | **KLGKRIERIGQHAVLVVGYG** |  | **CATK** | **HAVLVVGYG** | **CECC1** | **KLGKRIERIGQ** | **CATK_CECC1** | **HAVLVVGYGKLGKRIERIGQ** |
|  | **KLGKRIERIGQ** | **CATS** | **HGVLVVGYG** | **CECC1_CATS** | **KLGKRIERIGQHGVLVVGYG** |  | **CATS** | **HGVLVVGYG** |  | **KLGKRIERIGQ** | **CATS_CECC1** | **HGVLVVGYGKLGKRIERIGQ** |
|  | **KLGKRIERIGQ** | **CATH1** | **PVKNQGACGSCWTFS** | **CECC1_CATH1** | **KLGKRIERIGQPVKNQGACGSCWTFS** |  | **CATH1** | **PVKNQGACGSCWTFS** |  | **KLGKRIERIGQ** | **CATH1_CECC1** | **PVKNQGACGSCWTFSKLGKRIERIGQ** |
|  | **KLGKRIERIGQ** | **CATL1** | **PVKNQGQCGSCWAFS** | **CECC1_CATL1** | **KLGKRIERIGQPVKNQGQCGSCWAFS** |  | **CATL1** | **PVKNQGQCGSCWAFS** |  | **KLGKRIERIGQ** | **CATL1_CECC1** | **PVKNQGQCGSCWAFSKLGKRIERIGQ** |
|  | **KLGKRIERIGQ** | **CATH2** | **YWIVKNSWG** | **CECC1_CATH2** | **KLGKRIERIGQYWIVKNSWG** |  | **CATH2** | **YWIVKNSWG** |  | **KLGKRIERIGQ** | **CATH2_CECC1** | **YWIVKNSWGKLGKRIERIGQ** |
|  | **KLGKRIERIGQ** | **CATL1.2** | **YWLVKNSWG** | **CECC1_CATL1.2** | **KLGKRIERIGQYWLVKNSWG** |  | **CATL1.2** | **YWLVKNSWG** |  | **KLGKRIERIGQ** | **CATL1.2_CECC1** | **YWLVKNSWGKLGKRIERIGQ** |
| **CECC2** | **KLGKKLEGAGK** | **CATK** | **HAVLVVGYG** | **CECC2_CATK** | **KLGKKLEGAGKHAVLVVGYG** |  | **CATK** | **HAVLVVGYG** | **CECC2** | **KLGKKLEGAGK** | **CATK_CECC2** | **HAVLVVGYGKLGKKLEGAGK** |
|  | **KLGKKLEGAGK** | **CATS** | **HGVLVVGYG** | **CECC2_CATS** | **KLGKKLEGAGKHGVLVVGYG** |  | **CATS** | **HGVLVVGYG** |  | **KLGKKLEGAGK** | **CATS_CECC2** | **HGVLVVGYGKLGKKLEGAGK** |
|  | **KLGKKLEGAGK** | **CATH1** | **PVKNQGACGSCWTFS** | **CECC2_CATH1** | **KLGKKLEGAGKPVKNQGACGSCWTFS** |  | **CATH1** | **PVKNQGACGSCWTFS** |  | **KLGKKLEGAGK** | **CATH1_CECC2** | **PVKNQGACGSCWTFSKLGKKLEGAGK** |
|  | **KLGKKLEGAGK** | **CATL1** | **PVKNQGQCGSCWAFS** | **CECC2_CATL1** | **KLGKKLEGAGKPVKNQGQCGSCWAFS** |  | **CATL1** | **PVKNQGQCGSCWAFS** |  | **KLGKKLEGAGK** | **CATL1_CECC2** | **PVKNQGQCGSCWAFSKLGKKLEGAGK** |
|  | **KLGKKLEGAGK** | **CATH2** | **YWIVKNSWG** | **CECC2_CATH2** | **KLGKKLEGAGKYWIVKNSWG** |  | **CATH2** | **YWIVKNSWG** |  | **KLGKKLEGAGK** | **CATH2_CECC2** | **YWIVKNSWGKLGKKLEGAGK** |
|  | **KLGKKLEGAGK** | **CATL1.2** | **YWLVKNSWG** | **CECC2_CATL1.2** | **KLGKKLEGAGKYWLVKNSWG** |  | **CATL1.2** | **YWLVKNSWG** |  | **KLGKKLEGAGK** | **CATL1.2_CECC2** | **YWLVKNSWGKLGKKLEGAGK** |
| **CECD1** | **GQRIRDAIISAAPAV** | **CATK** | **HAVLVVGYG** | **CECD1_CATK** | **GQRIRDAIISAAPAVHAVLVVGYG** |  | **CATK** | **HAVLVVGYG** | **CECD1** | **GQRIRDAIISAAPAV** | **CATK_CECD1** | **HAVLVVGYGGQRIRDAIISAAPAV** |
|  | **GQRIRDAIISAAPAV** | **CATS** | **HGVLVVGYG** | **CECD1_CATS** | **GQRIRDAIISAAPAVHGVLVVGYG** |  | **CATS** | **HGVLVVGYG** |  | **GQRIRDAIISAAPAV** | **CATS_CECD1** | **HGVLVVGYGGQRIRDAIISAAPAV** |
|  | **GQRIRDAIISAAPAV** | **CATH1** | **PVKNQGACGSCWTFS** | **CECD1_CATH1** | **GQRIRDAIISAAPAVPVKNQGACGSCWTFS** |  | **CATH1** | **PVKNQGACGSCWTFS** |  | **GQRIRDAIISAAPAV** | **CATH1_CECD1** | **PVKNQGACGSCWTFSGQRIRDAIISAAPAV** |
|  | **GQRIRDAIISAAPAV** | **CATL1** | **PVKNQGQCGSCWAFS** | **CECD1_CATL1** | **GQRIRDAIISAAPAVPVKNQGQCGSCWAFS** |  | **CATL1** | **PVKNQGQCGSCWAFS** |  | **GQRIRDAIISAAPAV** | **CATL1_CECD1** | **PVKNQGQCGSCWAFSGQRIRDAIISAAPAV** |
|  | **GQRIRDAIISAAPAV** | **CATH2** | **YWIVKNSWG** | **CECD1_CATH2** | **GQRIRDAIISAAPAVYWIVKNSWG** |  | **CATH2** | **YWIVKNSWG** |  | **GQRIRDAIISAAPAV** | **CATH2_CECD1** | **YWIVKNSWGGQRIRDAIISAAPAV** |
|  | **GQRIRDAIISAAPAV** | **CATL1.2** | **YWLVKNSWG** | **CECD1_CATL1.2** | **GQRIRDAIISAAPAVYWLVKNSWG** |  | **CATL1.2** | **YWLVKNSWG** |  | **GQRIRDAIISAAPAV** | **CATL1.2_CECD1** | **YWLVKNSWGGQRIRDAIISAAPAV** |
| **CECD2** | **GQRVRDAVISAGPAV** | **CATK** | **HAVLVVGYG** | **CECD2_CATK** | **GQRVRDAVISAGPAVHAVLVVGYG** |  | **CATK** | **HAVLVVGYG** | **CECD2** | **GQRVRDAVISAGPAV** | **CATK_CECD2** | **HAVLVVGYGGQRVRDAVISAGPAV** |
|  | **GQRVRDAVISAGPAV** | **CATS** | **HGVLVVGYG** | **CECD2_CATS** | **GQRVRDAVISAGPAVHGVLVVGYG** |  | **CATS** | **HGVLVVGYG** |  | **GQRVRDAVISAGPAV** | **CATS_CECD2** | **HGVLVVGYGGQRVRDAVISAGPAV** |
|  | **GQRVRDAVISAGPAV** | **CATH1** | **PVKNQGACGSCWTFS** | **CECD2_CATH1** | **GQRVRDAVISAGPAVPVKNQGACGSCWTFS** |  | **CATH1** | **PVKNQGACGSCWTFS** |  | **GQRVRDAVISAGPAV** | **CATH1_CECD2** | **PVKNQGACGSCWTFSGQRVRDAVISAGPAV** |
|  | **GQRVRDAVISAGPAV** | **CATL1** | **PVKNQGQCGSCWAFS** | **CECD2_CATL1** | **GQRVRDAVISAGPAVPVKNQGQCGSCWAFS** |  | **CATL1** | **PVKNQGQCGSCWAFS** |  | **GQRVRDAVISAGPAV** | **CATL1_CECD2** | **PVKNQGQCGSCWAFSGQRVRDAVISAGPAV** |
|  | **GQRVRDAVISAGPAV** | **CATH2** | **YWIVKNSWG** | **CECD2_CATH2** | **GQRVRDAVISAGPAVYWIVKNSWG** |  | **CATH2** | **YWIVKNSWG** |  | **GQRVRDAVISAGPAV** | **CATH2_CECD2** | **YWIVKNSWGGQRVRDAVISAGPAV** |
|  | **GQRVRDAVISAGPAV** | **CATL1.2** | **YWLVKNSWG** | **CECD2_CATL1.2** | **GQRVRDAVISAGPAVYWLVKNSWG** |  | **CATL1.2** | **YWLVKNSWG** |  | **GQRVRDAVISAGPAV** | **CATL1.2_CECD2** | **YWLVKNSWGGQRVRDAVISAGPAV** |
| **CECD3** | **GQRVRDAIISAGPAV** | **CATK** | **HAVLVVGYG** | **CECD3_CATK** | **GQRVRDAIISAGPAVHAVLVVGYG** |  | **CATK** | **HAVLVVGYG** | **CECD3** | **GQRVRDAIISAGPAV** | **CATK_CECD3** | **HAVLVVGYGGQRVRDAIISAGPAV** |
|  | **GQRVRDAIISAGPAV** | **CATS** | **HGVLVVGYG** | **CECD3_CATS** | **GQRVRDAIISAGPAVHGVLVVGYG** |  | **CATS** | **HGVLVVGYG** |  | **GQRVRDAIISAGPAV** | **CATS_CECD3** | **HGVLVVGYGGQRVRDAIISAGPAV** |
|  | **GQRVRDAIISAGPAV** | **CATH1** | **PVKNQGACGSCWTFS** | **CECD3_CATH1** | **GQRVRDAIISAGPAVPVKNQGACGSCWTFS** |  | **CATH1** | **PVKNQGACGSCWTFS** |  | **GQRVRDAIISAGPAV** | **CATH1_CECD3** | **PVKNQGACGSCWTFSGQRVRDAIISAGPAV** |
|  | **GQRVRDAIISAGPAV** | **CATL1** | **PVKNQGQCGSCWAFS** | **CECD3_CATL1** | **GQRVRDAIISAGPAVPVKNQGQCGSCWAFS** |  | **CATL1** | **PVKNQGQCGSCWAFS** |  | **GQRVRDAIISAGPAV** | **CATL1_CECD3** | **PVKNQGQCGSCWAFSGQRVRDAIISAGPAV** |
|  | **GQRVRDAIISAGPAV** | **CATH2** | **YWIVKNSWG** | **CECD3_CATH2** | **GQRVRDAIISAGPAVYWIVKNSWG** |  | **CATH2** | **YWIVKNSWG** |  | **GQRVRDAIISAGPAV** | **CATH2_CECD3** | **YWIVKNSWGGQRVRDAIISAGPAV** |
|  | **GQRVRDAIISAGPAV** | **CATL1.2** | **YWLVKNSWG** | **CECD3_CATL1.2** | **GQRVRDAIISAGPAVYWLVKNSWG** |  | **CATL1.2** | **YWLVKNSWG** |  | **GQRVRDAIISAGPAV** | **CATL1.2_CECD3** | **YWLVKNSWGGQRVRDAIISAGPAV** |

**Table S3 - CPP and AMP prediction**

|  |  |  | **PROTPARAM** | **CellPPD** | **C2 Pred** | | **CAMP** | **AMPA** | |
| --- | --- | --- | --- | --- | --- | --- | --- | --- | --- |
|  | **NAME** | **HYBRID PEPTIDE** | **N° AMINO ACID** | **PREDICTION CPP** | | | **PREDICTION AMP** | | |
| **CECROPIN N TERMINAL** | **CEC1_CATK** | **KKIERVGQNTRDAHAVLVVGYG** | **22** | **N-CPP** | **N-CPP** | **0,163254** | **0,604** | **-** | **None** |
|  | **CEC1_CATS** | **KKIERVGQNTRDAHGVLVVGYG** | **22** | **N-CPP** | **N-CPP** | **0,170956** | **0,611** | **-** | **None** |
|  | **CEC1_CATH1** | **KKIERVGQNTRDAPVKNQGACGSCWTFS** | **28** | **N-CPP** | **N-CPP** | **0,344028** | **0,6485** | **-** | **None** |
|  | **CEC1_CATL1** | **KKIERVGQNTRDAPVKNQGQCGSCWAFS** | **28** | **N-CPP** | **N-CPP** | **0,40466** | **0,653** | **-** | **None** |
|  | **CEC1_CATH2** | **KKIERVGQNTRDAYWIVKNSWG** | **22** | **N-CPP** | **N-CPP** | **0,388872** | **0,7795** | **-** | **None** |
|  | **CEC1_CATL1.2** | **KKIERVGQNTRDAYWLVKNSWG** | **22** | **N-CPP** | **CPP** | **0,548823** | **0,747** | **-** | **None** |
|  | **CEC2_CATK** | **KKIEKAVRRVRDGHAVLVVGYG** | **22** | **N-CPP** | **N-CPP** | **0,050031** | **0,802** | **KIEKAVRRVRDGH** | **0,15** |
|  | **CEC2_CATS** | **KKIEKAVRRVRDGHGVLVVGYG** | **22** | **N-CPP** | **N-CPP** | **0,045587** | **0,792** | **KIEKAVRRVRDGH** | **0,14** |
|  | **CEC2_CATH1** | **KKIEKAVRRVRDGPVKNQGACGSCWTFS** | **28** | **N-CPP** | **N-CPP** | **0,15708** | **0,838** | **KIEKAVRRVRDG** | **0,14** |
|  | **CEC2_CATL1** | **KKIEKAVRRVRDGPVKNQGQCGSCWAFS** | **28** | **N-CPP** | **N-CPP** | **0,177573** | **0,823** | **KIEKAVRRVRDG** | **0,14** |
|  | **CEC2_CATH2** | **KKIEKAVRRVRDGYWIVKNSWG** | **22** | **N-CPP** | **N-CPP** | **0,163295** | **0,965** | **KIEKAVRRVRDGYW** | **0,11** |
|  | **CEC2_CATL.2** | **KKIEKAVRRVRDGYWLVKNSWG** | **22** | **N-CPP** | **N-CPP** | **0,269049** | **0,963** | **KIEKAVRRVRDGYW** | **0,12** |
|  | **CEC3_CATK** | **KKIEKVGQNIRDGHAVLVVGYG** | **22** | **N-CPP** | **N-CPP** | **0,052893** | **0,729** | **-** | **None** |
|  | **CEC3_CATS** | **KKIEKVGQNIRDGHGVLVVGYG** | **22** | **N-CPP** | **N-CPP** | **0,056891** | **0,7315** | **-** | **None** |
|  | **CEC3_CATH1** | **KKIEKVGQNIRDGPVKNQGACGSCWTFS** | **28** | **N-CPP** | **N-CPP** | **0,187981** | **0,78** | **-** | **None** |
|  | **CEC3_CATL1** | **KKIEKVGQNIRDGPVKNQGQCGSCWAFS** | **28** | **N-CPP** | **N-CPP** | **0,2127** | **0,759** | **-** | **None** |
|  | **CEC3_CATH2** | **KKIEKVGQNIRDGYWIVKNSWG** | **22** | **N-CPP** | **N-CPP** | **0,177917** | **0,891** | **-** | **None** |
|  | **CEC3_CATL1.2** | **KKIEKVGQNIRDGYWLVKNSWG** | **22** | **N-CPP** | **N-CPP** | **0,238066** | **0,892** | **-** | **None** |
|  | **CEC4_CATK** | **KKIGRVGQHTRDAHAVLVVGYG** | **22** | **N-CPP** | **N-CPP** | **0,166423** | **0,9015** | **-** | **None** |
|  | **CEC4_CATS** | **KKIGRVGQHTRDAHGVLVVGYG** | **22** | **N-CPP** | **N-CPP** | **0,172981** | **0,8965** | **-** | **None** |
|  | **CEC4_CATH1** | **KKIGRVGQHTRDAPVKNQGACGSCWTFS** | **28** | **N-CPP** | **N-CPP** | **0,223645** | **0,896** | **-** | **None** |
|  | **CEC4_CATL1** | **KKIGRVGQHTRDAPVKNQGQCGSCWAFS** | **28** | **N-CPP** | **N-CPP** | **0,296512** | **0,881** | **-** | **None** |
|  | **CEC4_CATH2** | **KKIGRVGQHTRDAYWIVKNSWG** | **22** | **N-CPP** | **N-CPP** | **0,299454** | **0,898** | **-** | **None** |
|  | **CEC4_CATL1.2** | **KKIGRVGQHTRDAYWLVKNSWG** | **22** | **N-CPP** | **N-CPP** | **0,437945** | **0,8835** | **-** | **None** |
|  | **CECA1_CATK** | **KLGKKLEGAGKRHAVLVVGYG** | **21** | **N-CPP** | **N-CPP** | **0,085642** | **0,9245** | **-** | **None** |
|  | **CECA1_CATS** | **KLGKKLEGAGKRHGVLVVGYG** | **21** | **N-CPP** | **N-CPP** | **0,090973** | **0,913** | **-** | **None** |
|  | **CECA1_CATH1** | **KLGKKLEGAGKRPVKNQGACGSCWTFS** | **27** | **N-CPP** | **N-CPP** | **0,162218** | **0,926** | **-** | **None** |
|  | **CECA1_CATL1** | **KLGKKLEGAGKRPVKNQGQCGSCWAFS** | **27** | **N-CPP** | **N-CPP** | **0,157737** | **0,9165** | **-** | **None** |
|  | **CECA1_CATH2** | **KLGKKLEGAGKRYWIVKNSWG** | **21** | **N-CPP** | **N-CPP** | **0,219563** | **0,959** | **AGKRYWIVKNSW** | **0** |
|  | **CECA1_CATL1.2** | **KLGKKLEGAGKRYWLVKNSWG** | **21** | **N-CPP** | **N-CPP** | **0,269012** | **0,954** | **AGKRYWLVKNSW** | **0** |
|  | **CECA2_CATK** | **KIGKKIERVGQHHAVLVVGYG** | **21** | **N-CPP** | **N-CPP** | **0,104935** | **0,8115** | **-** | **None** |
|  | **CECA2_CATS** | **KIGKKIERVGQHHGVLVVGYG** | **21** | **N-CPP** | **N-CPP** | **0,109176** | **0,8135** | **-** | **None** |
|  | **CECA2_CATH1** | **KIGKKIERVGQHPVKNQGACGSCWTFS** | **27** | **N-CPP** | **N-CPP** | **0,176366** | **0,954** | **IGKKIERVGQHP** | **0,13** |
|  | **CECA2_CATL1** | **KIGKKIERVGQHPVKNQGQCGSCWAFS** | **27** | **N-CPP** | **N-CPP** | **0,247446** | **0,936** | **IGKKIERVGQHP** | **0,13** |
|  | **CECA2_CATH2** | **KIGKKIERVGQHYWIVKNSWG** | **21** | **N-CPP** | **N-CPP** | **0,261233** | **0,9355** | **KIGKKIERVGQHYWIVKNSW** | **0,04** |
|  | **CECA2_CATL1.2** | **KIGKKIERVGQHYWLVKNSWG** | **21** | **N-CPP** | **N-CPP** | **0,378201** | **0,9395** | **KIGKKIERVGQHYWLVKNSW** | **0,05** |
|  | **CECA3_CATK** | **KVFKKIEKVGRNHAVLVVGYG** | **21** | **N-CPP** | **N-CPP** | **0,065151** | **0,9335** | **VFKKIEKVGRNHAVLV** | **0,07** |
|  | **CECA3_CATS** | **KVFKKIEKVGRNHGVLVVGYG** | **21** | **N-CPP** | **N-CPP** | **0,070169** | **0,931** |  |  |
|  | **CECA3_CATH1** | **KVFKKIEKVGRNPVKNQGACGSCWTFS** | **27** | **N-CPP** | **N-CPP** | **0,197573** | **0,991** | **VFKKIEKVGRNPVKN** | **0,08** |
|  | **CECA3_CATL1** | **KVFKKIEKVGRNPVKNQGQCGSCWAFS** | **27** | **N-CPP** | **N-CPP** | **0,213682** | **0,9895** | **VFKKIEKVGRNPVKN** | **0,07** |
|  | **CECA3_CATH2** | **KVFKKIEKVGRNYWIVKNSWG** | **21** | **N-CPP** | **N-CPP** | **0,193971** | **0,948** | **-** | **None** |
|  | **CECA3_CATL1.2** | **KVFKKIEKVGRNYWLVKNSWG** | **21** | **N-CPP** | **N-CPP** | **0,266868** | **0,95** | **-** | **None** |
|  | **CECA4_CATK** | **KFGKKLEGVGKRHAVLVVGYG** | **21** | **N-CPP** | **N-CPP** | **0,057452** | **0,9265** | **-** | **None** |
|  | **CECA4_CATS** | **KFGKKLEGVGKRHGVLVVGYG** | **21** | **N-CPP** | **N-CPP** | **0,068722** | **0,917** | **-** | **None** |
|  | **CECA4_CATH1** | **KFGKKLEGVGKRPVKNQGACGSCWTFS** | **27** | **N-CPP** | **N-CPP** | **0,121489** | **0,955** | **-** | **None** |
|  | **CECA4_CATL1** | **KFGKKLEGVGKRPVKNQGQCGSCWAFS** | **27** | **N-CPP** | **N-CPP** | **0,146847** | **0,9385** | **-** | **None** |
|  | **CECA4_CATH2** | **KFGKKLEGVGKRYWIVKNSWG** | **21** | **N-CPP** | **N-CPP** | **0,149415** | **0,961** | **VGKRYWIVKNSW** | **0** |
|  | **CECA4_CATL1.2** | **KFGKKLEGVGKRYWLVKNSWG** | **21** | **N-CPP** | **N-CPP** | **0,199268** | **0,9585** | **VGKRYWLVKNSW** | **0** |
|  | **CECB1_CATK** | **KLGKKIEHAVLVVGYG** | **16** | **N-CPP** | **N-CPP** | **0,076305** | **0,576** | **-** | **None** |
|  | **CECB1_CATS** | **KLGKKIEHGVLVVGYG** | **16** | **N-CPP** | **N-CPP** | **0,079039** | **0,577** | **-** | **None** |
|  | **CECB1_CATH1** | **KLGKKIEPVKNQGACGSCWTFS** | **22** | **N-CPP** | **N-CPP** | **0,219404** | **0,9815** | **-** | **None** |
|  | **CECB1_CATL1** | **KLGKKIEPVKNQGQCGSCWAFS** | **22** | **N-CPP** | **N-CPP** | **0,257045** | **0,981** | **-** | **None** |
|  | **CECB1_CATH2** | **KLGKKIEYWIVKNSWG** | **16** | **N-CPP** | **N-CPP** | **0,323416** | **0,862** | **-** | **None** |
|  | **CECB1_CATL1.2** | **KLGKKIEYWLVKNSWG** | **16** | **N-CPP** | **N-CPP** | **0,492165** | **0,855** |  |  |
|  | **CECB2_CATK** | **KIFKKIEHAVLVVGYG** | **16** | **N-CPP** | **N-CPP** | **0,097911** | **0,5805** | **-** | **None** |
|  | **CECB2_CATS** | **KIFKKIEHGVLVVGYG** | **16** | **N-CPP** | **N-CPP** | **0,100622** | **0,6** | **-** | **None** |
|  | **CECB2_CATH1** | **KIFKKIEPVKNQGACGSCWTFS** | **22** | **N-CPP** | **N-CPP** | **0,257303** | **0,99** | **-** | **None** |
|  | **CECB2_CATL1** | **KIFKKIEPVKNQGQCGSCWAFS** | **22** | **N-CPP** | **N-CPP** | **0,320251** | **0,9895** | **-** | **None** |
|  | **CECB2_CATH2** | **KIFKKIEYWIVKNSWG** | **16** | **N-CPP** | **N-CPP** | **0,375959** | **0,8435** | **-** | **None** |
|  | **CECB2_CATL1.2** | **KIFKKIEYWLVKNSWG** | **16** | **N-CPP** | **CPP** | **0,589472** | **0,843** |  |  |
|  | **CECB3_CATK** | **KVFKKIEHAVLVVGYG** | **16** | **N-CPP** | **N-CPP** | **0,069281** | **0,5715** | **-** | **None** |
|  | **CECB3_CATS** | **KVFKKIEHGVLVVGYG** | **16** | **N-CPP** | **N-CPP** | **0,07486** | **0,5765** | **-** | **None** |
|  | **CECB3_CATH1** | **KVFKKIEPVKNQGACGSCWTFS** | **22** | **N-CPP** | **N-CPP** | **0,191852** | **0,989** | **-** | **None** |
|  | **CECB3_CATL1** | **KVFKKIEPVKNQGQCGSCWAFS** | **22** | **N-CPP** | **N-CPP** | **0,218518** | **0,988** | **-** | **None** |
|  | **CECB3_CATH2** | **KVFKKIEYWIVKNSWG** | **16** | **N-CPP** | **N-CPP** | **0,244385** | **0,839** | **-** | **None** |
|  | **CECB3_CATL1.2** | **KVFKKIEYWLVKNSWG** | **16** | **N-CPP** | **N-CPP** | **0,379286** | **0,835** |  |  |
|  | **CECC1_CATK** | **KLGKRIERIGQHAVLVVGYG** | **20** | **N-CPP** | **N-CPP** | **0,135352** | **0,7765** | **-** | **None** |
|  | **CECC1_CATS** | **KLGKRIERIGQHGVLVVGYG** | **20** | **N-CPP** | **N-CPP** | **0,130612** | **0,7805** | **-** | **None** |
|  | **CECC1_CATH1** | **KLGKRIERIGQPVKNQGACGSCWTFS** | **26** | **N-CPP** | **N-CPP** | **0,158409** | **0,9865** | **-** | **None** |
|  | **CECC1_CATL1** | **KLGKRIERIGQPVKNQGQCGSCWAFS** | **26** | **N-CPP** | **N-CPP** | **0,208509** | **0,96** | **-** | **None** |
|  | **CECC1_CATH2** | **KLGKRIERIGQYWIVKNSWG** | **20** | **N-CPP** | **N-CPP** | **0,266102** | **0,9455** | **KLGKRIERIGQYWIVKNSW** | **0,05** |
|  | **CECC1_CATL1.2** | **KLGKRIERIGQYWLVKNSWG** | **20** | **N-CPP** | **N-CPP** | **0,376259** | **0,948** | **KLGKRIERIGQYWLVKNSW** | **0,07** |
|  | **CECC2_CATK** | **KLGKKLEGAGKHAVLVVGYG** | **20** | **N-CPP** | **N-CPP** | **0,04773** | **0,9175** | **-** | **None** |
|  | **CECC2_CATS** | **KLGKKLEGAGKHGVLVVGYG** | **20** | **N-CPP** | **N-CPP** | **0,05241** | **0,912** | **-** | **None** |
|  | **CECC2_CATH1** | **KLGKKLEGAGKPVKNQGACGSCWTFS** | **26** | **N-CPP** | **N-CPP** | **0,099684** | **0,9315** | **-** | **None** |
|  | **CECC2_CATL1** | **KLGKKLEGAGKPVKNQGQCGSCWAFS** | **26** | **N-CPP** | **N-CPP** | **0,087104** | **0,928** | **-** | **None** |
|  | **CECC2_CATH2** | **KLGKKLEGAGKYWIVKNSWG** | **20** | **N-CPP** | **N-CPP** | **0,132093** | **0,966** | **-** | **None** |
|  | **CECC2_CATL1.2** | **KLGKKLEGAGKYWLVKNSWG** | **20** | **N-CPP** | **N-CPP** | **0,159986** | **0,9615** | **-** | **None** |
|  | **CECD1_CATK** | **GQRIRDAIISAAPAVHAVLVVGYG** | **24** | **N-CPP** | **N-CPP** | **0,085453** | **0,776** | **-** | **None** |
|  | **CECD1_CATS** | **GQRIRDAIISAAPAVHGVLVVGYG** | **24** | **N-CPP** | **N-CPP** | **0,08534** | **0,7805** | **-** | **None** |
|  | **CECD1_CATH1** | **GQRIRDAIISAAPAVPVKNQGACGSCWTFS** | **30** | **N-CPP** | **N-CPP** | **0,168648** | **0,897** | **-** | **None** |
|  | **CECD1_CATL1** | **GQRIRDAIISAAPAVPVKNQGQCGSCWAFS** | **30** | **N-CPP** | **N-CPP** | **0,173044** | **0,915** | **-** | **None** |
|  | **CECD1_CATH2** | **GQRIRDAIISAAPAVYWIVKNSWG** | **24** | **N-CPP** | **N-CPP** | **0,149347** | **0,975** | **-** | **None** |
|  | **CECD1_CATL1.2** | **GQRIRDAIISAAPAVYWLVKNSWG** | **24** | **N-CPP** | **N-CPP** | **0,200345** | **0,98** | **-** | **None** |
|  | **CECD2_CATK** | **GQRVRDAVISAGPAVHAVLVVGYG** | **24** | **N-CPP** | **N-CPP** | **0,051261** | **0,7685** | **-** | **None** |
|  | **CECD2_CATS** | **GQRVRDAVISAGPAVHGVLVVGYG** | **24** | **N-CPP** | **N-CPP** | **0,04462** | **0,776** | **-** | **None** |
|  | **CECD2_CATH1** | **GQRVRDAVISAGPAVPVKNQGACGSCWTFS** | **30** | **N-CPP** | **N-CPP** | **0,100757** | **0,884** | **-** | **None** |
|  | **CECD2_CATL1** | **GQRVRDAVISAGPAVPVKNQGQCGSCWAFS** | **30** | **N-CPP** | **N-CPP** | **0,102526** | **0,875** | **-** | **None** |
|  | **CECD2_CATH2** | **GQRVRDAVISAGPAVYWIVKNSWG** | **24** | **N-CPP** | **N-CPP** | **0,10296** | **0,977** | **-** | **None** |
|  | **CECD2_CATL1.2** | **GQRVRDAVISAGPAVYWLVKNSWG** | **24** | **N-CPP** | **N-CPP** | **0,120743** | **0,9795** | **-** | **None** |
|  | **CECD3_CATK** | **GQRVRDAIISAGPAVHAVLVVGYG** | **24** | **N-CPP** | **N-CPP** | **0,039399** | **0,7735** | **-** | **None** |
|  | **CECD3_CATS** | **GQRVRDAIISAGPAVHGVLVVGYG** | **24** | **N-CPP** | **N-CPP** | **0,039273** | **0,781** | **-** | **None** |
|  | **CECD3_CATH1** | **GQRVRDAIISAGPAVPVKNQGACGSCWTFS** | **30** | **N-CPP** | **N-CPP** | **0,079332** | **0,8845** | **-** | **None** |
|  | **CECD3_CATL1** | **GQRVRDAIISAGPAVPVKNQGQCGSCWAFS** | **30** | **N-CPP** | **N-CPP** | **0,079308** | **0,892** | **-** | **None** |
|  | **CECD3_CATH2** | **GQRVRDAIISAGPAVYWIVKNSWG** | **24** | **N-CPP** | **N-CPP** | **0,077835** | **0,9785** | **-** | **None** |
|  | **CECD3_CATL1.2** | **GQRVRDAIISAGPAVYWLVKNSWG** | **24** | **N-CPP** | **N-CPP** | **0,096734** | **0,98** | **-** | **None** |
|  |  |  |  |  |  |  |  |  |  |
| **CATHEPSINS N TERMINAL** | **CATK_CEC1** | **HAVLVVGYGKKIERVGQNTRDA** | **22** | **N-CPP** | **N-CPP** | **0,11264** | **0,767** | **-** | **None** |
|  | **CATS_CEC1** | **HGVLVVGYGKKIERVGQNTRDA** | **22** | **N-CPP** | **N-CPP** | **0,119089** | **0,7705** | **VLVVGYGKKIERVG** | **0,16** |
|  | **CATH1_CEC1** | **PVKNQGACGSCWTFSKKIERVGQNTRDA** | **28** | **N-CPP** | **N-CPP** | **0,344028** | **0,754** | **-** | **None** |
|  | **CATL1_CEC1** | **PVKNQGQCGSCWAFSKKIERVGQNTRDA** | **28** | **N-CPP** | **N-CPP** | **0,40466** | **0,7715** | **-** | **None** |
|  | **CATH2_CEC1** | **YWIVKNSWGKKIERVGQNTRDA** | **22** | **N-CPP** | **N-CPP** | **0,388872** | **0,7655** | **WIVKNSWGKKIERVG** | **0,07** |
|  | **CATL1.2_CEC1** | **YWLVKNSWGKKIERVGQNTRDA** | **22** | **N-CPP** | **CPP** | **0,548823** | **0,7355** | **WLVKNSWGKKIERVG** | **0,08** |
|  | **CATK_CEC2** | **HAVLVVGYGKKIEKAVRRVRDG** | **22** | **N-CPP** | **N-CPP** | **0,071052** | **0,9215** | **VGYGKKIEKAVRRVRD** | **0,08** |
|  | **CATS_CEC2** | **HGVLVVGYGKKIEKAVRRVRDG** | **22** | **N-CPP** | **N-CPP** | **0,066902** | **0,914** | **GVLVVGYGKKIEKAVRRVRD** | **0,08** |
|  | **CATH1_CEC2** | **PVKNQGACGSCWTFSKKIEKAVRRVRDG** | **28** | **N-CPP** | **N-CPP** | **0,140233** | **0,943** | **WTFSKKIEKAVRRVRD** | **0,09** |
|  | **CATL1_CEC2** | **PVKNQGQCGSCWAFSKKIEKAVRRVRDG** | **28** | **N-CPP** | **N-CPP** | **0,159293** | **0,9465** | **AFSKKIEKAVRRVRD** | **0,11** |
|  | **CATH2_CEC2** | **YWIVKNSWGKKIEKAVRRVRDG** | **22** | **N-CPP** | **N-CPP** | **0,163295** | **0,9545** | **YWIVKNSWGKKIEKAVRRVRD** | **0,04** |
|  | **CATL1.2_CEC2** | **YWLVKNSWGKKIEKAVRRVRDG** | **22** | **N-CPP** | **N-CPP** | **0,269049** | **0,9585** | **YWLVKNSWGKKIEKAVRRVRD** | **0,05** |
|  | **CATK_CEC3** | **HAVLVVGYGKKIEKVGQNIRDG** | **22** | **N-CPP** | **N-CPP** | **0,064263** | **0,909** | **-** | **None** |
|  | **CATS_CEC3** | **HGVLVVGYGKKIEKVGQNIRDG** | **22** | **N-CPP** | **N-CPP** | **0,069106** | **0,9055** | **VLVVGYGKKIEKV** | **0,14** |
|  | **CATH1_CEC3** | **PVKNQGACGSCWTFSKKIEKVGQNIRDG** | **28** | **N-CPP** | **N-CPP** | **0,162919** | **0,9185** | **-** | **None** |
|  | **CATL1_CEC3** | **PVKNQGQCGSCWAFSKKIEKVGQNIRDG** | **28** | **N-CPP** | **N-CPP** | **0,186202** | **0,895** | **-** | **None** |
|  | **CATH2_CEC3** | **YWIVKNSWGKKIEKVGQNIRDG** | **22** | **N-CPP** | **N-CPP** | **0,177917** | **0,914** | **WIVKNSWGKKIEKV** | **0,06** |
|  | **CATL1.2_CEC3** | **YWLVKNSWGKKIEKVGQNIRDG** | **22** | **N-CPP** | **N-CPP** | **0,238066** | **0,9155** | **WLVKNSWGKKIEKV** | **0,07** |
|  | **CATK_CEC4** | **HAVLVVGYGKKIGRVGQHTRDA** | **22** | **N-CPP** | **N-CPP** | **0,121356** | **0,888** | **GYGKKIGRVGQHTR** | **0,09** |
|  | **CATS_CEC4** | **HGVLVVGYGKKIGRVGQHTRDA** | **22** | **N-CPP** | **N-CPP** | **0,126987** | **0,892** | **VLVVGYGKKIGRVGQHTR** | **0,08** |
|  | **CATH1_CEC4** | **PVKNQGACGSCWTFSKKIGRVGQHTRDA** | **28** | **N-CPP** | **N-CPP** | **0,223645** | **0,8865** | **WTFSKKIGRVGQHT** | **0,09** |
|  | **CATL1_CEC4** | **PVKNQGQCGSCWAFSKKIGRVGQHTRDA** | **28** | **N-CPP** | **N-CPP** | **0,296512** | **0,885** | **FSKKIGRVGQHTR** | **0,12** |
|  | **CATH2_CEC4** | **YWIVKNSWGKKIGRVGQHTRDA** | **22** | **N-CPP** | **N-CPP** | **0,299454** | **0,876** | **WIVKNSWGKKIGRVGQHTR** | **0,04** |
|  | **CATL1.2_CEC4** | **YWLVKNSWGKKIGRVGQHTRDA** | **22** | **N-CPP** | **N-CPP** | **0,437945** | **0,866** | **WLVKNSWGKKIGRVGQHTR** | **0,05** |
|  | **CATK_CECA1** | **HAVLVVGYGKLGKKLEGAGKR** | **21** | **N-CPP** | **N-CPP** | **0,086158** | **0,857** | **-** | **None** |
|  | **CATS_CECA1** | **HGVLVVGYGKLGKKLEGAGKR** | **21** | **N-CPP** | **N-CPP** | **0,091459** | **0,845** | **VLVVGYGKLGKKLE** | **0,18** |
|  | **CATH1_CECA1** | **PVKNQGACGSCWTFSKLGKKLEGAGKR** | **27** | **N-CPP** | **N-CPP** | **0,162218** | **0,928** | **-** | **None** |
|  | **CATL1_CECA1** | **PVKNQGQCGSCWAFSKLGKKLEGAGKR** | **27** | **N-CPP** | **N-CPP** | **0,157737** | **0,9185** | **-** | **None** |
|  | **CATH2_CECA1** | **YWIVKNSWGKLGKKLEGAGKR** | **21** | **N-CPP** | **N-CPP** | **0,219563** | **0,871** | **WIVKNSWGKLGKKL** | **0,07** |
|  | **CATL1.2_CECA1** | **YWLVKNSWGKLGKKLEGAGKR** | **21** | **N-CPP** | **N-CPP** | **0,269012** | **0,872** | **WLVKNSWGKLGKKL** | **0,09** |
|  | **CATK_CECA2** | **HAVLVVGYGKIGKKIERVGQH** | **21** | **N-CPP** | **N-CPP** | **0,101213** | **0,8385** | **VGYGKIGKKIERVGQ** | **0,09** |
|  | **CATS_CECA2** | **HGVLVVGYGKIGKKIERVGQH** | **21** | **N-CPP** | **N-CPP** | **0,105749** | **0,831** | **GVLVVGYGKIGKKIERVGQ** | **0,08** |
|  | **CATH1_CECA2** | **PVKNQGACGSCWTFSKIGKKIERVGQH** | **27** | **N-CPP** | **N-CPP** | **0,176366** | **0,969** | **WTFSKIGKKIERVG** | **0,11** |
|  | **CATL1_CECA2** | **PVKNQGQCGSCWAFSKIGKKIERVGQH** | **27** | **N-CPP** | **N-CPP** | **0,247446** | **0,977** | **FSKIGKKIERVG** | **0,14** |
|  | **CATH2_CECA2** | **YWIVKNSWGKIGKKIERVGQH** | **21** | **N-CPP** | **N-CPP** | **0,261233** | **0,8525** | **YWIVKNSWGKIGKKIERVGQ** | **0,04** |
|  | **CATL1.2_CECA2** | **YWLVKNSWGKIGKKIERVGQH** | **21** | **N-CPP** | **N-CPP** | **0,378201** | **0,86** | **YWLVKNSWGKIGKKIERVGQ** | **0,05** |
|  | **CATK_CECA3** | **HAVLVVGYGKVFKKIEKVGRN** | **21** | **N-CPP** | **N-CPP** | **0,065151** | **0,875** | **LVVGYGKVFKKIEKVGR** | **0** |
|  | **CATS_CECA3** | **HGVLVVGYGKVFKKIEKVGRN** | **21** | **N-CPP** | **N-CPP** | **0,070169** | **0,866** |  |  |
|  | **CATH1_CECA3** | **PVKNQGACGSCWTFSKVFKKIEKVGRN** | **27** | **N-CPP** | **N-CPP** | **0,197573** | **0,9695** | **SCWTFSKVFKKIEKVGR** | **0,02** |
|  | **CATL1_CECA3** | **PVKNQGQCGSCWAFSKVFKKIEKVGRN** | **27** | **N-CPP** | **N-CPP** | **0,213682** | **0,9685** | **CWAFSKVFKKIEKVGR** | **0,01** |
|  | **CATH2_CECA3** | **YWIVKNSWGKVFKKIEKVGRN** | **21** | **N-CPP** | **N-CPP** | **0,193971** | **0,8615** | **-** | **None** |
|  | **CATL1.2_CECA3** | **YWLVKNSWGKVFKKIEKVGRN** | **21** | **N-CPP** | **N-CPP** | **0,266868** | **0,866** | **-** | **None** |
|  | **CATK_CECA4** | **HAVLVVGYGKFGKKLEGVGKR** | **21** | **N-CPP** | **N-CPP** | **0,058707** | **0,852** | **-** | **None** |
|  | **CATS_CECA4** | **HGVLVVGYGKFGKKLEGVGKR** | **21** | **N-CPP** | **N-CPP** | **0,069939** | **0,84** | **VLVVGYGKFGKKLE** | **0,16** |
|  | **CATH1_CECA4** | **PVKNQGACGSCWTFSKFGKKLEGVGKR** | **27** | **N-CPP** | **N-CPP** | **0,121489** | **0,954** | **-** | **None** |
|  | **CATL1_CECA4** | **PVKNQGQCGSCWAFSKFGKKLEGVGKR** | **27** | **N-CPP** | **N-CPP** | **0,146847** | **0,9385** | **-** | **None** |
|  | **CATH2_CECA4** | **YWIVKNSWGKFGKKLEGVGKR** | **21** | **N-CPP** | **N-CPP** | **0,149415** | **0,8675** | **WIVKNSWGKFGKKL** | **0,07** |
|  | **CATL1.2_CECA4** | **YWLVKNSWGKFGKKLEGVGKR** | **21** | **N-CPP** | **N-CPP** | **0,199268** | **0,8705** | **WLVKNSWGKFGKKL** | **0,08** |
|  | **CATK_CECB1** | **HAVLVVGYGKLGKKIE** | **16** | **N-CPP** | **N-CPP** | **0,076305** | **0,7355** | **-** | **None** |
|  | **CATS_CECB1** | **HGVLVVGYGKLGKKIE** | **16** | **N-CPP** | **N-CPP** | **0,079039** | **0,7285** | **HGVLVVGYGKLGKKI** | **0,04** |
|  | **CATH1_CECB1** | **PVKNQGACGSCWTFSKLGKKIE** | **22** | **N-CPP** | **N-CPP** | **0,219404** | **0,944** | **-** | **None** |
|  | **CATL1_CECB1** | **PVKNQGQCGSCWAFSKLGKKIE** | **22** | **N-CPP** | **N-CPP** | **0,257045** | **0,9375** | **-** | **None** |
|  | **CATH2_CECB1** | **YWIVKNSWGKLGKKIE** | **16** | **N-CPP** | **N-CPP** | **0,323416** | **0,8345** | **WIVKNSWGKLGKKI** | **0,05** |
|  | **CATL1.2_CECB1** | **YWLVKNSWGKLGKKIE** | **16** | **N-CPP** | **N-CPP** | **0,492165** | **0,8365** | **WLVKNSWGKLGKKI** | **0,07** |
|  | **CATK_CECB2** | **HAVLVVGYGKIFKKIE** | **16** | **N-CPP** | **N-CPP** | **0,097911** | **0,7505** | **VLVVGYGKIFKKI** | **0** |
|  | **CATS_CECB2** | **HGVLVVGYGKIFKKIE** | **16** | **N-CPP** | **N-CPP** | **0,100622** | **0,7545** | **-** | **None** |
|  | **CATH1_CECB2** | **PVKNQGACGSCWTFSKIFKKIE** | **22** | **N-CPP** | **N-CPP** | **0,257303** | **0,946** | **GSCWTFSKIFKKI** | **0** |
|  | **CATL1_CECB2** | **PVKNQGQCGSCWAFSKIFKKIE** | **22** | **N-CPP** | **N-CPP** | **0,320251** | **0,943** | **SCWAFSKIFKKI** | **0** |
|  | **CATH2_CECB2** | **YWIVKNSWGKIFKKIE** | **16** | **N-CPP** | **N-CPP** | **0,375959** | **0,81** | **-** | **None** |
|  | **CATL1.2_CECB2** | **YWLVKNSWGKIFKKIE** | **16** | **N-CPP** | **CPP** | **0,589472** | **0,8155** | **-** | **None** |
|  | **CATK_CECB3** | **HAVLVVGYGKVFKKIE** | **16** | **N-CPP** | **N-CPP** | **0,069281** | **0,737** | **VLVVGYGKVFKKI** | **0** |
|  | **CATS_CECB3** | **HGVLVVGYGKVFKKIE** | **16** | **N-CPP** | **N-CPP** | **0,07486** | **0,7365** | **-** | **None** |
|  | **CATH1_CECB3** | **PVKNQGACGSCWTFSKVFKKIE** | **22** | **N-CPP** | **N-CPP** | **0,191852** | **0,943** | **GSCWTFSKVFKKI** | **0** |
|  | **CATL1_CECB3** | **PVKNQGQCGSCWAFSKVFKKIE** | **22** | **N-CPP** | **N-CPP** | **0,218518** | **0,9435** | **SCWAFSKVFKKI** | **0** |
|  | **CATH2_CECB3** | **YWIVKNSWGKVFKKIE** | **16** | **N-CPP** | **N-CPP** | **0,244385** | **0,807** | **-** | **None** |
|  | **CATL1.2_CECB3** | **YWLVKNSWGKVFKKIE** | **16** | **N-CPP** | **N-CPP** | **0,379286** | **0,809** | **-** | **None** |
|  | **CATK_CECC1** | **HAVLVVGYGKLGKRIERIGQ** | **20** | **N-CPP** | **N-CPP** | **0,135352** | **0,8625** | **VVGYGKLGKRIERIG** | **0,03** |
|  | **CATS_CECC1** | **HGVLVVGYGKLGKRIERIGQ** | **20** | **N-CPP** | **N-CPP** | **0,130612** | **0,855** | **HGVLVVGYGKLGKRIERIG** | **0,04** |
|  | **CATH1_CECC1** | **PVKNQGACGSCWTFSKLGKRIERIGQ** | **26** | **N-CPP** | **N-CPP** | **0,158409** | **0,984** | **WTFSKLGKRIERIG** | **0,14** |
|  | **CATL1_CECC1** | **PVKNQGQCGSCWAFSKLGKRIERIGQ** | **26** | **N-CPP** | **N-CPP** | **0,208509** | **0,9655** | **FSKLGKRIERIG** | **0,18** |
|  | **CATH2_CECC1** | **YWIVKNSWGKLGKRIERIGQ** | **20** | **N-CPP** | **N-CPP** | **0,266102** | **0,8655** | **WIVKNSWGKLGKRIERIG** | **0,05** |
|  | **CATL1.2_CECC1** | **YWLVKNSWGKLGKRIERIGQ** | **20** | **N-CPP** | **N-CPP** | **0,376259** | **0,871** | **WLVKNSWGKLGKRIERIG** | **0,06** |
|  | **CATK_CECC2** | **HAVLVVGYGKLGKKLEGAGK** | **20** | **N-CPP** | **N-CPP** | **0,04773** | **0,8455** | **-** | **None** |
|  | **CATS_CECC2** | **HGVLVVGYGKLGKKLEGAGK** | **20** | **N-CPP** | **N-CPP** | **0,05241** | **0,8425** | **VLVVGYGKLGKKLE** | **0,18** |
|  | **CATH1_CECC2** | **PVKNQGACGSCWTFSKLGKKLEGAGK** | **26** | **N-CPP** | **N-CPP** | **0,099684** | **0,94** | **-** | **None** |
|  | **CATL1_CECC2** | **PVKNQGQCGSCWAFSKLGKKLEGAGK** | **26** | **N-CPP** | **N-CPP** | **0,087104** | **0,9345** | **-** | **None** |
|  | **CATH2_CECC2** | **YWIVKNSWGKLGKKLEGAGK** | **20** | **N-CPP** | **N-CPP** | **0,132093** | **0,8775** | **WIVKNSWGKLGKKL** | **0,07** |
|  | **CATL1.2_CECC2** | **YWLVKNSWGKLGKKLEGAGK** | **20** | **N-CPP** | **N-CPP** | **0,159986** | **0,876** | **WLVKNSWGKLGKKL** | **0,09** |
|  | **CATK_CECD1** | **HAVLVVGYGGQRIRDAIISAAPAV** | **24** | **N-CPP** | **N-CPP** | **0,085453** | **0,619** | **-** | **None** |
|  | **CATS_CECD1** | **HGVLVVGYGGQRIRDAIISAAPAV** | **24** | **N-CPP** | **N-CPP** | **0,08534** | **0,6365** | **-** | **None** |
|  | **CATH1_CECD1** | **PVKNQGACGSCWTFSGQRIRDAIISAAPAV** | **30** | **N-CPP** | **N-CPP** | **0,132093** | **0,784** | **-** | **None** |
|  | **CATL1_CECD1** | **PVKNQGQCGSCWAFSGQRIRDAIISAAPAV** | **30** | **N-CPP** | **N-CPP** | **0,136013** | **0,877** | **-** | **None** |
|  | **CATH2_CECD1** | **YWIVKNSWGGQRIRDAIISAAPAV** | **24** | **N-CPP** | **N-CPP** | **0,182708** | **0,9625** | **WIVKNSWGGQRI** | **0,12** |
|  | **CATL1.2_CECD1** | **YWLVKNSWGGQRIRDAIISAAPAV** | **24** | **N-CPP** | **N-CPP** | **0,25874** | **0,968** | **WLVKNSWGGQRI** | **0,15** |
|  | **CATK_CECD2** | **HAVLVVGYGGQRVRDAVISAGPAV** | **24** | **N-CPP** | **N-CPP** | **0,051261** | **0,566** | **-** | **None** |
|  | **CATS_CECD2** | **HGVLVVGYGGQRVRDAVISAGPAV** | **24** | **N-CPP** | **N-CPP** | **0,04462** | **0,579** | **-** | **None** |
|  | **CATH1_CECD2** | **PVKNQGACGSCWTFSGQRVRDAVISAGPAV** | **30** | **N-CPP** | **N-CPP** | **0,071976** | **0,7015** | **-** | **None** |
|  | **CATL1_CECD2** | **PVKNQGQCGSCWAFSGQRVRDAVISAGPAV** | **30** | **N-CPP** | **N-CPP** | **0,073531** | **0,7295** | **-** | **None** |
|  | **CATH2_CECD2** | **YWIVKNSWGGQRVRDAVISAGPAV** | **24** | **N-CPP** | **N-CPP** | **0,121515** | **0,9635** | **WIVKNSWGGQRV** | **0,12** |
|  | **CATL1.2_CECD2** | **YWLVKNSWGGQRVRDAVISAGPAV** | **24** | **N-CPP** | **N-CPP** | **0,150697** | **0,9585** | **WLVKNSWGGQRV** | **0,15** |
|  | **CATK_CECD3** | **HAVLVVGYGGQRVRDAIISAGPAV** | **24** | **N-CPP** | **N-CPP** | **0,039399** | **0,5985** | **-** | **None** |
|  | **CATS_CECD3** | **HGVLVVGYGGQRVRDAIISAGPAV** | **24** | **N-CPP** | **N-CPP** | **0,039273** | **0,6115** | **-** | **None** |
|  | **CATH1_CECD3** | **PVKNQGACGSCWTFSGQRVRDAIISAGPAV** | **30** | **N-CPP** | **N-CPP** | **0,059985** | **0,7415** | **-** | **None** |
|  | **CATL1_CECD3** | **PVKNQGQCGSCWAFSGQRVRDAIISAGPAV** | **30** | **N-CPP** | **N-CPP** | **0,060106** | **0,768** | **-** | **None** |
|  | **CATH2_CECD3** | **YWIVKNSWGGQRVRDAIISAGPAV** | **24** | **N-CPP** | **N-CPP** | **0,089923** | **0,967** | **WIVKNSWGGQRV** | **0,12** |
|  | **CATL1.2_CECD3** | **YWLVKNSWGGQRVRDAIISAGPAV** | **24** | **N-CPP** | **N-CPP** | **0,119279** | **0,966** | **WLVKNSWGGQRV** | **0,15** |
|  | **CATL1.2_CECB1_2** | **WLVKNSWGKLGKKI** | **14** | **-** | **N-CPP** | **0,427371** | **0,99** | **-** | **None** |
|  | **Parameters** | **-** | **-** | **-** | **-** | **>0.5: CPP**  **<0.5: N-CPP** | **>0.8: AMP**  **<0.8: N-AMP** | **-** | **Probability of being present in a non-AMP sequence** |

**Table S4 - Physicochemical predictions**

|  |  |  | **PROTPARAM** | | **PepCalc** | **PROTPARAM** | | **APD3** | **HELIQUEST** | | | | | | **TMHMM 2** | |
| --- | --- | --- | --- | --- | --- | --- | --- | --- | --- | --- | --- | --- | --- | --- | --- | --- |
|  | **NAME** | **HYBRID PEPTIDE** | **N° AMINO ACID** | **MOL WT** | **SOLUBILITY IN WATER** | **INSTABILITY** | **GRAVY** | **BOMAN (kcal/mol)** | **Hydrophobicity** | **HYDROPHOBIC MOMENT µH** | **Polar residues + GLY** | | | **Nonpolar residues** | **Cellular Localization** | **Total probability of N-in by TMHMM server** |
|  |  |  |  |  |  |  |  |  |  |  | **Polar residues + GLY (n / %)** | **Uncharged residues + GLY** | **Charged residues** | **Nonpolar residues (n / %)** |  |  |
| **CECROPIN N TERMINAL** | **CEC1_CATK** | **KKIERVGQNTRDAHAVLVVGYG** | **22** | **2410,76** | **GOOD** | **2,5** | **-0,386** | **1,98** | **0,187** | **0,315** | **13 / 59.09** | **GLN 1, HIS 1, THR 1, ASN 1, GLY 3** | **LYS 2, ARG 2, GLU 1, ASP 1,** | **9 / 40.91** | **inside** | **0,81** |
|  | **CEC1_CATS** | **KKIERVGQNTRDAHGVLVVGYG** | **22** | **2396,73** | **GOOD** | **-2,21** | **-0,486** | **2,02** | **0,173** | **0,325** | **14 / 63.64** | **GLN 1, HIS 1, THR 1, ASN 1, GLY 4** | **LYS 2, ARG 2, GLU 1, ASP 1,** | **8 / 36.36** | **inside** | **0,80** |
|  | **CEC1_CATH1** | **KKIERVGQNTRDAPVKNQGACGSCWTFS** | **28** | **3081,47** | **GOOD** | **58,07** | **-0,861** | **2,53** | **0,182** | **0,327** | **18 / 64.29** | **GLN 2, SER 2, THR 2, ASN 2, GLY 3** | **LYS 3, ARG 2, GLU 1, ASP 1,** | **10 / 35.71** | **inside** | **0,80** |
|  | **CEC1_CATL1** | **KKIERVGQNTRDAPVKNQGQCGSCWAFS** | **28** | **3108,5** | **GOOD** | **38,31** | **-0,961** | **2,64** | **0,165** | **0,327** | **18 / 64.29** | **GLN 3, SER 2, THR 1, ASN 2, GLY 3** | **LYS 3, ARG 2, GLU 1, ASP 1,** | **10 / 35.71** | **inside** | **0,80** |
|  | **CEC1_CATH2** | **KKIERVGQNTRDAYWIVKNSWG** | **22** | **2649,01** | **GOOD** | **3,35** | **-1,109** | **2,75** | **0,191** | **0,362** | **14 / 63.64** | **GLN 1, SER 1, THR 1, ASN 2, GLY 2** | **LYS 3, ARG 2, GLU 1, ASP 1,** | **8 / 36.36** | **inside** | **0,81** |
|  | **CEC1_CATL1.2** | **KKIERVGQNTRDAYWLVKNSWG** | **22** | **2649,01** | **GOOD** | **12,81** | **-1,141** | **2,75** | **0,187** | **0,364** | **14 / 63.64** | **GLN 1, SER 1, THR 1, ASN 2, GLY 2** | **LYS 3, ARG 2, GLU 1, ASP 1,** | **8 / 36.36** | **inside** | **0,81** |
|  | **CEC2_CATK** | **KKIEKAVRRVRDGHAVLVVGYG** | **22** | **2450,92** | **GOOD** | **40,12** | **-0,227** | **2,05** | **0,177** | **0,401** | **12 / 54.55** | **HIS 1, GLY 3** | **LYS 3, ARG 3, GLU 1, ASP 1,** | **10 / 45.45** | **inside** | **0,88** |
|  | **CEC2_CATS** | **KKIEKAVRRVRDGHGVLVVGYG** | **22** | **2436,89** | **GOOD** | **35,4** | **-0,327** | **2,09** | **0,163** | **0,409** | **13 / 59.09** | **HIS 1, GLY 4** | **LYS 3, ARG 3, GLU 1, ASP 1,** | **9 / 40.91** | **inside** | **0,88** |
|  | **CEC2_CATH1** | **KKIEKAVRRVRDGPVKNQGACGSCWTFS** | **28** | **3121,63** | **GOOD** | **77,72** | **-0,736** | **2,59** | **0,174** | **0,4** | **17 / 60.71** | **GLN 1, SER 2, THR 1, ASN 1, GLY 3** | **LYS 4, ARG 3, GLU 1, ASP 1,** | **11 / 39.29** | **inside** | **0,88** |
|  | **CEC2_CATL1** | **KKIEKAVRRVRDGPVKNQGQCGSCWAFS** | **28** | **3148,65** | **GOOD** | **57,96** | **-0,836** | **2,70** | **0,157** | **0,403** | **17 / 60.71** | **GLN 2, SER 2, ASN 1, GLY 3** | **LYS 4, ARG 3, GLU 1, ASP 1,** | **11 / 39.29** | **inside** | **0,88** |
|  | **CEC2_CATH2** | **KKIEKAVRRVRDGYWIVKNSWG** | **22** | **2689,16** | **GOOD** | **33,24** | **-0,95** | **2,83** | **0,181** | **0,444** | **13 / 59.09** | **SER 1, ASN 1, GLY 2** | **LYS 4, ARG 3, GLU 1, ASP 1,** | **9 / 40.91** | **inside** | **0,89** |
|  | **CEC2_CATL.2** | **KKIEKAVRRVRDGYWLVKNSWG** | **22** | **2689,16** | **GOOD** | **42,71** | **-0,982** | **2,83** | **0,177** | **0,447** | **13 / 59.09** | **SER 1, ASN 1, GLY 2** | **LYS 4, ARG 3, GLU 1, ASP 1,** | **9 / 40.91** | **inside** | **0,89** |
|  | **CEC3_CATK** | **KKIEKVGQNIRDGHAVLVVGYG** | **22** | **2380,78** | **GOOD** | **26,34** | **-0,223** | **1,25** | **0,244** | **0,378** | **13 / 59.09** | **GLN 1, HIS 1, ASN 1, GLY 4** | **LYS 3, ARG 1, GLU 1, ASP 1,** | **9 / 40.91** | **inside** | **0,73** |
|  | **CEC3_CATS** | **KKIEKVGQNIRDGHGVLVVGYG** | **22** | **2366,75** | **GOOD** | **21,62** | **-0,323** | **1,29** | **0,230** | **0,388** | **14 / 63.64** | **GLN 1, HIS 1, ASN 1, GLY 5** | **LYS 3, ARG 1, GLU 1, ASP 1,** | **8 / 36.36** | **inside** | **0,73** |
|  | **CEC3_CATH1** | **KKIEKVGQNIRDGPVKNQGACGSCWTFS** | **28** | **3051,49** | **GOOD** | **66,89** | **-0,732** | **1,96** | **0,227** | **0,376** | **18 / 64.29** | **GLN 2, SER 2, THR 1, ASN 2, GLY 4** | **LYS 4, ARG 1, GLU 1, ASP 1,** | **10 / 35.71** | **inside** | **0,72** |
|  | **CEC3_CATL1** | **KKIEKVGQNIRDGPVKNQGQCGSCWAFS** | **28** | **3078,1** | **GOOD** | **47,13** | **-0,832** | **2,07** | **0,21** | **0,376** | **18 / 64.29** | **GLN 3, SER 2, ASN 2, GLY 4** | **LYS 4, ARG 1, GLU 1, ASP 1,** | **10 / 35.71** | **inside** | **0,72** |
|  | **CEC3_CATH2** | **KKIEKVGQNIRDGYWIVKNSWG** | **22** | **2619,02** | **GOOD** | **19,46** | **-0,945** | **2,03** | **0,248** | **0,425** | **14 / 63.64** | **GLN 1, SER 1, ASN 2, GLY 3** | **LYS 4, ARG 1, GLU 1, ASP 1,** | **8 / 36.36** | **inside** | **0,73** |
|  | **CEC3_CATL1.2** | **KKIEKVGQNIRDGYWLVKNSWG** | **22** | **2619,02** | **GOOD** | **28,93** | **-0,977** | **2,03** | **0,244** | **0,427** | **14 / 63.64** | **GLN 1, SER 1, ASN 2, GLY 3** | **LYS 4, ARG 1, GLU 1, ASP 1,** | **8 / 36.36** | **inside** | **0,73** |
|  | **CEC4_CATK** | **KKIGRVGQHTRDAHAVLVVGYG** | **22** | **2361,74** | **GOOD** | **-17,04** | **-0,232** | **1,53** | **0,25** | **0,294** | **13 / 59.09** | **GLN 1, HIS 2, THR 1, GLY 4** | **LYS 2, ARG 2, ASP 1,** | **9 / 40.91** | **inside** | **0,73** |
|  | **CEC4_CATS** | **KKIGRVGQHTRDAHGVLVVGYG** | **22** | **2347,71** | **GOOD** | **-21,75** | **-0,332** | **1,57** | **0,235** | **0,305** | **14 / 63.64** | **GLN 1, HIS 2, THR 1, GLY 5** | **LYS 2, ARG 2, ASP 1,** | **8 / 36.36** | **inside** | **0,73** |
|  | **CEC4_CATH1** | **KKIGRVGQHTRDAPVKNQGACGSCWTFS** | **28** | **3032,45** | **GOOD** | **42,72** | **-0,739** | **2,18** | **0,231** | **0,31** | **18 / 64.29** | **GLN 2, HIS 1, SER 2, THR 2, ASN 1, GLY 4** | **LYS 3, ARG 2, ASP 1,** | **10 / 35.71** | **inside** | **0,73** |
|  | **CEC4_CATL1** | **KKIGRVGQHTRDAPVKNQGQCGSCWAFS** | **28** | **3059,47** | **GOOD** | **22,96** | **-0,839** | **2,29** | **0,214** | **0,31** | **18 / 64.29** | **GLN 3, HIS 1, SER 2, THR 1, ASN 1, GLY 4** | **LYS 3, ARG 2, ASP 1,** | **10 / 35.71** | **inside** | **0,73** |
|  | **CEC4_CATH2** | **KKIGRVGQHTRDAYWIVKNSWG** | **22** | **2599,98** | **GOOD** | **-16,2** | **-0,955** | **2,31** | **0,254** | **0,341** | **14 / 63.64** | **GLN 1, HIS 1, SER 1, THR 1, ASN 1, GLY 3** | **LYS 3, ARG 2, ASP 1,** | **8 / 36.36** | **inside** | **0,74** |
|  | **CEC4_CATL1.2** | **KKIGRVGQHTRDAYWLVKNSWG** | **22** | **2599,98** | **GOOD** | **-6,73** | **-0,986** | **2,31** | **0,249** | **0,344** | **14 / 63.64** | **GLN 1, HIS 1, SER 1, THR 1, ASN 1, GLY 3** | **LYS 3, ARG 2, ASP 1,** | **8 / 36.36** | **inside** | **0,73** |
|  | **CECA1_CATK** | **KLGKKLEGAGKRHAVLVVGYG** | **21** | **2180,62** | **GOOD** | **1,88** | **-0,119** | **0,64** | **0,231** | **0,333** | **12 / 57.14** | **HIS 1, GLY 5** | **LYS 4, ARG 1, GLU 1,** | **9 / 42.86** | **outside** | **0,25** |
|  | **CECA1_CATS** | **KLGKKLEGAGKRHGVLVVGYG** | **21** | **2166,6** | **GOOD** | **-3,06** | **-0,224** | **0,68** | **0,217** | **0,333** | **13 / 61.90** | **HIS 1, GLY 6** | **LYS 4, ARG 1, GLU 1,** | **8 / 38.10** | **outside** | **0,25** |
|  | **CECA1_CATH1** | **KLGKKLEGAGKRPVKNQGACGSCWTFS** | **27** | **2851,34** | **GOOD** | **49,37** | **-0,67** | **1,51** | **0,216** | **0,348** | **17 / 62.96** | **GLN 1, SER 2, THR 1, ASN 1, GLY 5** | **LYS 5, ARG 1, GLU 1,** | **10 / 37.04** | **outside** | **0,25** |
|  | **CECA1_CATL1** | **KLGKKLEGAGKRPVKNQGQCGSCWAFS** | **27** | **2878,36** | **GOOD** | **28,87** | **-0,774** | **1,62** | **0,199** | **0,36** | **17 / 62.96** | **GLN 2, SER 2, ASN 1, GLY 5** | **LYS 5, ARG 1, GLU 1,** | **10 / 37.04** | **outside** | **0,25** |
|  | **CECA1_CATH2** | **KLGKKLEGAGKRYWIVKNSWG** | **21** | **2418,87** | **GOOD** | **-14,05** | **-0,876** | **1,45** | **0,236** | **0,358** | **13 / 61.90** | **SER 1, ASN 1, GLY 4** | **LYS 5, ARG 1, GLU 1,** | **8 / 38.10** | **outside** | **0,26** |
|  | **CECA1_CATL1.2** | **KLGKKLEGAGKRYWLVKNSWG** | **21** | **2418,87** | **GOOD** | **-4,13** | **-0,91** | **1,45** | **0,231** | **0,362** | **13 / 61.90** | **SER 1, ASN 1, GLY 4** | **LYS 5, ARG 1, GLU 1,** | **8 / 38.10** | **outside** | **0,26** |
|  | **CECA2_CATK** | **KIGKKIERVGQHHAVLVVGYG** | **21** | **2288,72** | **GOOD** | **2,15** | **-0,052** | **0,80** | **0,327** | **0,363** | **12 / 57.14** | **GLN 1, HIS 2, GLY 4** | **LYS 3, ARG 1, GLU 1,** | **9 / 42.86** | **inside** | **0,68** |
|  | **CECA2_CATS** | **KIGKKIERVGQHHGVLVVGYG** | **21** | **2274,7** | **GOOD** | **-2,79** | **-0,157** | **0,84** | **0,312** | **0,364** | **13 / 61.90** | **GLN 1, HIS 2, GLY 5** | **LYS 3, ARG 1, GLU 1,** | **8 / 38.10** | **inside** | **0,67** |
|  | **CECA2_CATH1** | **KIGKKIERVGQHPVKNQGACGSCWTFS** | **27** | **2959,44** | **GOOD** | **48,51** | **-0,619** | **1,64** | **0,291** | **0,373** | **17 / 62.96** | **GLN 2, HIS 1, SER 2, THR 1, ASN 1, GLY 4** | **LYS 4, ARG 1, GLU 1,** | **10 / 37.04** | **inside** | **0,67** |
|  | **CECA2_CATL1** | **KIGKKIERVGQHPVKNQGQCGSCWAFS** | **27** | **2986,46** | **GOOD** | **28,02** | **-0,722** | **1,75** | **0,273** | **0,383** | **17 / 62.96** | **GLN 3, HIS 1, SER 2, ASN 1, GLY 4** | **LYS 4, ARG 1, GLU 1,** | **10 / 37.04** | **inside** | **0,67** |
|  | **CECA2_CATH2** | **KIGKKIERVGQHYWIVKNSWG** | **21** | **2526,97** | **GOOD** | **19,91** | **-0,81** | **1,61** | **0,331** | **0,392** | **13 / 61.90** | **GLN 1, HIS 1, SER 1, ASN 1, GLY 3** | **LYS 4, ARG 1, GLU 1,** | **8 / 38.10** | **inside** | **0,68** |
|  | **CECA2_CATL1.2** | **KIGKKIERVGQHYWLVKNSWG** | **21** | **2526,97** | **GOOD** | **29,83** | **-0,843** | **1,61** | **0,327** | **0,397** | **13 / 61.90** | **GLN 1, HIS 1, SER 1, ASN 1, GLY 3** | **LYS 4, ARG 1, GLU 1,** | **8 / 38.10** | **inside** | **0,68** |
|  | **CECA3_CATK** | **KVFKKIEKVGRNHAVLVVGYG** | **21** | **2341,83** | **GOOD** | **0,87** | **0,052** | **0,84** | **0,313** | **0,416** | **11 / 52.38** | **HIS 1, ASN 1, GLY 3** | **LYS 4, ARG 1, GLU 1,** | **10 / 47.62** | **inside** | **0,60** |
|  | **CECA3_CATS** | **KVFKKIEKVGRNHGVLVVGYG** | **21** | **2327,8** | **GOOD** | **-4,07** | **-0,052** | **0,88** | **0,299** | **0,414** | **12 / 57.14** | **HIS 1, ASN 1, GLY 4** | **LYS 4, ARG 1, GLU 1,** | **9 / 42.86** | **inside** | **0,60** |
|  | **CECA3_CATH1** | **KVFKKIEKVGRNPVKNQGACGSCWTFS** | **27** | **3012,54** | **GOOD** | **47,51** | **-0,537** | **1,67** | **0,28** | **0,409** | **16 / 59.26** | **GLN 1, SER 2, THR 1, ASN 2, GLY 3** | **LYS 5, ARG 1, GLU 1,** | **11 / 40.74** | **inside** | **0,60** |
|  | **CECA3_CATL1** | **KVFKKIEKVGRNPVKNQGQCGSCWAFS** | **27** | **3039,57** | **GOOD** | **27,02** | **-0,641** | **1,78** | **0,262** | **0,423** | **16 / 59.26** | **GLN 2, SER 2, ASN 2, GLY 3** | **LYS 5, ARG 1, GLU 1,** | **11 / 40.74** | **inside** | **0,60** |
|  | **CECA3_CATH2** | **KVFKKIEKVGRNYWIVKNSWG** | **21** | **2580,07** | **GOOD** | **-2,3** | **-0,705** | **1,65** | **0,318** | **0,433** | **12 / 57.14** | **SER 1, ASN 2, GLY 2** | **LYS 5, ARG 1, GLU 1,** | **9 / 42.86** | **inside** | **0,61** |
|  | **CECA3_CATL1.2** | **KVFKKIEKVGRNYWLVKNSWG** | **21** | **2580,07** | **GOOD** | **7,62** | **-0,738** | **1,65** | **0,313** | **0,438** | **12 / 57.14** | **SER 1, ASN 2, GLY 2** | **LYS 5, ARG 1, GLU 1,** | **9 / 42.86** | **inside** | **0,60** |
|  | **CECA4_CATK** | **KFGKKLEGVGKRHAVLVVGYG** | **21** | **2242,7** | **GOOD** | **5,92** | **-0,052** | **0,63** | **0,279** | **0,365** | **12 / 57.14** | **HIS 1, GLY 5** | **LYS 4, ARG 1, GLU 1,** | **9 / 42.86** | **outside** | **0,23** |
|  | **CECA4_CATS** | **KFGKKLEGVGKRHGVLVVGYG** | **21** | **2228,67** | **GOOD** | **0,98** | **-0,157** | **0,67** | **0,264** | **0,367** | **13 / 61.90** | **HIS 1, GLY 6** | **LYS 4, ARG 1, GLU 1,** | **8 / 38.10** | **outside** | **0,23** |
|  | **CECA4_CATH1** | **KFGKKLEGVGKRPVKNQGACGSCWTFS** | **27** | **2913,41** | **GOOD** | **52,51** | **-0,619** | **1,50** | **0,253** | **0,375** | **17 / 62.96** | **GLN 1, SER 2, THR 1, ASN 1, GLY 5** | **LYS 5, ARG 1, GLU 1,** | **10 / 37.04** | **outside** | **0,23** |
|  | **CECA4_CATL1** | **KFGKKLEGVGKRPVKNQGQCGSCWAFS** | **27** | **2940,43** | **GOOD** | **32,02** | **-0,722** | **1,61** | **0,236** | **0,385** | **17 / 62.96** | **GLN 2, SER 2, ASN 1, GLY 5** | **LYS 5, ARG 1, GLU 1,** | **10 / 37.04** | **outside** | **0,23** |
|  | **CECA4_CATH2** | **KFGKKLEGVGKRYWIVKNSWG** | **21** | **2480,94** | **GOOD** | **-10** | **-0,81** | **1,44** | **0,283** | **0,394** | **13 / 61.90** | **SER 1, ASN 1, GLY 4** | **LYS 5, ARG 1, GLU 1,** | **8 / 38.10** | **outside** | **0,24** |
|  | **CECA4_CATL1.2** | **KFGKKLEGVGKRYWLVKNSWG** | **21** | **2480,94** | **GOOD** | **-0,09** | **-0,843** | **1,44** | **0,279** | **0,399** | **13 / 61.90** | **SER 1, ASN 1, GLY 4** | **LYS 5, ARG 1, GLU 1,** | **8 / 38.10** | **outside** | **0,24** |
|  | **CECB1_CATK** | **KLGKKIEHAVLVVGYG** | **16** | **1711,08** | **GOOD** | **0,29** | **0,35** | **-0,20** | **0,416** | **0,271** | **8 / 50.00** | **HIS 1, GLY 3** | **LYS 3, GLU 1,** | **8 / 50.00** | **inside** | **0,53** |
|  | **CECB1_CATS** | **KLGKKIEHGVLVVGYG** | **16** | **1697,05** | **GOOD** | **-6,19** | **0,212** | **-0,14** | **0,396** | **0,26** | **9 / 56.25** | **HIS 1, GLY 4** | **LYS 3, GLU 1,** | **7 / 43.75** | **inside** | **0,44** |
|  | **CECB1_CATH1** | **KLGKKIEPVKNQGACGSCWTFS** | **22** | **2381,79** | **GOOD** | **71,19** | **-0,455** | **1,09** | **0,347** | **0,1** | **13 / 59.09** | **GLN 1, SER 2, THR 1, ASN 1, GLY 3** | **LYS 4, GLU 1,** | **9 / 40.91** | **inside** | **0,49** |
|  | **CECB1_CATL1** | **KLGKKIEPVKNQGQCGSCWAFS** | **22** | **2408,82** | **GOOD** | **46,04** | **-0,582** | **1,23** | **0,325** | **0,112** | **13 / 59.09** | **GLN 2, SER 2, ASN 1, GLY 3** | **LYS 4, GLU 1,** | **9 / 40.91** | **inside** | **0,49** |
|  | **CECB1_CATH2** | **KLGKKIEYWIVKNSWG** | **16** | **1949,33** | **GOOD** | **0,85** | **-0,644** | **0,86** | **0,421** | **0,214** | **9 / 56.25** | **SER 1, ASN 1, GLY 2** | **LYS 4, GLU 1,** | **7 / 43.75** | **inside** | **0,61** |
|  | **CECB1_CATL1.2** | **KLGKKIEYWLVKNSWG** | **16** | **1949,33** | **GOOD** | **13,87** | **-0,688** | **0,86** | **0,415** | **0,209** | **9 / 56.25** | **SER 1, ASN 1, GLY 2** | **LYS 4, GLU 1,** | **7 / 43.75** | **inside** | **0,48** |
|  | **CECB2_CATK** | **KIFKKIEHAVLVVGYG** | **16** | **1801,21** | **POOR** | **-3,8** | **0,594** | **-0,33** | **0,534** | **0,338** | **7 / 43.75** | **HIS 1, GLY 2** | **LYS 3, GLU 1,** | **9 / 56.25** | **inside** | **0,62** |
|  | **CECB2_CATS** | **KIFKKIEHGVLVVGYG** | **16** | **1787,18** | **GOOD** | **-10,28** | **0,456** | **-0,27** | **0,514** | **0,332** | **8 / 50.00** | **HIS 1, GLY 3** | **LYS 3, GLU 1,** | **8 / 50.00** | **inside** | **0,52** |
|  | **CECB2_CATH1** | **KIFKKIEPVKNQGACGSCWTFS** | **22** | **2471,92** | **GOOD** | **68,21** | **-0,277** | **1,00** | **0,433** | **0,134** | **12 / 54.55** | **GLN 1, SER 2, THR 1, ASN 1, GLY 2** | **LYS 4, GLU 1,** | **10 / 45.45** | **inside** | **0,56** |
|  | **CECB2_CATL1** | **KIFKKIEPVKNQGQCGSCWAFS** | **22** | **2498,94** | **GOOD** | **43,06** | **-0,405** | **1,14** | **0,411** | **0,13** | **12 / 54.55** | **GLN 2, SER 2, ASN 1, GLY 2** | **LYS 4, GLU 1,** | **10 / 45.45** | **inside** | **0,56** |
|  | **CECB2_CATH2** | **KIFKKIEYWIVKNSWG** | **16** | **2039,45** | **GOOD** | **-3,24** | **-0,4** | **0,73** | **0,539** | **0,295** | **8 / 50.00** | **SER 1, ASN 1, GLY 1** | **LYS 4, GLU 1,** | **8 / 50.00** | **inside** | **0,68** |
|  | **CECB2_CATL1.2** | **KIFKKIEYWLVKNSWG** | **16** | **2039,45** | **GOOD** | **9,78** | **-0,44** | **0,73** | **0,533** | **0,289** | **8 / 50.00** | **SER 1, ASN 1, GLY 1** | **LYS 4, GLU 1,** | **8 / 50.00** | **inside** | **0,56** |
|  | **CECB3_CATK** | **KVFKKIEHAVLVVGYG** | **16** | **1787,18** | **GOOD** | **-3,8** | **0,575** | **-0,27** | **0,497** | **0,317** | **7 / 43.75** | **HIS 1, GLY 2** | **LYS 3, GLU 1,** | **9 / 56.25** | **inside** | **0,60** |
|  | **CECB3_CATS** | **KVFKKIEHGVLVVGYG** | **16** | **1773,15** | **GOOD** | **-10,28** | **0,438** | **-0,22** | **0,478** | **0,313** | **8 / 50.00** | **HIS 1, GLY 3** | **LYS 3, GLU 1,** | **8 / 50.00** | **inside** | **0,51** |
|  | **CECB3_CATH1** | **KVFKKIEPVKNQGACGSCWTFS** | **22** | **2457,89** | **GOOD** | **68,21** | **-0,291** | **1,04** | **0,406** | **0,118** | **12 / 54.55** | **GLN 1, SER 2, THR 1, ASN 1, GLY 2** | **LYS 4, GLU 1,** | **10 / 45.45** | **inside** | **0,56** |
|  | **CECB3_CATL1** | **KVFKKIEPVKNQGQCGSCWAFS** | **22** | **2424,92** | **GOOD** | **43,06** | **-0,418** | **1,18** | **0,385** | **0,11** | **12 / 54.55** | **GLN 2, SER 2, ASN 1, GLY 2** | **LYS 4, GLU 1,** | **10 / 45.45** | **inside** | **0,56** |
|  | **CECB3_CATH2** | **KVFKKIEYWIVKNSWG** | **16** | **2025,42** | **GOOD** | **-3,24** | **-0,419** | **0,79** | **0,503** | **0,279** | **8 / 50.00** | **SER 1, ASN 1, GLY 1** | **LYS 4, GLU 1,** | **8 / 50.00** | **inside** | **0,67** |
|  | **CECB3_CATL1.2** | **KVFKKIEYWLVKNSWG** | **16** | **2025,42** | **GOOD** | **9,78** | **-0,463** | **0,79** | **0,497** | **0,273** | **8 / 50.00** | **SER 1, ASN 1, GLY 1** | **LYS 4, GLU 1,** | **8 / 50.00** | **inside** | **0,55** |
|  | **CECC1_CATK** | **KLGKRIERIGQHAVLVVGYG** | **20** | **2193,62** | **GOOD** | **26,55** | **0,055** | **1,03** | **0,36** | **0,341** | **11 / 55.00** | **GLN 1, HIS 1, GLY 4** | **LYS 2, ARG 2, GLU 1,** | **9 / 45.00** | **inside** | **0,60** |
|  | **CECC1_CATS** | **KLGKRIERIGQHGVLVVGYG** | **20** | **2179,6** | **GOOD** | **21,36** | **-0,055** | **1,07** | **0,344** | **0,326** | **12 / 60.00** | **GLN 1, HIS 1, GLY 5** | **LYS 2, ARG 2, GLU 1,** | **8 / 40.00** | **inside** | **0,60** |
|  | **CECC1_CATH1** | **KLGKRIERIGQPVKNQGACGSCWTFS** | **26** | **2864,34** | **GOOD** | **77,58** | **-0,558** | **1,85** | **0,315** | **0,254** | **16 / 61.54** | **GLN 2, SER 2, THR 1, ASN 1, GLY 4** | **LYS 3, ARG 2, GLU 1,** | **10 / 38.46** | **inside** | **0,58** |
|  | **CECC1_CATL1** | **KLGKRIERIGQPVKNQGQCGSCWAFS** | **26** | **2891,36** | **GOOD** | **56,3** | **-0,665** | **1,96** | **0,296** | **0,275** | **16 / 61.54** | **GLN 3, SER 2, ASN 1, GLY 4** | **LYS 3, ARG 2, GLU 1,** | **10 / 38.46** | **inside** | **0,58** |
|  | **CECC1_CATH2** | **KLGKRIERIGQYWIVKNSWG** | **20** | **2431,87** | **GOOD** | **19,46** | **-0,74** | **1,89** | **0,364** | **0,293** | **12 / 60.00** | **GLN 1, SER 1, ASN 1, GLY 3** | **LYS 3, ARG 2, GLU 1,** | **8 / 40.00** | **inside** | **0,64** |
|  | **CECC1_CATL1.2** | **KLGKRIERIGQYWLVKNSWG** | **20** | **2431,87** | **GOOD** | **29,87** | **-0,775** | **1,89** | **0,359** | **0,294** | **12 / 60.00** | **GLN 1, SER 1, ASN 1, GLY 3** | **LYS 3, ARG 2, GLU 1,** | **8 / 40.00** | **inside** | **0,63** |
|  | **CECC2_CATK** | **KLGKKLEGAGKHAVLVVGYG** | **20** | **2024,44** | **GOOD** | **-24,46** | **0,1** | **-0,06** | **0,293** | **0,277** | **11 / 55.00** | **HIS 1, GLY 5** | **LYS 4, GLU 1,** | **9 / 45.00** | **outside** | **0,27** |
|  | **CECC2_CATS** | **KLGKKLEGAGKHGVLVVGYG** | **20** | **2010,41** | **GOOD** | **-29,65** | **-0,01** | **-0,02** | **0,278** | **0,262** | **12 / 60.00** | **HIS 1, GLY 6** | **LYS 4, GLU 1,** | **8 / 40.00** | **outside** | **0,27** |
|  | **CECC2_CATH1** | **KLGKKLEGAGKPVKNQGACGSCWTFS** | **26** | **2695,15** | **GOOD** | **28,03** | **-0,523** | **1,00** | **0,263** | **0,209** | **16 / 61.54** | **GLN 1, SER 2, THR 1, ASN 1, GLY 5** | **LYS 5, GLU 1,** | **10 / 38.46** | **outside** | **0,25** |
|  | **CECC2_CATL1** | **KLGKKLEGAGKPVKNQGQCGSCWAFS** | **26** | **2722,18** | **GOOD** | **6,75** | **-0,631** | **1,11** | **0,245** | **0,23** | **16 / 61.54** | **GLN 2, SER 2, ASN 1, GLY 5** | **LYS 5, GLU 1,** | **10 / 38.46** | **outside** | **0,25** |
|  | **CECC2_CATH2** | **KLGKKLEGAGKYWIVKNSWG** | **20** | **2262,68** | **GOOD** | **-27,78** | **-0,695** | **0,78** | **0,298** | **0,229** | **12 / 60.00** | **SER 1, ASN 1, GLY 4** | **LYS 5, GLU 1,** | **8 / 40.00** | **outside** | **0,33** |
|  | **CECC2_CATL1.2** | **KLGKKLEGAGKYWLVKNSWG** | **20** | **2262,68** | **GOOD** | **-17,36** | **-0,73** | **0,78** | **0,293** | **0,231** | **12 / 60.00** | **SER 1, ASN 1, GLY 4** | **LYS 5, GLU 1,** | **8 / 40.00** | **outside** | **0,31** |
|  | **CECD1_CATK** | **GQRIRDAIISAAPAVHAVLVVGYG** | **24** | **2433,84** | **POOR** | **15,02** | **0,792** | **0,19** | **0,512** | **0,218** | **9 / 37.50** | **GLN 1, HIS 1, SER 1, GLY 3** | **ARG 2, ASP 1,** | **15 / 62.50** | **inside** | **0,64** |
|  | **CECD1_CATS** | **GQRIRDAIISAAPAVHGVLVVGYG** | **24** | **2419,81** | **POOR** | **10,7** | **0,7** | **0,22** | **0,499** | **0,231** | **10 / 41.67** | **GLN 1, HIS 1, SER 1, GLY 4** | **ARG 2, ASP 1,** | **14 / 58.33** | **inside** | **0,64** |
|  | **CECD1_CATH1** | **GQRIRDAIISAAPAVPVKNQGACGSCWTFS** | **30** | **3104,55** | **POOR** | **61,55** | **0,113** | **1,06** | **0,442** | **0,21** | **14 / 46.67** | **GLN 2, SER 3, THR 1, ASN 1, GLY 3** | **LYS 1, ARG 2, ASP 1,** | **16 / 53.33** | **inside** | **0,62** |
|  | **CECD1_CATL1** | **GQRIRDAIISAAPAVPVKNQGQCGSCWAFS** | **30** | **3131,58** | **POOR** | **43,11** | **0,02** | **1,16** | **0,426** | **0,201** | **14 / 46.67** | **GLN 3, SER 3, ASN 1, GLY 3** | **LYS 1, ARG 2, ASP 1,** | **16 / 53.33** | **inside** | **0,62** |
|  | **CECD1_CATH2** | **GQRIRDAIISAAPAVYWIVKNSWG** | **24** | **2672,08** | **POOR** | **9,11** | **0,129** | **0,90** | **0,516** | **0,259** | **10 / 41.67** | **GLN 1, SER 2, ASN 1, GLY 2** | **LYS 1, ARG 2, ASP 1,** | **14 / 58.33** | **inside** | **0,64** |
|  | **CECD1_CATL1.2** | **GQRIRDAIISAAPAVYWLVKNSWG** | **24** | **2672,08** | **POOR** | **17,79** | **0,1** | **0,90** | **0,512** | **0,259** | **10 / 41.67** | **GLN 1, SER 2, ASN 1, GLY 2** | **LYS 1, ARG 2, ASP 1,** | **14 / 58.33** | **inside** | **0,63** |
|  | **CECD2_CATK** | **GQRVRDAVISAGPAVHAVLVVGYG** | **24** | **2391,76** | **POOR** | **7** | **0,675** | **0,30** | **0,451** | **0,166** | **10 / 41.67** | **GLN 1, HIS 1, SER 1, GLY 4** | **ARG 2, ASP 1,** | **14 / 58.33** | **inside** | **0,64** |
|  | **CECD2_CATS** | **GQRVRDAVISAGPAVHGVLVVGYG** | **24** | **2377,73** | **POOR** | **2,68** | **0,583** | **0,33** | **0,438** | **0,179** | **11 / 45.83** | **GLN 1, HIS 1, SER 1, GLY 5** | **ARG 2, ASP 1,** | **13 / 54.17** | **inside** | **0,63** |
|  | **CECD2_CATH1** | **GQRVRDAVISAGPAVPVKNQGACGSCWTFS** | **30** | **3062,47** | **POOR** | **55,13** | **0,02** | **1,15** | **0,393** | **0,175** | **15 / 50.00** | **GLN 2, SER 3, THR 1, ASN 1, GLY 4** | **LYS 1, ARG 2, ASP 1,** | **15 / 50.00** | **inside** | **0,63** |
|  | **CECD2_CATL1** | **GQRVRDAVISAGPAVPVKNQGQCGSCWAFS** | **30** | **3089,5** | **POOR** | **36,69** | **-0,073** | **1,25** | **0,377** | **0,168** | **15 / 50.00** | **GLN 3, SER 3, ASN 1, GLY 4** | **LYS 1, ARG 2, ASP 1,** | **15 / 50.00** | **inside** | **0,62** |
|  | **CECD2_CATH2** | **GQRVRDAVISAGPAVYWIVKNSWG** | **24** | **2630** | **POOR** | **1,09** | **0,013** | **1,01** | **0,455** | **0,208** | **11 / 45.83** | **GLN 1, SER 2, ASN 1, GLY 3** | **LYS 1, ARG 2, ASP 1,** | **13 / 54.17** | **inside** | **0,64** |
|  | **CECD2_CATL1.2** | **GQRVRDAVISAGPAVYWLVKNSWG** | **24** | **2630** | **POOR** | **9,77** | **-0,017** | **1,01** | **0,45** | **0,208** | **11 / 45.83** | **GLN 1, SER 2, ASN 1, GLY 3** | **LYS 1, ARG 2, ASP 1,** | **13 / 54.17** | **inside** | **0,63** |
|  | **CECD3_CATK** | **GQRVRDAIISAGPAVHAVLVVGYG** | **24** | **2405,79** | **POOR** | **7** | **0,688** | **0,26** | **0,475** | **0,19** | **10 / 41.67** | **GLN 1, HIS 1, SER 1, GLY 4** | **ARG 2, ASP 1,** | **14 / 58.33** | **inside** | **0,64** |
|  | **CECD3_CATS** | **GQRVRDAIISAGPAVHGVLVVGYG** | **24** | **2391,76** | **POOR** | **2,68** | **0,596** | **0,30** | **0,462** | **0,203** | **11 / 45.83** | **GLN 1, HIS 1, SER 1, GLY 5** | **ARG 2, ASP 1,** | **13 / 54.17** | **inside** | **0,64** |
|  | **CECD3_CATH1** | **GQRVRDAIISAGPAVPVKNQGACGSCWTFS** | **30** | **3076,5** | **POOR** | **55,13** | **0,03** | **1,12** | **0,413** | **0,192** | **15 / 50.00** | **GLN 2, SER 3, THR 1, ASN 1, GLY 4** | **LYS 1, ARG 2, ASP 1,** | **15 / 50.00** | **inside** | **0,62** |
|  | **CECD3_CATL1** | **GQRVRDAIISAGPAVPVKNQGQCGSCWAFS** | **30** | **3103,52** | **POOR** | **36,96** | **-0,063** | **1,22** | **0,397** | **0,184** | **15 / 50.00** | **GLN 3, SER 3, ASN 1, GLY 4** | **LYS 1, ARG 2, ASP 1,** | **15 / 50.00** | **inside** | **0,62** |
|  | **CECD3_CATH2** | **GQRVRDAIISAGPAVYWIVKNSWG** | **24** | **2644,03** | **POOR** | **1,09** | **0,025** | **0,97** | **0,479** | **0,232** | **11 / 45.83** | **GLN 1, SER 2, ASN 1, GLY 3** | **LYS 1, ARG 2, ASP 1,** | **13 / 54.17** | **inside** | **0,64** |
|  | **CECD3_CATL1.2** | **GQRVRDAIISAGPAVYWLVKNSWG** | **24** | **2644,03** | **POOR** | **9,77** | **-0,004** | **0,97** | **0,475** | **0,232** | **11 / 45.83** | **GLN 1, SER 2, ASN 1, GLY 3** | **LYS 1, ARG 2, ASP 1,** | **13 / 54.17** | **inside** | **0,63** |
|  |  |  |  |  |  |  |  |  |  |  |  |  |  |  |  |  |
| **CATHEPSINS N TERMINAL** | **CATK_CEC1** | **HAVLVVGYGKKIERVGQNTRDA** | **22** | **2410,76** | **GOOD** | **2,5** | **-0,386** | **1,98** | **0,187** | **0,311** | **13 / 59.09** | **GLN 1, HIS 1, THR 1, ASN 1, GLY 3** | **LYS 2, ARG 2, GLU 1, ASP 1,** | **9 / 40.91** | **outside** | **0,28** |
|  | **CATS_CEC1** | **HGVLVVGYGKKIERVGQNTRDA** | **22** | **2396,73** | **GOOD** | **-2,21** | **-0,486** | **2,02** | **0,173** | **0,314** | **14 / 63.64** | **GLN 1, HIS 1, THR 1, ASN 1, GLY 4** | **LYS 2, ARG 2, GLU 1, ASP 1,** | **8 / 36.36** | **outside** | **0,21** |
|  | **CATH1_CEC1** | **PVKNQGACGSCWTFSKKIERVGQNTRDA** | **28** | **3081,47** | **GOOD** | **51,2** | **-0,861** | **2,53** | **0,182** | **0,217** | **18 / 64.29** | **GLN 2, SER 2, THR 2, ASN 2, GLY 3** | **LYS 3, ARG 2, GLU 1, ASP 1,** | **10 / 35.71** | **inside** | **0,42** |
|  | **CATL1_CEC1** | **PVKNQGQCGSCWAFSKKIERVGQNTRDA** | **28** | **3108,5** | **GOOD** | **31,44** | **-0,961** | **2,64** | **0,165** | **0,2** | **18 / 64.29** | **GLN 3, SER 2, THR 1, ASN 2, GLY 3** | **LYS 3, ARG 2, GLU 1, ASP 1,** | **10 / 35.71** | **inside** | **0,48** |
|  | **CATH2_CEC1** | **YWIVKNSWGKKIERVGQNTRDA** | **22** | **2649,01** | **GOOD** | **-0,51** | **-1,109** | **2,75** | **0,191** | **0,342** | **14 / 63.64** | **GLN 1, SER 1, THR 1, ASN 2, GLY 2** | **LYS 3, ARG 2, GLU 1, ASP 1,** | **8 / 36.36** | **inside** | **0,61** |
|  | **CATL1.2_CEC1** | **YWLVKNSWGKKIERVGQNTRDA** | **22** | **2649,01** | **GOOD** | **8,95** | **-1,141** | **2,75** | **0,187** | **0,346** | **14 / 63.64** | **GLN 1, SER 1, THR 1, ASN 2, GLY 2** | **LYS 3, ARG 2, GLU 1, ASP 1,** | **8 / 36.36** | **inside** | **0,47** |
|  | **CATK_CEC2** | **HAVLVVGYGKKIEKAVRRVRDG** | **22** | **2450,92** | **GOOD** | **36,26** | **-0,227** | **2,05** | **0,177** | **0,392** | **12 / 54.55** | **HIS 1, GLY 3** | **LYS 3, ARG 3, GLU 1, ASP 1,** | **10 / 45.45** | **outside** | **0,31** |
|  | **CATS_CEC2** | **HGVLVVGYGKKIEKAVRRVRDG** | **22** | **2436,89** | **GOOD** | **31,55** | **-0,327** | **2,09** | **0,163** | **0,391** | **13 / 59.09** | **HIS 1, GLY 4** | **LYS 3, ARG 3, GLU 1, ASP 1,** | **9 / 40.91** | **outside** | **0,23** |
|  | **CATH1_CEC2** | **PVKNQGACGSCWTFSKKIEKAVRRVRDG** | **28** | **3121,63** | **GOOD** | **77,72** | **-0,736** | **2,59** | **0,174** | **0,304** | **17 / 60.71** | **GLN 1, SER 2, THR 1, ASN 1, GLY 3** | **LYS 4, ARG 3, GLU 1, ASP 1,** | **11 / 39.29** | **inside** | **0,46** |
|  | **CATL1_CEC2** | **PVKNQGQCGSCWAFSKKIEKAVRRVRDG** | **28** | **3148,65** | **GOOD** | **57,96** | **-0,836** | **2,70** | **0,157** | **0,288** | **17 / 60.71** | **GLN 2, SER 2, ASN 1, GLY 3** | **LYS 4, ARG 3, GLU 1, ASP 1,** | **11 / 39.29** | **inside** | **0,51** |
|  | **CATH2_CEC2** | **YWIVKNSWGKKIEKAVRRVRDG** | **22** | **2689,16** | **GOOD** | **33,24** | **-0,95** | **2,83** | **0,181** | **0,413** | **13 / 59.09** | **SER 1, ASN 1, GLY 2** | **LYS 4, ARG 3, GLU 1, ASP 1,** | **9 / 40.91** | **inside** | **0,65** |
|  | **CATL1.2_CEC2** | **YWLVKNSWGKKIEKAVRRVRDG** | **22** | **2689,16** | **GOOD** | **42,71** | **-0,982** | **2,83** | **0,177** | **0,417** | **13 / 59.09** | **SER 1, ASN 1, GLY 2** | **LYS 4, ARG 3, GLU 1, ASP 1,** | **9 / 40.91** | **inside** | **0,51** |
|  | **CATK_CEC3** | **HAVLVVGYGKKIEKVGQNIRDG** | **22** | **2380,78** | **GOOD** | **22,48** | **-0,223** | **1,25** | **0,244** | **0,374** | **13 / 59.09** | **GLN 1, HIS 1, ASN 1, GLY 4** | **LYS 3, ARG 1, GLU 1, ASP 1,** | **9 / 40.91** | **outside** | **0,26** |
|  | **CATS_CEC3** | **HGVLVVGYGKKIEKVGQNIRDG** | **22** | **2366,75** | **GOOD** | **17,76** | **-0,323** | **1,29** | **0,230** | **0,376** | **14 / 63.64** | **GLN 1, HIS 1, ASN 1, GLY 5** | **LYS 3, ARG 1, GLU 1, ASP 1,** | **8 / 36.36** | **outside** | **0,19** |
|  | **CATH1_CEC3** | **PVKNQGACGSCWTFSKKIEKVGQNIRDG** | **28** | **3051,49** | **GOOD** | **66,89** | **-0,732** | **1,96** | **0,227** | **0,264** | **18 / 64.29** | **GLN 2, SER 2, THR 1, ASN 2, GLY 4** | **LYS 4, ARG 1, GLU 1, ASP 1,** | **10 / 35.71** | **outside** | **0,40** |
|  | **CATL1_CEC3** | **PVKNQGQCGSCWAFSKKIEKVGQNIRDG** | **28** | **3078,51** | **GOOD** | **47,13** | **-0,832** | **2,07** | **0,210** | **0,246** | **18 / 64.29** | **GLN 3, SER 2, ASN 2, GLY 4** | **LYS 4, ARG 1, GLU 1, ASP 1,** | **10 / 35.71** | **inside** | **0,46** |
|  | **CATH2_CEC3** | **YWIVKNSWGKKIEKVGQNIRDG** | **22** | **2619,02** | **GOOD** | **19,46** | **-0,945** | **2,03** | **0,248** | **0,403** | **14 / 63.64** | **GLN 1, SER 1, ASN 2, GLY 3** | **LYS 4, ARG 1, GLU 1, ASP 1,** | **8 / 36.36** | **inside** | **0,59** |
|  | **CATL1.2_CEC3** | **YWLVKNSWGKKIEKVGQNIRDG** | **22** | **2619,02** | **GOOD** | **28,93** | **-0,977** | **2,03** | **0,244** | **0,407** | **14 / 63.64** | **GLN 1, SER 1, ASN 2, GLY 3** | **LYS 4, ARG 1, GLU 1, ASP 1,** | **8 / 36.36** | **inside** | **0,45** |
|  | **CATK_CEC4** | **HAVLVVGYGKKIGRVGQHTRDA** | **22** | **2361,74** | **GOOD** | **-17,04** | **-0,232** | **1,53** | **0,250** | **0,291** | **13 / 59.09** | **GLN 1, HIS 2, THR 1, GLY 4** | **LYS 2, ARG 2, ASP 1,** | **9 / 40.91** | **outside** | **0,27** |
|  | **CATS_CEC4** | **HGVLVVGYGKKIGRVGQHTRDA** | **22** | **2347,71** | **GOOD** | **-21,75** | **-0,332** | **1,57** | **0,235** | **0,294** | **14 / 63.64** | **GLN 1, HIS 2, THR 1, GLY 5** | **LYS 2, ARG 2, ASP 1,** | **8 / 36.36** | **outside** | **0,20** |
|  | **CATH1_CEC4** | **PVKNQGACGSCWTFSKKIGRVGQHTRDA** | **28** | **3032,45** | **GOOD** | **35,84** | **-0,739** | **2,18** | **0,231** | **0,201** | **18 / 64.29** | **GLN 2, HIS 1, SER 2, THR 2, ASN 1, GLY 4** | **LYS 3, ARG 2, ASP 1,** | **10 / 35.71** | **inside** | **0,41** |
|  | **CATL1_CEC4** | **PVKNQGQCGSCWAFSKKIGRVGQHTRDA** | **28** | **3059,47** | **GOOD** | **16,08** | **-0,39** | **2,29** | **0,214** | **0,184** | **18 / 64.29** | **GLN 3, HIS 1, SER 2, THR 1, ASN 1, GLY 4** | **LYS 3, ARG 2, ASP 1,** | **10 / 35.71** | **inside** | **0,47** |
|  | **CATH2_CEC4** | **YWIVKNSWGKKIGRVGQHTRDA** | **22** | **2599,98** | **GOOD** | **-20,05** | **-0,955** | **2,31** | **0,254** | **0,323** | **14 / 63.64** | **GLN 1, HIS 1, SER 1, THR 1, ASN 1, GLY 3** | **LYS 3, ARG 2, ASP 1,** | **8 / 36.36** | **inside** | **0,60** |
|  | **CATL1.2_CEC4** | **YWLVKNSWGKKIGRVGQHTRDA** | **22** | **2599,98** | **GOOD** | **-10,59** | **-0,986** | **2,31** | **0,249** | **0,327** | **14 / 63.64** | **GLN 1, HIS 1, SER 1, THR 1, ASN 1, GLY 3** | **LYS 3, ARG 2, ASP 1,** | **8 / 36.36** | **inside** | **0,46** |
|  | **CATK_CECA1** | **HAVLVVGYGKLGKKLEGAGKR** | **21** | **2180,62** | **GOOD** | **-11,34** | **-0,119** | **0,64** | **0,231** | **0,34** | **12 / 57.14** | **HIS 1, GLY 5** | **LYS 4, ARG 1, GLU 1,** | **9 / 42.86** | **outside** | **0,29** |
|  | **CATS_CECA1** | **HGVLVVGYGKLGKKLEGAGKR** | **21** | **2166,6** | **GOOD** | **-16,28** | **-0,224** | **0,68** | **0,217** | **0,352** | **13 / 61.90** | **HIS 1, GLY 6** | **LYS 4, ARG 1, GLU 1,** | **8 / 38.10** | **outside** | **0,21** |
|  | **CATH1_CECA1** | **PVKNQGACGSCWTFSKLGKKLEGAGKR** | **27** | **2851,34** | **GOOD** | **42,23** | **-0,67** | **1,51** | **0,216** | **0,14** | **17 / 62.96** | **GLN 1, SER 2, THR 1, ASN 1, GLY 5** | **LYS 5, ARG 1, GLU 1,** | **10 / 37.04** | **inside** | **0,43** |
|  | **CATL1_CECA1** | **PVKNQGQCGSCWAFSKLGKKLEGAGKR** | **27** | **2878,36** | **GOOD** | **21,74** | **-0,774** | **1,62** | **0,199** | **0,122** | **17 / 62.96** | **GLN 2, SER 2, ASN 1, GLY 5** | **LYS 5, ARG 1, GLU 1,** | **10 / 37.04** | **inside** | **0,49** |
|  | **CATH2_CECA1** | **YWIVKNSWGKLGKKLEGAGKR** | **21** | **2418,87** | **GOOD** | **-14,5** | **-0,876** | **1,45** | **0,236** | **0,391** | **13 / 61.90** | **SER 1, ASN 1, GLY 4** | **LYS 5, ARG 1, GLU 1,** | **8 / 38.10** | **inside** | **0,62** |
|  | **CATL1.2_CECA1** | **YWLVKNSWGKLGKKLEGAGKR** | **21** | **2418,87** | **GOOD** | **-4,58** | **-0,91** | **1,45** | **0,231** | **0,393** | **13 / 61.90** | **SER 1, ASN 1, GLY 4** | **LYS 5, ARG 1, GLU 1,** | **8 / 38.10** | **inside** | **0,48** |
|  | **CATK_CECA2** | **HAVLVVGYGKIGKKIERVGQH** | **21** | **2288,72** | **GOOD** | **-1,9** | **-0,052** | **0,80** | **0,327** | **0,365** | **12 / 57.14** | **GLN 1, HIS 2, GLY 4** | **LYS 3, ARG 1, GLU 1,** | **9 / 42.86** | **outside** | **0,27** |
|  | **CATS_CECA2** | **HGVLVVGYGKIGKKIERVGQH** | **21** | **2274,7** | **GOOD** | **-6,83** | **-0,157** | **0,84** | **0,312** | **0,378** | **13 / 61.90** | **GLN 1, HIS 2, GLY 5** | **LYS 3, ARG 1, GLU 1,** | **8 / 38.10** | **outside** | **0,20** |
|  | **CATH1_CECA2** | **PVKNQGACGSCWTFSKIGKKIERVGQH** | **27** | **2959,44** | **GOOD** | **49,58** | **-0,619** | **1,64** | **0,291** | **0,149** | **17 / 62.96** | **GLN 2, HIS 1, SER 2, THR 1, ASN 1, GLY 4** | **LYS 4, ARG 1, GLU 1,** | **10 / 37.04** | **outside** | **0,41** |
|  | **CATL1_CECA2** | **PVKNQGQCGSCWAFSKIGKKIERVGQH** | **27** | **2986,46** | **GOOD** | **29,09** | **-0,722** | **1,75** | **0,273** | **0,135** | **17 / 62.96** | **GLN 3, HIS 1, SER 2, ASN 1, GLY 4** | **LYS 4, ARG 1, GLU 1,** | **10 / 37.04** | **inside** | **0,47** |
|  | **CATH2_CECA2** | **YWIVKNSWGKIGKKIERVGQH** | **21** | **2526,97** | **GOOD** | **-5,06** | **-0,81** | **1,61** | **0,331** | **0,417** | **13 / 61.90** | **GLN 1, HIS 1, SER 1, ASN 1, GLY 3** | **LYS 4, ARG 1, GLU 1,** | **8 / 38.10** | **inside** | **0,60** |
|  | **CATL1.2_CECA2** | **YWLVKNSWGKIGKKIERVGQH** | **21** | **2526,97** | **GOOD** | **4,86** | **-0,843** | **1,61** | **0,327** | **0,418** | **13 / 61.90** | **GLN 1, HIS 1, SER 1, ASN 1, GLY 3** | **LYS 4, ARG 1, GLU 1,** | **8 / 38.10** | **inside** | **0,46** |
|  | **CATK_CECA3** | **HAVLVVGYGKVFKKIEKVGRN** | **21** | **2341,83** | **GOOD** | **-3,18** | **0,052** | **0,84** | **0,313** | **0,428** | **11 / 52.38** | **HIS 1, ASN 1, GLY 3** | **LYS 4, ARG 1, GLU 1,** | **10 / 47.62** | **outside** | **0,30** |
|  | **CATS_CECA3** | **HGVLVVGYGKVFKKIEKVGRN** | **21** | **2327,8** | **GOOD** | **-8,11** | **-0,052** | **0,88** | **0,313** | **0,428** | **11 / 52.38** | **HIS 1, ASN 1, GLY 3** | **LYS 4, ARG 1, GLU 1,** | **10 / 47.62** | **outside** | **0,22** |
|  | **CATH1_CECA3** | **PVKNQGACGSCWTFSKVFKKIEKVGRN** | **27** | **3012,54** | **GOOD** | **48,58** | **-0,537** | **1,67** | **0,280** | **0,216** | **16 / 59.26** | **GLN 1, SER 2, THR 1, ASN 2, GLY 3** | **LYS 5, ARG 1, GLU 1,** | **11 / 40.74** | **inside** | **0,45** |
|  | **CATL1_CECA3** | **PVKNQGQCGSCWAFSKVFKKIEKVGRN** | **27** | **3039,57** | **GOOD** | **28,09** | **-0,641** | **1,78** | **0,262** | **0,197** | **16 / 59.26** | **GLN 2, SER 2, ASN 2, GLY 3** | **LYS 5, ARG 1, GLU 1,** | **11 / 40.74** | **inside** | **0,50** |
|  | **CATH2_CECA3** | **YWIVKNSWGKVFKKIEKVGRN** | **21** | **2580,07** | **GOOD** | **-6,34** | **-0,705** | **1,65** | **0,318** | **0,477** | **12 / 57.14** | **SER 1, ASN 2, GLY 2** | **LYS 5, ARG 1, GLU 1,** | **9 / 42.86** | **inside** | **0,64** |
|  | **CATL1.2_CECA3** | **YWLVKNSWGKVFKKIEKVGRN** | **21** | **2580,07** | **GOOD** | **3,58** | **-0,738** | **1,65** | **0,313** | **0,48** | **12 / 57.14** | **SER 1, ASN 2, GLY 2** | **LYS 5, ARG 1, GLU 1,** | **9 / 42.86** | **inside** | **0,50** |
|  | **CATK_CECA4** | **HAVLVVGYGKFGKKLEGVGKR** | **21** | **2242,7** | **GOOD** | **-7,3** | **-0,052** | **0,63** | **0,279** | **0,369** | **12 / 57.14** | **HIS 1, GLY 5** | **LYS 4, ARG 1, GLU 1,** | **9 / 42.86** | **outside** | **0,28** |
|  | **CATS_CECA4** | **HGVLVVGYGKFGKKLEGVGKR** | **21** | **2228,67** | **GOOD** | **-12,23** | **-0,157** | **0,67** | **0,264** | **0,382** | **13 / 61.90** | **HIS 1, GLY 6** | **LYS 4, ARG 1, GLU 1,** | **8 / 38.10** | **outside** | **0,21** |
|  | **CATH1_CECA4** | **PVKNQGACGSCWTFSKFGKKLEGVGKR** | **27** | **2913,41** | **GOOD** | **45,38** | **-0,619** | **1,50** | **0,253** | **0,153** | **17 / 62.96** | **GLN 1, SER 2, THR 1, ASN 1, GLY 5** | **LYS 5, ARG 1, GLU 1,** | **10 / 37.04** | **inside** | **0,43** |
|  | **CATL1_CECA4** | **PVKNQGQCGSCWAFSKFGKKLEGVGKR** | **27** | **2940,43** | **GOOD** | **24,89** | **-0,722** | **1,61** | **0,236** | **0,138** | **17 / 62.96** | **GLN 2, SER 2, ASN 1, GLY 5** | **LYS 5, ARG 1, GLU 1,** | **10 / 37.04** | **inside** | **0,48** |
|  | **CATH2_CECA4** | **YWIVKNSWGKFGKKLEGVGKR** | **21** | **2480,94** | **GOOD** | **-10,46** | **-0,81** | **1,44** | **0,283** | **0,42** | **13 / 61.90** | **SER 1, ASN 1, GLY 4** | **LYS 5, ARG 1, GLU 1,** | **8 / 38.10** | **inside** | **0,61** |
|  | **CATL1.2_CECA4** | **YWLVKNSWGKFGKKLEGVGKR** | **21** | **2480,94** | **GOOD** | **-0,54** | **-0,843** | **1,44** | **0,279** | **0,422** | **13 / 61.90** | **SER 1, ASN 1, GLY 4** | **LYS 5, ARG 1, GLU 1,** | **8 / 38.10** | **inside** | **0,47** |
|  | **CATK_CECB1** | **HAVLVVGYGKLGKKIE** | **16** | **1711,08** | **GOOD** | **-0,31** | **0,35** | **-0,20** | **0,416** | **0,364** | **8 / 50.00** | **HIS 1, GLY 3** | **LYS 3, GLU 1,** | **8 / 50.00** | **outside** | **0,32** |
|  | **CATS_CECB1** | **HGVLVVGYGKLGKKIE** | **16** | **1697,05** | **GOOD** | **-6,79** | **0,212** | **-0,14** | **0,396** | **0,38** | **9 / 56.25** | **HIS 1, GLY 4** | **LYS 3, GLU 1,** | **7 / 43.75** | **outside** | **0,24** |
|  | **CATH1_CECB1** | **PVKNQGACGSCWTFSKLGKKIE** | **22** | **2381,79** | **GOOD** | **62,43** | **-0,455** | **1,09** | **0,347** | **0,116** | **13 / 59.09** | **GLN 1, SER 2, THR 1, ASN 1, GLY 3** | **LYS 4, GLU 1,** | **9 / 40.91** | **inside** | **0,41** |
|  | **CATL1_CECB1** | **PVKNQGQCGSCWAFSKLGKKIE** | **22** | **2408,82** | **GOOD** | **37,28** | **-0,582** | **1,23** | **0,325** | **0,092** | **13 / 59.09** | **GLN 2, SER 2, ASN 1, GLY 3** | **LYS 4, GLU 1,** | **9 / 40.91** | **inside** | **0,47** |
|  | **CATH2_CECB1** | **YWIVKNSWGKLGKKIE** | **16** | **1949,33** | **GOOD** | **-4,46** | **-0,644** | **0,86** | **0,421** | **0,431** | **9 / 56.25** | **SER 1, ASN 1, GLY 2** | **LYS 4, GLU 1,** | **7 / 43.75** | **inside** | **0,65** |
|  | **CATL1.2_CECB1** | **YWLVKNSWGKLGKKIE** | **16** | **1949,33** | **GOOD** | **8,56** | **-0,688** | **0,86** | **0,415** | **0,433** | **9 / 56.25** | **SER 1, ASN 1, GLY 2** | **LYS 4, GLU 1,** | **7 / 43.75** | **inside** | **0,51** |
|  | **CATK_CECB2** | **HAVLVVGYGKIFKKIE** | **16** | **1801,21** | **POOR** | **-4,39** | **0,594** | **-0,33** | **0,534** | **0,431** | **7 / 43.75** | **HIS 1, GLY 2** | **LYS 3, GLU 1,** | **9 / 56.25** | **inside** | **0,38** |
|  | **CATS_CECB2** | **HGVLVVGYGKIFKKIE** | **16** | **1787,18** | **GOOD** | **-10,88** | **0,456** | **-0,27** | **0,514** | **0,444** | **8 / 50.00** | **HIS 1, GLY 3** | **LYS 3, GLU 1,** | **8 / 50.00** | **outside** | **0,29** |
|  | **CATH1_CECB2** | **PVKNQGACGSCWTFSKIFKKIE** | **22** | **2471,92** | **GOOD** | **59,18** | **-0,177** | **1,00** | **0,433** | **0,19** | **12 / 54.55** | **GLN 1, SER 2, THR 1, ASN 1, GLY 2** | **LYS 4, GLU 1,** | **10 / 45.45** | **inside** | **0,43** |
|  | **CATL1_CECB2** | **PVKNQGQCGSCWAFSKIFKKIE** | **22** | **2498,94** | **GOOD** | **34,31** | **-0,405** | **1,14** | **0,411** | **0,165** | **12 / 54.55** | **GLN 2, SER 2, ASN 1, GLY 2** | **LYS 4, GLU 1,** | **10 / 45.45** | **inside** | **0,49** |
|  | **CATH2_CECB2** | **YWIVKNSWGKIFKKIE** | **16** | **2039,45** | **GOOD** | **-8,54** | **-0,4** | **0,73** | **0,539** | **0,494** | **8 / 50.00** | **SER 1, ASN 1, GLY 1** | **LYS 4, GLU 1,** | **8 / 50.00** | **inside** | **0,71** |
|  | **CATL1.2_CECB2** | **YWLVKNSWGKIFKKIE** | **16** | **2039,45** | **GOOD** | **4,47** | **-0,444** | **0,73** | **0,533** | **0,497** | **8 / 50.00** | **SER 1, ASN 1, GLY 1** | **LYS 4, GLU 1,** | **8 / 50.00** | **inside** | **0,57** |
|  | **CATK_CECB3** | **HAVLVVGYGKVFKKIE** | **16** | **1787,18** | **GOOD** | **-4,39** | **0,575** | **-0,27** | **0,497** | **0,409** | **7 / 43.75** | **HIS 1, GLY 2** | **LYS 3, GLU 1,** | **9 / 56.25** | **outside** | **0,35** |
|  | **CATS_CECB3** | **HGVLVVGYGKVFKKIE** | **16** | **1773,15** | **GOOD** | **-10,88** | **0,438** | **-0,22** | **0,478** | **0,42** | **8 / 50.00** | **HIS 1, GLY 3** | **LYS 3, GLU 1,** | **8 / 50.00** | **outside** | **0,27** |
|  | **CATH1_CECB3** | **PVKNQGACGSCWTFSKVFKKIE** | **22** | **2457,89** | **GOOD** | **59,46** | **-0,291** | **1,04** | **0,406** | **0,186** | **12 / 54.55** | **GLN 1, SER 2, THR 1, ASN 1, GLY 2** | **LYS 4, GLU 1,** | **10 / 45.45** | **inside** | **0,42** |
|  | **CATL1_CECB3** | **PVKNQGQCGSCWAFSKVFKKIE** | **22** | **2484,92** | **GOOD** | **34,31** | **-0,418** | **1,18** | **0,385** | **0,161** | **12 / 54.55** | **GLN 2, SER 2, ASN 1, GLY 2** | **LYS 4, GLU 1,** | **10 / 45.45** | **inside** | **0,48** |
|  | **CATH2_CECB3** | **YWIVKNSWGKVFKKIE** | **16** | **2025,42** | **GOOD** | **-8,54** | **-0,419** | **0,79** | **0,503** | **0,47** | **8 / 50.00** | **SER 1, ASN 1, GLY 1** | **LYS 4, GLU 1,** | **8 / 50.00** | **inside** | **0,68** |
|  | **CATL1.2_CECB3** | **YWLVKNSWGKVFKKIE** | **16** | **2025,42** | **GOOD** | **4,47** | **-0,463** | **0,79** | **0,497** | **0,474** | **8 / 50.00** | **SER 1, ASN 1, GLY 1** | **LYS 4, GLU 1,** | **8 / 50.00** | **inside** | **0,54** |
|  | **CATK_CECC1** | **HAVLVVGYGKLGKRIERIGQ** | **20** | **2193,62** | **GOOD** | **22,3** | **0,055** | **1,03** | **0,360** | **0,4** | **11 / 55.00** | **GLN 1, HIS 1, GLY 4** | **LYS 2, ARG 2, GLU 1,** | **9 / 45.00** | **outside** | **0,37** |
|  | **CATS_CECC1** | **HGVLVVGYGKLGKRIERIGQ** | **20** | **2179,6** | **GOOD** | **17,11** | **-0,055** | **1,07** | **0,344** | **0,414** | **12 / 60.00** | **GLN 1, HIS 1, GLY 5** | **LYS 2, ARG 2, GLU 1,** | **8 / 40.00** | **outside** | **0,29** |
|  | **CATH1_CECC1** | **PVKNQGACGSCWTFSKLGKRIERIGQ** | **26** | **2864,34** | **GOOD** | **70,17** | **-0,558** | **1,85** | **0,315** | **0,166** | **16 / 61.54** | **GLN 2, SER 2, THR 1, ASN 1, GLY 4** | **LYS 3, ARG 2, GLU 1,** | **10 / 38.46** | **inside** | **0,43** |
|  | **CATL1_CECC1** | **PVKNQGQCGSCWAFSKLGKRIERIGQ** | **26** | **2891,36** | **GOOD** | **48,89** | **-0,665** | **1,96** | **0,296** | **0,152** | **16 / 61.54** | **GLN 3, SER 2, ASN 1, GLY 4** | **LYS 3, ARG 2, GLU 1,** | **10 / 38.46** | **inside** | **0,49** |
|  | **CATH2_CECC1** | **YWIVKNSWGKLGKRIERIGQ** | **20** | **2431,87** | **GOOD** | **18,98** | **-0,74** | **1,89** | **0,364** | **0,454** | **12 / 60.00** | **GLN 1, SER 1, ASN 1, GLY 3** | **LYS 3, ARG 2, GLU 1,** | **8 / 40.00** | **inside** | **0,71** |
|  | **CATL1.2_CECC1** | **YWLVKNSWGKLGKRIERIGQ** | **20** | **2431,87** | **GOOD** | **29,39** | **-0,775** | **1,89** | **0,360** | **0,456** | **12 / 60.00** | **GLN 1, SER 1, ASN 1, GLY 3** | **LYS 3, ARG 2, GLU 1,** | **8 / 40.00** | **inside** | **0,58** |
|  | **CATK_CECC2** | **HAVLVVGYGKLGKKLEGAGK** | **20** | **2024,44** | **GOOD** | **-28,71** | **0,1** | **-0,06** | **0,293** | **0,337** | **11 / 55.00** | **HIS 1, GLY 5** | **LYS 4, GLU 1,** | **9 / 45.00** | **outside** | **0,28** |
|  | **CATS_CECC2** | **HGVLVVGYGKLGKKLEGAGK** | **20** | **2021,41** | **GOOD** | **-33,89** | **-0,01** | **-0,02** | **0,278** | **0,35** | **12 / 60.00** | **HIS 1, GLY 6** | **LYS 4, GLU 1,** | **8 / 40.00** | **outside** | **0,21** |
|  | **CATH1_CECC2** | **PVKNQGACGSCWTFSKLGKKLEGAGK** | **26** | **2695,15** | **GOOD** | **30,93** | **-0,523** | **1,00** | **0,263** | **0,12** | **16 / 61.54** | **GLN 1, SER 2, THR 1, ASN 1, GLY 5** | **LYS 5, GLU 1,** | **10 / 38.46** | **outside** | **0,39** |
|  | **CATL1_CECC2** | **PVKNQGQCGSCWAFSKLGKKLEGAGK** | **26** | **2722,18** | **GOOD** | **9,65** | **-0,631** | **1,11** | **0,245** | **0,104** | **16 / 61.54** | **GLN 2, SER 2, ASN 1, GLY 5** | **LYS 5, GLU 1,** | **10 / 38.46** | **inside** | **0,45** |
|  | **CATH2_CECC2** | **YWIVKNSWGKLGKKLEGAGK** | **20** | **2262,68** | **GOOD** | **-32,03** | **-0,695** | **0,78** | **0,298** | **0,391** | **12 / 60.00** | **SER 1, ASN 1, GLY 4** | **LYS 5, GLU 1,** | **8 / 40.00** | **inside** | **0,61** |
|  | **CATL1.2_CECC2** | **YWLVKNSWGKLGKKLEGAGK** | **20** | **2262,68** | **GOOD** | **-21,61** | **-0,73** | **0,78** | **0,293** | **0,392** | **12 / 60.00** | **SER 1, ASN 1, GLY 4** | **LYS 5, GLU 1,** | **8 / 40.00** | **inside** | **0,47** |
|  | **CATK_CECD1** | **HAVLVVGYGGQRIRDAIISAAPAV** | **24** | **2433,84** | **POOR** | **20,16** | **0,792** | **0,19** | **0,512** | **0,169** | **9 / 37.50** | **GLN 1, HIS 1, SER 1, GLY 3** | **ARG 2, ASP 1,** | **15 / 62.50** | **outside** | **0,13** |
|  | **CATS_CECD1** | **HGVLVVGYGGQRIRDAIISAAPAV** | **24** | **2419,81** | **POOR** | **15,84** | **0,7** | **0,22** | **0,499** | **0,162** | **10 / 41.67** | **GLN 1, HIS 1, SER 1, GLY 4** | **ARG 2, ASP 1,** | **14 / 58.33** | **outside** | **0,09** |
|  | **CATH1_CECD1** | **PVKNQGACGSCWTFSGQRIRDAIISAAPAV** | **30** | **3104,55** | **POOR** | **55,13** | **0,113** | **1,06** | **0,442** | **0,251** | **14 / 46.67** | **GLN 2, SER 3, THR 1, ASN 1, GLY 3** | **LYS 1, ARG 2, ASP 1,** | **16 / 53.33** | **outside** | **0,38** |
|  | **CATL1_CECD1** | **PVKNQGQCGSCWAFSGQRIRDAIISAAPAV** | **30** | **3131,58** | **POOR** | **36,69** | **0,02** | **1,16** | **0,426** | **0,263** | **14 / 46.67** | **GLN 3, SER 3, ASN 1, GLY 3** | **LYS 1, ARG 2, ASP 1,** | **16 / 53.33** | **inside** | **0,44** |
|  | **CATH2_CECD1** | **YWIVKNSWGGQRIRDAIISAAPAV** | **24** | **2672,08** | **POOR** | **17,4** | **0,129** | **0,90** | **0,516** | **0,13** | **10 / 41.67** | **GLN 1, SER 2, ASN 1, GLY 2** | **LYS 1, ARG 2, ASP 1,** | **14 / 58.33** | **inside** | **0,37** |
|  | **CATL1.2_CECD1** | **YWLVKNSWGGQRIRDAIISAAPAV** | **24** | **2672,08** | **POOR** | **26,08** | **0,1** | **0,90** | **0,512** | **0,127** | **10 / 41.67** | **GLN 1, SER 2, ASN 1, GLY 2** | **LYS 1, ARG 2, ASP 1,** | **14 / 58.33** | **outside** | **0,25** |
|  | **CATK_CECD2** | **HAVLVVGYGGQRVRDAVISAGPAV** | **24** | **2391,76** | **POOR** | **12,14** | **0,675** | **0,30** | **0,451** | **0,116** | **10 / 41.67** | **GLN 1, HIS 1, SER 1, GLY 4** | **ARG 2, ASP 1,** | **14 / 58.33** | **outside** | **0,12** |
|  | **CATS_CECD2** | **HGVLVVGYGGQRVRDAVISAGPAV** | **24** | **2377,73** | **POOR** | **7,82** | **0,583** | **0,33** | **0,438** | **0,109** | **11 / 45.83** | **GLN 1, HIS 1, SER 1, GLY 5** | **ARG 2, ASP 1,** | **13 / 54.17** | **outside** | **0,09** |
|  | **CATH1_CECD2** | **PVKNQGACGSCWTFSGQRVRDAVISAGPAV** | **30** | **3062,47** | **POOR** | **48,71** | **0,02** | **1,15** | **0,393** | **0,21** | **15 / 50.00** | **GLN 2, SER 3, THR 1, ASN 1, GLY 4** | **LYS 1, ARG 2, ASP 1,** | **15 / 50.00** | **outside** | **0,38** |
|  | **CATL1_CECD2** | **PVKNQGQCGSCWAFSGQRVRDAVISAGPAV** | **30** | **3089,5** | **POOR** | **30,27** | **-0,073** | **1,25** | **0,377** | **0,222** | **15 / 50.00** | **GLN 3, SER 3, ASN 1, GLY 4** | **LYS 1, ARG 2, ASP 1,** | **15 / 50.00** | **inside** | **0,43** |
|  | **CATH2_CECD2** | **YWIVKNSWGGQRVRDAVISAGPAV** | **24** | **2630** | **POOR** | **9,37** | **0,013** | **1,01** | **0,455** | **0,078** | **11 / 45.83** | **GLN 1, SER 2, ASN 1, GLY 3** | **LYS 1, ARG 2, ASP 1,** | **13 / 54.17** | **inside** | **0,36** |
|  | **CATL1.2_CECD2** | **YWLVKNSWGGQRVRDAVISAGPAV** | **24** | **2630** | **POOR** | **18,05** | **-0,017** | **1,01** | **0,450** | **0,075** | **11 / 45.83** | **GLN 1, SER 2, ASN 1, GLY 3** | **LYS 1, ARG 2, ASP 1,** | **13 / 54.17** | **outside** | **0,25** |
|  | **CATK_CECD3** | **HAVLVVGYGGQRVRDAIISAGPAV** | **24** | **2405,79** | **POOR** | **12,14** | **0,688** | **0,26** | **0,475** | **0,14** | **10 / 41.67** | **GLN 1, HIS 1, SER 1, GLY 4** | **ARG 2, ASP 1,** | **14 / 58.33** | **outside** | **0,12** |
|  | **CATS_CECD3** | **HGVLVVGYGGQRVRDAIISAGPAV** | **24** | **2391,76** | **POOR** | **7,82** | **0,596** | **0,30** | **0,462** | **0,133** | **11 / 45.83** | **GLN 1, HIS 1, SER 1, GLY 5** | **ARG 2, ASP 1,** | **13 / 54.17** | **outside** | **0,09** |
|  | **CATH1_CECD3** | **PVKNQGACGSCWTFSGQRVRDAIISAGPAV** | **30** | **3076,5** | **POOR** | **48,71** | **0,03** | **1,12** | **0,413** | **0,228** | **15 / 50.00** | **GLN 2, SER 3, THR 1, ASN 1, GLY 4** | **LYS 1, ARG 2, ASP 1,** | **15 / 50.00** | **outside** | **0,38** |
|  | **CATL1_CECD3** | **PVKNQGQCGSCWAFSGQRVRDAIISAGPAV** | **30** | **3103,52** | **POOR** | **30,27** | **-0,063** | **1,22** | **0,397** | **0,24** | **15 / 50.00** | **GLN 3, SER 3, ASN 1, GLY 4** | **LYS 1, ARG 2, ASP 1,** | **15 / 50.00** | **inside** | **0,43** |
|  | **CATH2_CECD3** | **YWIVKNSWGGQRVRDAIISAGPAV** | **24** | **2644,03** | **POOR** | **9,37** | **0,025** | **0,97** | **0,479** | **0,102** | **11 / 45.83** | **GLN 1, SER 2, ASN 1, GLY 3** | **LYS 1, ARG 2, ASP 1,** | **13 / 54.17** | **inside** | **0,37** |
|  | **CATL1.2_CECD3** | **YWLVKNSWGGQRVRDAIISAGPAV** | **24** | **2644,03** | **POOR** | **18,05** | **-0,004** | **0,97** | **0,475** | **0,099** | **11 / 45.83** | **GLN 1, SER 2, ASN 1, GLY 3** | **LYS 1, ARG 2, ASP 1,** | **13 / 54.17** | **outside** | **0,25** |
|  | **CATL1.2_CECB1_2** | **WLVKNSWGKLGKKI** | **14** | **1657,03** | **GOOD** | **-15,62** | **-0,443** | **0,49** | **0,451** | **0,435** | **8 / 57.14** | **SER 1, ASN 1, GLY 2** | **LYS 4,** | **6 / 42.86** | **inside** | **0,38** |
|  | **Parameters** | **-** | **-** | **-** | **-** | **< 40 Stable**  **>40 Unstable** | **>0 Hydrophobic**  **<0 Hydrophilic** | **>2.48 High <1 Low** | **>0.5 Low Hydrophobicity**  **<0.5 High Hydrophobicity** | **-** | **-** | **-** | **-** | **-** | **-** | **-** |

**Table S5 - Biological activity prediction**

|  |  |  | **IEDB** | **AllergenFP** | **AllerTOP** | **ToxinPred** | **ANTICANCER - ACPred** | | **ANTIVIRAL - Meta-iAVP** | | **HALF LIFE** | | | **HemoPI** |
| --- | --- | --- | --- | --- | --- | --- | --- | --- | --- | --- | --- | --- | --- | --- |
|  | **NAME** | **HYBRID PEPTIDE** | **Immunogenicity** | **Allergenicity** | | **Toxicity** | **Prediction** | **ACP** | **Prediction** | **AVP** | **IN VITRO** | **IN VIVO - YEAST** | **IN VIVO - E. coli** |  |
| **CECROPIN N TERMINAL** | **CEC1_CATK** | **KKIERVGQNTRDAHAVLVVGYG** | **0,50085** | **N-ALLERGEN** | **ALLERGEN** | **Non-Toxic** | **non-ACP** | **0,207** | **AVP** | **0,928** | **1,3 HOUR** | **3 MIN** | **3 MIN** | **0,47** |
|  | **CEC1_CATS** | **KKIERVGQNTRDAHGVLVVGYG** | **0,49575** | **N-ALLERGEN** | **ALLERGEN** | **Non-Toxic** | **non-ACP** | **0,363** | **Non-AVP** | **0,036** | **1,3 HOUR** | **3 MIN** | **3 MIN** | **0,48** |
|  | **CEC1_CATH1** | **KKIERVGQNTRDAPVKNQGACGSCWTFS** | **0,14432** | **N-ALLERGEN** | **ALLERGEN** | **Non-Toxic** | **non-ACP** | **0,134** | **AVP** | **0,752** | **1,3 HOUR** | **3 MIN** | **3 MIN** | **0,39** |
|  | **CEC1_CATL1** | **KKIERVGQNTRDAPVKNQGQCGSCWAFS** | **-0,00632** | **N-ALLERGEN** | **N-ALLERGEN** | **Non-Toxic** | **non-ACP** | **0,068** | **Non-AVP** | **0,11** | **1,3 HOUR** | **3 MIN** | **3 MIN** | **0,4** |
|  | **CEC1_CATH2** | **KKIERVGQNTRDAYWIVKNSWG** | **0,45196** | **ALLERGEN** | **ALLERGEN** | **Non-Toxic** | **ACP** | **0,688** | **AVP** | **0,994** | **1,3 HOUR** | **3 MIN** | **3 MIN** | **0,45** |
|  | **CEC1_CATL1.2** | **KKIERVGQNTRDAYWLVKNSWG** | **0,31156** | **ALLERGEN** | **ALLERGEN** | **Non-Toxic** | **non-ACP** | **0,39** | **AVP** | **0,81** | **1,3 HOUR** | **3 MIN** | **3 MIN** | **0,48** |
|  | **CEC2_CATK** | **KKIEKAVRRVRDGHAVLVVGYG** | **0,46275** | **N-ALLERGEN** | **N-ALLERGEN** | **Non-Toxic** | **ACP** | **0,875** | **Non-AVP** | **0,142** | **1,3 HOUR** | **3 MIN** | **3 MIN** | **0,48** |
|  | **CEC2_CATS** | **KKIEKAVRRVRDGHGVLVVGYG** | **0,45765** | **N-ALLERGEN** | **N-ALLERGEN** | **Non-Toxic** | **ACP** | **0,919** | **AVP** | **0,678** | **1,3 HOUR** | **3 MIN** | **3 MIN** | **0,48** |
|  | **CEC2_CATH1** | **KKIEKAVRRVRDGPVKNQGACGSCWTFS** | **0,10622** | **N-ALLERGEN** | **N-ALLERGEN** | **Non-Toxic** | **non-ACP** | **0,627** | **AVP** | **0,992** | **1,3 HOUR** | **3 MIN** | **3 MIN** | **0,43** |
|  | **CEC2_CATL1** | **KKIEKAVRRVRDGPVKNQGQCGSCWAFS** | **-0,04442** | **ALLERGEN** | **N-ALLERGEN** | **Non-Toxic** | **non-ACP** | **0,502** | **AVP** | **0,998** | **1,3 HOUR** | **3 MIN** | **3 MIN** | **0,43** |
|  | **CEC2_CATH2** | **KKIEKAVRRVRDGYWIVKNSWG** | **0,41386** | **N-ALLERGEN** | **N-ALLERGEN** | **Non-Toxic** | **ACP** | **0,943** | **Non-AVP** | **0,11** | **1,3 HOUR** | **3 MIN** | **3 MIN** | **0,49** |
|  | **CEC2_CATL.2** | **KKIEKAVRRVRDGYWLVKNSWG** | **0,27346** | **N-ALLERGEN** | **N-ALLERGEN** | **Non-Toxic** | **ACP** | **0,854** | **Non-AVP** | **0,43** | **1,3 HOUR** | **3 MIN** | **3 MIN** | **0,49** |
|  | **CEC3_CATK** | **KKIEKVGQNIRDGHAVLVVGYG** | **0,32715** | **N-ALLERGEN** | **ALLERGEN** | **Non-Toxic** | **ACP** | **0,857** | **AVP** | **0,64** | **1,3 HOUR** | **3 MIN** | **3 MIN** | **0,44** |
|  | **CEC3_CATS** | **KKIEKVGQNIRDGHGVLVVGYG** | **0,32205** | **ALLERGEN** | **ALLERGEN** | **Non-Toxic** | **ACP** | **0,915** | **Non-AVP** | **0,4** | **1,3 HOUR** | **3 MIN** | **3 MIN** | **0,47** |
|  | **CEC3_CATH1** | **KKIEKVGQNIRDGPVKNQGACGSCWTFS** | **-0,02938** | **N-ALLERGEN** | **N-ALLERGEN** | **Non-Toxic** | **ACP** | **0,697** | **AVP** | **0,846** | **1,3 HOUR** | **3 MIN** | **3 MIN** | **0,35** |
|  | **CEC3_CATL1** | **KKIEKVGQNIRDGPVKNQGQCGSCWAFS** | **-0,18002** | **ALLERGEN** | **N-ALLERGEN** | **Non-Toxic** | **non-ACP** | **0,535** | **AVP** | **0,972** | **1,3 HOUR** | **3 MIN** | **3 MIN** | **0,39** |
|  | **CEC3_CATH2** | **KKIEKVGQNIRDGYWIVKNSWG** | **0,27826** | **N-ALLERGEN** | **N-ALLERGEN** | **Non-Toxic** | **ACP** | **0,966** | **Non-AVP** | **0,316** | **1,3 HOUR** | **3 MIN** | **3 MIN** | **0,45** |
|  | **CEC3_CATL1.2** | **KKIEKVGQNIRDGYWLVKNSWG** | **0,13786** | **ALLERGEN** | **N-ALLERGEN** | **Non-Toxic** | **ACP** | **0,91** | **AVP** | **0,99** | **1,3 HOUR** | **3 MIN** | **3 MIN** | **0,44** |
|  | **CEC4_CATK** | **KKIGRVGQHTRDAHAVLVVGYG** | **0,472** | **ALLERGEN** | **ALLERGEN** | **Non-Toxic** | **ACP** | **0,821** | **AVP** | **1** | **1,3 HOUR** | **3 MIN** | **3 MIN** | **0,48** |
|  | **CEC4_CATS** | **KKIGRVGQHTRDAHGVLVVGYG** | **0,4669** | **ALLERGEN** | **ALLERGEN** | **Non-Toxic** | **ACP** | **0,893** | **Non-AVP** | **0,106** | **1,3 HOUR** | **3 MIN** | **3 MIN** | **0,48** |
|  | **CEC4_CATH1** | **KKIGRVGQHTRDAPVKNQGACGSCWTFS** | **0,11547** | **N-ALLERGEN** | **ALLERGEN** | **Non-Toxic** | **non-ACP** | **0,495** | **AVP** | **0,81** | **1,3 HOUR** | **3 MIN** | **3 MIN** | **0,42** |
|  | **CEC4_CATL1** | **KKIGRVGQHTRDAPVKNQGQCGSCWAFS** | **-0,03517** | **N-ALLERGEN** | **N-ALLERGEN** | **Non-Toxic** | **non-ACP** | **0,326** | **AVP** | **0,98** | **1,3 HOUR** | **3 MIN** | **3 MIN** | **0,42** |
|  | **CEC4_CATH2** | **KKIGRVGQHTRDAYWIVKNSWG** | **0,42311** | **N-ALLERGEN** | **ALLERGEN** | **Non-Toxic** | **ACP** | **0,94** | **AVP** | **0,972** | **1,3 HOUR** | **3 MIN** | **3 MIN** | **0,43** |
|  | **CEC4_CATL1.2** | **KKIGRVGQHTRDAYWLVKNSWG** | **0,28271** | **N-ALLERGEN** | **ALLERGEN** | **Non-Toxic** | **ACP** | **0,829** | **AVP** | **1** | **1,3 HOUR** | **3 MIN** | **3 MIN** | **0,45** |
|  | **CECA1_CATK** | **KLGKKLEGAGKRHAVLVVGYG** | **-0,1803** | **N-ALLERGEN** | **N-ALLERGEN** | **Non-Toxic** | **ACP** | **0,854** | **Non-AVP** | **0,002** | **1,3 HOUR** | **3 MIN** | **3 MIN** | **0,43** |
|  | **CECA1_CATS** | **KLGKKLEGAGKRHGVLVVGYG** | **-0,1854** | **N-ALLERGEN** | **N-ALLERGEN** | **Non-Toxic** | **ACP** | **0,893** | **AVP** | **0,64** | **1,3 HOUR** | **3 MIN** | **3 MIN** | **0,49** |
|  | **CECA1_CATH1** | **KLGKKLEGAGKRPVKNQGACGSCWTFS** | **-0,53683** | **ALLERGEN** | **N-ALLERGEN** | **Non-Toxic** | **ACP** | **0,937** | **AVP** | **0,854** | **1,3 HOUR** | **3 MIN** | **3 MIN** | **0,51** |
|  | **CECA1_CATL1** | **KLGKKLEGAGKRPVKNQGQCGSCWAFS** | **-0,68747** | **N-ALLERGEN** | **N-ALLERGEN** | **Non-Toxic** | **ACP** | **0,891** | **Non-AVP** | **0,27** | **1,3 HOUR** | **3 MIN** | **3 MIN** | **0,47** |
|  | **CECA1_CATH2** | **KLGKKLEGAGKRYWIVKNSWG** | **-0,22919** | **N-ALLERGEN** | **N-ALLERGEN** | **Non-Toxic** | **ACP** | **0,985** | **AVP** | **0,964** | **1,3 HOUR** | **3 MIN** | **3 MIN** | **0,47** |
|  | **CECA1_CATL1.2** | **KLGKKLEGAGKRYWLVKNSWG** | **-0,36959** | **ALLERGEN** | **N-ALLERGEN** | **Non-Toxic** | **ACP** | **0,956** | **AVP** | **0,994** | **1,3 HOUR** | **3 MIN** | **3 MIN** | **0,49** |
|  | **CECA2_CATK** | **KIGKKIERVGQHHAVLVVGYG** | **0,0579** | **N-ALLERGEN** | **ALLERGEN** | **Non-Toxic** | **ACP** | **0,996** | **AVP** | **0,668** | **1,3 HOUR** | **3 MIN** | **3 MIN** | **0,47** |
|  | **CECA2_CATS** | **KIGKKIERVGQHHGVLVVGYG** | **0,0528** | **N-ALLERGEN** | **ALLERGEN** | **Non-Toxic** | **ACP** | **0,997** | **AVP** | **0,998** | **1,3 HOUR** | **3 MIN** | **3 MIN** | **0,48** |
|  | **CECA2_CATH1** | **KIGKKIERVGQHPVKNQGACGSCWTFS** | **-0,29863** | **ALLERGEN** | **N-ALLERGEN** | **Non-Toxic** | **ACP** | **0,984** | **AVP** | **0,726** | **1,3 HOUR** | **3 MIN** | **3 MIN** | **0,37** |
|  | **CECA2_CATL1** | **KIGKKIERVGQHPVKNQGQCGSCWAFS** | **-0,44927** | **N-ALLERGEN** | **ALLERGEN** | **Non-Toxic** | **ACP** | **0,968** | **AVP** | **0,954** | **1,3 HOUR** | **3 MIN** | **3 MIN** | **0,4** |
|  | **CECA2_CATH2** | **KIGKKIERVGQHYWIVKNSWG** | **0,00901** | **N-ALLERGEN** | **N-ALLERGEN** | **Non-Toxic** | **ACP** | **0,997** | **AVP** | **0,892** | **1,3 HOUR** | **3 MIN** | **3 MIN** | **0,45** |
|  | **CECA2_CATL1.2** | **KIGKKIERVGQHYWLVKNSWG** | **-0,13139** | **N-ALLERGEN** | **ALLERGEN** | **Non-Toxic** | **ACP** | **0,993** | **AVP** | **0,77** | **1,3 HOUR** | **3 MIN** | **3 MIN** | **0,45** |
|  | **CECA3_CATK** | **KVFKKIEKVGRNHAVLVVGYG** | **-0,0501** | **N-ALLERGEN** | **ALLERGEN** | **Non-Toxic** | **ACP** | **0,996** | **AVP** | **0,726** | **1,3 HOUR** | **3 MIN** | **3 MIN** | **0,47** |
|  | **CECA3_CATS** | **KVFKKIEKVGRNHGVLVVGYG** | **-0,0552** | **N-ALLERGEN** | **ALLERGEN** | **Non-Toxic** | **ACP** | **0,997** | **AVP** | **0,774** | **1,3 HOUR** | **3 MIN** | **3 MIN** | **0,48** |
|  | **CECA3_CATH1** | **KVFKKIEKVGRNPVKNQGACGSCWTFS** | **-0,40663** | **N-ALLERGEN** | **N-ALLERGEN** | **Non-Toxic** | **ACP** | **0,995** | **AVP** | **1** | **1,3 HOUR** | **3 MIN** | **3 MIN** | **0,47** |
|  | **CECA3_CATL1** | **KVFKKIEKVGRNPVKNQGQCGSCWAFS** | **-0,55727** | **ALLERGEN** | **ALLERGEN** | **Non-Toxic** | **ACP** | **0,99** | **AVP** | **0,64** | **1,3 HOUR** | **3 MIN** | **3 MIN** | **0,46** |
|  | **CECA3_CATH2** | **KVFKKIEKVGRNYWIVKNSWG** | **-0,09899** | **N-ALLERGEN** | **N-ALLERGEN** | **Non-Toxic** | **ACP** | **0,998** | **AVP** | **1** | **1,3 HOUR** | **3 MIN** | **3 MIN** | **0,5** |
|  | **CECA3_CATL1.2** | **KVFKKIEKVGRNYWLVKNSWG** | **-0,23939** | **N-ALLERGEN** | **N-ALLERGEN** | **Non-Toxic** | **ACP** | **0,996** | **AVP** | **0,98** | **1,3 HOUR** | **3 MIN** | **3 MIN** | **0,5** |
|  | **CECA4_CATK** | **KFGKKLEGVGKRHAVLVVGYG** | **-0,1782** | **N-ALLERGEN** | **ALLERGEN** | **Non-Toxic** | **ACP** | **0,964** | **AVP** | **0,81** | **1,3 HOUR** | **3 MIN** | **3 MIN** | **0,44** |
|  | **CECA4_CATS** | **KFGKKLEGVGKRHGVLVVGYG** | **-0,1833** | **ALLERGEN** | **N-ALLERGEN** | **Non-Toxic** | **ACP** | **0,971** | **Non-AVP** | **0,038** | **1,3 HOUR** | **3 MIN** | **3 MIN** | **0,49** |
|  | **CECA4_CATH1** | **KFGKKLEGVGKRPVKNQGACGSCWTFS** | **-0,53473** | **N-ALLERGEN** | **ALLERGEN** | **Non-Toxic** | **ACP** | **0,981** | **AVP** | **0,996** | **1,3 HOUR** | **3 MIN** | **3 MIN** | **0,48** |
|  | **CECA4_CATL1** | **KFGKKLEGVGKRPVKNQGQCGSCWAFS** | **-0,68537** | **N-ALLERGEN** | **N-ALLERGEN** | **Non-Toxic** | **ACP** | **0,964** | **AVP** | **1** | **1,3 HOUR** | **3 MIN** | **3 MIN** | **0,47** |
|  | **CECA4_CATH2** | **KFGKKLEGVGKRYWIVKNSWG** | **-0,22709** | **ALLERGEN** | **N-ALLERGEN** | **Non-Toxic** | **ACP** | **0,994** | **AVP** | **0,906** | **1,3 HOUR** | **3 MIN** | **3 MIN** | **0,47** |
|  | **CECA4_CATL1.2** | **KFGKKLEGVGKRYWLVKNSWG** | **-0,36749** | **N-ALLERGEN** | **N-ALLERGEN** | **Non-Toxic** | **ACP** | **0,983** | **AVP** | **0,972** | **1,3 HOUR** | **3 MIN** | **3 MIN** | **0,46** |
|  | **CECB1_CATK** | **KLGKKIEHAVLVVGYG** | **0,0156** | **ALLERGEN** | **ALLERGEN** | **Non-Toxic** | **ACP** | **0,99** | **AVP** | **0,588** | **1,3 HOUR** | **3 MIN** | **3 MIN** | **0,43** |
|  | **CECB1_CATS** | **KLGKKIEHGVLVVGYG** | **0,0105** | **N-ALLERGEN** | **ALLERGEN** | **Non-Toxic** | **ACP** | **0,993** | **Non-AVP** | **0,068** | **1,3 HOUR** | **3 MIN** | **3 MIN** | **0,49** |
|  | **CECB1_CATH1** | **KLGKKIEPVKNQGACGSCWTFS** | **-0,34093** | **ALLERGEN** | **ALLERGEN** | **Non-Toxic** | **ACP** | **0,943** | **Non-AVP** | **0,218** | **1,3 HOUR** | **3 MIN** | **3 MIN** | **0,43** |
|  | **CECB1_CATL1** | **KLGKKIEPVKNQGQCGSCWAFS** | **-0,49157** | **ALLERGEN** | **N-ALLERGEN** | **Non-Toxic** | **ACP** | **0,88** | **AVP** | **0,676** | **1,3 HOUR** | **3 MIN** | **3 MIN** | **0,41** |
|  | **CECB1_CATH2** | **KLGKKIEYWIVKNSWG** | **-0,03329** | **N-ALLERGEN** | **N-ALLERGEN** | **Non-Toxic** | **ACP** | **0,998** | **AVP** | **1** | **1,3 HOUR** | **3 MIN** | **3 MIN** | **0,49** |
|  | **CECB1_CATL1.2** | **KLGKKIEYWLVKNSWG** | **-0,17369** | **N-ALLERGEN** | **N-ALLERGEN** | **Non-Toxic** | **ACP** | **0,991** | **AVP** | **0,892** | **1,3 HOUR** | **3 MIN** | **3 MIN** | **0,51** |
|  | **CECB2_CATK** | **KIFKKIEHAVLVVGYG** | **0,0426** | **N-ALLERGEN** | **N-ALLERGEN** | **Non-Toxic** | **ACP** | **1** | **AVP** | **0,98** | **1,3 HOUR** | **3 MIN** | **3 MIN** | **0,46** |
|  | **CECB2_CATS** | **KIFKKIEHGVLVVGYG** | **0,0375** | **N-ALLERGEN** | **N-ALLERGEN** | **Non-Toxic** | **ACP** | **1** | **AVP** | **0,64** | **1,3 HOUR** | **3 MIN** | **3 MIN** | **0,47** |
|  | **CECB2_CATH1** | **KIFKKIEPVKNQGACGSCWTFS** | **-0,31393** | **N-ALLERGEN** | **ALLERGEN** | **Non-Toxic** | **ACP** | **0,995** | **AVP** | **0,774** | **1,3 HOUR** | **3 MIN** | **3 MIN** | **0,43** |
|  | **CECB2_CATL1** | **KIFKKIEPVKNQGQCGSCWAFS** | **-0,46457** | **ALLERGEN** | **ALLERGEN** | **Non-Toxic** | **ACP** | **0,988** | **AVP** | **0,544** | **1,3 HOUR** | **3 MIN** | **3 MIN** | **0,43** |
|  | **CECB2_CATH2** | **KIFKKIEYWIVKNSWG** | **-0,00629** | **N-ALLERGEN** | **N-ALLERGEN** | **Non-Toxic** | **ACP** | **0,999** | **AVP** | **0,752** | **1,3 HOUR** | **3 MIN** | **3 MIN** | **0,49** |
|  | **CECB2_CATL1.2** | **KIFKKIEYWLVKNSWG** | **-0,14669** | **ALLERGEN** | **N-ALLERGEN** | **Non-Toxic** | **ACP** | **0,999** | **AVP** | **0,788** | **1,3 HOUR** | **3 MIN** | **3 MIN** | **0,5** |
|  | **CECB3_CATK** | **KVFKKIEHAVLVVGYG** | **0,0426** | **N-ALLERGEN** | **N-ALLERGEN** | **Non-Toxic** | **ACP** | **0,998** | **AVP** | **0,936** | **1,3 HOUR** | **3 MIN** | **3 MIN** | **0,48** |
|  | **CECB3_CATS** | **KVFKKIEHGVLVVGYG** | **0,0375** | **N-ALLERGEN** | **N-ALLERGEN** | **Non-Toxic** | **ACP** | **0,999** | **AVP** | **0,954** | **1,3 HOUR** | **3 MIN** | **3 MIN** | **0,49** |
|  | **CECB3_CATH1** | **KVFKKIEPVKNQGACGSCWTFS** | **-0,31393** | **N-ALLERGEN** | **ALLERGEN** | **Non-Toxic** | **ACP** | **0,987** | **AVP** | **0,966** | **1,3 HOUR** | **3 MIN** | **3 MIN** | **0,47** |
|  | **CECB3_CATL1** | **KVFKKIEPVKNQGQCGSCWAFS** | **-0,46457** | **N-ALLERGEN** | **ALLERGEN** | **Non-Toxic** | **ACP** | **0,972** | **AVP** | **0,966** | **1,3 HOUR** | **3 MIN** | **3 MIN** | **0,47** |
|  | **CECB3_CATH2** | **KVFKKIEYWIVKNSWG** | **-0,00629** | **N-ALLERGEN** | **N-ALLERGEN** | **Non-Toxic** | **ACP** | **0,999** | **AVP** | **0,998** | **1,3 HOUR** | **3 MIN** | **3 MIN** | **0,5** |
|  | **CECB3_CATL1.2** | **KVFKKIEYWLVKNSWG** | **-0,14669** | **ALLERGEN** | **N-ALLERGEN** | **Non-Toxic** | **ACP** | **0,997** | **AVP** | **0,972** | **1,3 HOUR** | **3 MIN** | **3 MIN** | **0,51** |
|  | **CECC1_CATK** | **KLGKRIERIGQHAVLVVGYG** | **0,3762** | **ALLERGEN** | **ALLERGEN** | **Non-Toxic** | **ACP** | **0,952** | **AVP** | **0,906** | **1,3 HOUR** | **3 MIN** | **3 MIN** | **0,51** |
|  | **CECC1_CATS** | **KLGKRIERIGQHGVLVVGYG** | **0,3711** | **ALLERGEN** | **ALLERGEN** | **Non-Toxic** | **ACP** | **0,967** | **AVP** | **0,586** | **1,3 HOUR** | **3 MIN** | **3 MIN** | **0,5** |
|  | **CECC1_CATH1** | **KLGKRIERIGQPVKNQGACGSCWTFS** | **0,01967** | **N-ALLERGEN** | **N-ALLERGEN** | **Non-Toxic** | **ACP** | **0,804** | **AVP** | **1** | **1,3 HOUR** | **3 MIN** | **3 MIN** | **0,3** |
|  | **CECC1_CATL1** | **KLGKRIERIGQPVKNQGQCGSCWAFS** | **-0,13097** | **N-ALLERGEN** | **N-ALLERGEN** | **Non-Toxic** | **ACP** | **0,692** | **AVP** | **0,828** | **1,3 HOUR** | **3 MIN** | **3 MIN** | **0,35** |
|  | **CECC1_CATH2** | **KLGKRIERIGQYWIVKNSWG** | **0,32731** | **N-ALLERGEN** | **N-ALLERGEN** | **Non-Toxic** | **ACP** | **0,973** | **AVP** | **0,97** | **1,3 HOUR** | **3 MIN** | **3 MIN** | **0,42** |
|  | **CECC1_CATL1.2** | **KLGKRIERIGQYWLVKNSWG** | **0,18691** | **N-ALLERGEN** | **N-ALLERGEN** | **Non-Toxic** | **ACP** | **0,918** | **AVP** | **0,928** | **1,3 HOUR** | **3 MIN** | **3 MIN** | **0,44** |
|  | **CECC2_CATK** | **KLGKKLEGAGKHAVLVVGYG** | **-0,2307** | **N-ALLERGEN** | **ALLERGEN** | **Non-Toxic** | **ACP** | **0,967** | **AVP** | **0,876** | **1,3 HOUR** | **3 MIN** | **3 MIN** | **0,45** |
|  | **CECC2_CATS** | **KLGKKLEGAGKHGVLVVGYG** | **-0,2358** | **N-ALLERGEN** | **N-ALLERGEN** | **Non-Toxic** | **ACP** | **0,974** | **Non-AVP** | **0** | **1,3 HOUR** | **3 MIN** | **3 MIN** | **0,49** |
|  | **CECC2_CATH1** | **KLGKKLEGAGKPVKNQGACGSCWTFS** | **-0,58723** | **N-ALLERGEN** | **N-ALLERGEN** | **Non-Toxic** | **ACP** | **0,957** | **AVP** | **0,928** | **1,3 HOUR** | **3 MIN** | **3 MIN** | **0,51** |
|  | **CECC2_CATL1** | **KLGKKLEGAGKPVKNQGQCGSCWAFS** | **-0,73787** | **N-ALLERGEN** | **N-ALLERGEN** | **Non-Toxic** | **ACP** | **0,921** | **AVP** | **0,678** | **1,3 HOUR** | **3 MIN** | **3 MIN** | **0,47** |
|  | **CECC2_CATH2** | **KLGKKLEGAGKYWIVKNSWG** | **-0,27959** | **N-ALLERGEN** | **ALLERGEN** | **Non-Toxic** | **ACP** | **0,996** | **AVP** | **0,846** | **1,3 HOUR** | **3 MIN** | **3 MIN** | **0,49** |
|  | **CECC2_CATL1.2** | **KLGKKLEGAGKYWLVKNSWG** | **-0,41999** | **N-ALLERGEN** | **N-ALLERGEN** | **Non-Toxic** | **ACP** | **0,985** | **Non-AVP** | **0,106** | **1,3 HOUR** | **3 MIN** | **3 MIN** | **0,51** |
|  | **CECD1_CATK** | **GQRIRDAIISAAPAVHAVLVVGYG** | **0,70712** | **N-ALLERGEN** | **N-ALLERGEN** | **Non-Toxic** | **non-ACP** | **0,095** | **Non-AVP** | **0,354** | **30 HOUR** | **>20 HOUR** | **>10 HOUR** | **0,48** |
|  | **CECD1_CATS** | **GQRIRDAIISAAPAVHGVLVVGYG** | **0,70202** | **N-ALLERGEN** | **ALLERGEN** | **Non-Toxic** | **non-ACP** | **0,124** | **AVP** | **0,698** | **30 HOUR** | **>20 HOUR** | **>10 HOUR** | **0,49** |
|  | **CECD1_CATH1** | **GQRIRDAIISAAPAVPVKNQGACGSCWTFS** | **0,35059** | **N-ALLERGEN** | **N-ALLERGEN** | **Non-Toxic** | **non-ACP** | **0,026** | **AVP** | **0,994** | **30 HOUR** | **>20 HOUR** | **>10 HOUR** | **0,47** |
|  | **CECD1_CATL1** | **GQRIRDAIISAAPAVPVKNQGQCGSCWAFS** | **0,19995** | **ALLERGEN** | **N-ALLERGEN** | **Non-Toxic** | **non-ACP** | **0,019** | **Non-AVP** | **0,008** | **30 HOUR** | **>20 HOUR** | **>10 HOUR** | **0,47** |
|  | **CECD1_CATH2** | **GQRIRDAIISAAPAVYWIVKNSWG** | **0,65823** | **ALLERGEN** | **N-ALLERGEN** | **Non-Toxic** | **non-ACP** | **0,257** | **Non-AVP** | **0,022** | **30 HOUR** | **>20 HOUR** | **>10 HOUR** | **0,48** |
|  | **CECD1_CATL1.2** | **GQRIRDAIISAAPAVYWLVKNSWG** | **0,51783** | **ALLERGEN** | **ALLERGEN** | **Non-Toxic** | **non-ACP** | **0,072** | **AVP** | **0,678** | **30 HOUR** | **>20 HOUR** | **>10 HOUR** | **0,49** |
|  | **CECD2_CATK** | **GQRVRDAVISAGPAVHAVLVVGYG** | **0,52024** | **N-ALLERGEN** | **ALLERGEN** | **Non-Toxic** | **non-ACP** | **0,064** | **Non-AVP** | **0,294** | **30 HOUR** | **>20 HOUR** | **>10 HOUR** | **0,49** |
|  | **CECD2_CATS** | **GQRVRDAVISAGPAVHGVLVVGYG** | **0,51514** | **N-ALLERGEN** | **ALLERGEN** | **Non-Toxic** | **non-ACP** | **0,093** | **Non-AVP** | **0,004** | **30 HOUR** | **>20 HOUR** | **>10 HOUR** | **0,49** |
|  | **CECD2_CATH1** | **GQRVRDAVISAGPAVPVKNQGACGSCWTFS** | **0,16371** | **ALLERGEN** | **ALLERGEN** | **Non-Toxic** | **non-ACP** | **0,009** | **AVP** | **0,994** | **30 HOUR** | **>20 HOUR** | **>10 HOUR** | **0,49** |
|  | **CECD2_CATL1** | **GQRVRDAVISAGPAVPVKNQGQCGSCWAFS** | **0,01307** | **N-ALLERGEN** | **ALLERGEN** | **Non-Toxic** | **non-ACP** | **0,007** | **Non-AVP** | **0,47** | **30 HOUR** | **>20 HOUR** | **>10 HOUR** | **0,48** |
|  | **CECD2_CATH2** | **GQRVRDAVISAGPAVYWIVKNSWG** | **0,47135** | **ALLERGEN** | **ALLERGEN** | **Non-Toxic** | **non-ACP** | **0,101** | **Non-AVP** | **0,398** | **30 HOUR** | **>20 HOUR** | **>10 HOUR** | **0,5** |
|  | **CECD2_CATL1.2** | **GQRVRDAVISAGPAVYWLVKNSWG** | **0,33095** | **ALLERGEN** | **ALLERGEN** | **Non-Toxic** | **non-ACP** | **0,029** | **AVP** | **0,81** | **30 HOUR** | **>20 HOUR** | **>10 HOUR** | **0,51** |
|  | **CECD3_CATK** | **GQRVRDAIISAGPAVHAVLVVGYG** | **0,60964** | **N-ALLERGEN** | **ALLERGEN** | **Non-Toxic** | **non-ACP** | **0,076** | **Non-AVP** | **0,43** | **30 HOUR** | **>20 HOUR** | **>10 HOUR** | **0,49** |
|  | **CECD3_CATS** | **GQRVRDAIISAGPAVHGVLVVGYG** | **0,60454** | **N-ALLERGEN** | **ALLERGEN** | **Non-Toxic** | **non-ACP** | **0,114** | **Non-AVP** | **0,43** | **30 HOUR** | **>20 HOUR** | **>10 HOUR** | **0,5** |
|  | **CECD3_CATH1** | **GQRVRDAIISAGPAVPVKNQGACGSCWTFS** | **0,25311** | **ALLERGEN** | **ALLERGEN** | **Non-Toxic** | **non-ACP** | **0,015** | **AVP** | **0,676** | **30 HOUR** | **>20 HOUR** | **>10 HOUR** | **0,48** |
|  | **CECD3_CATL1** | **GQRVRDAIISAGPAVPVKNQGQCGSCWAFS** | **0,10247** | **N-ALLERGEN** | **ALLERGEN** | **Non-Toxic** | **non-ACP** | **0,011** | **Non-AVP** | **0,398** | **30 HOUR** | **>20 HOUR** | **>10 HOUR** | **0,47** |
|  | **CECD3_CATH2** | **GQRVRDAIISAGPAVYWIVKNSWG** | **0,56075** | **ALLERGEN** | **ALLERGEN** | **Non-Toxic** | **non-ACP** | **0,171** | **Non-AVP** | **0,122** | **30 HOUR** | **>20 HOUR** | **>10 HOUR** | **0,49** |
|  | **CECD3_CATL1.2** | **GQRVRDAIISAGPAVYWLVKNSWG** | **0,42035** | **N-ALLERGEN** | **ALLERGEN** | **Non-Toxic** | **non-ACP** | **0,047** | **AVP** | **1** | **30 HOUR** | **>20 HOUR** | **>10 HOUR** | **0,51** |
|  |  |  |  |  |  |  |  |  |  |  |  |  |  |  |
| **CATHEPSINS N TERMINAL** | **CATK_CEC1** | **HAVLVVGYGKKIERVGQNTRDA** | **0,04982** | **N-ALLERGEN** | **N-ALLERGEN** | **Non-Toxic** | **non-ACP** | **0,271** | **AVP** | **0,928** | **3,5 HOUR** | **10 MIM** | **> 10 HOUR** | **0,47** |
|  | **CATS_CEC1** | **HGVLVVGYGKKIERVGQNTRDA** | **0,04982** | **ALLERGEN** | **N-ALLERGEN** | **Non-Toxic** | **non-ACP** | **0,43** | **Non-AVP** | **0,036** | **3,5 HOUR** | **10 MIM** | **> 10 HOUR** | **0,48** |
|  | **CATH1_CEC1** | **PVKNQGACGSCWTFSKKIERVGQNTRDA** | **-0,24013** | **ALLERGEN** | **ALLERGEN** | **Non-Toxic** | **non-ACP** | **0,218** | **AVP** | **0,752** | **>20 HOUR** | **>20 HOUR** | **?** | **0,39** |
|  | **CATL1_CEC1** | **PVKNQGQCGSCWAFSKKIERVGQNTRDA** | **-0,39073** | **N-ALLERGEN** | **N-ALLERGEN** | **Non-Toxic** | **non-ACP** | **0,106** | **Non-AVP** | **0,11** | **>20 HOUR** | **>20 HOUR** | **?** | **0,4** |
|  | **CATH2_CEC1** | **YWIVKNSWGKKIERVGQNTRDA** | **-0,13918** | **N-ALLERGEN** | **ALLERGEN** | **Non-Toxic** | **ACP** | **0,797** | **AVP** | **0,994** | **2,8 HOUR** | **10 MIN** | **2 MIN** | **0,45** |
|  | **CATL1.2_CEC1** | **YWLVKNSWGKKIERVGQNTRDA** | **-0,18598** | **N-ALLERGEN** | **ALLERGEN** | **Non-Toxic** | **non-ACP** | **0,533** | **AVP** | **0,81** | **2,8 HOUR** | **10 MIN** | **2 MIN** | **0,48** |
|  | **CATK_CEC2** | **HAVLVVGYGKKIEKAVRRVRDG** | **0,01674** | **ALLERGEN** | **N-ALLERGEN** | **Non-Toxic** | **ACP** | **0,913** | **Non-AVP** | **0,142** | **3,5 HOUR** | **10 MIN** | **>10 HOUR** | **0,48** |
|  | **CATS_CEC2** | **HGVLVVGYGKKIEKAVRRVRDG** | **0,01674** | **N-ALLERGEN** | **N-ALLERGEN** | **Non-Toxic** | **ACP** | **0,941** | **AVP** | **0,678** | **3,5 HOUR** | **10 MIN** | **>10 HOUR** | **0,48** |
|  | **CATH1_CEC2** | **PVKNQGACGSCWTFSKKIEKAVRRVRDG** | **-0,27321** | **N-ALLERGEN** | **N-ALLERGEN** | **Non-Toxic** | **ACP** | **0,73** | **AVP** | **0,992** | **>20 HOUR** | **>20 HOUR** | **?** | **0,43** |
|  | **CATL1_CEC2** | **PVKNQGQCGSCWAFSKKIEKAVRRVRDG** | **-0,42381** | **N-ALLERGEN** | **N-ALLERGEN** | **Non-Toxic** | **non-ACP** | **0,587** | **AVP** | **0,998** | **>20 HOUR** | **>20 HOUR** | **?** | **0,49** |
|  | **CATH2_CEC2** | **YWIVKNSWGKKIEKAVRRVRDG** | **-0,17226** | **N-ALLERGEN** | **N-ALLERGEN** | **Non-Toxic** | **ACP** | **0,97** | **Non-AVP** | **0,11** | **2,8 HOUR** | **10 MIN** | **2 MIN** | **0,49** |
|  | **CATL1.2_CEC2** | **YWLVKNSWGKKIEKAVRRVRDG** | **-0,21906** | **N-ALLERGEN** | **N-ALLERGEN** | **Non-Toxic** | **ACP** | **0,926** | **Non-AVP** | **0,43** | **2,8 HOUR** | **10 MIN** | **2 MIN** | **0,49** |
|  | **CATK_CEC3** | **HAVLVVGYGKKIEKVGQNIRDG** | **-0,12184** | **N-ALLERGEN** | **ALLERGEN** | **Non-Toxic** | **ACP** | **0,904** | **AVP** | **0,64** | **3,5 HOUR** | **10 MIN** | **>10 HOUR** | **0,44** |
|  | **CATS_CEC3** | **HGVLVVGYGKKIEKVGQNIRDG** | **-0,12184** | **ALLERGEN** | **ALLERGEN** | **Non-Toxic** | **ACP** | **0,94** | **Non-AVP** | **0,4** | **3,5 HOUR** | **10 MIN** | **>10 HOUR** | **0,47** |
|  | **CATH1_CEC3** | **PVKNQGACGSCWTFSKKIEKVGQNIRDG** | **-0,41179** | **N-ALLERGEN** | **ALLERGEN** | **Non-Toxic** | **ACP** | **0,795** | **AVP** | **0,846** | **>20 HOUR** | **>20 HOUR** | **?** | **0,35** |
|  | **CATL1_CEC3** | **PVKNQGQCGSCWAFSKKIEKVGQNIRDG** | **-0,56239** | **N-ALLERGEN** | **N-ALLERGEN** | **Non-Toxic** | **non-ACP** | **0,63** | **AVP** | **0,972** | **>20 HOUR** | **>20 HOUR** | **?** | **0,39** |
|  | **CATH2_CEC3** | **YWIVKNSWGKKIEKVGQNIRDG** | **-0,31084** | **N-ALLERGEN** | **ALLERGEN** | **Non-Toxic** | **ACP** | **0,98** | **Non-AVP** | **0,316** | **2,8 HOUR** | **10 MIN** | **2 MIN** | **0,45** |
|  | **CATL1.2_CEC3** | **YWLVKNSWGKKIEKVGQNIRDG** | **-0,35764** | **N-ALLERGEN** | **ALLERGEN** | **Non-Toxic** | **ACP** | **0,949** | **AVP** | **0,99** | **2,8 HOUR** | **10 MIN** | **2 MIN** | **0,44** |
|  | **CATK_CEC4** | **HAVLVVGYGKKIGRVGQHTRDA** | **0,02312** | **N-ALLERGEN** | **ALLERGEN** | **Non-Toxic** | **ACP** | **0,872** | **AVP** | **1** | **3,5 HOUR** | **10 MIN** | **>10 HOUR** | **0,48** |
|  | **CATS_CEC4** | **HGVLVVGYGKKIGRVGQHTRDA** | **0,02312** | **N-ALLERGEN** | **ALLERGEN** | **Non-Toxic** | **ACP** | **0,918** | **Non-AVP** | **0,106** | **3,5 HOUR** | **10 MIN** | **>10 HOUR** | **0,48** |
|  | **CATH1_CEC4** | **PVKNQGACGSCWTFSKKIGRVGQHTRDA** | **-0,26683** | **N-ALLERGEN** | **ALLERGEN** | **Non-Toxic** | **non-ACP** | **0,627** | **AVP** | **0,81** | **>20 HOUR** | **>20 HOUR** | **?** | **0,42** |
|  | **CATL1_CEC4** | **PVKNQGQCGSCWAFSKKIGRVGQHTRDA** | **-0,41743** | **N-ALLERGEN** | **N-ALLERGEN** | **Non-Toxic** | **non-ACP** | **0,424** | **AVP** | **0,98** | **>20 HOUR** | **>20 HOUR** | **?** | **0,42** |
|  | **CATH2_CEC4** | **YWIVKNSWGKKIGRVGQHTRDA** | **-0,16588** | **N-ALLERGEN** | **ALLERGEN** | **Non-Toxic** | **ACP** | **0,973** | **AVP** | **0,972** | **2,8 HOUR** | **10 MIN** | **2 MIN** | **0,43** |
|  | **CATL1.2_CEC4** | **YWLVKNSWGKKIGRVGQHTRDA** | **-0,21268** | **ALLERGEN** | **ALLERGEN** | **Non-Toxic** | **ACP** | **0,917** | **AVP** | **1** | **2,8 HOUR** | **10 MIN** | **2 MIN** | **0,45** |
|  | **CATK_CECA1** | **HAVLVVGYGKLGKKLEGAGKR** | **-0,40363** | **N-ALLERGEN** | **N-ALLERGEN** | **Non-Toxic** | **ACP** | **0,913** | **Non-AVP** | **0,002** | **3,5 HOUR** | **10 MIN** | **>10 HOUR** | **0,43** |
|  | **CATS_CECA1** | **HGVLVVGYGKLGKKLEGAGKR** | **-0,40363** | **N-ALLERGEN** | **N-ALLERGEN** | **Non-Toxic** | **ACP** | **0,943** | **AVP** | **0,64** | **3,5 HOUR** | **10 MIN** | **>10 HOUR** | **0,49** |
|  | **CATH1_CECA1** | **PVKNQGACGSCWTFSKLGKKLEGAGKR** | **-0,69358** | **N-ALLERGEN** | **N-ALLERGEN** | **Non-Toxic** | **ACP** | **0,881** | **AVP** | **0,854** | **>20 HOUR** | **>20 HOUR** | **?** | **0,51** |
|  | **CATL1_CECA1** | **PVKNQGQCGSCWAFSKLGKKLEGAGKR** | **-0,84418** | **N-ALLERGEN** | **N-ALLERGEN** | **Non-Toxic** | **ACP** | **0,812** | **Non-AVP** | **0,27** | **>20 HOUR** | **>20 HOUR** | **?** | **0,47** |
|  | **CATH2_CECA1** | **YWIVKNSWGKLGKKLEGAGKR** | **-0,59263** | **N-ALLERGEN** | **N-ALLERGEN** | **Non-Toxic** | **ACP** | **0,988** | **AVP** | **0,964** | **2,8 HOUR** | **10 MIN** | **2 MIN** | **0,47** |
|  | **CATL1.2_CECA1** | **YWLVKNSWGKLGKKLEGAGKR** | **-0,63943** | **ALLERGEN** | **N-ALLERGEN** | **Non-Toxic** | **ACP** | **0,959** | **AVP** | **0,994** | **2,8 HOUR** | **10 MIN** | **2 MIN** | **0,49** |
|  | **CATK_CECA2** | **HAVLVVGYGKIGKKIERVGQH** | **-0,04508** | **ALLERGEN** | **ALLERGEN** | **Non-Toxic** | **ACP** | **0,997** | **AVP** | **0,668** | **3,5 HOUR** | **10 MIN** | **>10 HOUR** | **0,47** |
|  | **CATS_CECA2** | **HGVLVVGYGKIGKKIERVGQH** | **-0,04508** | **N-ALLERGEN** | **N-ALLERGEN** | **Non-Toxic** | **ACP** | **0,997** | **AVP** | **0,998** | **3,5 HOUR** | **10 MIN** | **>10 HOUR** | **0,48** |
|  | **CATH1_CECA2** | **PVKNQGACGSCWTFSKIGKKIERVGQH** | **-0,33503** | **ALLERGEN** | **ALLERGEN** | **Non-Toxic** | **ACP** | **0,986** | **AVP** | **0,726** | **>20 HOUR** | **>20 HOUR** | **?** | **0,37** |
|  | **CATL1_CECA2** | **PVKNQGQCGSCWAFSKIGKKIERVGQH** | **-0,48563** | **ALLERGEN** | **ALLERGEN** | **Non-Toxic** | **ACP** | **0,973** | **AVP** | **0,954** | **>20 HOUR** | **>20 HOUR** | **?** | **0,4** |
|  | **CATH2_CECA2** | **YWIVKNSWGKIGKKIERVGQH** | **-0,23408** | **N-ALLERGEN** | **N-ALLERGEN** | **Non-Toxic** | **ACP** | **0,998** | **AVP** | **0,892** | **2,8 HOUR** | **10 MIN** | **2 MIN** | **0,45** |
|  | **CATL1.2_CECA2** | **YWLVKNSWGKIGKKIERVGQH** | **-0,28088** | **N-ALLERGEN** | **N-ALLERGEN** | **Non-Toxic** | **ACP** | **0,996** | **AVP** | **0,77** | **2,8 HOUR** | **10 MIN** | **2 MIN** | **0,45** |
|  | **CATK_CECA3** | **HAVLVVGYGKVFKKIEKVGRN** | **-0,21596** | **N-ALLERGEN** | **ALLERGEN** | **Non-Toxic** | **ACP** | **0,997** | **AVP** | **0,726** | **3,5 HOUR** | **10 MIN** | **>10 HOUR** | **0,47** |
|  | **CATS_CECA3** | **HGVLVVGYGKVFKKIEKVGRN** | **-0,21596** | **ALLERGEN** | **ALLERGEN** | **Non-Toxic** | **ACP** | **0,998** | **AVP** | **0,774** | **3,5 HOUR** | **10 MIN** | **>10 HOUR** | **0,48** |
|  | **CATH1_CECA3** | **PVKNQGACGSCWTFSKVFKKIEKVGRN** | **-0,50591** | **N-ALLERGEN** | **N-ALLERGEN** | **Non-Toxic** | **ACP** | **0,994** | **AVP** | **1** | **>20 HOUR** | **>20 HOUR** | **?** | **0,47** |
|  | **CATL1_CECA3** | **PVKNQGQCGSCWAFSKVFKKIEKVGRN** | **-0,65651** | **N-ALLERGEN** | **N-ALLERGEN** | **Non-Toxic** | **ACP** | **0,989** | **AVP** | **0,64** | **>20 HOUR** | **>20 HOUR** | **?** | **0,46** |
|  | **CATH2_CECA3** | **YWIVKNSWGKVFKKIEKVGRN** | **-0,40496** | **N-ALLERGEN** | **N-ALLERGEN** | **Non-Toxic** | **ACP** | **0,999** | **AVP** | **1** | **2,8 HOUR** | **10 MIN** | **2 MIN** | **0,5** |
|  | **CATL1.2_CECA3** | **YWLVKNSWGKVFKKIEKVGRN** | **-0,45176** | **N-ALLERGEN** | **N-ALLERGEN** | **Non-Toxic** | **ACP** | **0,997** | **AVP** | **0,98** | **2,8 HOUR** | **10 MIN** | **2 MIN** | **0,5** |
|  | **CATK_CECA4** | **HAVLVVGYGKFGKKLEGVGKR** | **-0,2768** | **ALLERGEN** | **N-ALLERGEN** | **Non-Toxic** | **ACP** | **0,98** | **AVP** | **0,81** | **3,5 HOUR** | **10 MIN** | **>10 HOUR** | **0,44** |
|  | **CATS_CECA4** | **HGVLVVGYGKFGKKLEGVGKR** | **-0,2768** | **N-ALLERGEN** | **ALLERGEN** | **Non-Toxic** | **ACP** | **0,985** | **Non-AVP** | **0,038** | **3,5 HOUR** | **10 MIN** | **>10 HOUR** | **0,49** |
|  | **CATH1_CECA4** | **PVKNQGACGSCWTFSKFGKKLEGVGKR** | **-0,56675** | **ALLERGEN** | **ALLERGEN** | **Non-Toxic** | **ACP** | **0,967** | **AVP** | **0,996** | **>20 HOUR** | **>20 HOUR** | **?** | **0,48** |
|  | **CATL1_CECA4** | **PVKNQGQCGSCWAFSKFGKKLEGVGKR** | **-0,71735** | **ALLERGEN** | **N-ALLERGEN** | **Non-Toxic** | **ACP** | **0,941** | **AVP** | **1** | **>20 HOUR** | **>20 HOUR** | **?** | **0,47** |
|  | **CATH2_CECA4** | **YWIVKNSWGKFGKKLEGVGKR** | **-0,4658** | **N-ALLERGEN** | **N-ALLERGEN** | **Non-Toxic** | **ACP** | **0,996** | **AVP** | **0,906** | **2,8 HOUR** | **10 MIN** | **2 MIN** | **0,47** |
|  | **CATL1.2_CECA4** | **YWLVKNSWGKFGKKLEGVGKR** | **-0,5126** | **N-ALLERGEN** | **ALLERGEN** | **Non-Toxic** | **ACP** | **0,986** | **AVP** | **0,972** | **2,8 HOUR** | **10 MIN** | **2 MIN** | **0,46** |
|  | **CATK_CECB1** | **HAVLVVGYGKLGKKIE** | **-0,35** | **N-ALLERGEN** | **N-ALLERGEN** | **Non-Toxic** | **ACP** | **0,986** | **AVP** | **0,588** | **3,5 HOUR** | **10 MIN** | **>10 HOUR** | **0,43** |
|  | **CATS_CECB1** | **HGVLVVGYGKLGKKIE** | **-0,35** | **ALLERGEN** | **N-ALLERGEN** | **Non-Toxic** | **ACP** | **0,991** | **Non-AVP** | **0,068** | **3,5 HOUR** | **10 MIN** | **>10 HOUR** | **0,49** |
|  | **CATH1_CECB1** | **PVKNQGACGSCWTFSKLGKKIE** | **-0,63995** | **N-ALLERGEN** | **ALLERGEN** | **Non-Toxic** | **ACP** | **0,972** | **Non-AVP** | **0,218** | **>20 HOUR** | **>20 HOUR** | **?** | **0,43** |
|  | **CATL1_CECB1** | **PVKNQGQCGSCWAFSKLGKKIE** | **-0,79055** | **ALLERGEN** | **N-ALLERGEN** | **Non-Toxic** | **ACP** | **0,941** | **AVP** | **0,676** | **>20 HOUR** | **>20 HOUR** | **?** | **0,41** |
|  | **CATH2_CECB1** | **YWIVKNSWGKLGKKIE** | **-0,539** | **N-ALLERGEN** | **N-ALLERGEN** | **Non-Toxic** | **ACP** | **0,998** | **AVP** | **1** | **2,8 HOUR** | **10 MIN** | **2 MIN** | **0,49** |
|  | **CATL1.2_CECB1** | **YWLVKNSWGKLGKKIE** | **-0,5858** | **ALLERGEN** | **N-ALLERGEN** | **Non-Toxic** | **ACP** | **0,993** | **AVP** | **0,892** | **2,8 HOUR** | **10 MIN** | **2 MIN** | **0,51** |
|  | **CATK_CECB2** | **HAVLVVGYGKIFKKIE** | **-0,1286** | **N-ALLERGEN** | **N-ALLERGEN** | **Non-Toxic** | **ACP** | **0,999** | **AVP** | **0,98** | **3,5 HOUR** | **10 MIN** | **>10 HOUR** | **0,46** |
|  | **CATS_CECB2** | **HGVLVVGYGKIFKKIE** | **-0,1286** | **N-ALLERGEN** | **ALLERGEN** | **Non-Toxic** | **ACP** | **1** | **AVP** | **0,64** | **3,5 HOUR** | **10 MIN** | **>10 HOUR** | **0,47** |
|  | **CATH1_CECB2** | **PVKNQGACGSCWTFSKIFKKIE** | **-0,41855** | **N-ALLERGEN** | **ALLERGEN** | **Non-Toxic** | **ACP** | **0,998** | **AVP** | **0,774** | **>20 HOUR** | **>20 HOUR** | **?** | **0,43** |
|  | **CATL1_CECB2** | **PVKNQGQCGSCWAFSKIFKKIE** | **-0,56915** | **ALLERGEN** | **N-ALLERGEN** | **Non-Toxic** | **ACP** | **0,995** | **AVP** | **0,544** | **>20 HOUR** | **>20 HOUR** | **?** | **0,43** |
|  | **CATH2_CECB2** | **YWIVKNSWGKIFKKIE** | **-0,3176** | **N-ALLERGEN** | **N-ALLERGEN** | **Non-Toxic** | **ACP** | **1** | **AVP** | **0,752** | **2,8 HOUR** | **10 MIN** | **2 MIN** | **0,49** |
|  | **CATL1.2_CECB2** | **YWLVKNSWGKIFKKIE** | **-0,3644** | **N-ALLERGEN** | **N-ALLERGEN** | **Non-Toxic** | **ACP** | **0,999** | **AVP** | **0,788** | **2,8 HOUR** | **10 MIN** | **2 MIN** | **0,5** |
|  | **CATK_CECB3** | **HAVLVVGYGKVFKKIE** | **-0,218** | **N-ALLERGEN** | **N-ALLERGEN** | **Non-Toxic** | **ACP** | **0,998** | **AVP** | **0,936** | **3,5 HOUR** | **10 MIN** | **>10 HOUR** | **0,48** |
|  | **CATS_CECB3** | **HGVLVVGYGKVFKKIE** | **-0,218** | **N-ALLERGEN** | **ALLERGEN** | **Non-Toxic** | **ACP** | **0,998** | **AVP** | **0,954** | **3,5 HOUR** | **10 MIN** | **>10 HOUR** | **0,49** |
|  | **CATH1_CECB3** | **PVKNQGACGSCWTFSKVFKKIE** | **-0,50795** | **N-ALLERGEN** | **ALLERGEN** | **Non-Toxic** | **ACP** | **0,994** | **AVP** | **0,966** | **>20 HOUR** | **>20 HOUR** | **?** | **0,47** |
|  | **CATL1_CECB3** | **PVKNQGQCGSCWAFSKVFKKIE** | **-0,65855** | **ALLERGEN** | **N-ALLERGEN** | **Non-Toxic** | **ACP** | **0,988** | **AVP** | **0,966** | **>20 HOUR** | **>20 HOUR** | **?** | **0,47** |
|  | **CATH2_CECB3** | **YWIVKNSWGKVFKKIE** | **-0,407** | **N-ALLERGEN** | **ALLERGEN** | **Non-Toxic** | **ACP** | **0,999** | **AVP** | **0,998** | **2,8 HOUR** | **10 MIN** | **2 MIN** | **0,5** |
|  | **CATL1.2_CECB3** | **YWLVKNSWGKVFKKIE** | **-0,4538** | **N-ALLERGEN** | **N-ALLERGEN** | **Non-Toxic** | **ACP** | **0,998** | **AVP** | **0,972** | **2,8 HOUR** | **10 MIN** | **2 MIN** | **0,51** |
|  | **CATK_CECC1** | **HAVLVVGYGKLGKRIERIGQ** | **0,20558** | **N-ALLERGEN** | **ALLERGEN** | **Non-Toxic** | **ACP** | **0,938** | **AVP** | **0,906** | **3,5 HOUR** | **10 MIN** | **>10 HOUR** | **0,51** |
|  | **CATS_CECC1** | **HGVLVVGYGKLGKRIERIGQ** | **0,20558** | **ALLERGEN** | **ALLERGEN** | **Non-Toxic** | **ACP** | **0,961** | **AVP** | **0,586** | **3,5 HOUR** | **10 MIN** | **>10 HOUR** | **0,5** |
|  | **CATH1_CECC1** | **PVKNQGACGSCWTFSKLGKRIERIGQ** | **-0,08437** | **N-ALLERGEN** | **N-ALLERGEN** | **Non-Toxic** | **ACP** | **0,821** | **AVP** | **1** | **>20 HOUR** | **>20 HOUR** | **?** | **0,3** |
|  | **CATL1_CECC1** | **PVKNQGQCGSCWAFSKLGKRIERIGQ** | **-0,23497** | **ALLERGEN** | **ALLERGEN** | **Non-Toxic** | **ACP** | **0,723** | **AVP** | **0,828** | **>20 HOUR** | **>20 HOUR** | **?** | **0,35** |
|  | **CATH2_CECC1** | **YWIVKNSWGKLGKRIERIGQ** | **0,01658** | **ALLERGEN** | **N-ALLERGEN** | **Non-Toxic** | **ACP** | **0,982** | **AVP** | **0,97** | **2,8 HOUR** | **10 MIN** | **2 MIN** | **0,42** |
|  | **CATL1.2_CECC1** | **YWLVKNSWGKLGKRIERIGQ** | **-0,03022** | **N-ALLERGEN** | **N-ALLERGEN** | **Non-Toxic** | **ACP** | **0,947** | **AVP** | **0,928** | **2,8 HOUR** | **10 MIN** | **2 MIN** | **0,44** |
|  | **CATK_CECC2** | **HAVLVVGYGKLGKKLEGAGK** | **-0,29134** | **N-ALLERGEN** | **N-ALLERGEN** | **Non-Toxic** | **ACP** | **0,967** | **AVP** | **0,876** | **3,5 HOUR** | **10 MIN** | **>10 HOUR** | **0,45** |
|  | **CATS_CECC2** | **HGVLVVGYGKLGKKLEGAGK** | **-0,29134** | **N-ALLERGEN** | **N-ALLERGEN** | **Non-Toxic** | **ACP** | **0,977** | **Non-AVP** | **0** | **3,5 HOUR** | **10 MIN** | **>10 HOUR** | **0,49** |
|  | **CATH1_CECC2** | **PVKNQGACGSCWTFSKLGKKLEGAGK** | **-0,58129** | **N-ALLERGEN** | **N-ALLERGEN** | **Non-Toxic** | **ACP** | **0,952** | **AVP** | **0,928** | **>20 HOUR** | **>20 HOUR** | **?** | **0,51** |
|  | **CATL1_CECC2** | **PVKNQGQCGSCWAFSKLGKKLEGAGK** | **-0,73189** | **N-ALLERGEN** | **N-ALLERGEN** | **Non-Toxic** | **ACP** | **0,916** | **AVP** | **0,678** | **>20 HOUR** | **>20 HOUR** | **?** | **0,47** |
|  | **CATH2_CECC2** | **YWIVKNSWGKLGKKLEGAGK** | **-0,48034** | **N-ALLERGEN** | **ALLERGEN** | **Non-Toxic** | **ACP** | **0,996** | **AVP** | **0,846** | **2,8 HOUR** | **10 MIN** | **2 MIN** | **0,49** |
|  | **CATL1.2_CECC2** | **YWLVKNSWGKLGKKLEGAGK** | **-0,52714** | **N-ALLERGEN** | **ALLERGEN** | **Non-Toxic** | **ACP** | **0,986** | **Non-AVP** | **0,106** | **2,8 HOUR** | **10 MIN** | **2 MIN** | **0,51** |
|  | **CATK_CECD1** | **HAVLVVGYGGQRIRDAIISAAPAV** | **0,54187** | **N-ALLERGEN** | **N-ALLERGEN** | **Non-Toxic** | **non-ACP** | **0,076** | **Non-AVP** | **0,354** | **3,5 HOUR** | **10 MIN** | **>10 HOUR** | **0,48** |
|  | **CATS_CECD1** | **HGVLVVGYGGQRIRDAIISAAPAV** | **0,54187** | **N-ALLERGEN** | **N-ALLERGEN** | **Non-Toxic** | **non-ACP** | **0,096** | **AVP** | **0,698** | **3,5 HOUR** | **10 MIN** | **>10 HOUR** | **0,49** |
|  | **CATH1_CECD1** | **PVKNQGACGSCWTFSGQRIRDAIISAAPAV** | **0,25192** | **N-ALLERGEN** | **N-ALLERGEN** | **Non-Toxic** | **non-ACP** | **0,025** | **AVP** | **0,994** | **>20 HOUR** | **>20 HOUR** | **?** | **0,47** |
|  | **CATL1_CECD1** | **PVKNQGQCGSCWAFSGQRIRDAIISAAPAV** | **0,10132** | **N-ALLERGEN** | **N-ALLERGEN** | **Non-Toxic** | **non-ACP** | **0,018** | **Non-AVP** | **0,008** | **>20 HOUR** | **>20 HOUR** | **?** | **0,47** |
|  | **CATH2_CECD1** | **YWIVKNSWGGQRIRDAIISAAPAV** | **0,35287** | **N-ALLERGEN** | **ALLERGEN** | **Non-Toxic** | **non-ACP** | **0,269** | **Non-AVP** | **0,022** | **2,8 HOUR** | **10 MIN** | **2 MIN** | **0,48** |
|  | **CATL1.2_CECD1** | **YWLVKNSWGGQRIRDAIISAAPAV** | **0,30607** | **N-ALLERGEN** | **ALLERGEN** | **Non-Toxic** | **non-ACP** | **0,081** | **AVP** | **0,678** | **2,8 HOUR** | **10 MIN** | **2 MIN** | **0,49** |
|  | **CATK_CECD2** | **HAVLVVGYGGQRVRDAVISAGPAV** | **0,35814** | **N-ALLERGEN** | **ALLERGEN** | **Non-Toxic** | **non-ACP** | **0,05** | **Non-AVP** | **0,294** | **3,5 HOUR** | **10 MIN** | **>10 HOUR** | **0,49** |
|  | **CATS_CECD2** | **HGVLVVGYGGQRVRDAVISAGPAV** | **0,35814** | **N-ALLERGEN** | **ALLERGEN** | **Non-Toxic** | **non-ACP** | **0,072** | **Non-AVP** | **0,004** | **3,5 HOUR** | **10 MIN** | **>10 HOUR** | **0,49** |
|  | **CATH1_CECD2** | **PVKNQGACGSCWTFSGQRVRDAVISAGPAV** | **0,06819** | **N-ALLERGEN** | **N-ALLERGEN** | **Non-Toxic** | **non-ACP** | **0,009** | **AVP** | **0,994** | **>20 HOUR** | **>20 HOUR** | **?** | **0,49** |
|  | **CATL1_CECD2** | **PVKNQGQCGSCWAFSGQRVRDAVISAGPAV** | **-0,08241** | **N-ALLERGEN** | **ALLERGEN** | **Non-Toxic** | **non-ACP** | **0,007** | **Non-AVP** | **0,47** | **>20 HOUR** | **>20 HOUR** | **?** | **0,48** |
|  | **CATH2_CECD2** | **YWIVKNSWGGQRVRDAVISAGPAV** | **0,16914** | **N-ALLERGEN** | **ALLERGEN** | **Non-Toxic** | **non-ACP** | **0,108** | **Non-AVP** | **0,398** | **2,8 HOUR** | **10 MIN** | **2 MIN** | **0,5** |
|  | **CATL1.2_CECD2** | **YWLVKNSWGGQRVRDAVISAGPAV** | **0,12234** | **N-ALLERGEN** | **ALLERGEN** | **Non-Toxic** | **non-ACP** | **0,034** | **AVP** | **0,81** | **2,8 HOUR** | **10 MIN** | **2 MIN** | **0,51** |
|  | **CATK_CECD3** | **HAVLVVGYGGQRVRDAIISAGPAV** | **0,44754** | **N-ALLERGEN** | **N-ALLERGEN** | **Non-Toxic** | **non-ACP** | **0,06** | **Non-AVP** | **0,43** | **3,5 HOUR** | **10 MIN** | **>10 HOUR** | **0,49** |
|  | **CATS_CECD3** | **HGVLVVGYGGQRVRDAIISAGPAV** | **0,44754** | **N-ALLERGEN** | **N-ALLERGEN** | **Non-Toxic** | **non-ACP** | **0,087** | **Non-AVP** | **0,43** | **3,5 HOUR** | **10 MIN** | **>10 HOUR** | **0,5** |
|  | **CATH1_CECD3** | **PVKNQGACGSCWTFSGQRVRDAIISAGPAV** | **0,15759** | **N-ALLERGEN** | **N-ALLERGEN** | **Non-Toxic** | **non-ACP** | **0,015** | **AVP** | **0,676** | **>20 HOUR** | **>20 HOUR** | **?** | **0,48** |
|  | **CATL1_CECD3** | **PVKNQGQCGSCWAFSGQRVRDAIISAGPAV** | **0,00699** | **N-ALLERGEN** | **N-ALLERGEN** | **Non-Toxic** | **non-ACP** | **0,01** | **Non-AVP** | **0,398** | **>20 HOUR** | **>20 HOUR** | **?** | **0,47** |
|  | **CATH2_CECD3** | **YWIVKNSWGGQRVRDAIISAGPAV** | **0,25854** | **N-ALLERGEN** | **ALLERGEN** | **Non-Toxic** | **non-ACP** | **0,18** | **Non-AVP** | **0,122** | **2,8 HOUR** | **10 MIN** | **2 MIN** | **0,49** |
|  | **CATL1.2_CECD3** | **YWLVKNSWGGQRVRDAIISAGPAV** | **0,21174** | **N-ALLERGEN** | **ALLERGEN** | **Non-Toxic** | **non-ACP** | **0,053** | **AVP** | **1** | **2,8 HOUR** | **10 MIN** | **2 MIN** | **0,51** |
|  | **CATL1.2_CECB1_2** | **WLVKNSWGKLGKKI** | **-0,61920** | **ALLERGEN** | **N-ALLERGEN** | **Non-Toxic** | **ACP** | **0,999** | **AVP** | **0,97** | **2,8 HOUR** | **3 MIN** | **2 MIN** | **0,51** |
| **Parameters** |  |  | **-** | **-** | **-** | **-** | **-** | **<0.5: low probability >0.5: high probability** | **-** | **<0.5: low probability >0.5: high probability** | **ND: not determined** | | | **>0.5: likely hemolytic <0.5: unlikely hemolytic** |

**Table S6 - Synthesized peptides**

|  |  |  |  | **C2 Pred** | | **CAMP** | **AMPA** | | **AmpGram** | **PepCalc** | **PROTPARAM** | | **APD3** | **HELIQUEST** | | **TMHMM 2** | | **ANTICANCER - ACPred** | | **ANTIVIRAL - Meta-iAVP** | | **HALF LIFE** | | | **HemoPI** |
| --- | --- | --- | --- | --- | --- | --- | --- | --- | --- | --- | --- | --- | --- | --- | --- | --- | --- | --- | --- | --- | --- | --- | --- | --- | --- |
| **NAME** | **HYBRID PEPTIDE** | **MW (g/mol)** | **N° aa** | **CPP prediction** | | **AMP prediction** | | |  | **SOLUBILITY IN WATER** | **INSTABILITY** | **GRAVY** | **BOMAN (kcal/mol)** | **Hydrophobicity** | **HYDROPHOBIC MOMENT µH** | **Cellular Localization** | **Total probability of N-in by TMHMM server** | **Prediction** | **ACP** | **Prediction** | **AVP** | ***In vitro*** | **In vivo - YEAST** | **In vivo - E. coli** |  |
| CATL1.2_CECB2 | YWLVKNSWGKIFKKIE | 2039,45 | 16 | CPP | 0,589472 | 0,8155 | - | None | 9.969 | GOOD | 4,47 | -0,444 | 0,73 | 0,533 | 0,497 | inside | 0,57 | ACP | 0,999 | AVP | 0,788 | 2,8 HOUR | 10 MIN | 2 MIN | 0,50 |
| CECB1_CATL1.2 | KLGKKIEYWLVKNSWG | 1949,33 | 16 | N-CPP | 0,492165 | 0,855 |  |  | 5.406 | GOOD | 13,87 | -0,688 | 0,86 | 0,415 | 0,209 | inside | 0,48 | ACP | 0,991 | AVP | 0,892 | 1,3 HOUR | 3 MIN | 3 MIN | 0,51 |
| CATL1.2_CECB1_2 | WLVKNSWGKLGKKI | 1657,03 | 14 | N-CPP | 0,427371 | 0,99 | - | None | 1 | GOOD | -15,62 | -0,443 | 0,49 | 0,451 | 0,435 | inside | 0,38 | ACP | 0,999 | AVP | 0,97 | 2,8 HOUR | 3 MIN | 2 MIN | 0,51 |

**Table S7 - ADMET of selected hybrid peptides**

| **Peptides** | | **Absorption** | | **Distribution** | | | **Metabolism** | | | | **Excretion** | | **Toxicity** | | | | |
| --- | --- | --- | --- | --- | --- | --- | --- | --- | --- | --- | --- | --- | --- | --- | --- | --- | --- |
| **NAME** | **HYBRID PEPTIDE** | **HIA (%)** | **Caco-2**  **Permeability (cm/s)** | **VD (L/Kg)** | **BBB Penetration (%)** | **PPB(%)** | **CYP1A2-inhibitor** | **CYP1A2-substrate** | **CYP3A4-inhibitor** | **CYP3A4-substrate** | **CL**  **(mL/min/Kg)** | **Half life** | **hERG blockers** | **DILI**  **Liver Injury** | **AMES** | **Carcinogenicity** | **Skin sensitization** |
| **CATL1.2_CECB2** | YWLVKNSWGKIFKKIE | 1 | -7662 | -0,184 | 0,004 | 34,34 | 0 | 0 | 0 | 0 | -2,098 | 0,937 | 0 | 0 | 0,28 | 0,009 | 0,051 |
| **CECB1_CATL1.2** | KLGKKIEYWLVKNSWG | 1 | -7,59 | -0,037 | 0,009 | 31,64 | 0 | 0 | 0 | 0 | -2,576 | 0,907 | 0 | 0 | 0,01 | 0,004 | 0,005 |
| **CATL1.2_CECB1_2** | WLVKNSWGKLGKKI | 0,986 | -6,372 | 0,48 | 0,023 | 24,62 | 0 | 0 | 0,084 | 0 | 1,076 | 0,92 | 0,016 | 0 | 0,009 | 0,041 | 0,131 |
| **Parameters** | | HIA > 0.3: HIA positive HIA < 0.3: HIA negative | Optimal: higher than -5.15 Log unit | Optimal: 0.04-20L/kg | Category 1: BBB+; Category 0: BBB-; The output | Optimal: < 90%. Drugs with high protein-bound | Category 1: Inhibitor; Category 0: Non-inhibitor; | Category 1: Substrate; Category 0: Non-substrate; | Category 1: Inhibitor; Category 0: Non-inhibitor; | Category 1: Substrate; Category 0: Non-substrate; | High: >15 mL/min/kg; moderate: 5-15 mL/min/kg; | Category 1: Long half-life; Category 0: Short half-life | Category 1: active; Category 0: inactive; | Category 1: drugs with a high risk of DILI; Category | Category 1: Ames positive(+); Category 0: Ames negative (-) | Category 1: carcinogens; Category 0: non-carcinogens; | Category 1: sensitizer; Category 0: non-sensitizer; |

**S8 – Table of Protein targets of CATL1.2_CECB1_2 and CECB1_CATL1.2 peptides ranked by highest normalized fit score values and originating from the microorganism E. coli. PM Rank (Overall rank by PharmMapper), PDB ID (Identification code in the Protein Database), Normalized fit score (value obtained by the ratio between the fit score and the number of features).XXXXX**

| **CATL1.2_CECB1_2** | | | |
| --- | --- | --- | --- |
| **PM Rank** | **PDB ID** | **Normalized fit score** | **Target name** |
| 1 | 1AOB | 0,9655 | Thymidylate synthase |
| 13 | 1EI1 | 0,9209 | DNA gyrase B |
| **CECB1_CATL1.2** | | | |
| **PM Rank** | **PDB ID** | **Normalized fit score** | **Target name** |
| 1 | 1YR3 | 0,9809 | Xanthosine phosphorylase |
| 16 | 1USQ | 0,948 | Dr hemagglutinin |
| 21 | 1EI1 | 0,9385 | DNA gyrase B |
| 23 | 1WEI | 0,9347 | A/G-specific DNA glycosylase |
| 48 | 1A99 | 0,8993 | Periplasmic putrescine-binding protein |

1. Below the water signal [↑](#footnote-ref-1)
2. Below the solvent signal [↑](#footnote-ref-2)
3. Below the solvent signal [↑](#footnote-ref-3)
